# Trifluoromethylbismuth dications: pronounced parallel substrate activation, Lewis superacidity, and catalytic application

**DOI:** 10.1039/d6sc04400j

**Published:** 2026-08-19

**Authors:** Ahmed Fetoh, Heba Youssef, Hendrik Kilian, Bernhard Roling, Crispin Lichtenberg

**Affiliations:** a Department of Chemistry, Philipps-University Marburg Hans-Meerwein-Str. 4 35032 Marburg Germany crispin.lichtenberg@chemie.uni-marburg.de; b Department of Chemistry, Faculty of Science, Mansoura University, El Gomhouria Mansoura Qism 2, Dakahlia Governorate 11432 Mansoura Egypt; c mar.quest|Marburg Center for Quantum Materials and Sustainable Technologies 35032 Marburg Germany

## Abstract

Understanding and exploiting the multiple facets of Lewis acidity represents a topical challenge in molecular chemistry. Here we present the synthesis of well-defined bismuth compounds bearing the electron-withdrawing CF_3_ group as a key structural feature. Most importantly, a range of dicationic species [Bi(CF_3_)(L)_*n*_]^2+^ could be isolated and characterized, the charge being balanced by different weakly coordinating anions (L = neutral ligand). The dicationic species are highly Lewis acidic (culminating in Lewis superacidity) and show the unusual ability to strongly activate two soft Lewis bases in parallel. The strong Lewis acidity was translated into a remarkable catalytic activity in the reduction of phosphane oxides.

## Abstract 2

Compounds featuring the [Bi(CF_3_)(L)_*n*_]^2+^ ions have been investigated (L = neutral ligand). The dications are exceptionally strong Lewis acids, engage in parallel substrate activation, and are highly active catalysts in phosphane oxide reduction.

## Graphical abstract

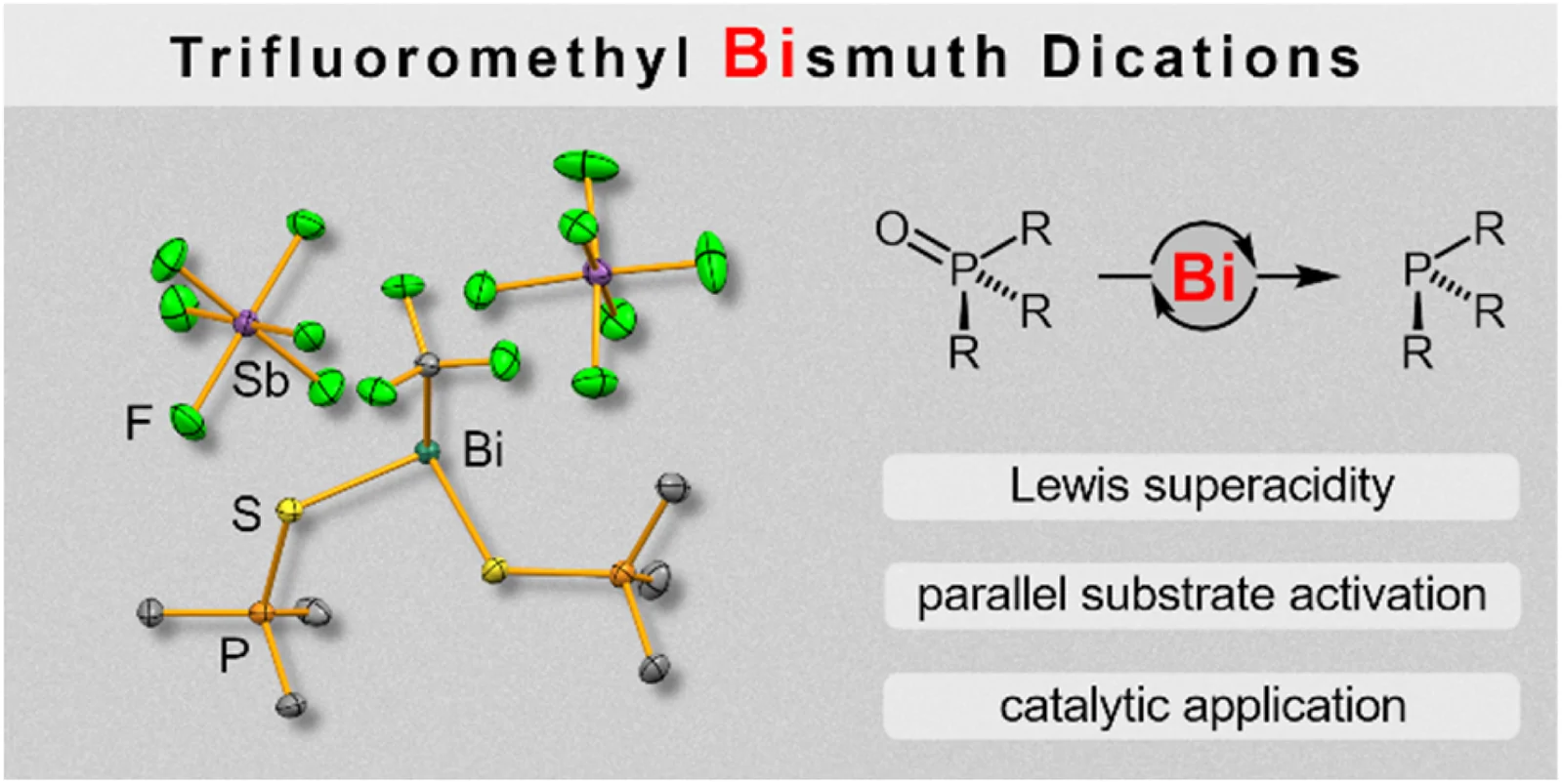

The conversion of neutral bismuth precursors into cationic species has yielded compounds with remarkable structures and reactivity.^1–3^ This includes low-coordinate and low-valent complexes (*e.g.*, [Bi(2,6-Mes_2_C_6_H_3_)_2_][B(3,5-(CF_3_)_2_-C_6_H_3_)_4_] and [Bi(cAAC)_2_][OTf]),^3,4^ unusual coordination geometries (*e.g.*, pentagonal (bi)pyramids),^5–7^ ring-strained entities,^8–10^ and novel motifs such as bisma-alkenes and Bi → Bi donor–acceptor interactions.^11,12^ These unique structural properties give rise to unprecedented reactivity patterns, including C–H activation,^8–10^ CO insertion,^13^ reversible olefin activation,^14^ and reversible one-electron transfer.^15^ In the catalytic regime, remarkable transformations have been realized, such as the C–H trifluoromethylation of (hetero)arenes,^16^ amide reduction,^17^ controlled radical polymerization of α-olefins,^18^ cationic polymerization of oxiranes and a vinyl ether,^19^ the hydrosilylation of olefins and carbonyl compounds,^20,21^ the reduction of ketones and phosphine oxides,^22^ allylic C–H activation,^23^ and the fluorination and triflation of arylboronic esters and acids *via* redox catalysis.^24^ Consequently, these findings have motivated a deeper exploration of Lewis acidity in bismuth chemistry. This research has uncovered the unique ability of bismuth(iii) centers to adopt formal charges ranging from +1 to +3,^3,12,20,22,25,26^ to present multiple Lewis acidic sites for reversible substrate binding,^20,27^ and to tunably function as moderately hard or pronouncedly soft Lewis acids.^5,28,29^ Furthermore, factors such as inverse solvent effects (where increased solvent polarity enhances Lewis acidity) have been reported.^6^ One key motivation remains to develop strategies for the amplification of Lewis acidity. Along these lines, the modulation of the (group-)electronegativity of bismuth-bound ligands has been identified as a tool to control Lewis acidity.^27,28,30^ Furthermore, sophisticated ligand design involving chelating ligands with controlled bite angles can exploit geometric factors to modify Lewis acidity.^27,30–32^ Counterintuitively, a higher steric bulk can also be used to increase the Lewis acidity of bismuth compounds by reducing the number of accessible Lewis acidic sites to one.^25^ Since cationic bismuth species tend to show higher Lewis acidities than their neutral parent compounds,^28^ dicationic species were an obvious target in order to further increase their Lewis acidic properties. With strong relevance to this work, for instance, the dication [BiMe(thf)_5_]^2+^ shows a significantly higher Lewis acidity than its monocationic congener [BiMe_2_]^+^ despite the presence of five neutral ligands.^7,11^ In order to push the limits of bismuth-based Lewis acidity, we aimed to combine the dicationic nature of well-defined molecular entities with a high (group-) electronegativity of the anionic supporting ligand. Importantly a sufficient solubility of the dicationic species in moderately polar solvents must be ensured in order to prevent quenching of the Lewis acidity by solvent molecules. In view of this profile of requirements, the incorporation of trifluoromethyl groups^33–37^ into dicationic bismuth compounds was identified as a promising strategy.

Here we present the synthesis, isolation, and characterization of a range of complexes containing the [Bi(CF_3_)]^2+^ building block, stabilized by varying combinations of labile neutral ligands and weakly coordinating counteranions, along with the evaluation of their Lewis acidity.

The precursor Bi(CF_3_)Ph_2_ (1) was required as a starting point for our studies. The literature-approach to this compound required the utilization of the organocadmium reagent Cd(CF_3_)_2_, long reaction times of 14 days, and a fractional distillation to give a moderate 58% yield of the target compound.^37^ A literature synthesis of the related compound Bi(C_2_F_5_)Ph_2_ required the lithiation of HC_2_F_5_,^34^ and this approach would thus require the handling of highly reactive and potentially explosive LiCF_3_,^38^ when targeting compound 1. We found that treatment of the readily available BiPh_2_Cl with two equivalents of CsF and four equivalents of commercially available TMS–CF_3_ in THF overnight afforded the precursor Bi(CF_3_)Ph_2_ in 85% isolated yield after crystallization (Scheme 1). The compound was for the first time characterized in detail by NMR spectroscopy, elemental analysis, and single-crystal XRD (SI). The ^19^F NMR spectrum of Bi(CF_3_)Ph_2_ in CH_3_CN-*d*_3_ showed a singlet at −38.8 ppm, consistent with the reported signal for the CF_3_ group in Ph_2_BiCF_3_ (singlet, ^19^F NMR: *δ* = −38.6 ppm)^36,37^ and closely matching the signal for CF_3_ in ^*t*^BuN(CH_2_C_6_H_2_)_2_Bi(CF_3_) (singlet, *δ*(^19^F) = −38.8 ppm).^39^ Subsequent protonolysis of BiPh_2_(CF_3_) with hydrogen chloride gave Bi(CF_3_)Cl_2_ (2) in 89% yield, which has previously been detected by ^19^F NMR spectroscopy as an intermediate *en route* to Bi(CF_3_)_3_.^36^ Adduct formation with pyridine gave [Bi(CF_3_)Cl_2_(py)_2_] (2-py), which was fully characterized and shows a square pyramidal coordination geometry with the CF_3_ group in the apical position and the Cl atoms in a *trans* configuration (Scheme 1). In addition, weak intermolecular Bi⋯Cl interactions are observed based on distance criteria (SI). Reactions of 2 with different silver salts of weakly coordinating anions (WCAs) yield distinct bismuth cations in simple salt elimination reactions (Scheme 1), demonstrating varying coordination and chemical properties.

**Scheme 1:**
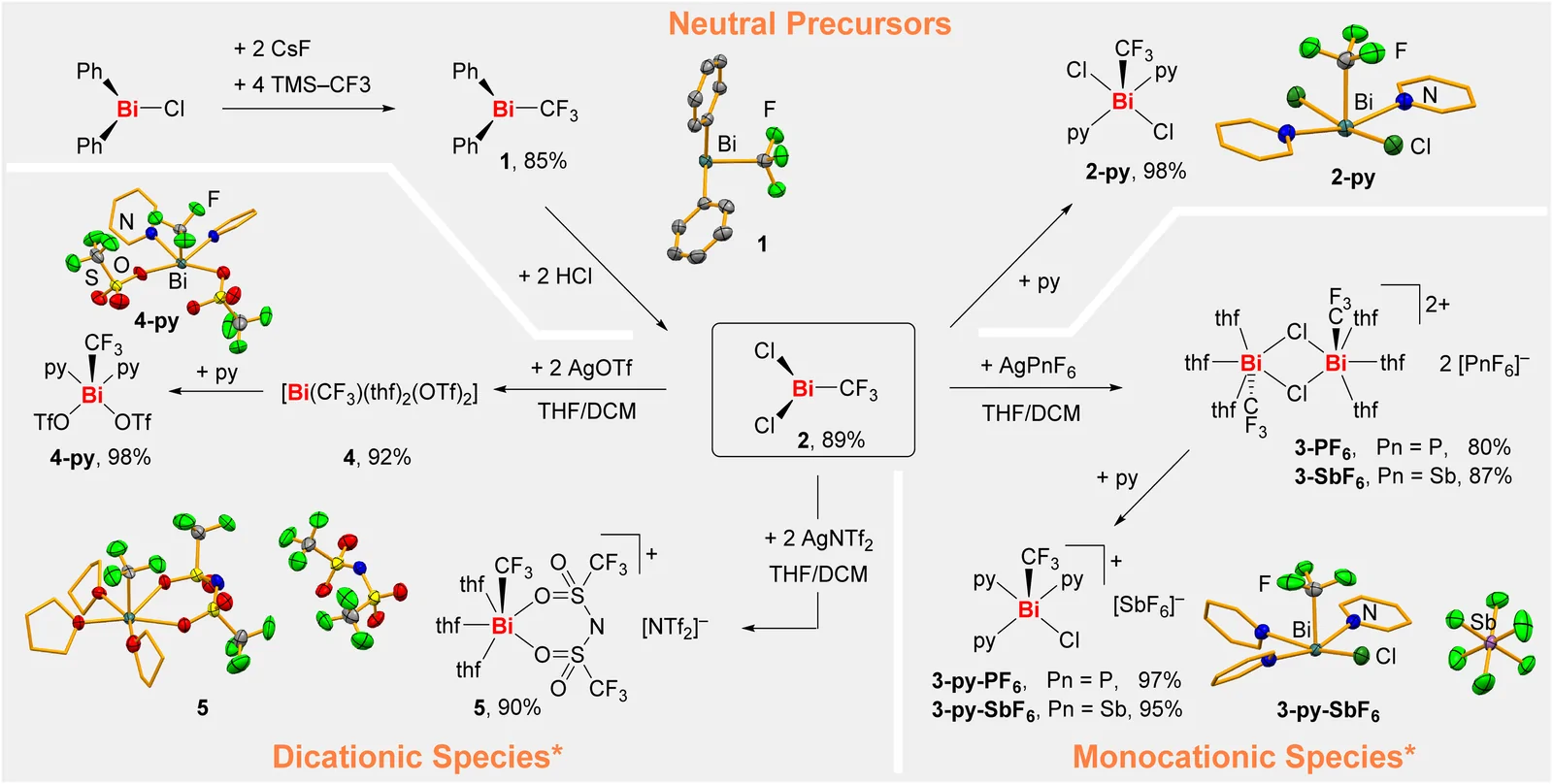
Synthesis of trifluoromethyl-substituted bismuth compound. In graphical representations of results of single-crystal X-ray diffraction analyses, displacement ellipsoids are shown at the 50% probability level (H atoms are omitted and C atoms of netural ligands are shown as wireframe for clarity). *: The number of weakly coordinating anions (PF_6_^−^, SbF_6_^−^, OTf^−^, NTf_2_^−^) per bismuth atom is used for the classification (neutral *vs.* monocationic *vs.* dicationic). Selected bonding parameters are presented in the text, details are presented in the SI.

For example, reactions with silver hexafluorophosphate (AgPF_6_) and silver hexafluoroantimonate (AgSbF_6_) consistently produce [Bi(CF_3_)µ_2_-Cl(thf)_3_]_2_(WCA)_2_ (WCA = PF_6_ (3-PF_6_), SbF_6_ (3-SbF_6_)), irrespective of the Bi : WCA ratio. These compounds could be isolated in high yields of 80–87%. The ^19^F NMR spectrum of 3-SbF_6_ indicates the presence of free (SbF_6_)^−^ anions in a solution of acetonitrile (SI). Very weak Bi⋯FSbF_5_ interactions may be assigned in the solid state based on distance criteria (as judged from single-crystal X-ray diffraction (sc-XRD) analysis), as the shortest interatomic Bi⋯F distance (3.166(9) Å) is somewhat smaller than the sum of the van-der-Waals radii (3.54 Å).^40^ When not taking these weak interactions into account, the bismuth atoms in 3-SbF_6_ are six-coordinate due to interactions of each bismuth atom with one CF_3_ group, three terminally-bound thf ligands and two µ_2_-bridging chlorido ligands. This creates a twofold positive charge for the dimeric arrangement [Bi_2_(CF_3_)_2_(µ_2_-Cl)_2_(thf)_6_]^2+^ and a pentagonal pyramidal coordination geometry around the bismuth atom with the CF_3_ group in the apical position (angle sum O/Cl–Bi–O/Cl, 360.2°).

Treatment of compound 3-PnF_6_ (Pn = P, Sb) with pyridine, followed by slow diffusion of pentane into the solution, afforded the corresponding pyridine adducts, [Bi(CF_3_)Cl(py)_3_][PnF_6_] (3-py-PnF_6_), which are found as mononuclear species due to the stronger σ-donor character of pyridine, as confirmed by sc-XRD analysis (triclinic space group *P*1̄, *Z* = 2, Scheme 1 and SI). The bismuth atoms in compound 3-py-PnF_6_ are five-coordinate due to interactions of each bismuth atom with one CF_3_ group in the apical position and three pyridine ligands and one chlorido ligand in the basal plane of a square pyramid as the coordination polyhedron. In addition, fluoride atoms of the [PnF_6_]^−^ counteranions have long contacts with the Bi atoms with a distance ranging from 3.147(2) to 3.490(2) Å.

In contrast, the reaction of 2 with two equivalents of silver triflate (AgOTf) or silver triflimide (AgNTf_2_) furnishes the dicationic species [Bi(CF_3_)(thf)_2_(OTf)_2_] (4), its pyridine-ligated derivative [Bi(CF_3_)(py)_2_(OTf)_2_] (4-py), and [BiCF_3_(thf)_3_NTf_2_](NTf_2_) (5) (Scheme 1). Compound 4 forms a tetranuclear arrangement in the solid state with triflate ions in terminal as well as bridging µ_3_-coordination modes (SI). Compounds 4-py and 5 show a square pyramidal and pentagonal pyramidal coordination geometry, respectively, with the CF_3_ group in the apical position. Both OTf^−^ ligands coordinate to the central atom in a *cis* configuration in 4-py, while only one NTf_2_^−^ group acts as a chelating ligand with primary bonding interactions to the bismuth atom in 5. Weak bonding interactions with additional OTf^−^/NTf_2_^−^ ligands are observed in the solid state in *trans*-position of the CF_3_ groups (Bi–O 2.481(4)–2.500(5) in 4-py; 2.967(3)–3.114(3) Å in 5) with Bi–O distances below the sum of the van der Waals radii (3.59 Å).^40^^19^F NMR spectra of compounds 4 and 4-py show two singlets at *δ* = −40.36/36.00 and −78.54/–78.84 ppm with a 3 : 6 integral ratio due to the CF_3_ and the OTf^−^ groups, respectively. This is in agreement with the presence of ionic triflate in solution (*δ* = −78.3 to −79.2 ppm for ionic triflate *vs. δ* = −76.3 ppm for MeOTf with covalent bonding interactions),^41,42^ as confirmed by conductivity measurements for selected compounds (SI). The observation of solvent-separated ion pairs in solution is consistent with other known bismuth, antimony, and arsenic ionic complexes, with triflates bearing similar ^19^F NMR shifts (−78.3 to −79.1 ppm) and weak interactions in the solid state attributed to electrostatic interactions.^42,43^ Similarly, ^19^F NMR spectra of 5 show two singlets at *δ* = −39.80 and −79.24 ppm due to the CF_3_ and the NTf_2_^−^ groups, respectively, with relative integrals of 3 : 12, indicating equivalence and thus rapid exchange of NTf_2_^−^ groups on the time scale of the spectroscopic experiment.

As a general observation, the presence of a polar solvent was found to be necessary in order to guarantee the efficacy of reactions between Bi(CF_3_)Cl_2_ and various silver salts. However, these reactions could not be conducted in pure THF, as the solvent polymerized, forming an insoluble solid that precluded characterization. A DCM/THF mixture (10 : 1 (v/v)) was found to provide conditions to sufficiently suppress polymerization, while maintaining the coordination of THF.

The Lewis acidity of pnictenium ions is a key factor in their reactivity, with stronger acidity commonly leading to greater reactivity.^44^ Current challenges in the field include (i) the creation of highly Lewis acidic compounds (ii) with a sufficient solubility in weakly-coordinating solvents, (iii) the ability to activate soft substrates without fostering unselective decomposition pathways, and (iv) the efficient activation of two substrates in close proximity to each other. We identified 3-SbF_6_, 4, and 5 as promising candidates to address some of these challenges. Indeed, DFT calculations on Bi(CF_3_)(OTf)_2_ at the B3LYP/def2-TZVP level of theory revealed an electrostatic potential (ESP) with a well-accessible, positively charged region at the bismuth atom (Fig. 1a). A molecular orbital analysis indicated that the LUMO and the LUMO+1 each show main contributions by one *p*(Bi) atomic orbital, are situated in the positively charged region identified in the ESP analysis, and should be suitable to realize binding interactions with two Lewis basic substrate molecules (Fig. 1b). Compared to the non-fluorinated analogue BiMe(OTf)_2_, the LUMO and LUMO+1 are lower in energy by 0.4 eV each, demonstrating a significant impact of the CF_3_ group on the energies of these MOs.

**Fig. 1:**
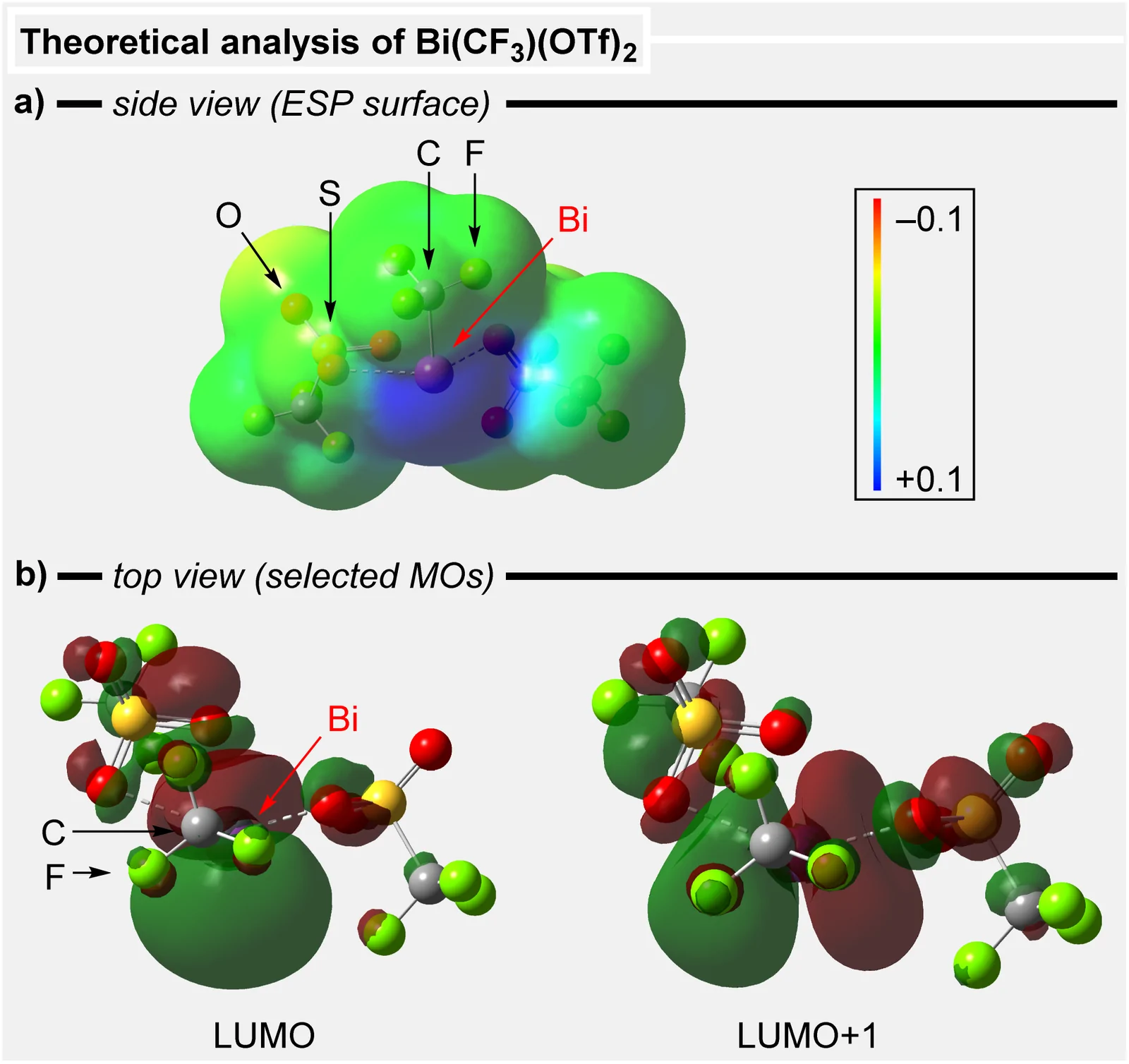
DFT calculations on Bi(CF_3_)(OTf)_2_: (a) electron density from total SCF density (isovalue = 0.0004) mapped with ESP. (b) LUMO and LUMO+1 (isovalue = 0.02).

Thus, we set out to experimentally evaluate the Lewis acidity of compounds 3-SbF_6_, 4, and 5, using the (modified) Gutmann–Beckett (GB) method.^29,45,46^ In this method, varying amounts of the spectroscopic probes OPEt_3_, SPMe_3_, or SePMe_3_ (showing an increasingly soft character according to the Pearson concept) are added to the Lewis acid under investigation.^2,29,45,46^ The ^31^P NMR spectroscopic chemical shifts are then translated into acceptor numbers (ANs), high acceptor numbers correlating with a high Lewis acidity. All compounds 3-SbF_6_, 4, and 5 show remarkably high ANs towards all three probes (Table 1). In fact, they can compete with the most Lewis acidic bismuth compounds towards the donor OPEt_3_ and outperform all other literature-known bismuth complexes in terms of Lewis acidity towards the softer donors SPMe_3_ and SePMe_3_.^5,7,9,20,22,25,28–32^ Using 4 as an example, the acceptor numbers in the presence of one or two equivalents of SPMe_3_ or SePMe_3_ range between 99 and 106, which clearly outperforms the recently reported non-fluorinated analog [BiMe]^2+^ (values of corresponding ANs: 76–94).^7^ This demonstrates that the combination of a high (twofold) positive charge of the complex ion with a high group electronegativity of the ligand scaffold adds up to pronounced Lewis acidity of bismuth compounds towards soft Lewis bases, while maintaining a sufficient solubility in the weakly-coordinating solvent DCM. Adducts of 3-SbF_6_, 4, and 5 with two or more equivalents of SPMe_3_ or SePMe_3_ were isolated and fully characterized (*vide infra* and SI). ^31^P NMR spectroscopic analyses of the isolated compounds confirmed the *in situ* determined ANs that are presented in Table 1. Single crystal X-ray analyses demonstrated a close proximity of the activated probe molecules SPMe_3_ or SePMe_3_ to each other, as they are consistently found in a *cis* configuration in the coordination sphere of the Lewis acidic bismuth center. For instance, compounds [Bi(CF_3_)(OTf)_2_(SPMe_3_)_2_] (6) and [Bi(CF_3_)(OTf)_2_(SePMe_3_)_2_] (7) were isolated and fully characterized from reactions of 4 with two equivalents of SPMe_3_ or SePMe_3_, respectively (Fig. 2a and b, for details and further examples, see SI). Aiming at a compound with a more weakly coordinating anion than OTf^−^, compound 2 was reacted with 2 equiv. of SPMe_3_ and 2 equiv. of AgSbF_6_, which afforded [Bi(CF_3_)(SPMe_3_)_2_][SbF_6_]_2_ (8) after workup. A full characterization confirmed the *cis*-orientation of the SePMe_3_ ligands (Fig. 2c) and revealed a record-breaking AN(SPMe_3_) of 111.^47^ This goes along with a substantial elongation of the P–S bond from 1.9694(6) Å in free SPMe_3_ ^29^ to 2.0466(14)–2.0514(13) Å in 8. The same synthetic approach with 4 equiv. of SPMe_3_ gave compound [Bi(CF_3_)(SPMe_3_)_4_][SbF_6_]_2_ (9), still yielding a remarkably high AN(SPMe_3_) of 88 despite the presence of four SPMe_3_ ligands (Fig. 2d). The efficient activation of the EPMe_3_ ligands in **6–9** is mirrored by relatively short Bi–S bonds (6: 2.661(2)–2.671(2) Å; 8: 2.5877(10)–2.6220(9); 9: 2.7576(11)–2.8382(10) Å) and Bi–Se bonds (7: 2.804(10)–2.805(6) Å).^48^ In addition, the S–P (6: 2.024(3)–2.031(3) Å; 8: 2.0466(14)–2.0514(13) Å; 9: 2.0114(16)–2.0135(16) Å) and Se–P bonds (7: 2.154(13)–2.188(6) Å) are significantly longer than those in the non-coordinated molecules (P–S, 1.9694(6) Å; P–Se, 2.1229(11) Å).^29^ For the structural characterization of further adducts between Lewis acidic bismuth compounds and EPMe_3_ (*E* = S, Se), see the SI.

**Table 1:** Acceptor numbers (ANs) for compounds [Bi(CF_3_)µ_2_-Cl(thf)_3_]_2_(SbF_6_)_2_ (3-SbF_6_), [Bi(CF_3_)(thf)_2_(OTf)_2_] (4), and [BiCF_3_(thf)_3_NTf_2_](NTf_2_) (5) according to the (modified) Gutmann–Beckett method in CD_2_Cl_2_

Equiv. of EPR_3_	AN(EPR_3_) for compound 3-SbF_6_| 4 | 5		
	AN(OPEt_3_)	AN(SPMe_3_)	AN(SePMe_3_)
1.0	90 | 88 | 87	97 | **106 | 105**	96 | **104 | 105**
1.5	86 | 87 | 77	96 | **104 | 104**	95 | **101 | 103**
2.0	79 | 85 | 73	88 | **102 | 100**	88 | **99 | 97**
3.0	70 | 71 | 71	70 | 81 | 83	71 | 77 | 82
4.0	72 | 66 | 70	64 | 68 | 73	64 | 65 | 79

**Fig. 2:**
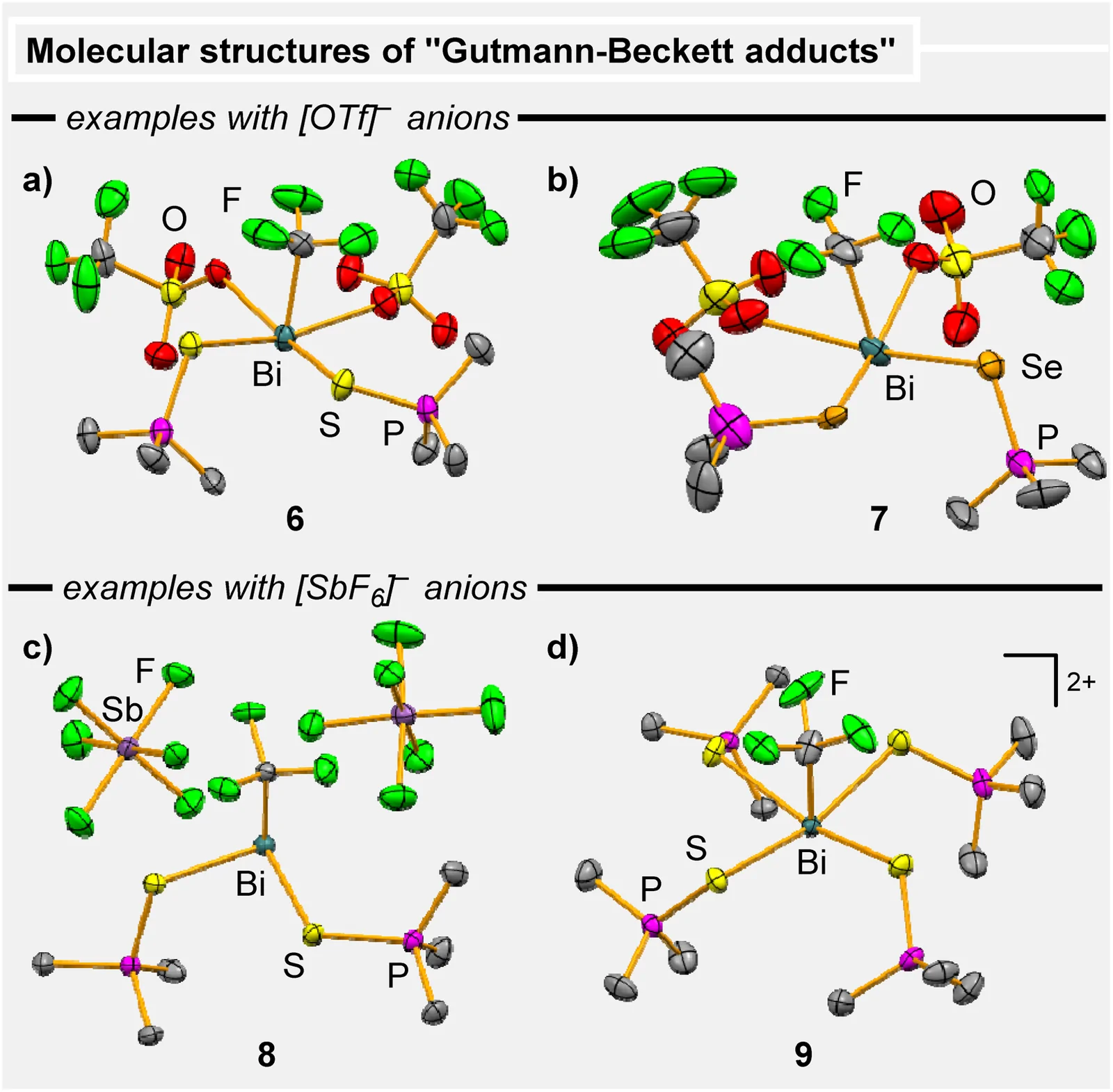
Molecular structures in the solid state of (a) [Bi(CF_3_)(OTf)_2_(SPMe_3_)_2_] (6), (b) [Bi(CF_3_)(OTf)_2_(SePMe_3_)_2_] (7), (c) [Bi(CF_3_)(SPMe_3_)_2_][SbF_6_]_2_ (8), and (d) [Bi(CF_3_)(SPMe_3_)_4_][SbF_6_]_2_ (9), as determined by single-crystal X-ray diffraction (for details, see SI). Displacement ellipsoids are shown at the 50% probability level. H atoms, lattice-bound solvent molecules (and in the case of 9 also the [SbF_6_]^−^ counter anions) are omitted for clarity.

The outstanding Lewis acidity of the [Bi(CF_3_)]^2+^ complex cation motivated further investigation of its reactivity. Reaction of BiCF_3_Cl_2_ with 1 equiv. 2,2′-bipyridine (bipy) and 2 equiv. AgSbF_6_ resulted in the formation of [Bi(CF_3_)µ_2_-F(bipy)]_2_[SbF_6_]_2_ (10), which was isolated in 74% yield and structurally characterized as its pyridine adduct 10-py (Scheme 2a).^49^ Compound 10 is formed *via* fluoride ion abstraction by the assumed intermediate [Bi(CF_3_)(bipy)]^2+^ from one of its [SbF_6_]^−^ counteranions, lending Lewis superacidic properties to this species.^50^ This is confirmed by the calculation of the fluoride ion affinity (FIA) of [Bi(CF_3_)(bipy)]^2+^ (Scheme 2b). The values for the dication are expectedly extremely high in the gas phase, which is without practical relevance for these species to date. However, they still outperform the benchmark compound SbF_5_, when solvent models are applied, confirming experimental findings. In polar solvents such as THF and DCM, the formation of a dinuclear aggregate is necessary to drive the reaction, as judged from FIA values, which is in agreement with experimental findings. When compared to the related non-fluorinated species [BiMe(phenanthroline)_2_]^2+^,^7^ the fluorinated species presented here brings about two major advances: the extraordinary FIA relates to one fluoride ion per bismuth (not to one fluoride ion per two bismuth atoms) and the reactions can be performed and monitored in the liquid phase (while fluoride ion transfer was realized during precipitation in the former case).

**Scheme 2:**
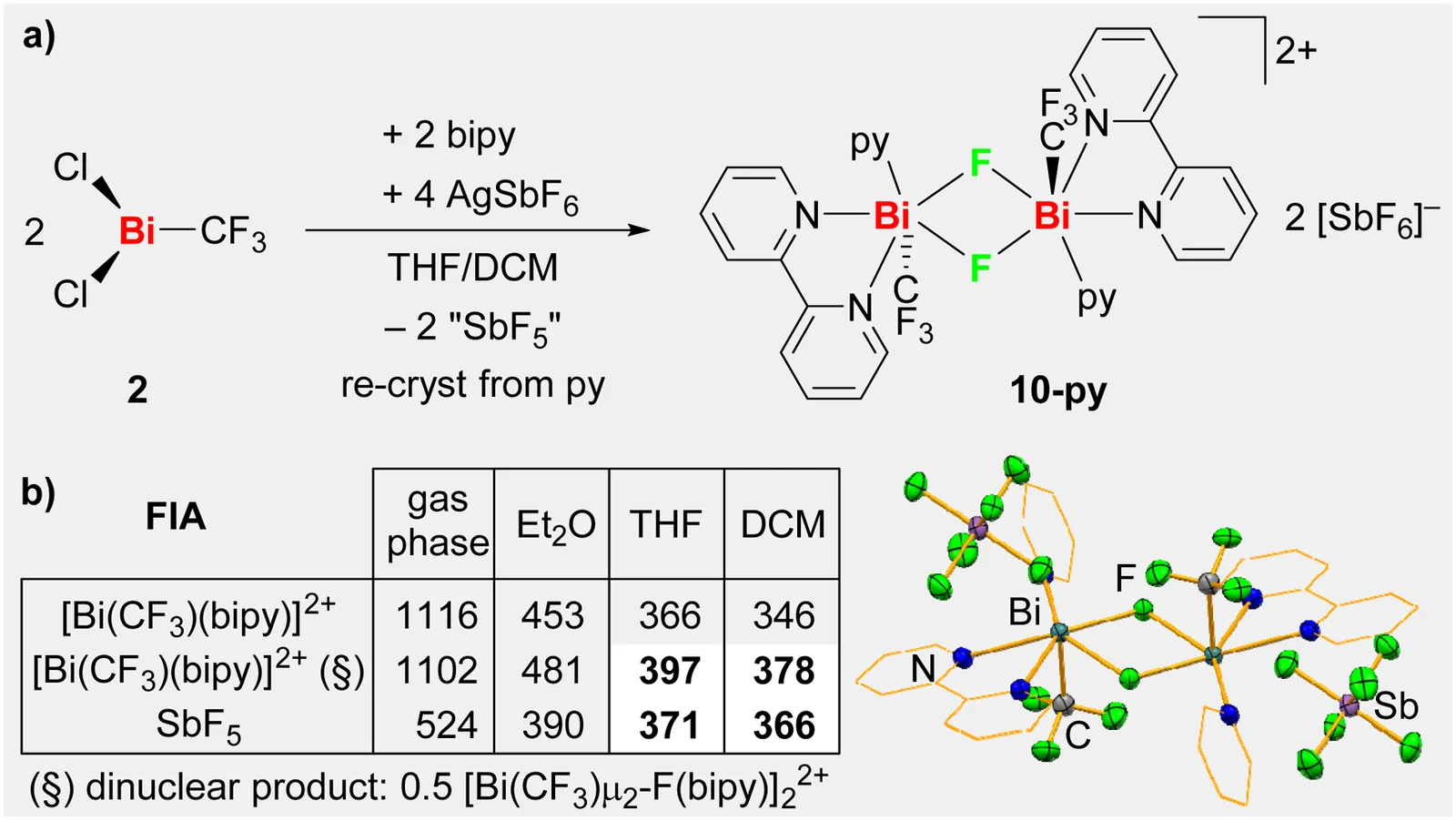
(a) Fluoride ion abstraction from [SbF_6_]^−^ by suggested intermediate [Bi(CF_s_)(bipy)]^2+^ to give 9 (displacement ellipsoids are shown at the 50% probability level, hydrogen atoms are omitted and carbon atoms of neutral ligands are shown in the wireframe model for clarity; for details, see SI). (b) Fluoride ion affinity (FIA) of selected compounds in kJ mol^−1^ calculated at the B3LYP/def2-tzvp level of theory (solvent model: PCM), using SiMe_3_F as an anchor point (for details, see SI).

The high Lewis acidity, as indicated by the high ANs compared to those of other reported bismuth cations, prompted us to probe if this characteristic can be translated into a catalytic application. Organophosphorus compounds are indispensable reagents for many important reactions such as the Wittig reaction, the Mitsunobu reaction, the Appel reaction or the Staudinger reaction, but they produce stoichiometric amounts of phosphane oxide waste. While recycling this waste *via* reduction is highly desirable, it can be challenging due to the exceptional strength of the phosphorus–oxygen bond. Therefore, a broad range of methods for the reduction of phosphane oxides to phosphanes has been developed, including catalyzed transformations.^51^ In addition to transition metal catalyzed reactions (*e.g.*: Ti(O*i*Pr),^52–55^ Cu(OTf)_2_),^56^ methodologies based on main group catalysts have also been developed using isodesmic reactions or Lewis acids as key approaches.^57^

Main group catalysts for phosphane oxide reduction include [(β-diketiminato)Mg(pinBB(*n*-Bu)pin)],^58^ B(C_6_F_5_)_3_, [(C_6_F_5_)_3_PF][B(C_6_F_5_)_4_], [(SIMes)PFPh_2_][B(C_6_F_5_)_4_]_2_ (SIMes = C_3_H_4_(NC_6_H_2_Me_3_)_2_),^59^ Me_3_SiOTf,^60^ (Mes)_2_SbOTf,^61^ and (Mes)_2_BiOTf^61^ have been shown to enable the deoxygenation of phosphane oxides in the presence of reducing agents. Such catalytic reactions are typically performed either at elevated temperatures using relatively high catalyst loadings of 5–10 mol%. With the substantial Lewis acidity and robustness of the dicationic CF_3_-decorated bismuth compounds in mind, we set out to test compound 4 as a catalyst for the reduction of phosphane oxides with phenyl silane.

Indeed, a smooth reduction of a variety of phosphane oxides to the corresponding phosphanes could be achieved with 2 mol% of catalyst 4 at 60 °C (Table 2). Hydrogen, H_2_, and the disiloxane (PhH_2_Si)_2_O were detected as by-products of the reaction, using ^1^H and ^29^Si NMR spectroscopy. The substrate scope covers compounds with alkyl substituents (P1, P2), aryl groups (P3) and mixed alkyl/aryl substitution patterns (P4). Polar functional groups (NH_2_ in P5) and olefins (P7) are accommodated without problems. In addition to tertiary phosphanes, secondary phosphanes are converted with even shorter reaction times (P6, P8). Diaryl(chloro)phosphane oxides give the corresponding secondary phosphanes (P6). A dinuclear phosphane oxide was reduced to the respective dinuclear phosphane with extended reaction times (P9). The catalyst loading could be lowered to 0.5 mol% with reactions times remaining short (90 min), when generating P6 as a target compound that was chosen exemplarily.

**Table 2:** Reduction of phosphane oxides to phosphanes catalyzed by compound 4. Yields were determined by ^31^P NMR spectroscopy (see SI). No conversion is observed in the absence of compound 4

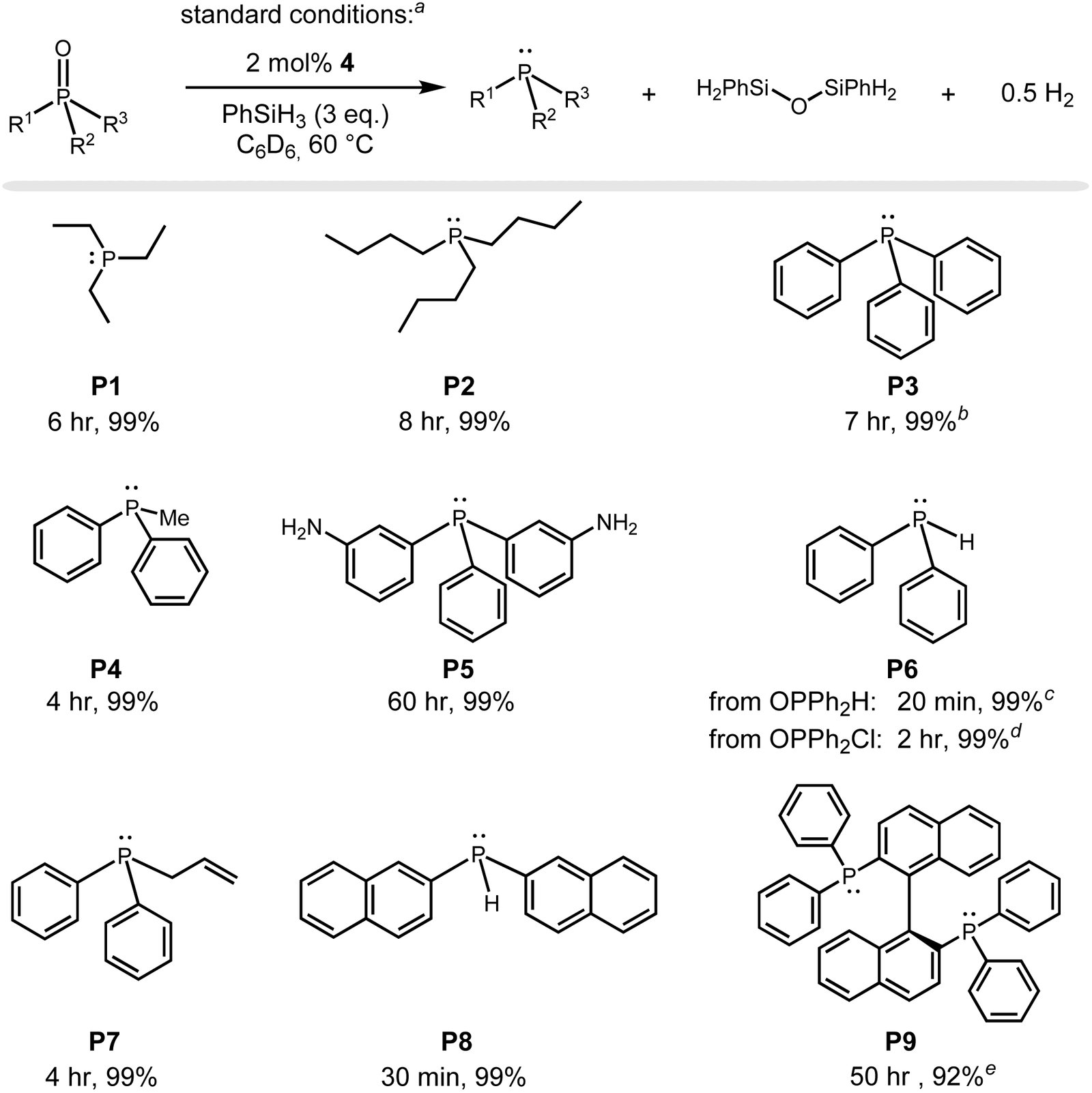

a Additional results obtained with deviations from standard conditions are given in footnotes *b*–*e*.

b When using [Bi(CH_3_)(OTf)_2_(thf)_2_] or Bi(OTf)_3_ instead of compound 4, 44% and 48% conversion is achieved under the same conditions.

c Full conversion is achieved in 30 min or 90 min, when lowering the catalyst loading to 1 mol% and 0.5 mol%, respectively.

d When using CDCl_2_ or CD_3_CN as the solvent, full conversion was obtained after 3 hours.

e Full conversion is achieved in 40 hours using 10 mol% of compound 4.

When compared to literature-known approaches, catalyst 4 requires lower catalyst loadings,^52,54–56,60,62^ lower reaction temperatures^52–56,59,62^ or shorter reaction times,^56,59,60,62^ offering advantages for the future development of this reaction.^51^ This includes both, protocols based on transition metal catalysts such as Ti(O*i*Pr)^52–55^ and (Cu(OTf)_2_)^56^ or main group compounds. Even problematic cases such as P9 that have made stoichiometric approaches necessary in previous reports,^60^ could be obtained catalytically with 4 as the catalyst. A closer comparison with cationic antimony and bismuth species as catalysts for phosphane oxide reduction may be worthwhile. With compounds (Mes)_2_SbOTf or (Mes)_2_BiOTf, a catalyst loading of 10 mol% was necessary to achieve 92–97% conversion of Ph_3_PO over 12–30 hours in the presence of PhSiH_3_.^61^ This demonstrates that the exceptional Lewis acidity of 4 can be translated into an effective catalytic procedure, outperforming a vast range of existing approaches in the catalyzed phosphane oxide reduction.

In conclusion we present the synthesis, isolation, and characterization of the first series of dicationic bismuth compounds with the strongly electron-withdrawing CF_3_ group, stabilized by substitutionally labile neutral ligands. The [Bi(CF_3_)(L)_*n*_]^2+^ complex fragment effectively activates two to four substrate molecules in parallel at one metal center (L = neutral ligand/substrate). This explicitly includes soft Lewis bases, as demonstrated by record-high acceptor numbers towards the probes SPMe_3_ and SePMe_3_ determined with the modified Gutmann–Beckett method. The *in situ* generated species [Bi(CF_3_)(bipy)]^2+^ cleanly abstracts a fluoride ion from [SbF_6_]^−^, indicating its Lewis superacidic properties. The ability of trifluoromethyl-substituted bismuth dications to very efficiently activate two or more substrates in parallel at one metal center in close proximity to each other (including soft Lewis bases) promises a rich potential for catalytic applications invoking Lewis acids in key steps. The extraordinary Lewis acidity of these compounds could be translated into a remarkably high catalytic activity in the reduction of phosphane oxides with phenyl silane to give the corresponding phosphanes with a good functional group tolerance.

## Author contributions

A. F. conceived the work, designed the experiments, performed the experiments, collected the experimental data, and wrote the manuscript. A. F. and H. Y. performed the crystallographic analyses. H. K. and B. R. performed and discussed the conductivity measurements. C. L. directed the project, received funding for the project, conducted the DFT calculations, co-designed the experiments, and co-wrote the manuscript. All authors discussed the results and finalized the manuscript and have given approval to the final version of the manuscript.

## Conflicts of interest

There are no conflicts to declare.

## Supplementary Material

SC-017-D6SC04400J-s001

SC-017-D6SC04400J-s002

## Data Availability

The data supporting this article have been included as part of the supplementary information (SI). Supplementary information is available. See DOI: https://doi.org/10.1039/d6sc04400j. CCDC 2546515 (1), 2546516 (2), 2546517 (2-Py-SbF_6_), 2546518 (3-Py-SbF_6_), 2546519 (3-SbF_6_), 2546520 (4), 2546521 (4-py), 2546522 (5), 2546523 (6), 2546524 (7), 2546525 (8), 2546526 (9), 2546527 (10-py), 2546528 (11), 2546529 (12), 2546530 (13), 2575824 ([Bi(CF_3_)(thf)(NTf_2_)_2_(bipy)]) and 2575825 ([Bi(CF_3_)(thf)(NTf_2_)_2_(phen)]) contain the supplementary crystallographic data for this paper.^63*a*–*r*^
