# Supplementary material for: Trifluoromethylbismuth dications: pronounced parallel substrate activation, Lewis superacidity, and catalytic application
Source: Chem Sci. 2026 Aug 19;17(41):19914–21. doi: 10.1039/d6sc04400j (PMC13551294; doi:10.1039/d6sc04400j)
Supplement: Supplementary file 1 [file SC-017-D6SC04400J-s001.pdf]

Supplementary Information (SI) for Chemical Science.
This journal is © The Royal Society of Chemistry 2026

Supporting Information for

Trifluoromethylbismuth dications: pronounced parallel substrate activation, Lewis superacidity, and catalytic application

Ahmed Fetoh[a,b,c] Heba Youssef,[a,b] Hendrik Kilian,[a,c] Bernhard Roling,[a,c] and Crispin Lichtenberg*[a,c]

[a] Dr. A. Fetoh, Dr. H. Youssef, H. Kilian, Prof. Dr. B. Roling, Prof. Dr. C. Lichtenberg
Department of Chemistry, Philipps-University Marburg, Hans-Meerwein-Str. 4, 35032 Marburg, Germany
E-mail: crispin.lichtenberg@chemie.uni-marburg.de

[b] Dr. A. Fetoh, Dr. H. Youssef
Department of Chemistry, Faculty of Science, Mansoura University, El Gomhouria, Mansoura Qism 2, Dakahlia Governorate 11432 Mansoura, Egypt

[c] Dr. A. Fetoh, H. Kilian, Prof. Dr. B. Roling, Prof. Dr. C. Lichtenberg
mar.quest | Marburg Center for Quantum Materials and Sustainable Technologies, 35032 Marburg, Germany.

Contents

1. **Experimental** S3
   - **Preparation of $Bi(CF_3)Ph_2$ (1)** S3
   - **Preparation of $[Bi(CF_3)Cl_2]$ (2)** S4
   - **Preparation of $[Bi(CF_3)\mu_2\text{-}Cl(thf)_3]_2[PF_6]_2$ (3-PF_6)** S5
   - **Preparation of $[BiCF_3Cl(py)_3][PF_6]$ (3-py-PF_6)** S5
   - **Preparation of $[Bi(CF_3)\mu_2\text{-}Cl(thf)_3]_2(SbF_6)_2$ (3-SbF_6)** S6
   - **Preparation of $[BiCF_3(py)_3][SbF_6]$ (3-py-SbF_6)** S6
   - **Preparation of $[Bi(CF_3)(OTf)_2(thf)_2]$ (4)** S7
   - **Preparation of $[Bi(CF_3)(OTf)_2(py)_2]$ (4-py)** S8
   - **Preparation of $[Bi(CF_3)(thf)_3(NTf_2)][NTf_2]$ (5)** S9
   - **Preparation of $[Bi(CF_3)(OTf)_2(SPMe_3)_2]$ (6)** S9
   - **Preparation of $[Bi(CF_3)(OTf)_2(SePMe_3)_2]$ (7)** S10
   - **Preparation of $[BiCF_3(SPMe_3)_2][SbF_6]_2$ (8)** S10
   - **Preparation of $[BiCF_3(SPMe_3)_4][SbF_6]_2$ (9)** S11
   - **Preparation of $[Bi(thf)(CF_3)\mu_2\text{-}F(bipy)]_2[SbF_6]_2$ (10)** S12
   - **Preparation of $[Bi_2(CF_3)_2(\mu_2\text{-}Cl)(\mu_2\text{-}SePMe_3)(SePMe_3)_4][SbF_6]_3$ (11)** S13
   - **Preparation of $[Bi(CF_3)\mu_2\text{-}Cl(SePMe_3)_2]_2[SbF_6]_2$ (12)** S14
   - **Preparation of $[Bi(CF_3)(SePMe_3)_4][NTf_2]_2$ (13)** S16
2. **Discussion of the crystal structures of compounds $Bi(CF_3)Ph_2$ and $[Bi(CF_3)Cl_2(py)_2]$** S18
3. **Determination of acceptor numbers by the (modified) Gutmann-Beckett method** S18
4. **Synthesis and characterization of $Bi(CF_3)^{2+}$ adducts with EPR_3 (E= S, Se; R=Me)** S19
5. **Additional adducts** S21
   - **Preparation of $[Bi(CF_3)(thf)(NTf_2)_2(bipy)]$** S21
   - **Preparation of $[Bi(CF_3)(thf)(NTf_2)_2(phen)]$** S22
6. **NMR Spectra** S24
7. **Catalysis** S60
8. **Single-crystal X-ray diffraction** S77
9. **DFT calculations** S86
10. **Conductivity measurements** S90
11. **References** S92

1. Experimental

General remarks

All Experiments were, if not stated otherwise, conducted under an atmosphere of dry argon using Schlenk- and glovebox techniques. Solvents were degassed and purified according to standard laboratory procedures. NMR spectra were recorded on Bruker Avance spectrometers operating at 300, 400 or 500 MHz with respect to ^{1}H. ^{1}H- and ^{13}C-NMR chemical shifts are reported relative to $SiMe_4$ using the residual signal of the deuterated solvent as a secondary standard.[1] ^{19}F-, ^{31}P-, and ^{77}Se-NMR chemical shifts are reported relative to $CFCl_3$, aqueous H_3PO_4 (85%), and $SeMe_2$ (plus 5 % C_6D_6), respectively, as external standards. NMR spectra were recorded at ambient temperature (typically 23 °C), if not otherwise noted. Elemental analyses were performed on a vario MICRO cube, and the results are given in %. Single-crystals suitable for X-ray diffraction were coated with polyisobutylene or perfluorinated polyether oil in a glovebox, transferred to a nylon loop and then transferred to the goniometer of a Bruker D8 Quest diffractometer or a Bruker D8 Venture diffractometer. Both diffractometers are equipped with a molybdenum (λ = 0.71073 Å) X-Ray tube. Using ShelXle[2] and Olex2,[3] the structure was solved with the XT structure solution program using intrinsic phasing and refined with the XL refinement package using least squares minimization.[4] The structure was solved and refined using the Bruker SHELX 2014 software package. All non-hydrogen atoms were refined with anisotropic displacement parameters. All hydrogen atoms were refined isotropically on calculated positions by using a riding model with their U_{iso} values constrained to 1.5 U_{eq} of their pivot atoms for terminal sp^3 carbon atoms and 1.2 for all other atoms. Crystallographic data have been deposited with the Cambridge Crystallographic Data Centre as supplementary publication numbers 2546515-2546530. These data can be obtained free of charge from The Cambridge Crystallographic Data Centre and Fachinformationszentrum Karlsruhe Access Structures service www.ccdc.cam.ac.uk/structures.

Preparation of $Bi(CF_3)Ph_2$ (1)

To a stirred solution of Ph_2BiCl (1.00 g, 2.52 mmol) in THF (15 mL), CsF (760 mg, 5.00 mmol) was added, followed by addition of $TMSCF_3$ (1.47 ml, 10.0 mmol). After 1 hour, $TMSCF_3$ (1.47 ml, 10.0 mmol) was added a second time. The reaction mixture was stirred overnight.

Complete conversion was confirmed by ^{1}H and ^{19}F NMR spectroscopy. The THF solution was then filtered through Celite, and the solvent was removed *in vacuo*. The remaining residue was extracted with DCM (3 × 10 mL), which was then removed under vacuum to yield the final product. The residue was dissolved in a minimum volume of *n*-pentane (0.8 mL), and the product was crystallized at −30 °C. The obtained solid was washed with minimal amounts of pre-cold *n*-pentane (2 × 0.1 mL) and dried *in vacuo*, yielding colorless crystals. The product can be further purified by sublimation at 100 °C and 10^{-3} mbar onto a cold finger that is kept at −80 °C. Yield: 916 mg, 2.12 mmol, 84.5%.

^{1}H NMR (300 MHz, CH_3CN-d_3): δ = 7.44 (m, 2H, H_{para}), 7.60 (t, ^{3}J(H,H) = 7.5 Hz, ^{3}J(H,H) = 5.1 Hz, 4H, H_{meta}), 8.02 ppm (d, ^{3}J(H,H) = 7.8 Hz, ^{4}J(H,H) = 1.8 Hz, 4H, H_{ortho}).

^{13}C NMR (300 MHz, CH_3CN-d_3): δ = 126.57 (s, C_{para}), 132.00 (s, C_{meta}), 138.81 (s, C_{ortho}, C_{ipso}), 164.22 (q, ^{1}J(F,C) = 389 Hz, CF_3) ppm.

^{19}F NMR (282.31 MHz, THF-d_8): δ = −39.21 (s, CF_3) ppm.

^{19}F NMR (282.31 MHz, CH_3CN-d_3): δ = −38.69 (s, CF_3) ppm.

Elemental analysis (%; a re-sublimed sample was analyzed), Anal. Calc. for [$C_{13}H_{10}BiF_3$] (432.20 g mol^{-1}): C 36.13, H 2.33; found C 36.40, H 2.44.

Preparation of [Bi(CF_3)Cl_2] (2)

A solution of hydrogen chloride in diethyl ether (0.61 mL, 2.31 mmol, 3.82 M) was added to a stirred solution of CF_3BiPh_2 (500 mg, 1.16 mmol) in dichloromethane (5 mL) at 0 °C over 3 hours. All volatile compounds were removed under vacuum. The residue was then washed with pentane (2 × 2 mL) and dried *in vacuo*, yielding pure [Bi(CF_3)Cl_2] as a yellow crystalline material. Yield: 360 mg, 1.03 mmol, 89%. Single crystals of **2** were obtained by the slow diffusion of pentane into a saturated solution of [Bi(CF_3)Cl_2] in pyridine at −30 °C after 2 days. The white precipitate was filtered, washed with pentane (3 × 2 mL) and dried *in vacuo*, yielding pure [Bi(CF_3)Cl_2(py)$_2$] as a white crystalline material. Yield: 513 mg, 1.03 mmol, 98%.

^{13}C NMR (300 MHz, THF-d_8): δ = 160.89 (q, ^{1}J(F,C) = 373 Hz, CF_3) ppm.

^{19}F NMR (300 MHz, THF-d_8): δ = −43.66 (s, CF_3) ppm.

^{19}F NMR (300 MHz, CH_2Cl_2-d_2): δ = −42.56 (s, CF_3) ppm.

^{19}F NMR (282.31 MHz, CH_3CN-d_3): $\delta = -41.71$ (s, CF_3) ppm.

^{19}F NMR (300 MHz, Pyridine-d_5): $\delta = -40.63$ (s, CF_3) ppm.

Elemental analysis (%; a sample re-crystallized from pyridine/pentane was analyzed): Anal. Calc. for [$C_1Bi_1Cl_2F_3$(pyridine)$_2$] (507.09 g mol^{-1}): C 26.05, H 1.99, N 5.52; found C 25.85, H 2.18, N 5.62.

Preparation of [$Bi(CF_3)\mu_2$-Cl(thf)$_3$]$_2$[PF_6]$_2$ (3-PF$_6$)

A solution of $AgPF_6$ (73 mg, 0.286 mmol) in DCM (1 mL) was added to a solution of $Bi(CF_3)Cl_2$ (100 mg, 0.286 mmol) in a mixture of both THF and DCM (0.1:1 mL). The precipitate was filtered off, and all volatiles were removed from the filtrate under reduced pressure, yielding a colorless powder that was dried *in vacuo*. Yield: 155 mg, 0.115 mmol, 80%. Single crystals suitable for X-ray diffraction analysis of **3-PF$_6$** were obtained by layering a solution of **3-PF$_6$** in pyridine with *n*-pentane and storage at –30 °C for 16 h.

Elemental analysis (%; a sample re-crystallized from reaction mixture by layering with *n*-pentane was analyzed): Anal. Calc. for [$C_2Bi_2Cl_2F_{18}P_2$(thf)$_5$] (1277.34 g mol^{-1}): C 20.69, H 3.16; found C 20.99, H 3.36.

^{1}H NMR (300 MHz, CD_2Cl_2): $\delta = 4.15$ (m, 24H, O-CH_2), 2.06 (m, 24H, C-CH_2) ppm.

^{13}C NMR (125 MHz, CD_2Cl_2): $\delta = 183.92$ (q, ^{1}J(F,C) = 377 Hz, CF_3) ppm.

^{19}F NMR (300 MHz, CH_2Cl_2): $\delta = -39.56$ (s, CF_3), −69.16 (d, ^{1}J(P,F) = 711.5 Hz, PF_6) ppm.

^{31}P NMR (122 MHz, CH_2Cl_2): $\delta = -143.94$ (sept, ^{1}J(P,F) = 715.1 Hz, PF_6) ppm.

Preparation of [$BiCF_3Cl(py)_3$][PF_6] (3-py-PF$_6$)

Compound **3-PF$_6$** (40 mg, 0.030 mmol) was dissolved in DCM (0.5 mL), and pyridine (14 mg, 0.178 mmol, 16 µL) was added. The colorless solution was layered with *n*-pentane (2 mL). After 1 day at –30 °C a colorless crystalline solid precipitated, was isolated by filtration, and dried *in vacuo*. Yield: 40 mg, 0.058 mmol, 97%.

^{1}H NMR (300 MHz, CD_3CN): $\delta = 7.63$ (t, ^{3}J(H,H) = 7.1 Hz, 6H, H2/H6), 8.07 (t, ^{3}J(H,H) = 7.7 Hz, 3H, H4), 8.76 (d, ^{3}J(H,H) = 4.6 Hz, 6H, H3/H5) ppm.

^{13}C NMR (125 MHz, CD_3CN): δ = 126.50 (s, C2/C6), 141.27 (s, C4), 148.27 (s, C3/C5), 204.70 (q, ^{1}J(F,C) = 363 Hz, CF_3) ppm.

^{19}F NMR (300 MHz, CD_3CN): δ = −41.95 (s, CF_3), −72.98 (d, ^{1}J(P,F) = 706.1 Hz, PF_6) ppm.

^{31}P NMR (122 MHz, CD_3CN): δ = −144.61 (sept, ^{1}J(P,F) = 715.1 Hz, PF_6) ppm.

Elemental analysis (%; a sample re-crystallized from pyridine/pentane was analyzed): Anal. Calc. for [$CBiClF_9P(py)_{2.5}$] (656.16 g mol^{-1}): C 24.71, H 1.92, N 5.34; found C 24.62, H 2.22, N 5.46.

Preparation of [$Bi(CF_3)\mu_2$-$Cl(thf)_3]_2(SbF_6)_2$ (3-SbF_6)

A solution of $AgSbF_6$ (99 mg, 0.287 mmol) in DCM (1 mL) was added to a solution of $Bi(CF_3)Cl_2$ (100 mg, 0.287 mmol) in a mixture of both THF and DCM (0.1:1 mL). The reaction mixture was stirred for 2 h under exclusion of light. The precipitate was filtered off, and all volatiles were removed from the filtrate under reduced pressure to give a colorless powder that was dried *in vacuo*. Yield: 190 mg, 0.125 mmol, 87%.

Single crystals suitable for X-ray diffraction analysis were obtained by layering a solution of **3-SbF_6** in a mixture of both THF and DCM (0.1:1 mL) with *n*-pentane and storage at –30 °C for 16 hours.

^{1}H NMR (300 MHz, CD_2Cl_2): δ = 3.86 (m, 24H, O-CH_2), 1.94 (m, 24H, C-CH_2) ppm.

^{13}C NMR (300 MHz, DCM-d_2): δ = 175.79 (q, ^{1}J(F,C) = 370 Hz, CF_3) ppm.

^{19}F NMR (300 MHz, DCM-d_2): δ = −43.58 (s, CF_3), −132.01 (s, SbF_6) ppm.

Elemental analysis (%; a sample crystallized from reaction mixture by layering with *n*-pentane was analyzed): Anal. Calc. for [$C_{26}H_{42}Bi_2Cl_2F_{18}O_6Sb_2$] (1524.97 g mol^{-1}): C 20.48, H 2.78; found C 20.25, H 2.61.

Preparation of [$BiCF_3(py)_3$][SbF_6] (3-py-SbF_6)

Compound **3** (50 mg, 0.032 mmol) was dissolved in DCM (1 mL), and pyridine (16 mg, 0.196 mmol, 16 µL) was added. The colorless solution was layered with *n*-pentane (2 mL). After 1 day at –30 °C a colorless crystalline solid precipitated, was isolated by filtration, and dried *in*

vacuo. Yield: 49 mg, 0.062 mmol, 95%. Single crystals suitable for X-ray diffraction analysis were obtained by layering a pyridine solution of **3-py-SbF$_6$** with *n*-pentane and storage at –30 °C for 18 hours.

^{1}H NMR (300 MHz, CD_2Cl_2): δ = 7.61 (t, ^3J(H,H) = 7.75 Hz, 6H, H2/H6), 8.04 (t, ^3J(H,H) = 7.74 Hz, 3H, H4), 8.66 (d, ^3J(H,H) = 4.69 Hz, 6H, H3/H5) ppm.

^{13}C NMR (125 MHz, CD_2Cl_2): δ = 125.81 (s, C2/C6), 140.40 (s, C4), 147.26 (s, C3/C5), 161.12 (q, ^1J(F,C) = 372 Hz, *C*F$_3$) ppm.

^{19}F NMR (300 MHz, CD_2Cl_2): δ = −37.32 (br s, CF_3), −127.26 (s, SbF_6) ppm.

^{19}F NMR (300 MHz, CD_3CN): δ = −41.82 (br s, CF_3), δ = –106 to –140.98 (br, m, SbF_6) ppm.

Elemental analysis (%; a sample re-crystallized from pyridine/pentane was analyzed): Anal. Calc. for [$C_{16}H_{15}BiClF_9N_3Sb$] (786.49 g mol^{-1}): C 24.43, H 1.92, N 5.34; found C 23.99, H 2.01, N 5.39.

Preparation of [Bi(CF$_3$)(OTf)$_2$(thf)$_2$] (4)

AgOTf (59 mg, 0.230 mmol) was added to a solution of Bi(CF$_3$)Cl$_2$ (40 mg, 0.115 mmol) in both THF and DCM (0.1:1 mL). The precipitate was filtered off. All volatiles were removed from the filtrate under reduced pressure to give a colorless powder, which was dried *in vacuo*. Yield: 76.0 mg, 0.106 mmol, 92%. Single-crystals suitable for X-ray diffraction analysis were obtained by layering a solution of **4** in mixture of THF and DCM (0.1:1 mL) with *n*-pentane and storage at –30 °C for 16 h.

^{1}H NMR (300 MHz, CH_3CN-*d*$_3$): δ = 3.79 (m, 8H, O-C*H*$_2$), 1.87 (m, 8H, C-C*H*$_2$) ppm.

^{13}C NMR (75 MHz, CD_3CN): δ = 121.09 (q, ^1J(F,C) = 318 Hz, OTf), 172.30 (q, ^1J(F,C) = 330 Hz, *C*F$_3$) ppm.

^{19}F NMR (282 MHz, CD_3CN): δ = −40.36 (s, 3F, CF_3), −78.54 (s, 6F, OTf) ppm.

Elemental analysis (%; a sample crystallized from reaction mixture by layering with *n*-pentane was analyzed): Anal. calc. for [$C_{11}H_{16}BiF_9O_8S_2$] (720.33 g mol^{-1}): C 18.34, H 2.24, S 8.90; found C 18.29, H 2.27, S 8.91.

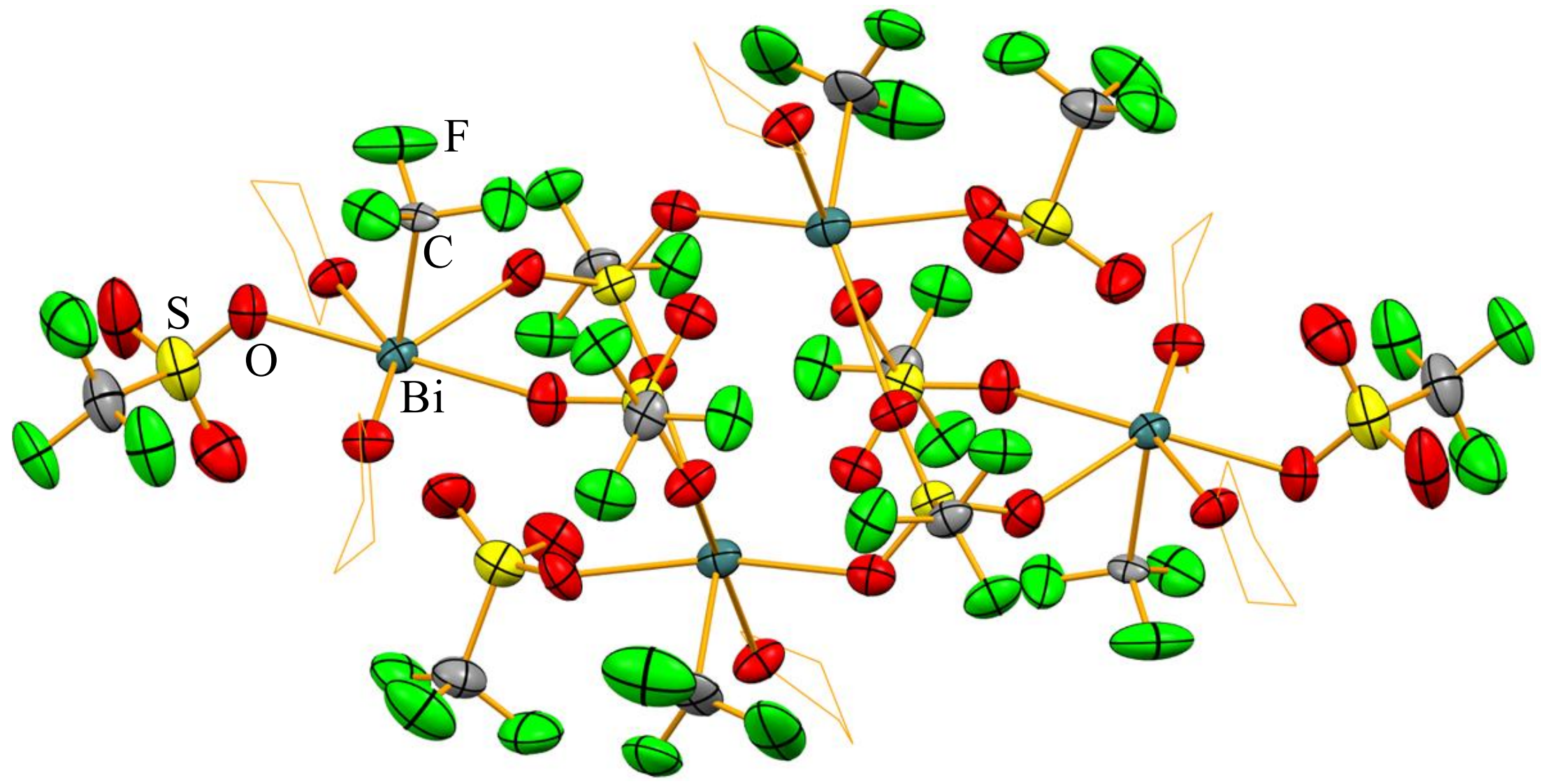

Figure S1. Molecular structure of **4** in the solid state. Displacement ellipsoids are shown at the 50% probability level. Carbon atoms of THF molecules are shown as wireframe for clarity. Hydrogen atoms are omitted for clarity.

Preparation of [Bi(CF_3)(OTf)$_2$(py)$_2$] (4-py)

A solution of **4** (20 mg, 0.028 mmol) in DCM (0.5 mL), and pyridine (4 mg, 0.055 mmol, 5 μL) was layered with pentane (0.8 mL). After 1 day at –30 °C colorless crystals of **4-py** had precipitated, were isolated by filtration and dried *in vacuo*. Yield: 20 mg, 0.027 mmol, 98%.

^{1}H NMR (300 MHz, CD_2Cl_2) δ 8.95 (d, ^3J(H,H)= 5.5 Hz, 4H, 2,6-pyridine), 8.17 (t, ^3J(H,H) = 7.8 Hz, 2H, 4-pyridine), 7.76 (t, ^3J(H,H) = 6.7 Hz, 4H, 3,5-pyridine).

^{13}C NMR (75 MHz, CD_2Cl_2): δ = 123.46 (q, ^1J(F,C) = 318 Hz, OTf), 126.19 (3,5-pyridine), 141.57 (4-pyridine), 146.89 (2,6-pyridine), 213.31 (q, ^1J(F,C) = 330 Hz, CF_3) ppm.

^{19}F NMR (282 MHz, CD_2Cl2): δ = −36.00 (s, 3F, CF_3), −78.84 (s, 6F, OTf) ppm.

Elemental analysis (%; a sample re-crystallized from pyridine/pentane was analyzed): Anal. calc. for [$C_3BiF_9O_6S_2$(py)$_2$] (734.32 g mol^{-1}): C 21.26, H 1.37, N 3.81, S 8.73; found C 21.18, H 1.39, N 3.76, S 8.70.

Preparation of [Bi(CF_3)(thf)$_3$(NTf_2)][NTf_2] (5)

A solution of $AgNTf_2$ (111 mg, 0.286 mmol) in DCM (1 mL) was added to a solution of Bi(CF_3)Cl_2 (50 mg, 0.143 mmol) in both THF and DCM (0.1:1 mL). The precipitate was filtered off. All volatiles were removed from the filtrate under reduced pressure to give a colorless powder, which was dried *in vacuo.* Yield: 136 mg, 0.129 mmol, 90%. Single-crystals suitable for X-ray diffraction analysis were obtained by layering a solution of **5** in both THF and DCM (0.1:1 mL) with *n*-pentane and storage at –30 °C for 16 h.

^{1}H NMR (300 MHz, CH_3CN-d_3): δ = 3.66 (m, 12H, O-CH_2), 1.81 (m, 12H, C-CH_2) ppm.

^{1}H NMR (300 MHz, CH_2Cl_2-d_2): δ = 3.73 (m, 12H, O-CH_2), 1.84 (m, 12H, C-CH_2) ppm.

^{13}C NMR (300 MHz, CH_3CN-d_3): δ = 174.73 (br s, CF_3), 120.10 (q, ^1J(F,C) = 320.8 Hz, NTf_2) ppm. The resonance of the CF_3 group appears as a broad singlet, which was ascribed to quadrupolar broadening due to the bismuth atom.

^{19}F NMR (300 MHz, DCM-d_2): δ = −39.80 (s, 3F, CF_3), −79.24 (s, 12F, NTf_2) ppm.

Elemental analysis (%; a sample re-crystallized from THF+DCM/PE and thoroughly dried *in vacuo* was analyzed): Anal. calc. for $C_{13}H_{16}BiF_{15}N_2O_{10}S_4$ [BiCF_3(thf)$_2$$NTf_2$]($NTf_2$) (982.47 g mol^{-1}): C 15.89, H 1.64, N 2.85, S 13.05; found C 15.61, H 1.68, N 2.87, S 13.03.

Preparation of [Bi(CF_3)(OTf)$_2$($SPMe_3$)$_2$] (6)

A solution of $SPMe_3$ (12 mg, 0.111 mmol) in DCM (1 mL) was added to a solution of [Bi(CF_3)(OTf)$_2$(thf)$_2$] (**4**) (40 mg, 0.056 mmol) in DCM (1 mL). The color of the reaction mixture changed into pale yellow immediately. The reaction mixture was stirred for 1 hour at room temperature, dried *in vacuo*, yielding a yellow crystalline product. Yield: 33 mg, 0.039 mmol, 70%.

Single-crystals suitable for X-ray diffraction analysis were obtained by layering an acetonitrile solution of **6** with dietheylether and storing it at –30 °C for 2 days.

^{1}H NMR (300 MHz, MeCN-d_3): δ = 2.09 (d, ^2J(P,H) = 13.8 Hz, 18 H, SP(CH_3)$_3$) ppm.

^{13}C NMR (75 MHz, MeCN-d_3): δ = 19.20 (d, ^1J(P,C) = 52.5 Hz, SP(CH_3)$_3$), 121 (q, ^1J(C,F) = 318 Hz, OTf), 188.70 (q, ^1J(C,F) = 326 Hz, CF$_3$) ppm.

^{19}F NMR (282 MHz, DCM-d_2): δ = −33.55 (s, CF_3), −79.13 (s, 6F, OTf) ppm.

^{31}P NMR (122 MHz, DCM-*d2*) δ = 44.69 (s, S*P*(CH$_3$)$_3$) ppm.

Elemental analysis (%; a sample re-crystallized from MeCN/Et$_2$O was analyzed): Anal. calc. for [$C_9H_{18}BiF_9O_6P_2S_4$] (792.39 g mol^{-1}): C 13.64, H 2.29, S 16.18; found: C 13.69 H 2.28, S 16.11.

Preparation of [Bi(CF$_3$)(OTf)$_2$(SePMe$_3$)$_2$] (7)

A solution of SePMe$_3$ (17 mg, 0.111 mmol) in DCM (1 mL) was added to a solution of [Bi(CF$_3$)(OTf)$_2$(thf)$_2$] (40 mg, 0.056 mmol) in DCM (1 mL). The color of the reaction mixture changed into pale yellow immediately. The reaction mixture was stirred for 1 hour at room temperature, dried *in vacuo*, yielding a yellow crystalline product. Yield: 44 mg, 0.048 mmol, 87%.

Single-crystals suitable for X-ray diffraction analysis were obtained by layering THF solution of **7** with pentane and storing it at –30 °C for 2 days.

^{1}H NMR (300 MHz, MeCN-*d3*): δ = 2.11 (d, ^{2}J(P,H) = 14.1 Hz, 18 H, SeP(C*H*$_3$)$_3$) ppm.

^{13}C NMR (126 MHz, MeCN-*d3*): δ = 18.85 (d, ^{1}J(P,C) = 46.8 Hz, SeP(CH$_3$)$_3$), 120.42 (q, ^{1}J(C,F) = 320.46 Hz, OTf), 175.53 (q, ^{1}J(C,F) = 324 Hz, *C*F$_3$) ppm.

^{19}F NMR (282 MHz, DCM-*d2*): δ = −30.67 (s, 3F, CF$_3$), −79.13 (s, 6F, OTf) ppm.

^{31}P NMR (101 MHz, DCM-*d2*): δ = 25.56 (s, ^{1}J(P,C) = 46.1 Hz, ^{1}J(P,Se) = 601.4 Hz, Se*P*(CH$_3$)$_3$) ppm.

Elemental analysis (%; a sample re-crystallized from THF/pentane was analyzed): Anal. calc. for [$C_9H_{18}BiF_9P_2S_2Se_2$(thf)] (958.32 g mol^{-1}): C 16.29, H 2.73, S 6.69; found C 16.11, H 2.73, S 6.68.

Preparation of [BiCF$_3$(SPMe$_3$)$_2$][SbF$_6$]$_2$ (8)

Solutions of SPMe$_3$ (31 mg, 0.286 mmol) in DCM (1 mL) and AgSbF$_6$ (99 mg, 0.288 mmol) in DCM (1 mL) were subsequently added to a solution of Bi(CF$_3$)Cl$_2$ (50 mg, 0.143 mmol) in both THF and DCM (0.1:1 mL). The color of the reaction mixture changed to yellow immediately, resulting in the formation of a precipitate. The reaction mixture was stirred for 1

hour at room temperature. The precipitate was filtered off and the filtrate was dried *in vacuo*, yielding a yellow crystalline product. Yield: 90 mg, 0.093 mmol, 65%.

Single crystals suitable for X-ray diffraction analysis were obtained by layering an acetonitrile solution of **8** with diethyl ether and storing it at –30 °C for 16 hours.

^{1}H NMR (300 MHz, MeCN-d_3): δ = 1.95 (d, ^{2}J(P,H) = 13.80 Hz, 18 H, SP(CH_3)$_3$) ppm.

^{13}C NMR (75 MHz, MeCN-d_3): δ = 19.71 (d, ^{1}J(P,H) = 53.9 Hz, SP(CH$_3$)$_3$), 191.54 (q, ^{1}J(F,C) = 387.5 Hz, CF$_3$) ppm.

^{19}F NMR (300 MHz, MeCN-d_3): δ = −37.70 (br s, CF_3), = –104.92 to –139.24 (br, m, SbF_6) ppm.

^{31}P NMR (122 MHz, MeCN-d_3) δ = 46.52 (s, SP(CH$_3$)$_3$) ppm.

Elemental analysis: Anal. Calc. for [$C_7H_{18}BiF_{15}P_2S_2Sb_2(Et_2O)$] (1039.89 g/mol): C 12.71, H 2.71, S 6.17; found: C 12.77, H 2.77, S 6.35.

Preparation of [$BiCF_3(SPMe_3)_4$][SbF_6]$_2$ (9)

Solutions of $SPMe_3$ (62 mg, 0.573 mmol) in DCM (1 mL) and $AgSbF_6$ (98.49 mg, 0.286 mmol) in DCM (1 mL) were subsequently added to a solution of Bi(CF_3)Cl_2 (50 mg, 0.143 mmol) in both THF and DCM (0.1:1 mL). The color of the reaction mixture changed into yellow immediately. The reaction mixture was stirred for 1 hour at room temperature. The precipitate was filtered off and the filtrate dried *in vacuo*, yielding a yellow crystalline product. Yield: 122 mg, 0.103 mmol, 72%. Single crystals suitable for X-ray diffraction analysis were obtained by layering an acetonitrile solution of compound **9** with diethyl ether and storing it at –30 °C for 16 hours.

^{1}H NMR (500 MHz, MeCN-d_3): δ = 1.97 (d, ^{2}J(P,H) = 13.70 Hz, 36 H, SP(CH_3)$_3$ ppm.

^{13}C NMR (125 MHz, MeCN-d_3): δ = 20.52 (d, ^{1}J(P,C) = 54.4 Hz, SP(CH$_3$)$_3$), 195.02 (q, ^{1}J(F,C) = 371 Hz, CF$_3$) ppm.

^{19}F NMR (300 MHz, MeCN-d_3): δ = –39.30 (s, CF_3), –103.83 to –141.06 (br, m, SbF_6) ppm.

^{31}P NMR (122 MHz, MeCN-d_3) δ = 42.89 (s, SP(CH$_3$)$_3$) ppm.
Elemental analysis: Anal. Calc. for [$C_{13}H_{36}BiF_{15}P_4S_4Sb_2$] (1182.04 g/mol): C 13.21, H 3.07, S 10.85; found: C 13.47, H 3.09, S 10.66.

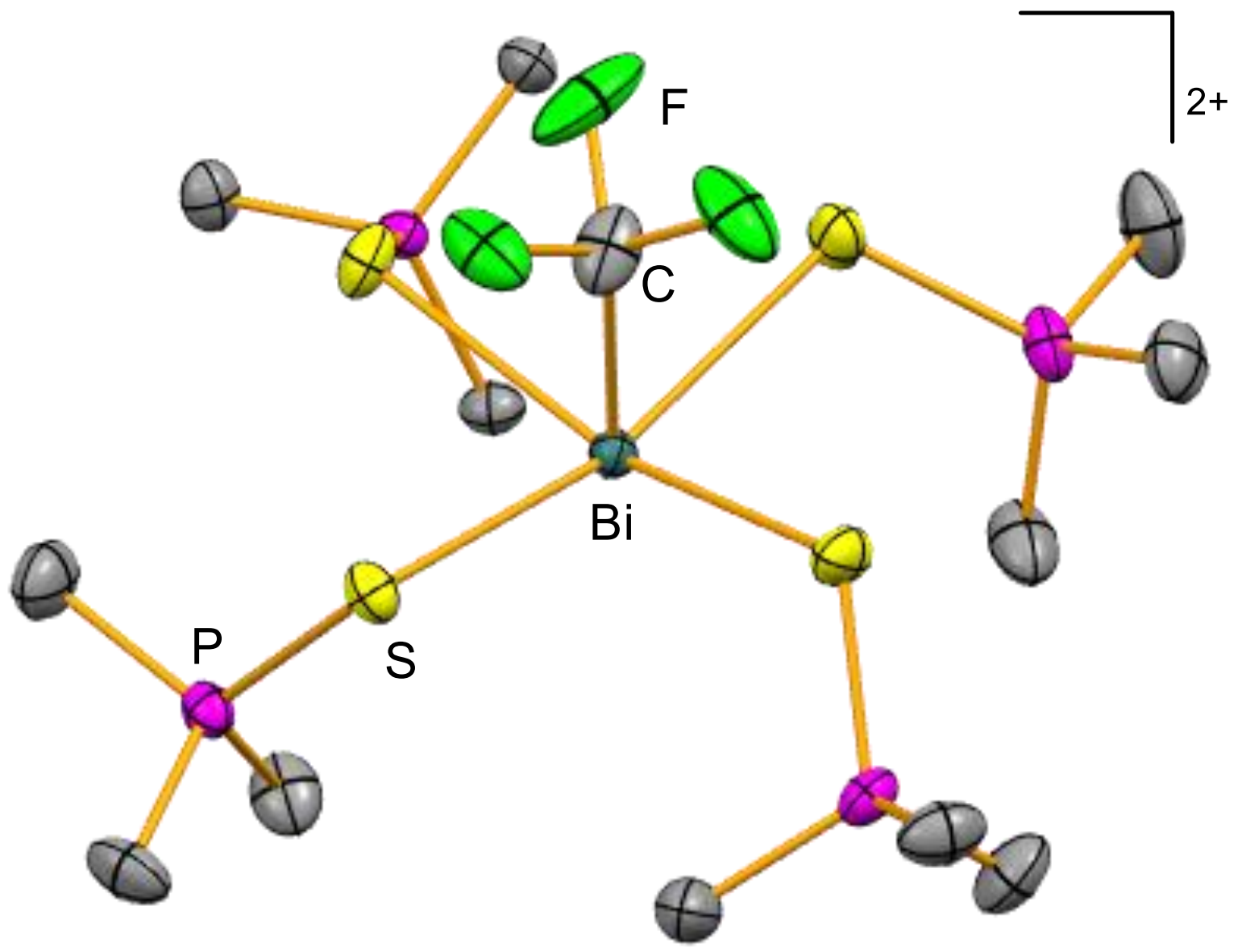

Figure S2. Molecular structure of compound **9**. Displacement ellipsoids are drawn at 50% probability level. Hydrogen atoms, SbF_6^- anions, split positions of disordered atoms, and lattice-bound solvent molecules are omitted for clarity.

Preparation of [Bi(thf)(CF_3)μ_2-F(bipy)]$_2$[SbF_6]$_2$ (10)

Solutions of 2,2'-bipyridine (9 mg, 0.058 mmol) in DCM (0.5 mL) and $AgSbF_6$ (40 mg, 0.116 mmol) in DCM (0.5 mL) were subsequently added to a solution of Bi(CF_3)Cl_2 (20 mg, 0.057 mmol) in both THF and DCM (0.1:1 mL) leading to a white precipitate. The precipitate was filtered off, and all volatiles were removed from the filtrate under reduced pressure to give a colorless powder of [Bi(solvent)(CF_3)μ_2-F(bipy)]$_2$[SbF_6]$_2$ that was dried *in vacuo*.

Single crystals of **10-py** suitable for X-ray diffraction analysis were obtained by layering a solution of **10** in pyridine (0.7 mL) with *n*-pentane and storage at –30 °C for 16 hours. The crystalline product was filtered off, washed with *n*-pentane (3 × 0.5 mL), and dried under reduced pressure to give a colorless powder of [Bi(py)(CF_3)μ_2-F(bipy)]$_2$[SbF_6]$_2$. Yield: 31.6 mg, 0.021 mmol, 74%.

Elemental analysis (%; a sample re-crystallized from pyridine/pentane was analyzed): Anal. Calc. for [$C_{26}H_{16}Bi_2F_{20}N_4Sb_2(py)_2$] (1536.05 g/mol): C 25.02, H 1.71, N 5.47; found: C 24.92, H 1.69, N 5.39.

^{1}H-NMR of **10-py** (300 MHz, MeCN-d_3): δ = 7.98 (ddd, ^3J(H,H) = 7.6 Hz, ^3J(H,H) = 5.1 Hz, $^4J_{HH}$ = 1.2 Hz, 4 H, bipy-*H*3), 8.46 (ddd, $^3J_{HH}$ = 7.8 Hz, ^3J(H,H) = 7.5 Hz, ^4J(H,H) = 1.6 Hz, 4 H, bipy-*H*2), 8.60 (d, ^3J(H,H) = 8.1 Hz, 4 H, bipy-*H*4), 9.32 (d, ^3J(H,H) = 4.8 Hz, 4 H, bipy-*H*1) ppm. The pyridine protons appear as broad signals at 7.63, 8.39 and 8.79 ppm after intense drying of the sample.

^{1}H-NMR of **10** (300 MHz, MeCN-d_3): δ = 7.74 (ddd, ^3J(H,H) = 7.7 Hz, ^3J(H,H) = 5.2 Hz, $^4J_{HH}$ = 1.2 Hz, 4 H, bipy-*H*3), 8.27 (ddd, $^3J_{HH}$ = 7.9 Hz, ^3J(H,H) = 7.7 Hz, ^4J(H,H) = 1.7 Hz, 4 H, bipy-*H*2), 8.43 (d, ^3J(H,H) = 8.1 Hz, 4 H, bipy-*H*4), 8.84 (d, ^3J(H,H) = 4.9 Hz, 4 H, bipy-*H*1) ppm.

^{13}C-NMR of **10** (75 MHz, MeCN-d$_3$): δ = 124.08 (s, bipy-C4), 127.63 (s, bipy-C3), 142.59 (s, bipy-C2), 148.99 (s, bipy-C1), 154.62 (s, bipy-C5), 206.39 (q, ^1J(F,C) = 381 Hz, *C*F$_3$) ppm.

^{19}F-NMR of **10** (283 MHz, MeCN-d_3): δ = –42.67 (s, CF$_3$), –80.23 (s, μ-*F*), –105.82 to –140.86 (br, m, Sb*F*$_6$) ppm.

Elemental analysis (%; a sample re-crystallized from THF/pentane was analyzed): Anal. Calc. for [$C_{26}H_{16}Bi_2F_{20}N_4Sb_2(thf)_2$] (1522.06 g/mol): C 23.67, H 2.12, N 3.68; found: C 23.40, H 2.06, N 3.62.

Elemental analysis: (%; a sample re-crystallized from MeCN/DEE was analyzed): Anal. Calc. for [$C_{26}H_{16}Bi_2F_{20}N_4Sb_2(CH_3CN)_2$] (1459.93 g/mol): C 21.39, H 1.52, N 5.76; found: C 21.19, H 1.66, N 5.78.

Preparation of [$Bi_2(CF_3)_2(\mu_2$-Cl)(μ_2-SePMe$_3$)(SePMe$_3$)$_4$][SbF$_6$]$_3$ (11)

Solutions of $AgSbF_6$ (30 mg, 0.087 mmol) in DCM (1 mL) and SePMe$_3$ (44.4 mg, 0.287 mmol) in DCM (1 mL) were subsequently added to a solution of Bi(CF$_3$)Cl$_2$ (20.0 mg, 0.057 mmol) in both THF and DCM (0.1:1 mL). The color of the reaction mixture changed into yellow-orange immediately. The reaction mixture was stirred for 1 hour at room temperature. The precipitate was filtered off and the filtrate dried *in vacuo*, yielding a yellow crystalline product. Yield: 34.0 mg, 0.0164 mmol, 57%. Single crystals suitable for X-ray diffraction analysis were obtained by layering a DCM solution of compound **11** with pentane and storing it at –30 °C for 16 hours.

^{1}H NMR (300 MHz, MeCN-d_3): δ = 2.21 (d, ^2J(P,H) = 14.0 Hz, 45 H, SeP(C*H*$_3$)$_3$ ppm.

^{13}C NMR (125 MHz, MeCN-d_3): δ = 19.75 (d, ^{1}J(P,C) = 48.1 Hz, SeP(CH$_3$)$_3$), 183.91 (q, ^{1}J(F,C) = 382 Hz, CF$_3$) ppm.

^{19}F NMR (300 MHz, MeCN-d_3): δ = −38.60 (s, CF$_3$), –105.82 to –141.14 (br, m, SbF$_6$) ppm.

^{19}F NMR (300 MHz, CH$_2$Cl$_2$-d_2): δ = −37.78 (s, CF$_3$) ppm. A signal for the [SbF$_6$]$^{-}$ anion in a ^{19}F NMR experiment could not be detected due to signal broadening.

^{31}P NMR (122 MHz, MeCN-d_3) δ = 26.81 (s, ^{1}J(P,C) = 46.6 Hz, ^{1}J(P,Se) = 508.3 Hz, SeP(CH$_3$)$_3$) ppm.

Elemental analysis: Anal. Calc. for [$C_{21}H_{53}Bi_2ClF_{24}OP_5Se_5Sb_3$] (2146.03 g/mol): C 11.75, H 2.49; found: C 11.63, H 2.51.

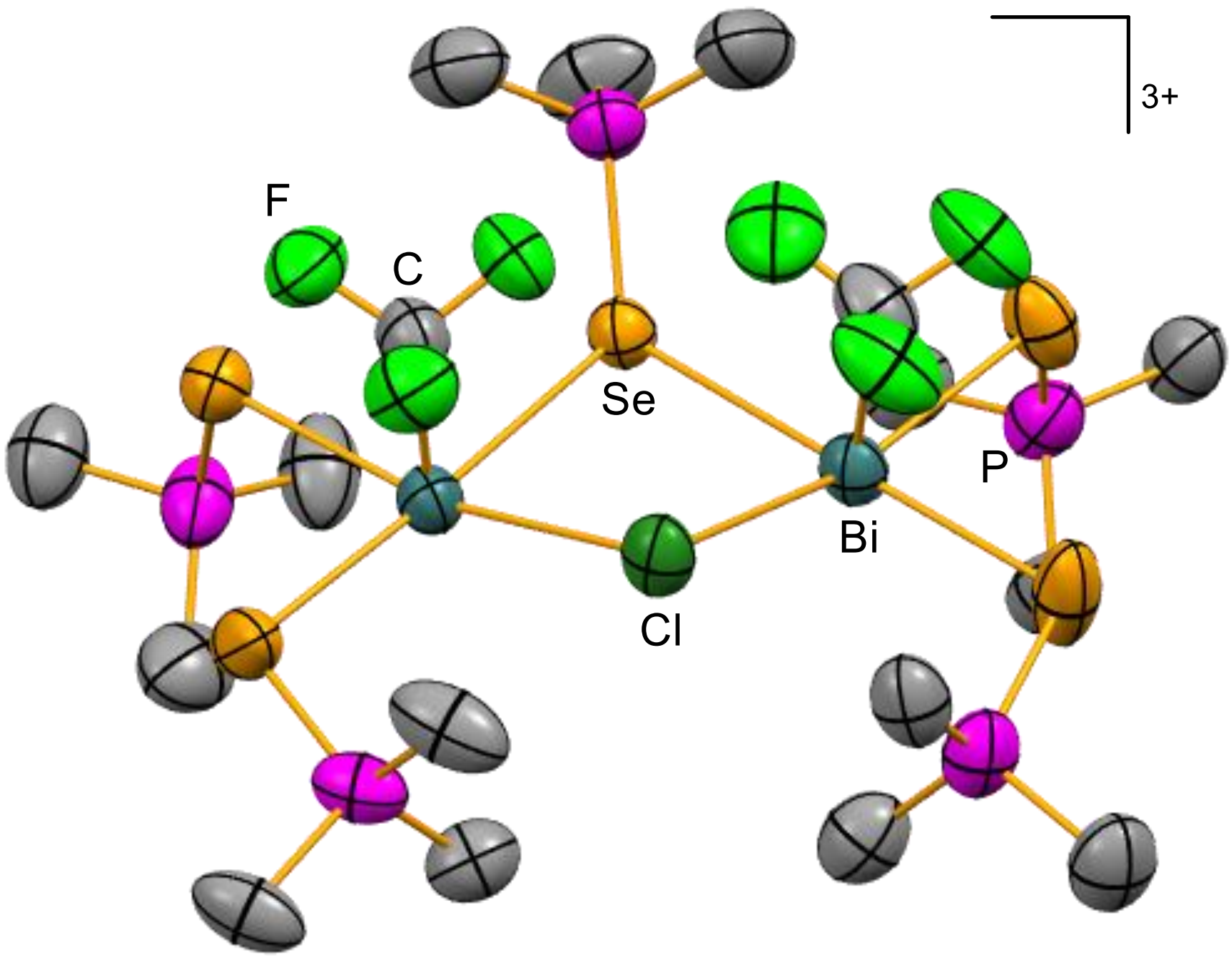

Figure S3. Molecular structure of compound **11**. Displacement ellipsoids are drawn at 50% probability level. Hydrogen atoms, SbF$_6^{-}$ anions, split positions of disordered atoms, and lattice-bound solvent molecules are omitted for clarity.

Preparation of [Bi(CF$_3$)μ$_2$-Cl(SePMe$_3$)$_2$]$_2$[SbF$_6$]$_2$ (12)

[Bi(CF$_3$)μ$_2$-Cl(thf)$_3$]$_2$[SbF$_6$]$_2$ (20 mg, 0.013 mmol, 1.0 eq.) was dissolved in acetonitrile (0.4 mL) and a solution of SePMe$_3$ (8 mg, 0.052 mmol, 4.0 eq.) in acetonitrile (0.4 mL) was added. The color of the reaction mixture changed to yellow immediately. The reaction mixture

was stirred for 1 hour at room temperature. The solution was layered with diethyl ether (5 mL). Storing the solution at –30 °C leads to the formation of a yellow crystalline product after 2 days. Filtration and drying *in vacuo* yields compound **12** (15 mg, 0.0087 mmol, 67%).

^{1}H NMR (300 MHz, DCM-d_2): δ = 2.23 (d, ^{2}J(P,H) = 13.60 Hz, 36 H, SeP(CH_3)$_3$ ppm.
^{13}C NMR (75 MHz, DCM-d_2): δ = 19.89 (d, ^{1}J(P,C) = 48.4 Hz, SeP(CH$_3$)$_3$), 172.19 (q, ^{1}J(F,C) = 375 Hz, CF$_3$) ppm.

^{19}F NMR (282 MHz, MeCN-d_3): δ = –40.94 (s, CF$_3$), –109.84 to –140.07 (br, m, SbF$_6$) ppm.

^{31}P NMR (122 MHz, DCM-d_2) δ = 19.08 (s, ^{1}J(P,C) = 48.2 Hz, ^{1}J(P,Se) = 498.2 Hz, SeP(CH$_3$)$_3$) ppm.
Elemental analysis: Anal. Calc. for [$C_{14}H_{36}BiF_{12}P_4Se_4Sb_2(thf)_{0.5}$] (1182.04 g/mol): C 11.25, H 2.35; found: C 11.18, H 2.36.

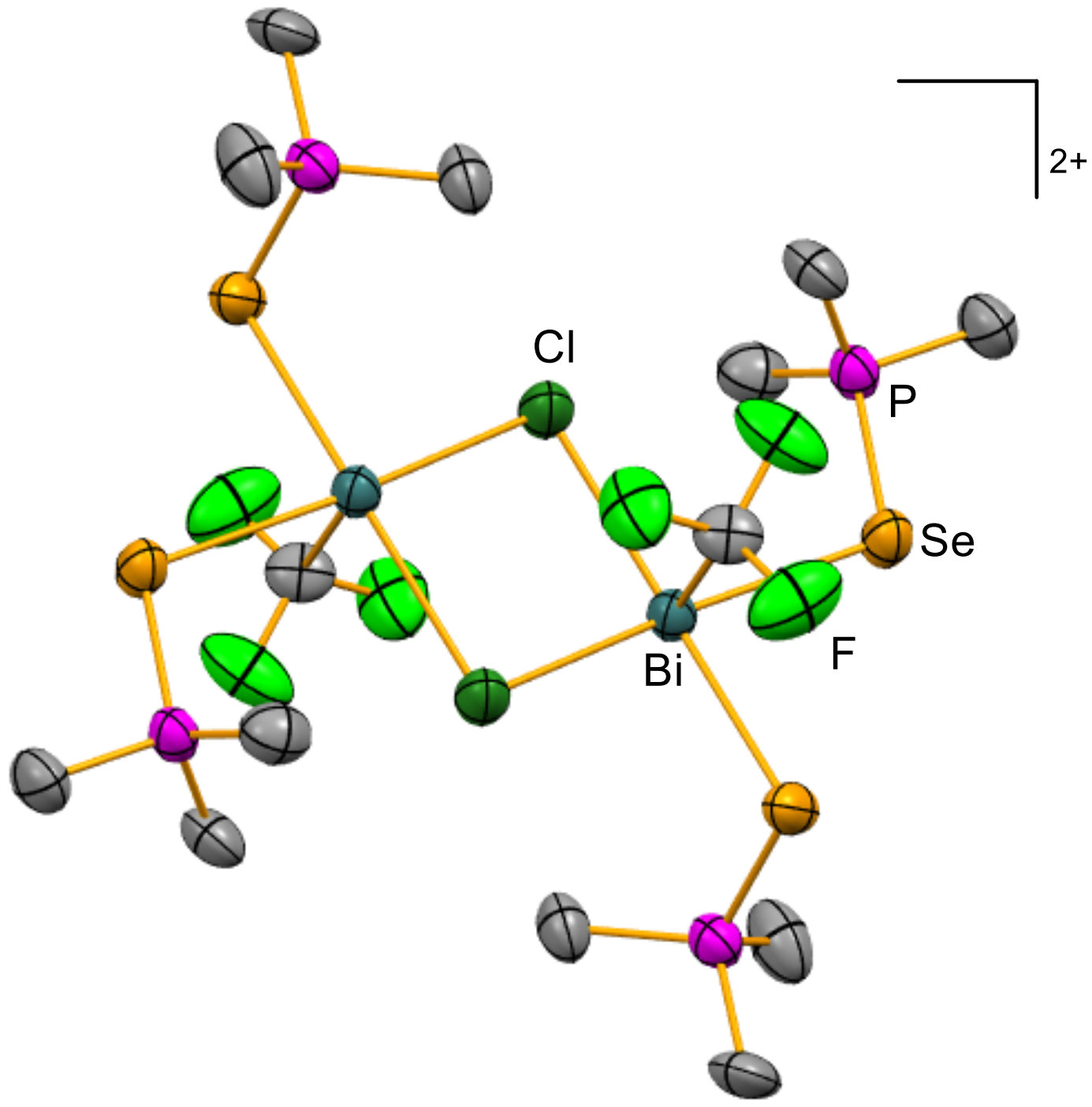

Figure S4. Molecular structure of compound **12**. Displacement ellipsoids are drawn at 50% probability level. Hydrogen atoms, SbF_6^- anions, split positions of disordered atoms, and lattice-bound solvent molecules are omitted for clarity.

Preparation of $[Bi(CF_3)(SePMe_3)_4][NTf_2]_2$ (13)

A solution of $SePMe_3$ (24 mg, 0.151 mmol) in DCM (1 mL) was added to a solution of $[Bi(CF_3)(thf)_3(NTf_2)][NTf_2]$ (40 mg, 0.038 mmol) in DCM (1 mL). The color of the reaction mixture changed to pale yellow immediately. The reaction mixture was stirred for 1 hour at room temperature, dried *in vacuo*, yielding a yellow crystalline product. Yield: 40 mg, 0.027 mmol, 72%.

Single-crystals suitable for X-ray diffraction analysis were obtained by layering DCM solution of **13** with pentane and storing it at –30 °C for 2 days.

^{1}H NMR (300 MHz, DCM-d_2): δ = 2.28 (d, ^2J(P,H) = 13.7 Hz, 36 H, SeP(CH_3)$_3$) ppm.

^{13}C NMR (126 MHz, DCM-d_2): δ = 20.88 (d, ^1J(P,C) = 47.9 Hz, SeP(CH_3)$_3$), 120.52 (q, ^1J(C,F) = 315.73 Hz, NTf_2), 172.74 (b, CF_3) ppm.

^{13}C NMR (75 MHz, CH_3CN-d_3): δ = 20.72 (d, ^1J(P,C) = 47.9 Hz, SeP(CH_3)$_3$), 123.47 (q, ^1J(C,F) = 317 Hz, NTf_2), 172.72 (b, CF_3) ppm.

^{19}F NMR (282 MHz, DCM-d_2): δ = −30.62 (s, 3F, CF_3), −79.19 (s, 12F, NTf_2) ppm.

^{31}P NMR (101 MHz, DCM-d_2): δ = 21.94 (s, ^1J(P,C) = 47.6 Hz, ^1J(P,Se) = 589.3 Hz, SeP(CH_3)$_3$) ppm.

Elemental analysis (%; a sample re-crystallized from DCM/pentane was analyzed): Anal. calc. for $[C_{17}H_{36}BiF_{15}P_4S_4Se_4O_8N_2]$ (1458.46 g mol^{-1}): C 14.00, H 2.49, N 1.92, S 8.79; found C 14.07, H 1.95, N 1.78, S 8.68.

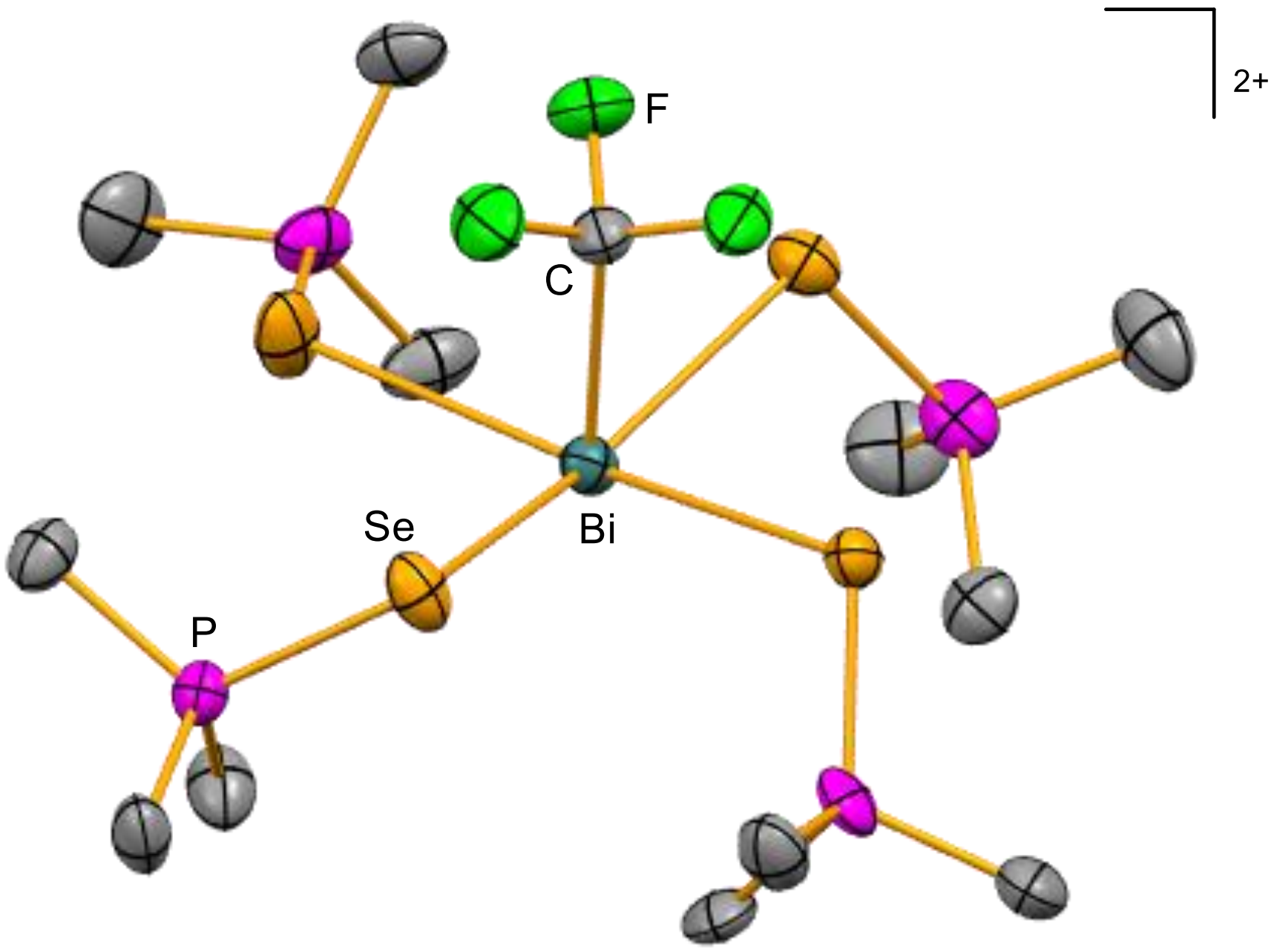

Figure S5. Molecular structure of compound **13**. Displacement ellipsoids are drawn at 50% probability level. Hydrogen atoms, NTf_2^- anions, split positions of disordered atoms, and lattice-bound solvent molecules are omitted for clarity.

The P–Se bond lengths in **13** average to 2.161(2) Å (omitting values from one disordered $SePMe_3$ group). These values are larger within a two-sigma-threshold than the corresponding values found in the previously reported compound $[BiMe(SePMe_3)_4][SbF_6]_2$, (Se–P(avg), 2.1530(11) Å),[5] which further supports the stronger substrate activation in the case of compound **13**.

2. Discussion of the crystal structures of compounds $Bi(CF_3)Ph_2$ and $[Bi(CF_3)Cl_2(py)_2]$

Compound $Bi(CF_3)Ph_2$ (**1**) crystallizes in the monoclinic space group $P2_1/c$ (Z = 4). The bismuth atom adopts a slightly distorted trigonal pyramidal coordination geometry, coordinated by two phenyl ligands and one trifluoromethyl group, with an angular sum of 278.69° around Bi.

The average Bi–C bond length is 2.271 Å, consistent with reported values for other arylbismuthine derivatives.[6] The distance of bond between bismuth–carbon of the trifluoromethyl group (2.327(2) Å)) is notably longer than the corresponding $Bi–C_{phenyl}$ (2.249(2)–2.236(2) Å), which may be attributed to the electron-withdrawing nature of the CF_3 group. Interatomic distances of 3.669 Å between the bismuth atom and a carbon atom of a neighboring molecule suggest weak intermoleculear $Bi\cdots C^{Aryl}$ contacts in the solid state, involving $\pi(C{=}C)\rightarrow\sigma^*(Bi–CF_3)$ interactions, leading to a dimeric arrangement.

Compound $[Bi(CF_3)Cl_2(py)_2]$ (**2**) crystallizes in the monoclinic space group $P2_1/n$ (Z = 4). The bismuth atom is pentacoordinate, adopting a square-pyramidal geometry in which the trifluoromethyl group occupies the apical position with two *trans* chloride ligands and two *trans* pyridine ligands residing in the basal plane. The N–Bi–N angle (162.88°) is smaller than the Cl–Bi–Cl angle (169.03°), likely due to steric reasons. In addition, each of the chloride ligands shows weak bonding interactions to the bismuth atoms of neighboring formula units ($Bi\cdots Cl$ 3.505(7)–3.667(6) Å) through $n(Cl)\rightarrow\sigma^*(Bi–CF_3)$ interactions. This results in a one-dimensional coordination polymer along the crystallographic a-axis with a $[Bi(\mu_2\text{-}Cl)_2Bi]_\infty$ strand.

3. Determination of acceptor numbers by the (modified) Gutmann-Beckett method

For an experimental determination of the Lewis acidity of compounds **3-SbF$_6$**, **4**, and **5**, the traditional Gutmann-Beckett method (using $OPEt_3$),[7] and the modified version (using $SPMe_3$ and $SePMe_3$) were applied.[8] The acceptor numbers were calculated based on the ^{31}P-NMR chemical shift of the respective reaction mixtures of **3-SbF$_6$** or **4** or **5** with the Lewis base. The relevant equations are:

$$OPEt_3\text{: } AN = 2.21 \cdot (\delta_P - 41.0) \qquad \text{(eq. 1)}$$

$$SPMe_3\text{: } AN = 6.41 \cdot (\delta_P - 29.2) \qquad \text{(eq. 2)}$$

$$SePEt_3\text{: } AN = 5.71 \cdot (\delta_P - 7.8) \qquad \text{(eq. 3)}$$

NMR spectroscopic experiments were conducted *in situ* first, in a 1:1 stoichiometry, and secondly with several measurements increasing the amount of the Lewis base gradually from 1.0 to 4.0 equivalents. All measurements were done in CD_2Cl_2. The results are summarized in Table 1 in the main part.

4. Synthesis and characterization of $Bi(CF_3)^{2+}$ adducts with EPR_3 (E= S, Se; R=Me)

Our objective was to investigate the coordination chemistry of bismuth dications by determining the precise quantity of each (modified) Gutmann-Beckett donor required to generate a stable product.

In case of the OTf^- anion, two equivalents of the donors were sufficient to isolate $[Bi(CF_3)(OTf)_2(SPMe_3)_2]$ (**6**) and $[Bi(CF_3)(OTf)_2(SePMe_3)_2]$ (**7**). Single-crystal X-Ray diffraction analysis revealed similar coordination geometries for **6** (triclinic space group $P\overline{1}$, Z = 4) and **7** (triclinic space group $P\overline{1}$, Z = 2). The equatorial plane is occupied by four atoms (two from the Lewis base and two from the triflate anions in a *cis* configuration), while the apical position is occupied by the trifluoromethyl group. The position *trans* to the trifluoromethyl group is not occupied because the bismuth atom is positioned on an inversion center, leading to a square pyramidal coordination geometry.

The range of Bi–O_{OTf} distances in compounds **6** and **7** [2.467(6)–2.840(2) Å] is significantly longer than the sum of the covalent radii [Bi–O = 2.25 Å] and significantly shorter than the sum of the van der Waals radii [Bi–O, 3.59 Å].[8,9] The observed Bi–O_{OTf} distances are generally consistent with those reported for representative bismuth triflate compounds [2.386(1)–2.879(2) Å][10] and are generally longer than the Bi–O distances in the covalent alkoxide $Bi(OCPh_3)_3$ [2.070(4)–2.081(4) Å].[9,11]

The range of Bi–S/Se distances in compounds **6** [2.661(3)–2.680(2) Å] and **7** [2.739(1)–2.805(5) Å] is significantly shorter than the sum of the van der Waals radii (S, 1.80 Å; Se, 1.90 Å; Bi, 2.07 Å).[12] They are also much shorter than the corresponding bonds in the literature-known adducts $[Bi(SC_6F_5)_3(SPPh_3)]$ (Bi-$SPPh_3$ 3.01 Å) and $[BiX_3(SeP(4\text{-}F\text{-}C_6H_4)_3)]$ (X=Cl, Br: Bi-Se, 3.35–3.37 Å).[13] In fact, they are in the range of values reported for regular covalent Bi−S/Se bonds in compounds of type $Bi(aryl)_2(EPh)$ (E=S, 2.54–2.63 Å; E=Se, 2.70–2.73 Å).[8] They are in agreement with the value reported for $[Bi(diaryl)(EPMe_3)][SbF_6]$ (E=S, 2.61 Å; E=Se, 2.72 Å).[8] The P−S/Se bonds in **6** (2.013(4)–2.034(3) Å) and **7** (2.159(2)–2.188(8) Å) are

significantly elongated compared to those in $SPMe_3$ (1.97 Å) and $SePMe_3$ (2.12 Å),[8,14] but still similar to the values reported for [Bi(diaryl)($EPMe_3$)][SbF_6] (E=S, 2.03 Å; E=Se, 2.19).[8]

In the case of the SbF_6^- anion, compounds [$BiCF_3(SPMe_3)_2$][SbF_6]$_2$ (**8**) and [$BiCF_3(SPMe_3)_4$][SbF_6]$_2$ (**9**) were isolated using two and four equivalents of the $SPMe_3$ donor, respectively. They were prepared by *in situ* reaction of $Bi(CF_3)Cl_2$ (**2**) with 2 or 4 equiv. of $SPMe_3$ and 2 equiv. of $AgSbF_6$. Single-crystal X-Ray diffraction shows distinct coordination geometries for **8** (triclinic space group $P\overline{1}$, Z = 2) and **9** (monoclinic space group $C2/c$, Z = 8). For compound **8**, an unusual coordination number of three is observed, with two $SPMe_3$ donors in the equatorial plane and the trifluoromethyl group in the apical position. The two SbF_6^- anions exhibit weak interactions with the bismuth atom, as judged by distance criteria.

In contrast, compound **9** has four $SPMe_3$ donors, forming a square pyramidal coordination geometry around the bismuth atom. An additional weak interaction with the SbF_6^- anion is observed in the solid state in *trans* position of the CF_3 groups (Bi···F 3.095(3) Å). The Bi–S distances in compound **9** [2.758(1)–2.838(1) Å] is slightly longer than the corresponding bonds in compound **8** [2.588(8)–2.622(1) Å] and compound **6** [2.661(3)–2.680(2) Å]. The P−S bonds in **8** (2.047(1)–2.051(2) Å) and **9** (2.011(2)–2.012(1) Å) follow the same bonding trend observed in compound **6**.

Attempt to isolate similar dicationic species using the $SePMe_3$ donor resulted in the formation of the tricationic species [$Bi_2(CF_3)_2(SePMe_3)_5$(thf)Cl][SbF_6]$_3$ (**11**). It crystallizes in the monoclinic space group $P2_1/n$ (Z = 4). Each bismuth atom in this molecular complex is pentacoordinate, adopting a square-pyramidal coordination geometry due to interactions of each bismuth atom with one trifluoromethyl group, two terminally bound $SePMe_3$ ligands, and two μ_2-bridging ligands (one chlorido and one $SePMe_3$ donor). Weak directional bonding interactions with the SbF_6^- counteranions (Bi···F 2.804(2)–2.838(2) Å). The Bi–Se/P–Se distances in compounds **11** follow the same bonding trend observed in compound **7**.

On the other hand, the reaction of compound **3-SbF₆** with 4 equivalents of $SePMe_3$ donors results in the formation of [$Bi(CF_3)\mu_2$-$Cl(SePMe_3)_2$]$_2$[SbF_6]$_2$ (**12**). It crystallizes in the triclinic space group ($P\overline{1}$, Z = 1). The bismuth atoms in **12** are penta-coordinate due to interactions of each bismuth atom with one trifluoromethyl group, two terminally bound $SePMe_3$ ligands and two μ_2-bridging chlorido ligands, creating a square pyramidal coordination geometry with a cis-configuration around bismuth atoms with the CF_3 group in the apical position. In addition, there are weak directional bonding interactions with the SbF_6^- counteranions (Bi···F 2.979(3)–

3.005(4) Å). The Bi–Se/P–Se distances in compound **12** have the same bonding trend as outlined above for compound **7**.

In case of NTf_2^- anion, we were able to separate $[Bi(CF_3)(SePMe_3)_4][NTf_2]_2$ (**13**) using four equivalents of the $SePMe_3$ donor. It crystallizes in the monoclinic space group $P2_1/c$ (Z = 4). It shows a square pyramidal coordination geometry, with the CF_3 group in the apical position. Additional weak bonding interactions of the central atom with one NTf_2^- ligand is observed in the solid state in *trans*-position of the CF_3 group (Bi–O 3.187(4)). The Bi–Se distances in compound **13** range from 2.902(1)–2.987(1) Å and are thus significantly shorter than the sum of the van der Waals radii, but slightly longer than those reported in compound **7**. They are slightly shorter than the corresponding bonds reported for $[Bi(CH_3)_2(SePMe_3)_4](SbF_6)_2$ (Bi–Se 2.9344(4)-2.9476(4) Å).[5] The P−Se bonds in **13** (2.153(3)–2.166(2) Å) follow the same trend as those in compound **7**.

5. Additional adducts

Preparation of $[Bi(CF_3)(thf)(NTf_2)_2(bipy)]$

$[Bi(CF_3)(thf)_3(NTf_2)](NTf_2)$ (40 mg, 0.038 mmol) was dissolved in DCM (0.5 mL) and 2,2′-bipyridine (6 mg, 0.038 mmol) in DCM (0.5 mL) was added. The product was crushed out by addition of pentane (5 mL). The precipitate was filtered off, and all volatiles were removed from the filtrate under reduced pressure to give a colorless powder that was dried *in vacuo*. Yield: 30 mg, 0.028 mmol, 74%. Single-crystals suitable for X-ray diffraction analysis were obtained by layering a solution of $[Bi(CF_3)(thf)(NTf_2)_2(bipy)]$ in DCM (0.7 mL) with pentane and storage at –30 °C for 16 hours.

^{1}H-NMR (300 MHz, MeCN-d_3): δ = 3.64 (m, 2H, O-CH_2), 1.79 (m, 8H, C-CH_2), 8.10 (ddd, ^3J(H,H) = 7.69 Hz, ^3J(H,H) = 5.1 Hz, $^4J_{HH}$ = 1.19 Hz, 2 H, bipy-*H*4), 8.59 (ddd, $^3J_{HH}$ = 7.9 Hz, ^3J(H,H) = 7.7 Hz, ^4J(H,H) = 1.6 Hz, 2 H, bipy-*H*3), 8.72 (d, ^3J(H,H) = 8.1 Hz, 2 H, bipy-*H*5), 9.29 (broad, 2 H, bipy-*H*2) ppm.THF molecule has two multiples at 1.80 and 3.65 ppm.

^{13}C-NMR (125 MHz, MeCN-d_3): δ = 119.20 (q, ^1J(F,C) = 320.4 Hz, NTf_2), 127.57 (s, bipy-C5), 130.43 (s, bipy-C4), 144.86 (s, bipy-C3), 151.79 (s, bipy-C2), 154.67 (s, bipy-C6), 173.03 (broad, *C*F_3) ppm.

^{19}F NMR (282 MHz, MeCN-d_3): δ = −38.29 (s, 3F, CF_3), –79.70 (s, 12F, NTf_2) ppm.

Elemental analysis: **Anal. Calc. for [$C_{19}H_{16}BiF_{15}N_4O_9S_4$]** (1066.55 g/mol): C 21.40, H 1.51, N 5.25; S 12.02 found: C 21.29, H 1.63, N 5.30, S 12.01.

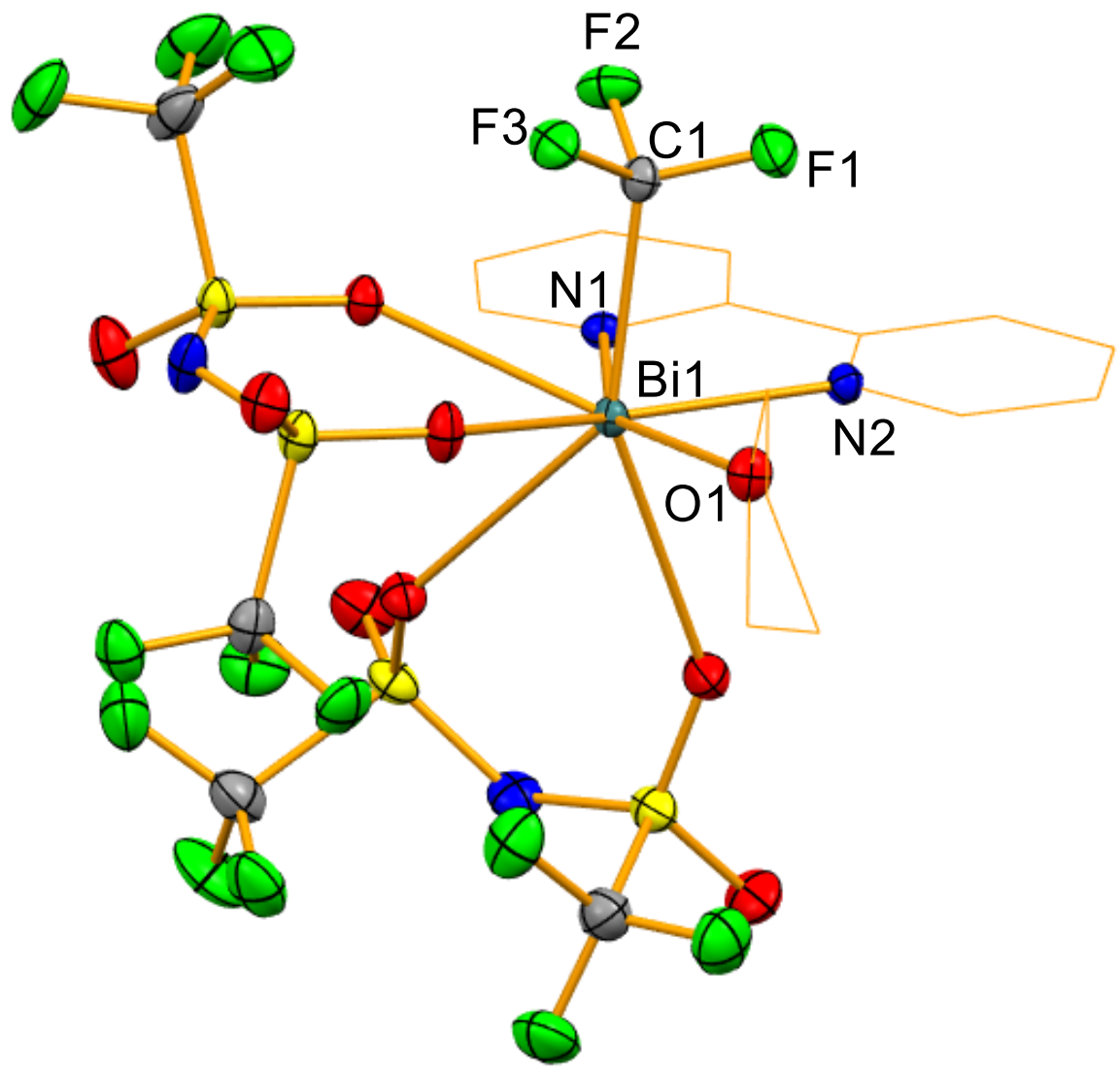

Figure S6. Molecular structure of **[Bi(CF_3)(thf)(NTf_2)$_2$(bipy)]** in the solid state. Displacement ellipsoids are shown at the 50% probability level. Hydrogen atoms are omitted for clarity. Carbon atoms of THF molecules and 2,2′-bipyridine are shown as wireframe for clarity. Hydrogen atoms are omitted for clarity.

Preparation of [Bi(CF_3)(thf)(NTf_2)$_2$(phen)]

[Bi(CF_3)(thf)$_3$(NTf_2)](NTf_2) (40 mg, 0.038 mmol) was dissolved in DCM (0.5 mL) and 1,10-phenanthroline (7 mg, 0.038 mmol) in DCM (0.5 mL) was added. The product was crushed out by addition of pentane (5 mL). The precipitate was filtered off, and all volatiles were removed from the filtrate under reduced pressure to give a colorless powder that was dried *in vacuo*. Yield: 36 mg, 0.033 mmol, 87%. Single crystals suitable for X-ray diffraction analysis were obtained by layering a solution of [Bi(CF_3)(thf)(NTf_2)$_2$(phen)] in DCM (0.7 mL) with pentane and storage at –30 °C for 20 hours.

^{1}H-NMR (300 MHz, MeCN-*d_3*): δ = 8.32 (broad, 4 H, phen-*H*12, *H*18), 9.07 (broad, 2 H, phen-*H*11), 9.58 (broad, 2 H, phen-*H*19) ppm. THF molecule has two multiples at 1.80 and 3.64 ppm. The number of protons corresponds to THF molecules is lower than expected because of the partial replacement of THF molecule by CD_3CN molecule.

^{13}**C-NMR** (75 MHz, MeCN-d_3): δ = 118.62 (q, ^{1}J(F,C) = 318.2 Hz, NTf$_2$),128.14 (s, phen-C3), 129.67 (s, phen-C6), 133.41 (s, phen-C5), 139.24 (s, phen-C4), 143.64 (s, phen-C13), 153.08 (s, phen-C2), 207.84 (broad, *C*F$_3$) ppm.

^{19}**F NMR** (282 MHz, MeCN-d_3): δ = −38.67 (s, 3F, CF$_3$), −79.83 (s, 12F, NTf$_2$) ppm.

Elemental analysis: **Anal. Calc. for [$C_{21}H_{16}BiF_{15}N_4O_9S_4$]** (1090.57 g/mol): C 23.13, H 1.48, N 5.14; S 11.76 found: C 23.25, H 1.55, N 5.16, S 11.88.

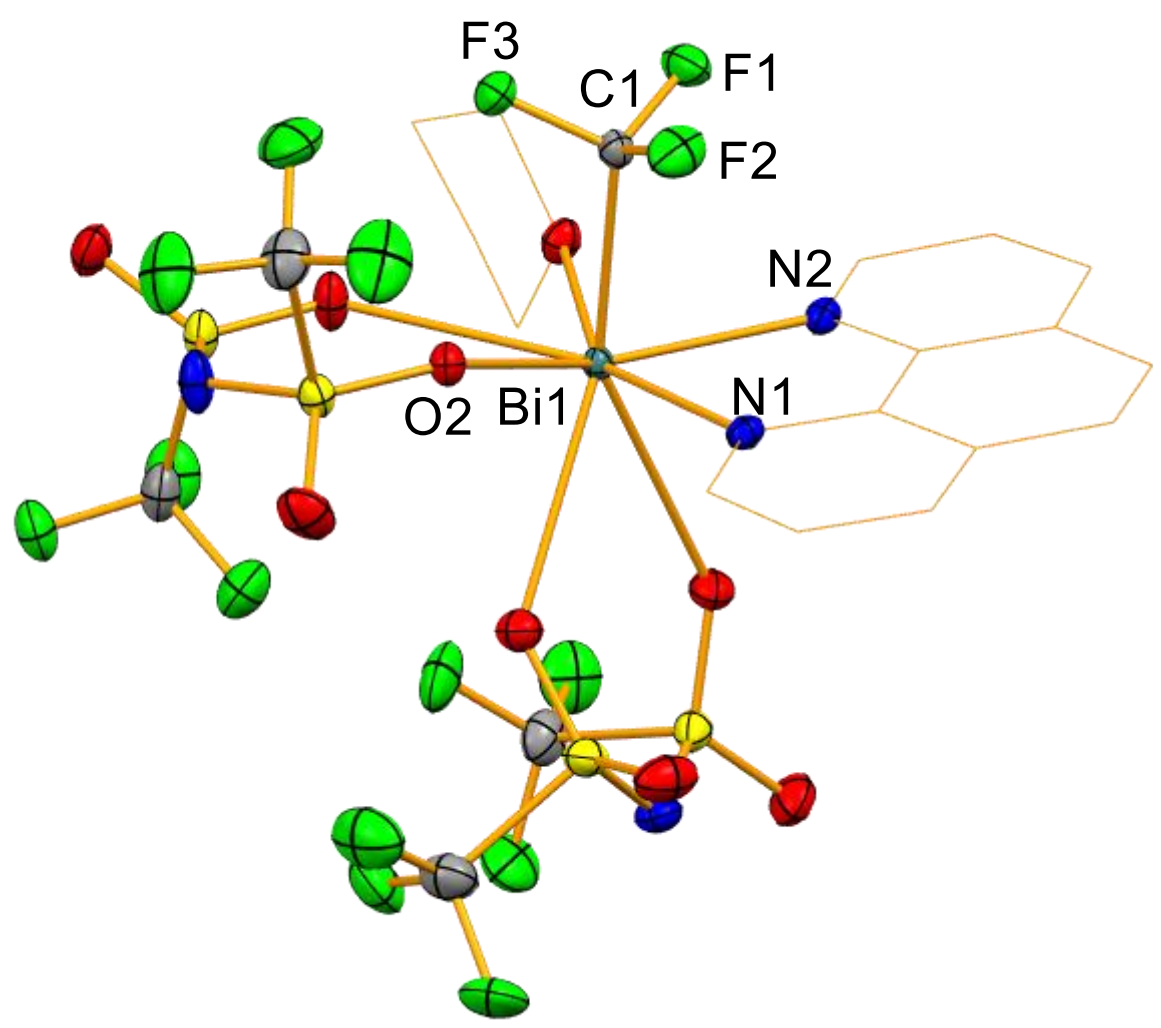

Figure S7. Molecular structure of **[Bi(CF$_3$)(thf)(NTf$_2$)$_2$(phen)]** in the solid state. Displacement ellipsoids are shown at the 50% probability level. Hydrogen atoms are omitted for clarity. Carbon atoms of THF molecules and 1,10-phenanthroline are shown as wireframe for clarity. Hydrogen atoms are omitted for clarity.

6. NMR Spectra

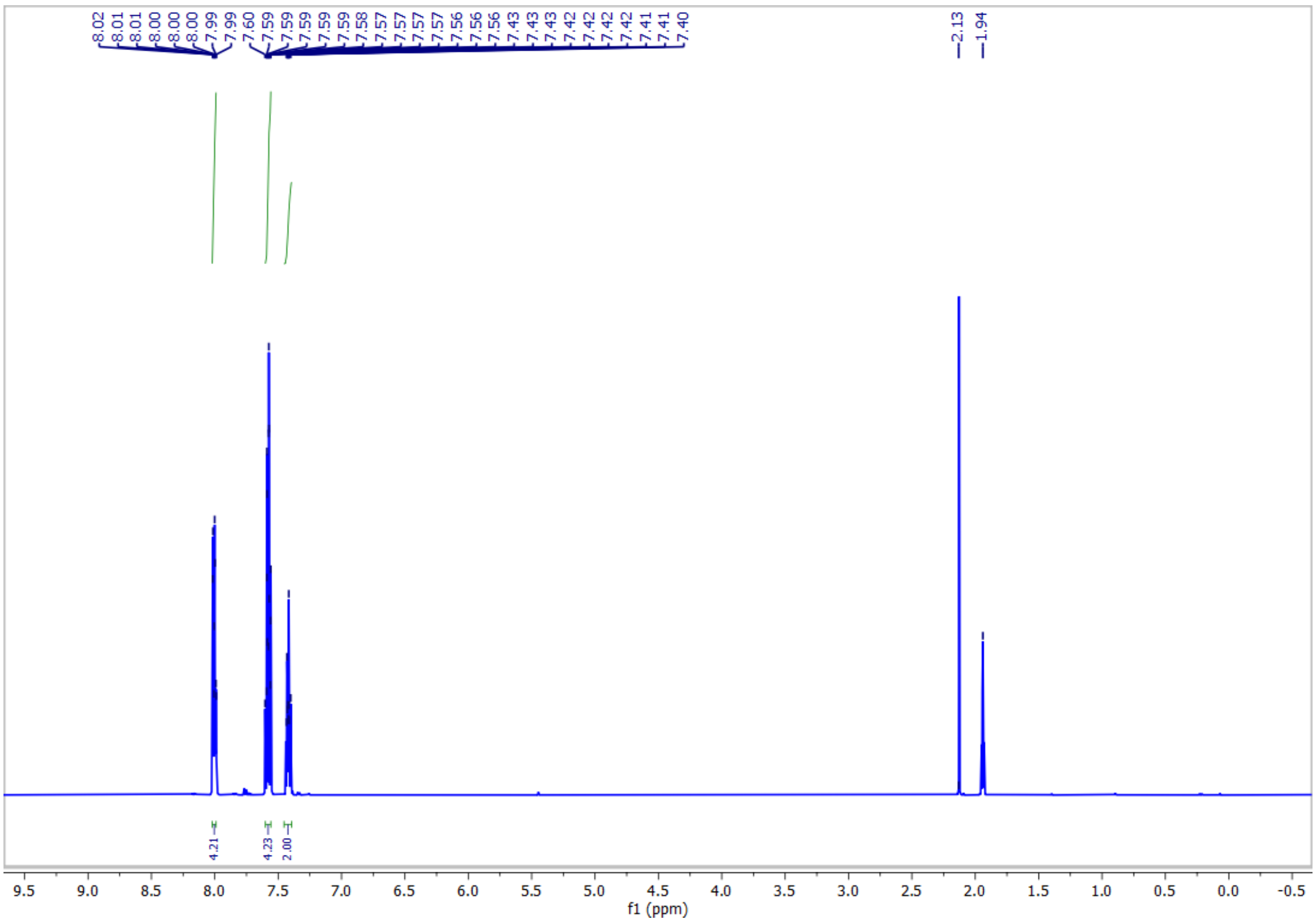

Figure S8. ^{1}H NMR spectrum of compound **1** in CH_3CN-d_3.

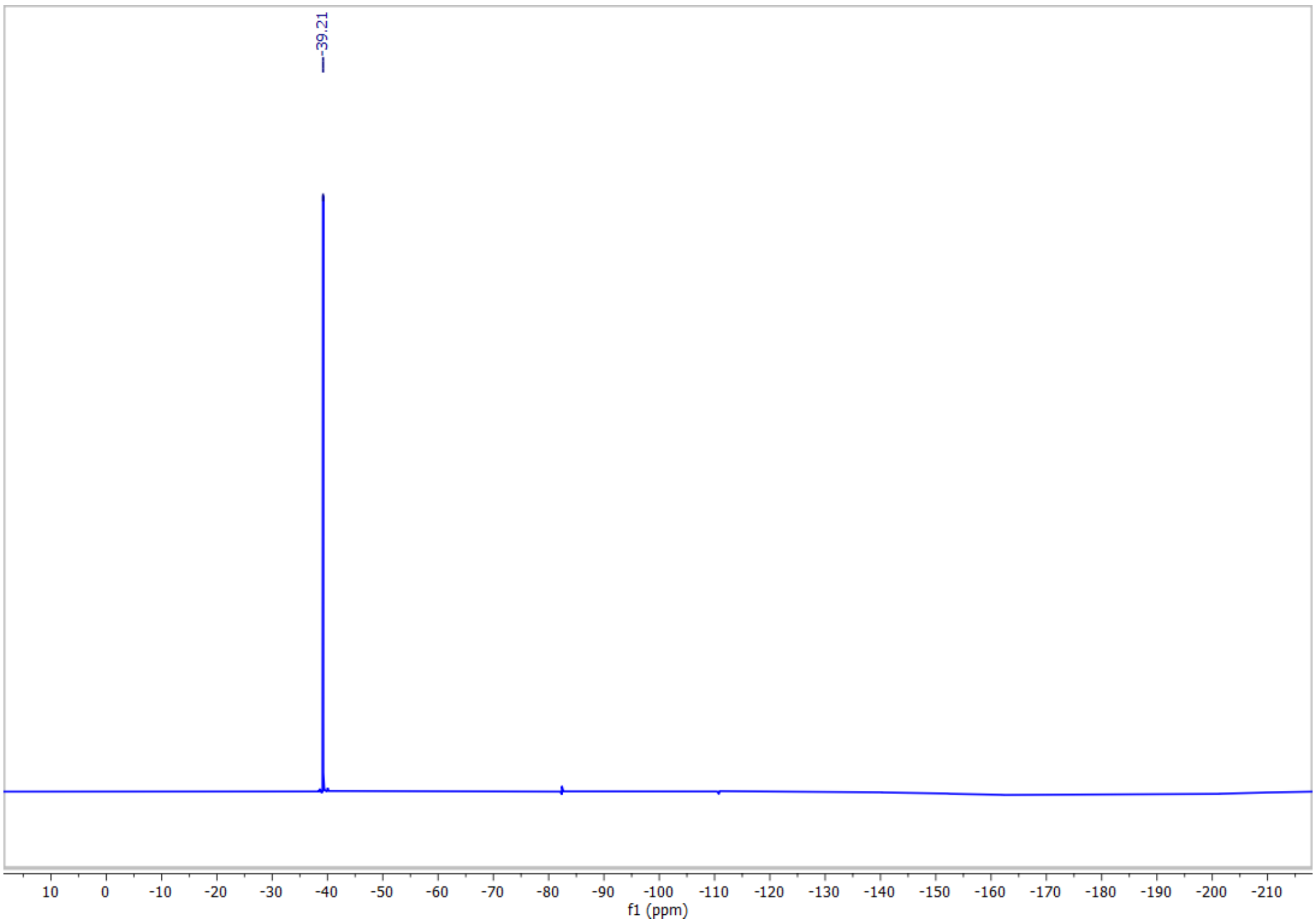

Figure S9. ^{19}F NMR spectrum of compound **1** in THF-d_8.

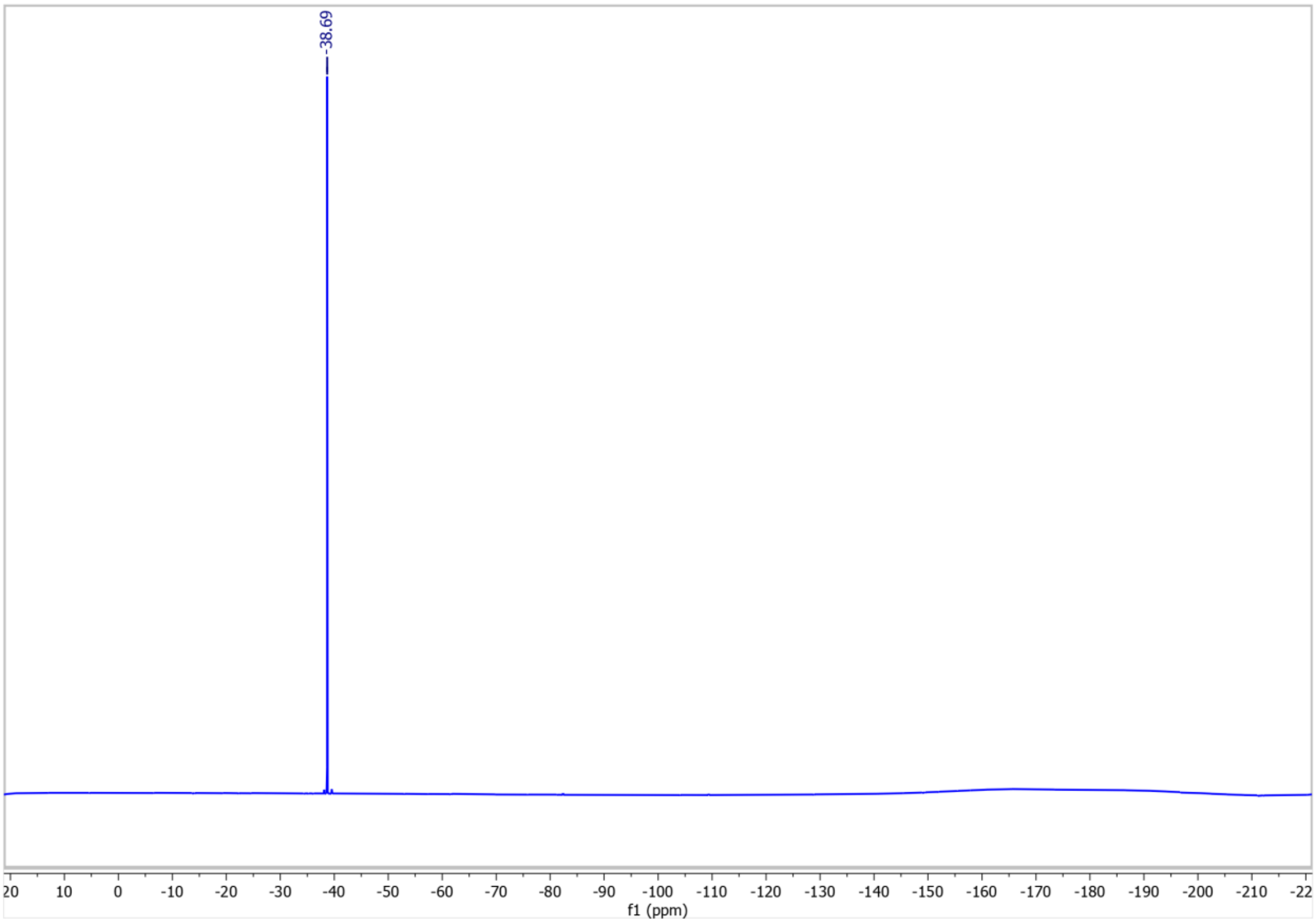

Figure S10. ^{19}F NMR spectrum of compound **1** in CH_3CN-*d*$_3$.

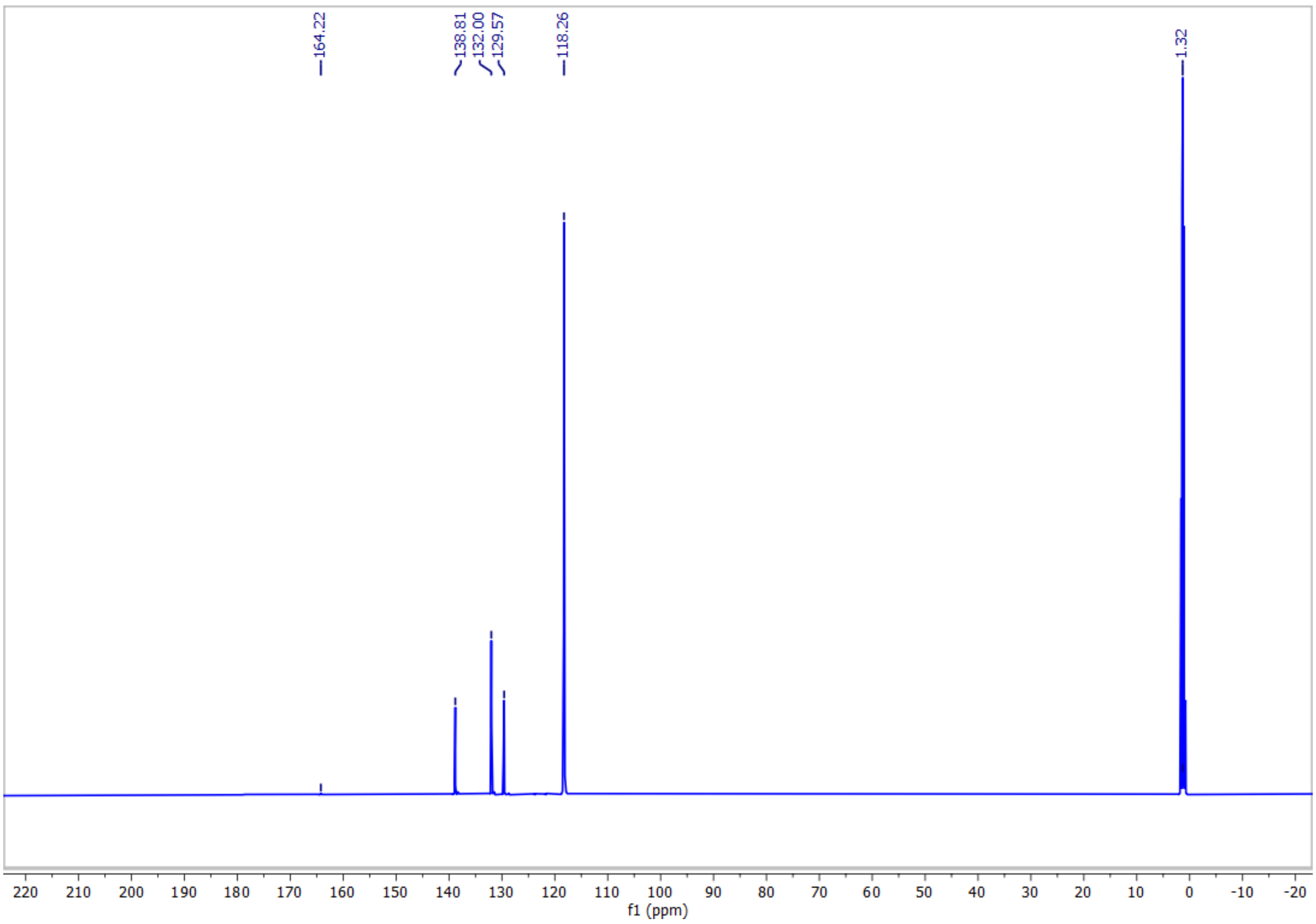

Figure S11. ^{13}C NMR spectrum of compound **1** in CH_3CN-*d*$_3$.

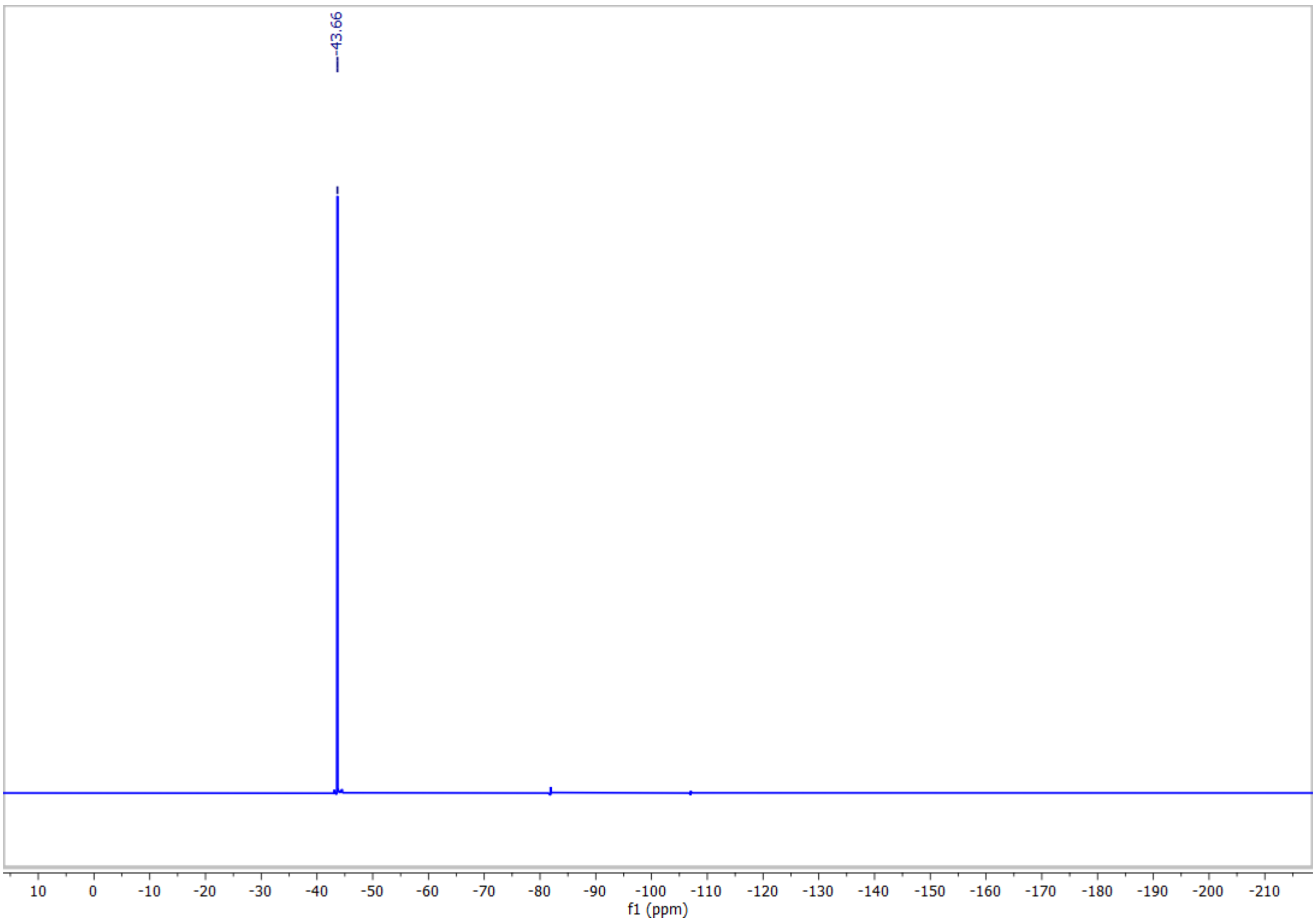

Figure S12. ^{19}F NMR spectrum of compound **2** in THF-d_8.

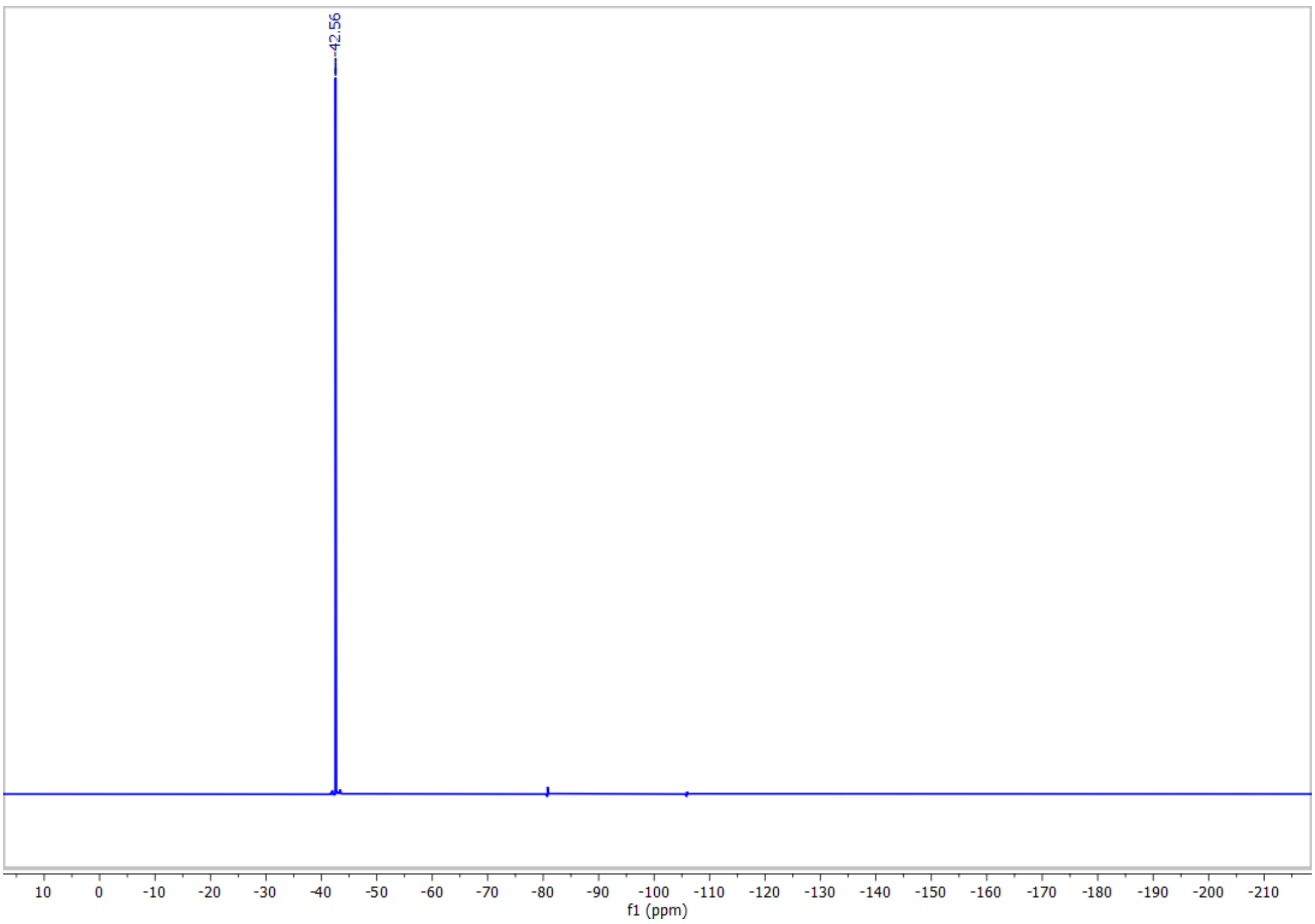

Figure S13. ^{19}F NMR spectrum of compound **2** in DCM-d_2.

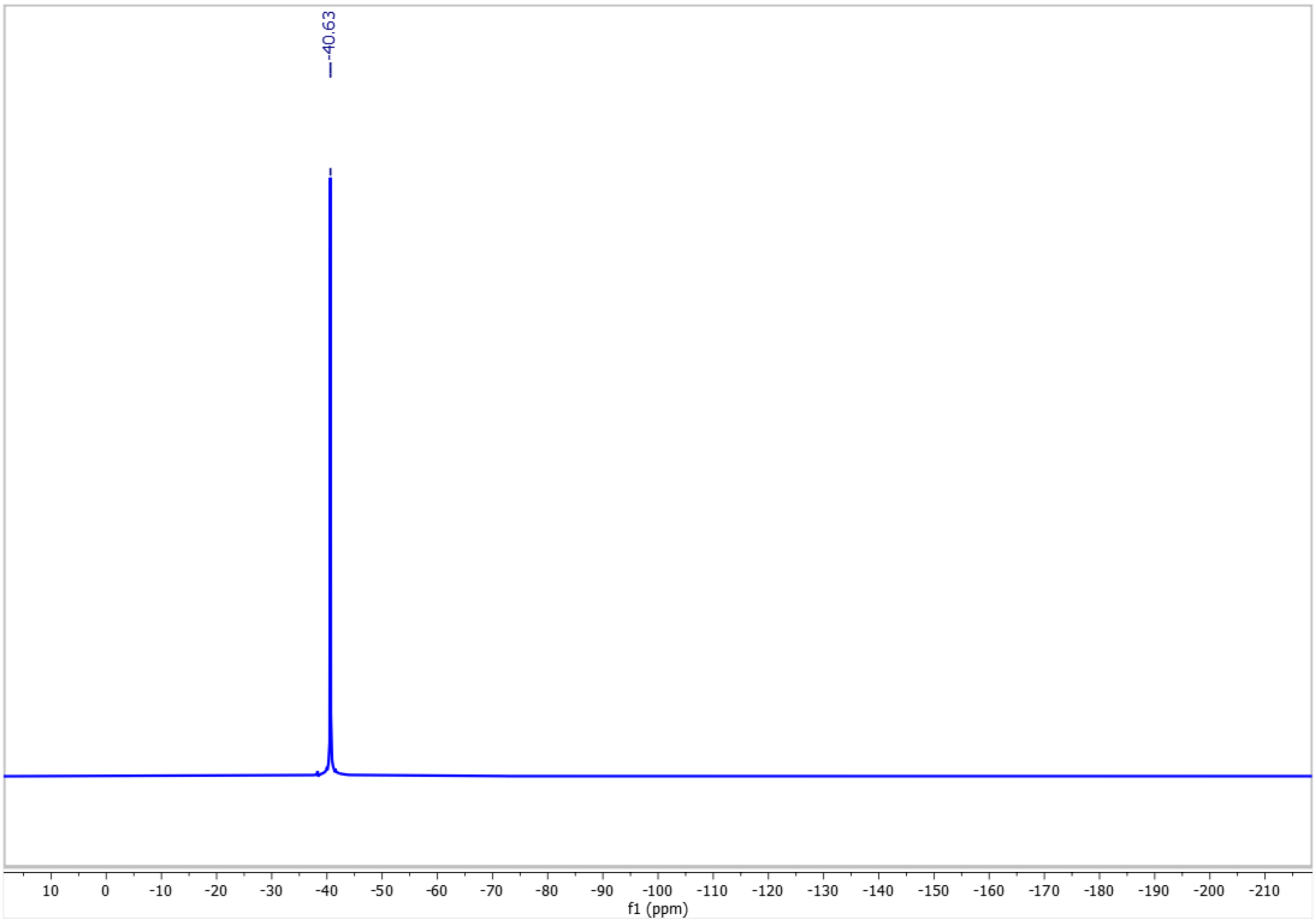

Figure S14. ^{19}F NMR spectrum of compound **2** in pyridine-d_5.

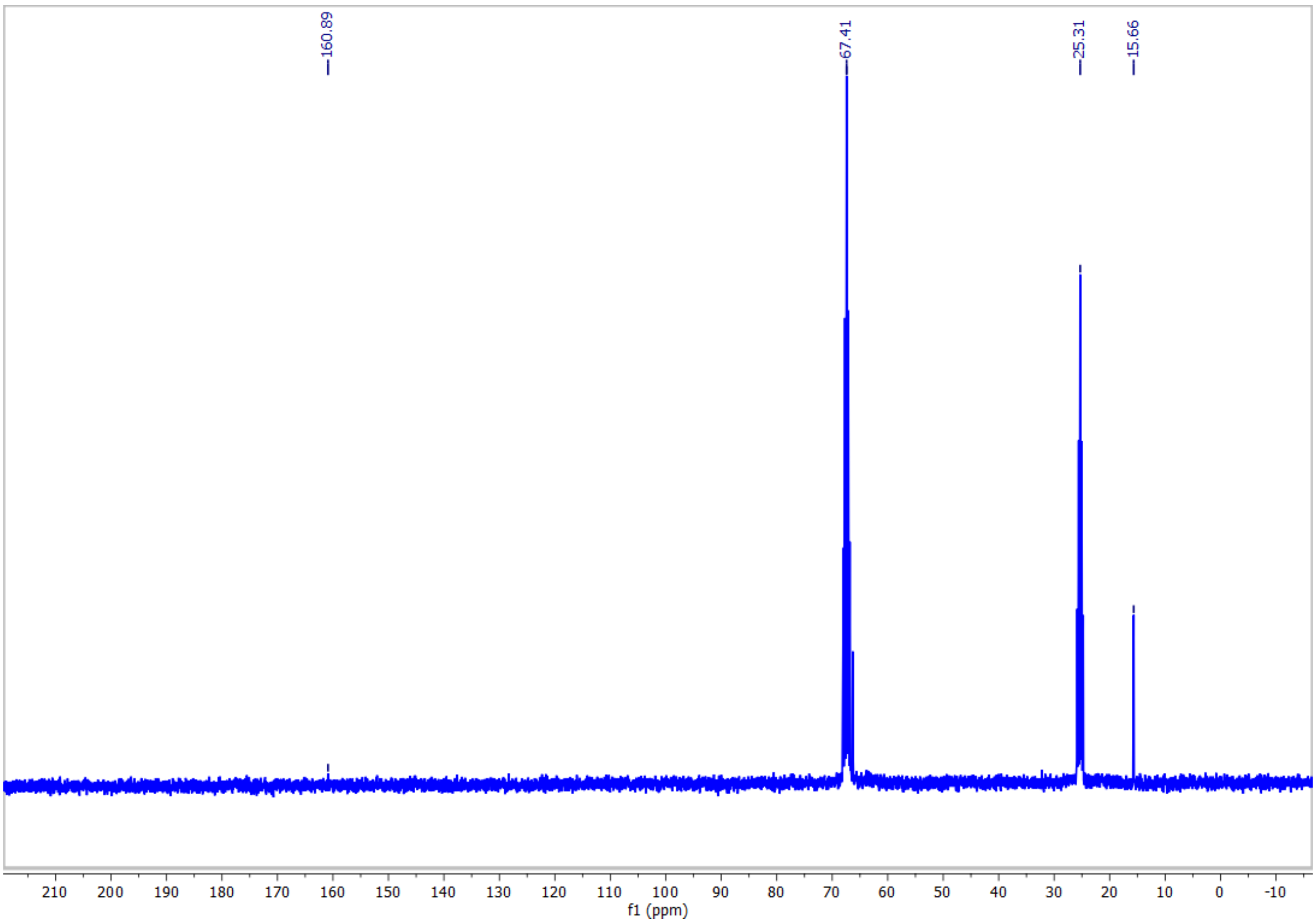

Figure S15. ^{13}C NMR spectrum of compound **2** in THF-d_8.

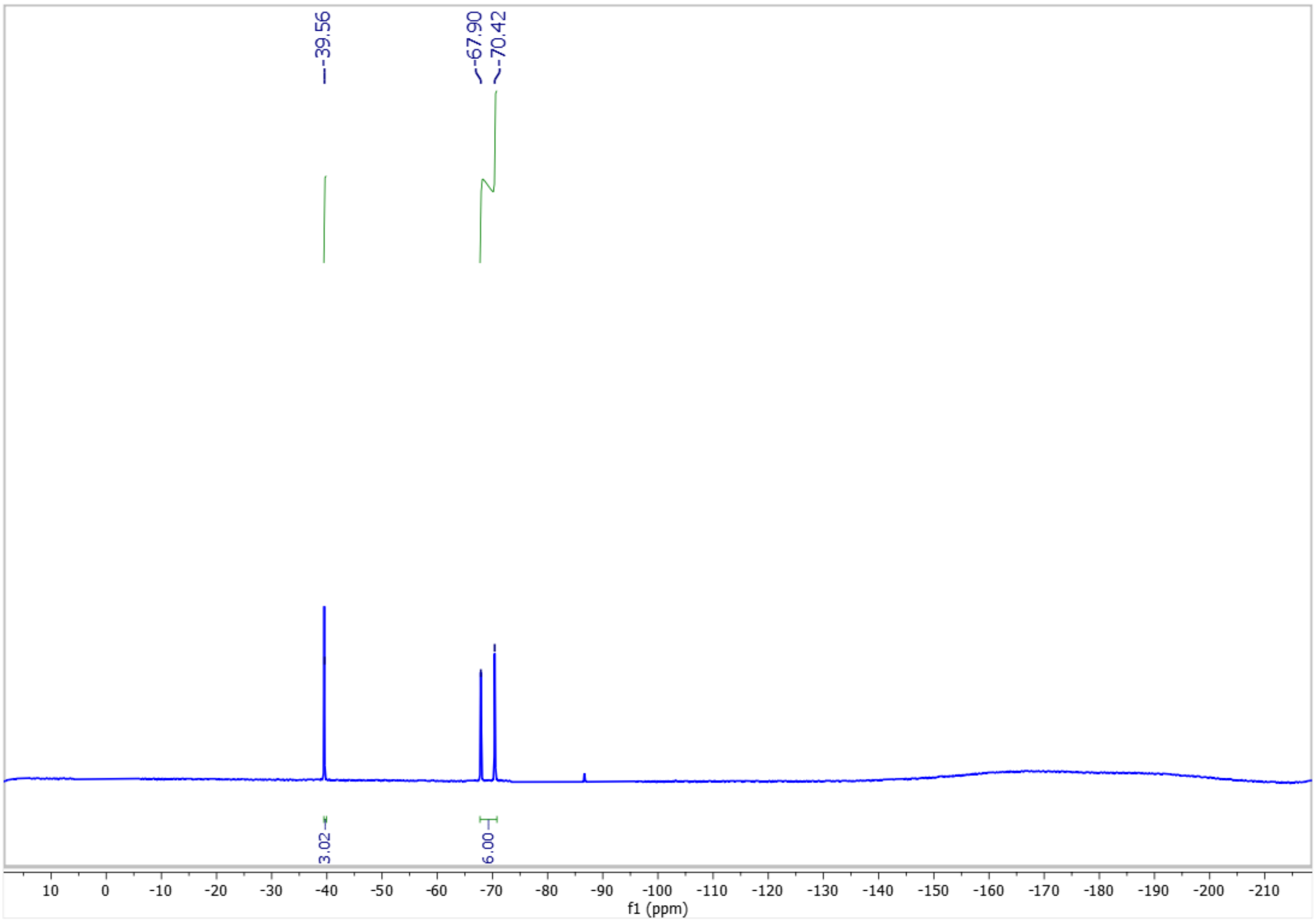

Figure S16. ^{19}F NMR spectrum of compound **3-PF₆** in DCM-*d*₂.

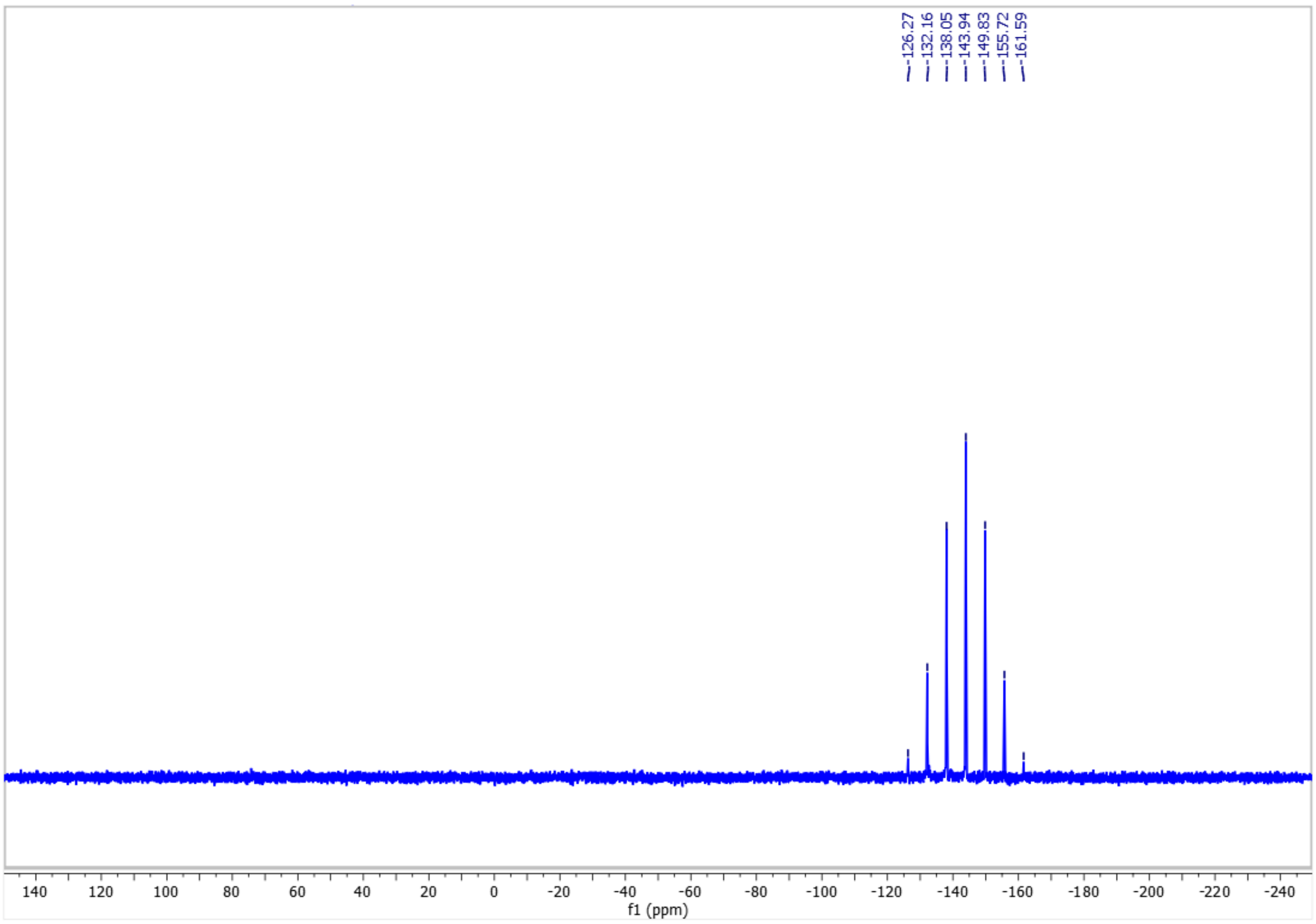

Figure S17. ^{31}P NMR spectrum of compound **3-PF₆** in DCM-*d*₂.

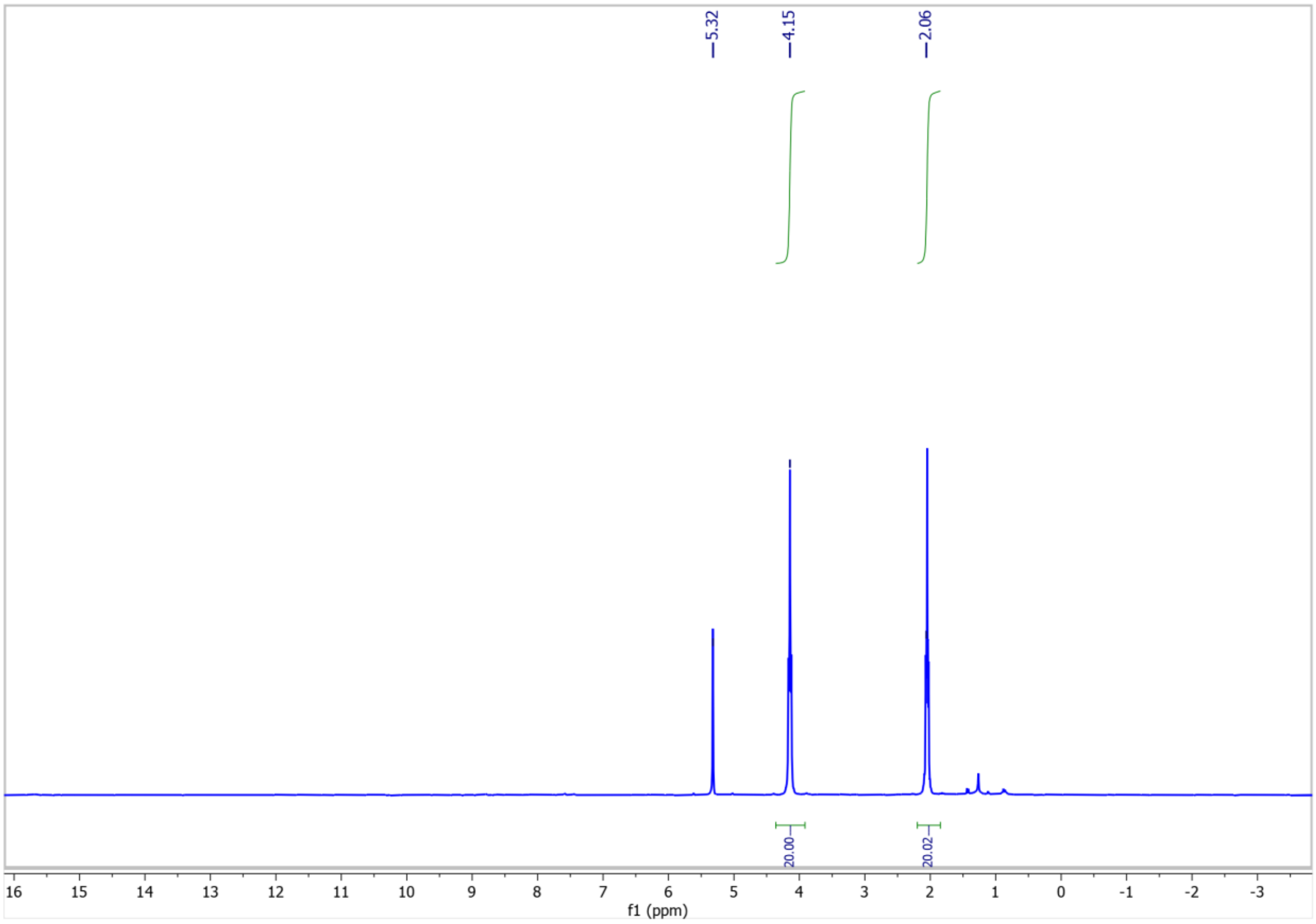

Figure S18. ^{1}H NMR spectrum of compound **3-PF₆** in DCM-*d*₂.

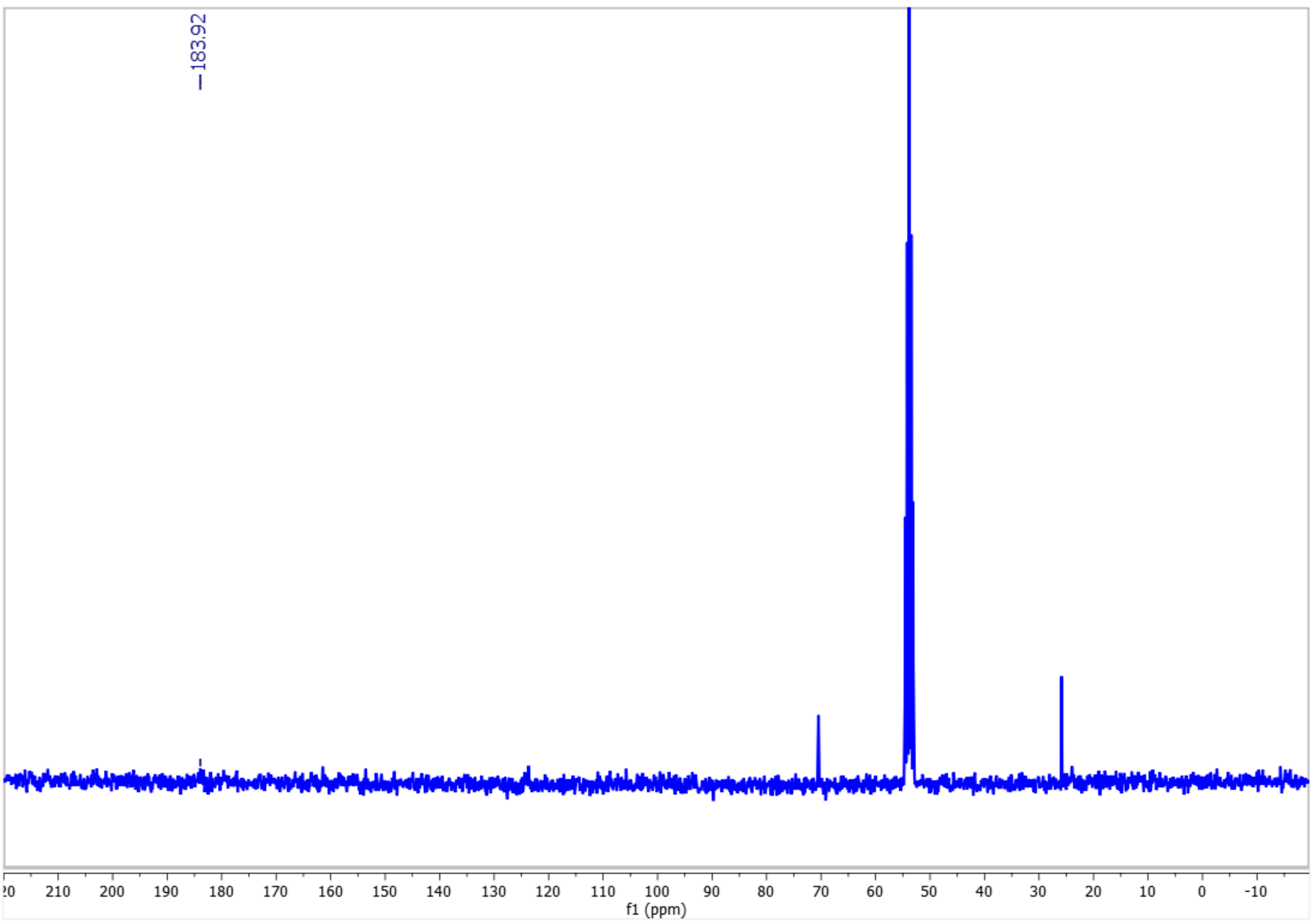

Figure S19. ^{13}C NMR spectrum of compound **3-PF₆** in DCM-*d*₂.

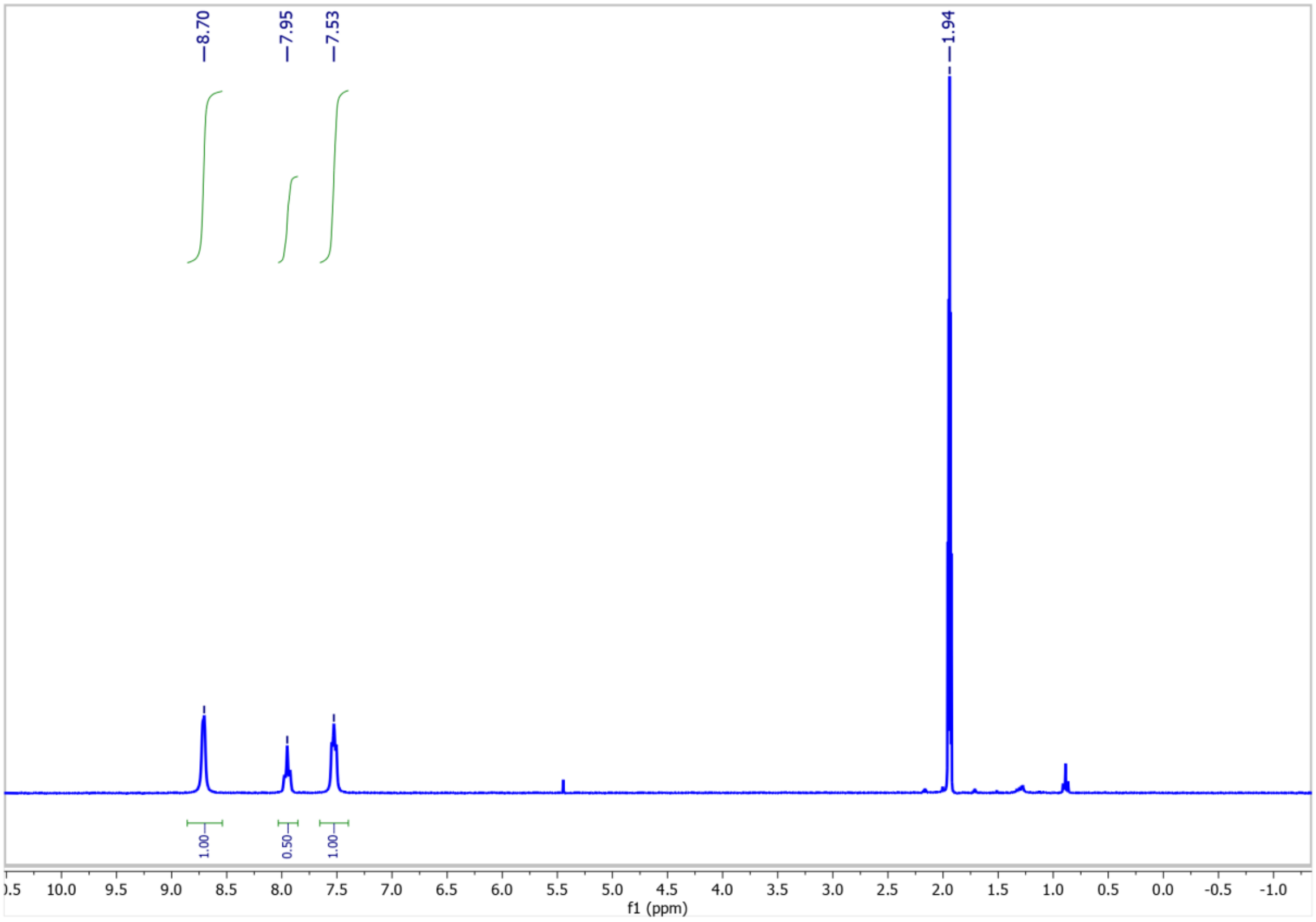

Figure S20. ^{1}H NMR spectrum of compound **3-py-PF₆** in CH_3CN-*d*₃.

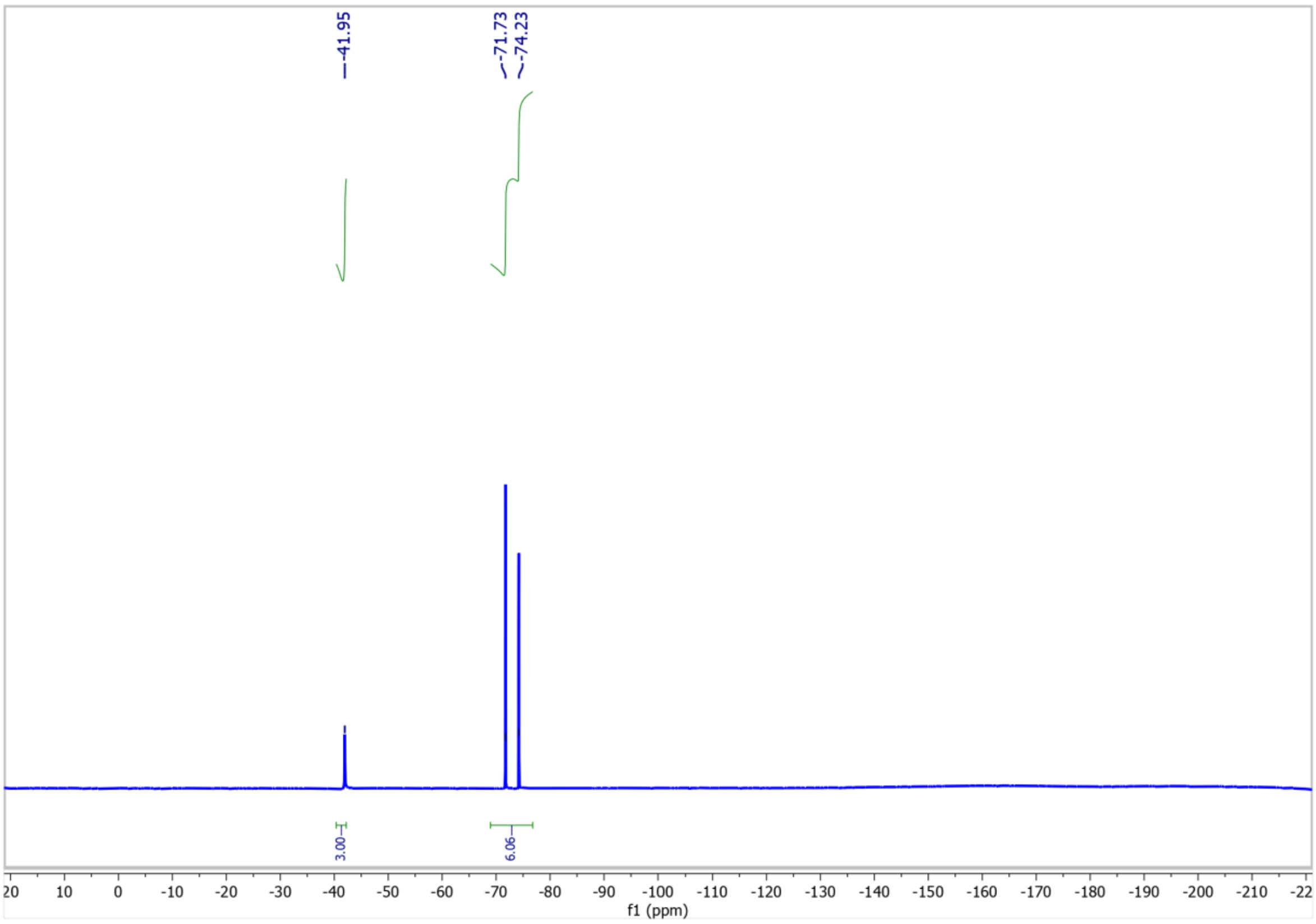

Figure S21. ^{19}F NMR spectrum of compound **3-py-PF₆** in CH_3CN-*d*₃.

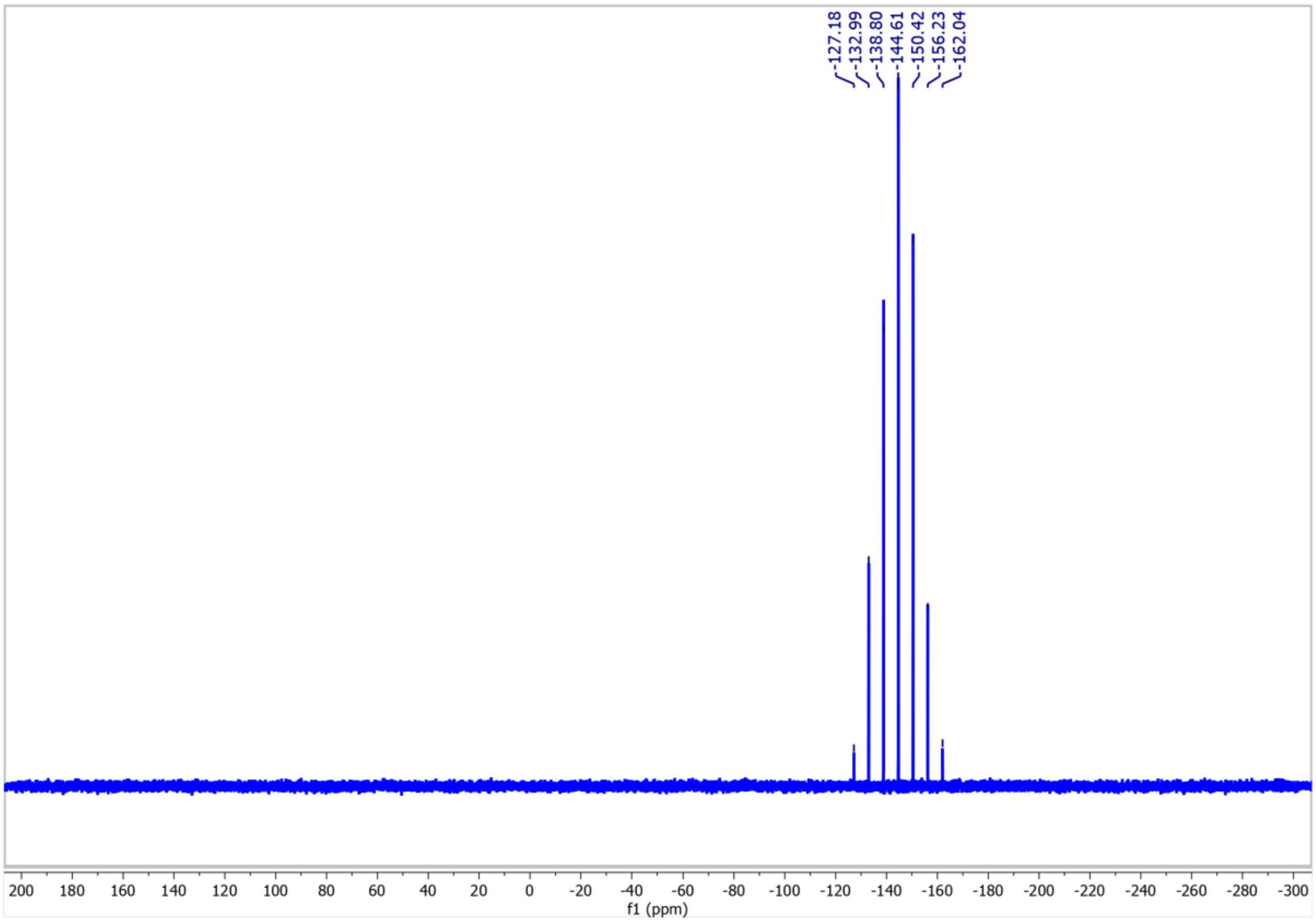

Figure S22. ^{31}P NMR spectrum of compound **3-py-PF₆** in CH_3CN-d_3.

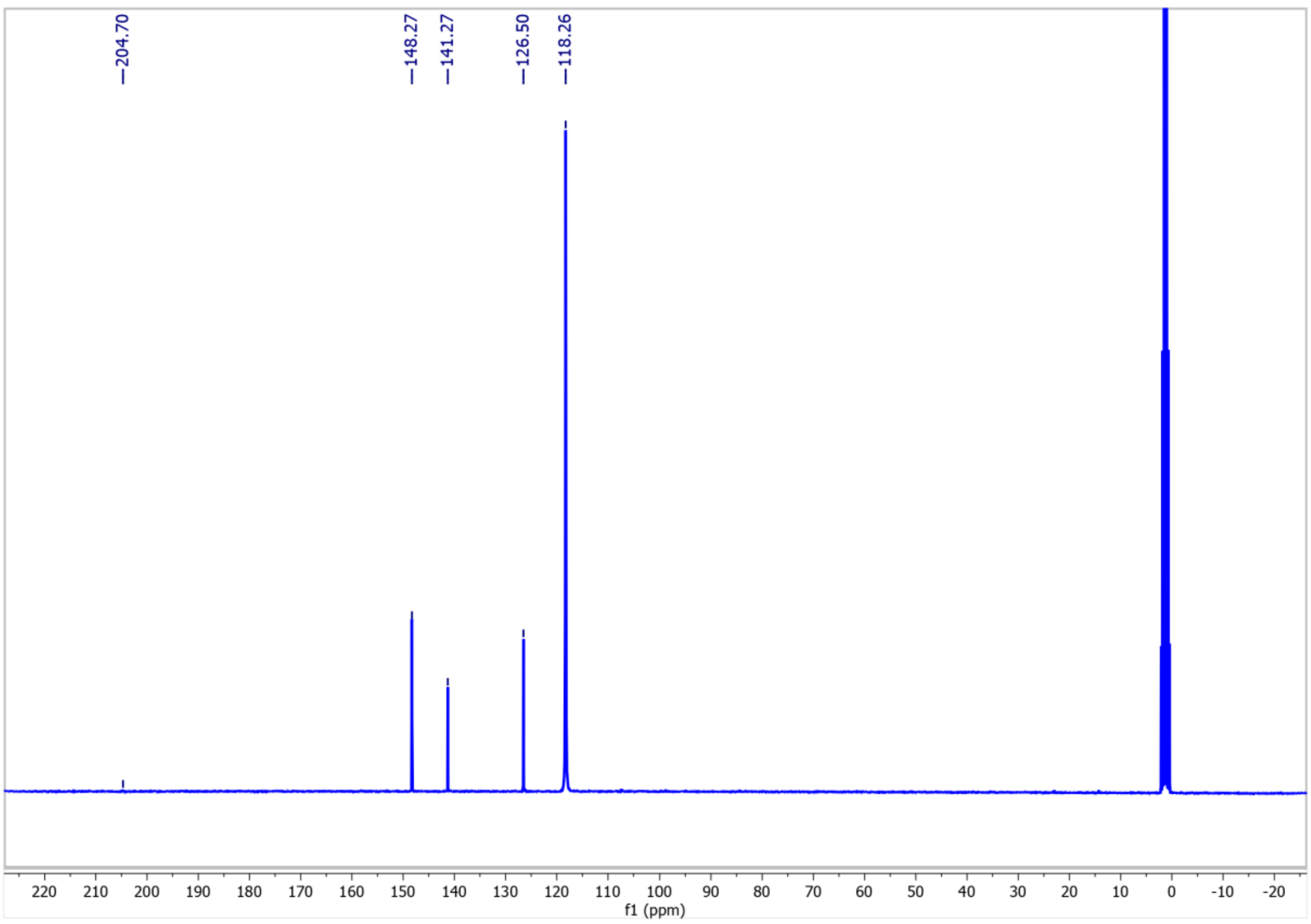

Figure S23. ^{13}C NMR spectrum of compound **3-py-PF₆** in CH_3CN-d_3.

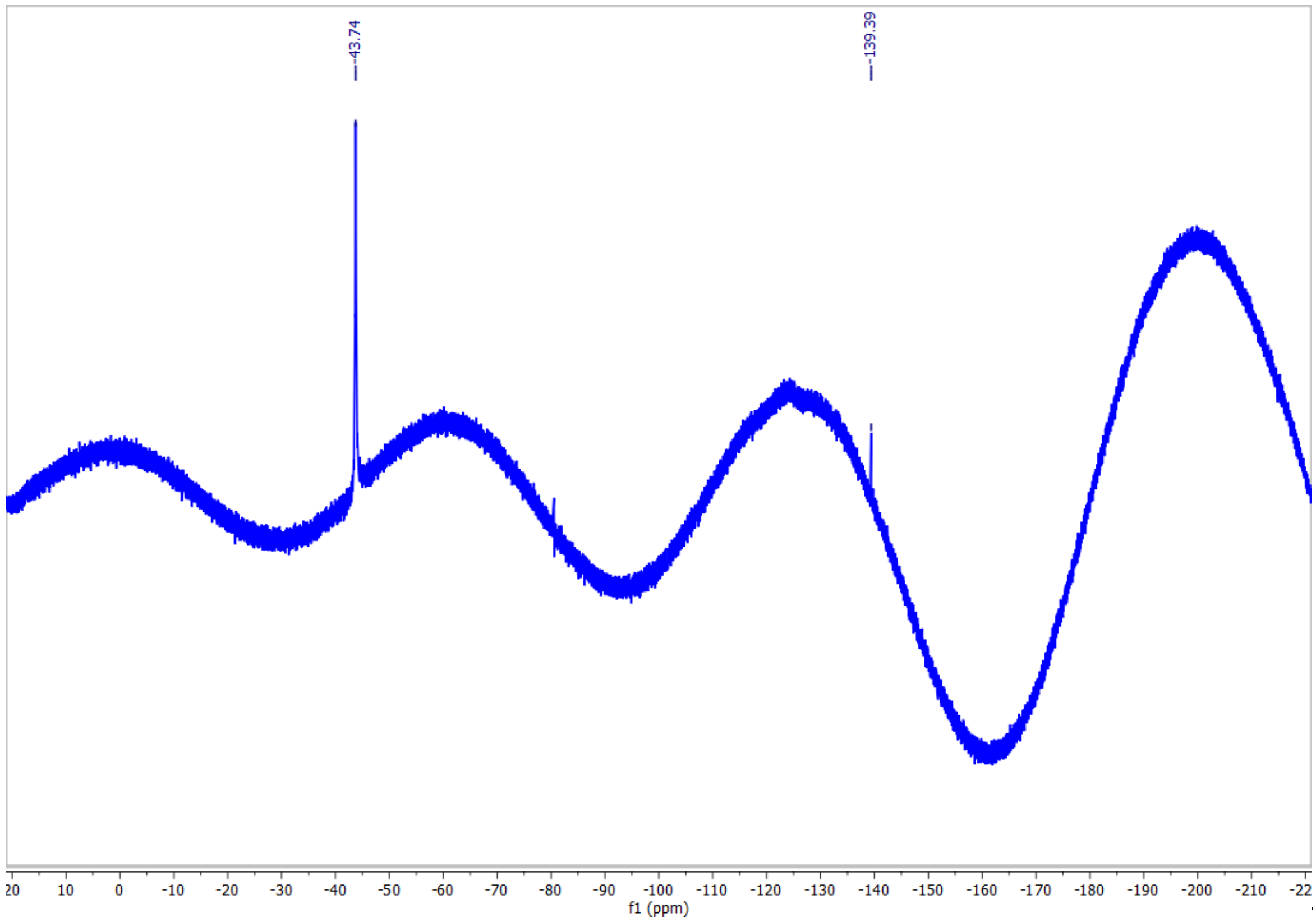

Figure S24. ^{19}F NMR spectrum of compound **3-SbF6** in DCM-d_2.

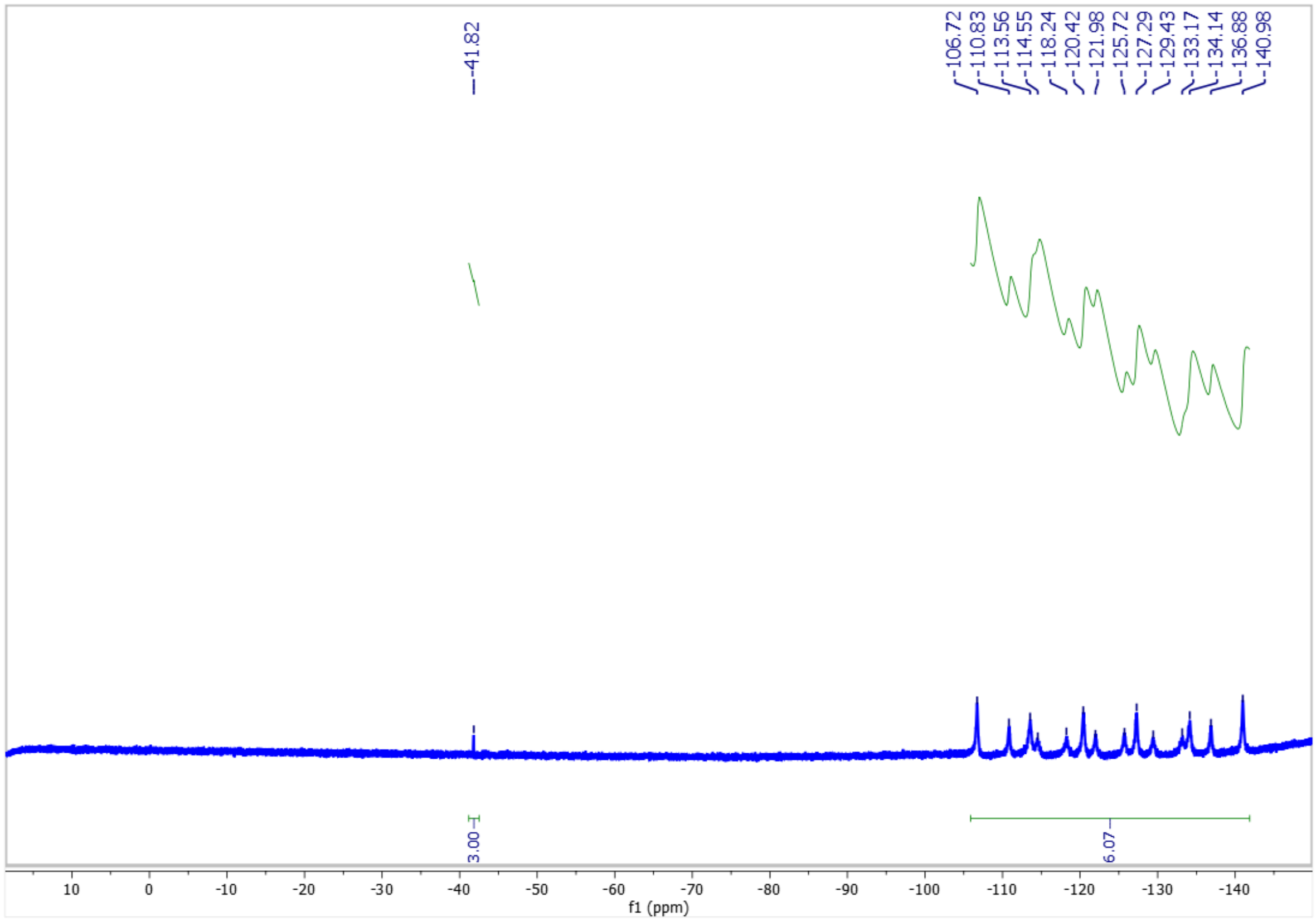

Figure S25. ^{19}F NMR spectrum of compound **3-SbF6** in CH_3CN-d_3.

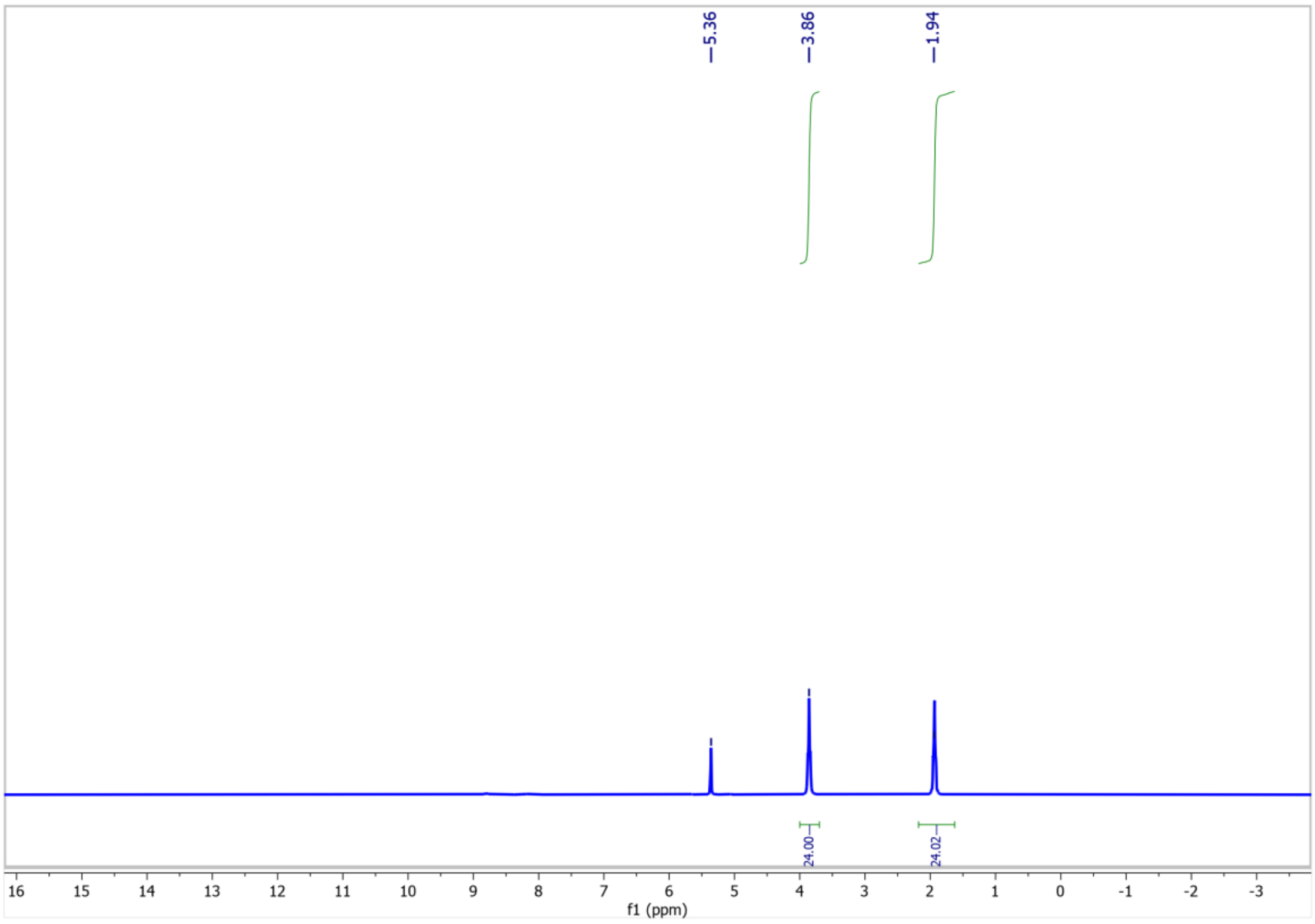

Figure S26. ^{1}H NMR spectrum of compound **3-SbF₆** in DCM-d_2.

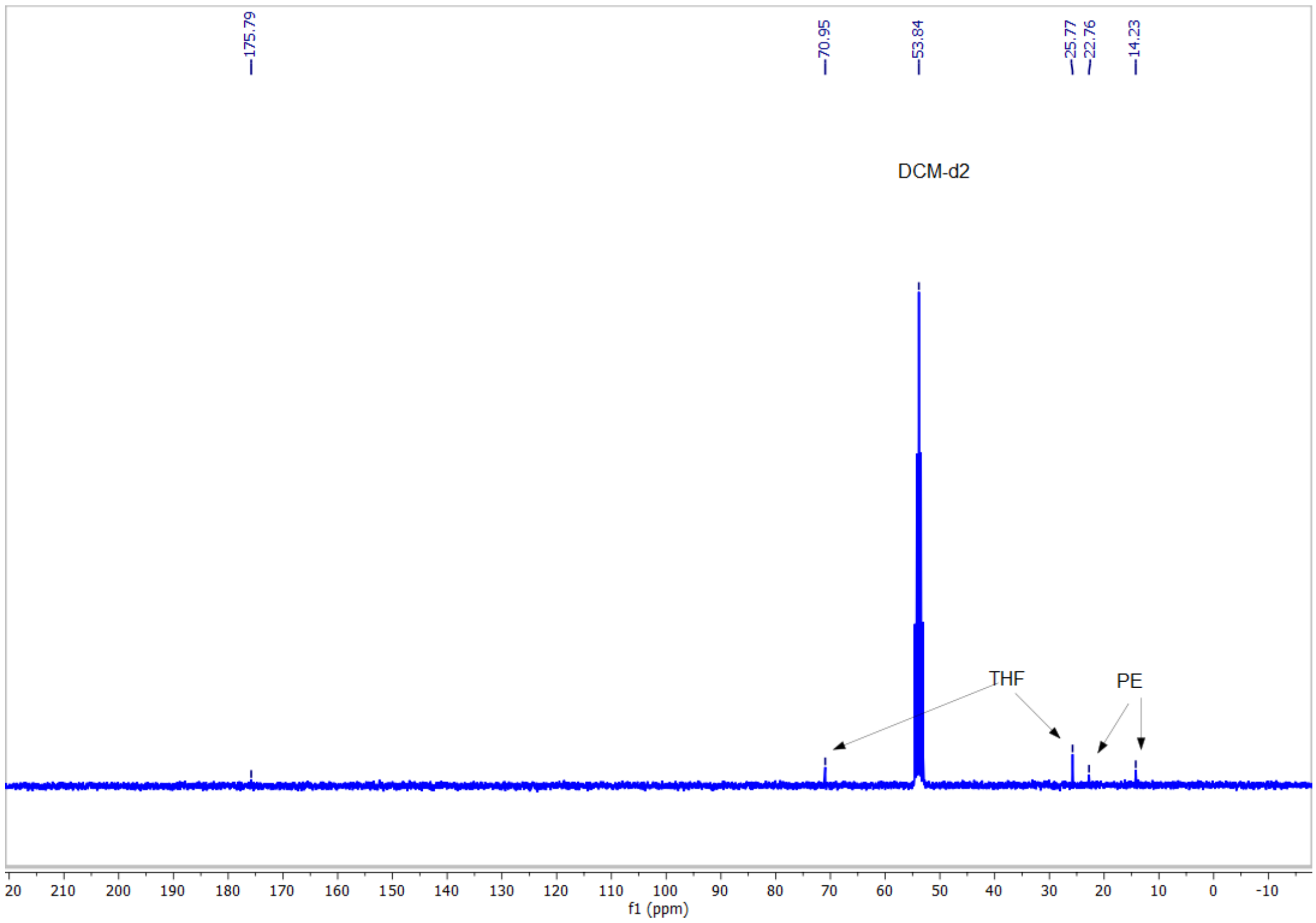

Figure S27. ^{13}C NMR spectrum of compound **3-SbF₆** in DCM-d_2. PE is pentane residual solvent.

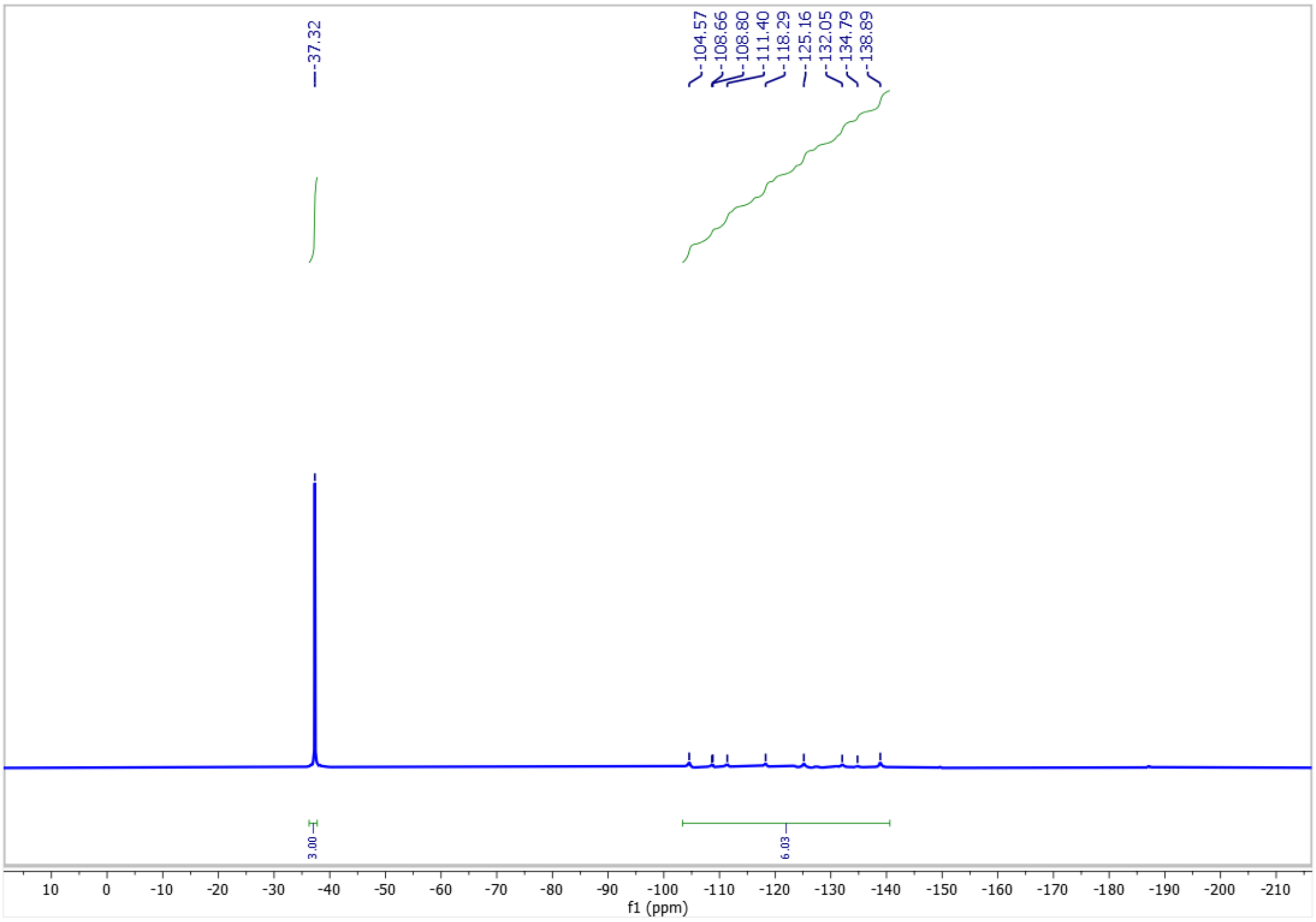

Figure S28. ^{19}F NMR spectrum of compound **3-py-SbF$_6$** in CH_3CN-*d*$_3$.

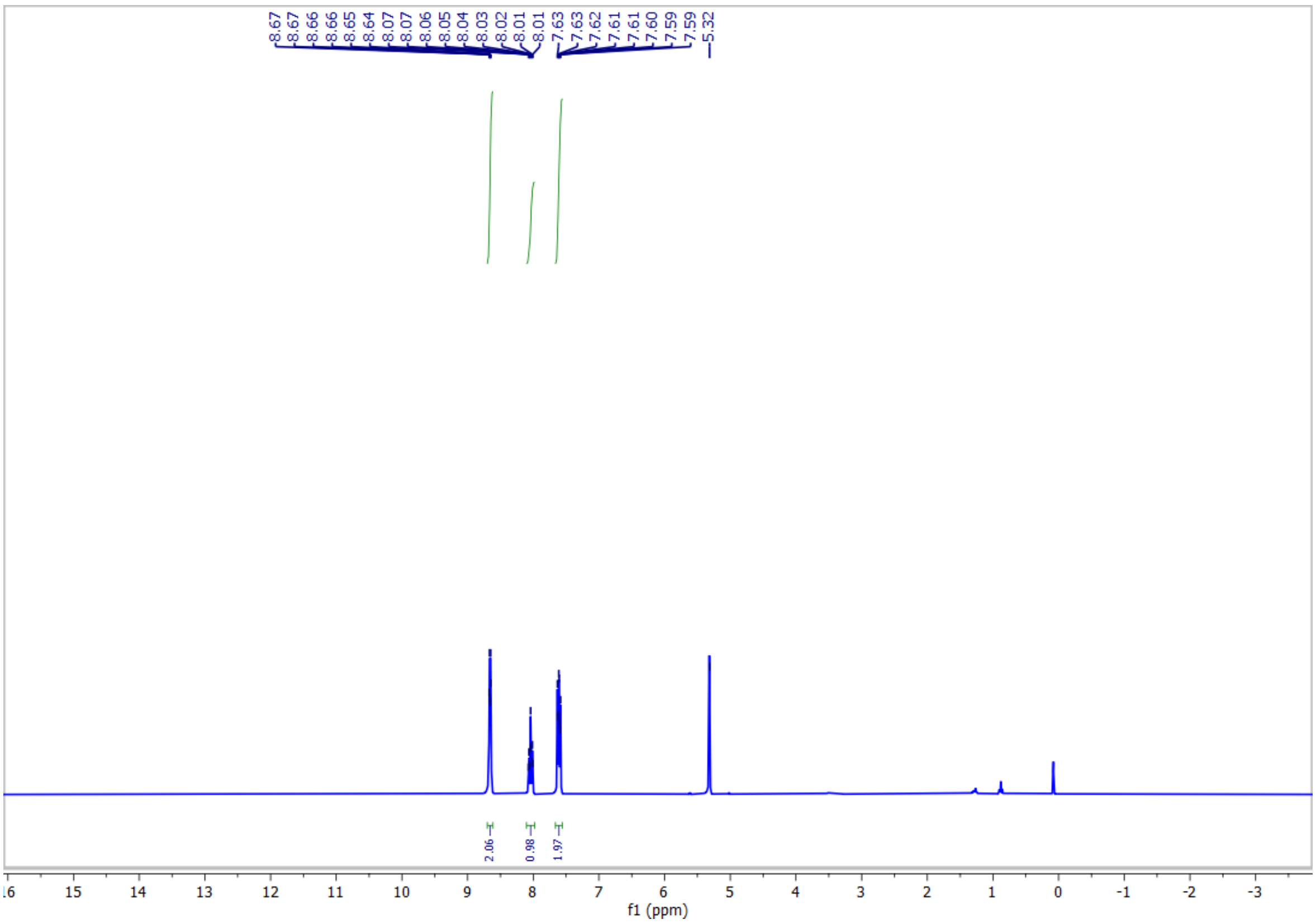

Figure S29. ^{1}H NMR spectrum of compound **3-py-SbF$_6$** in DCM-*d*$_2$.

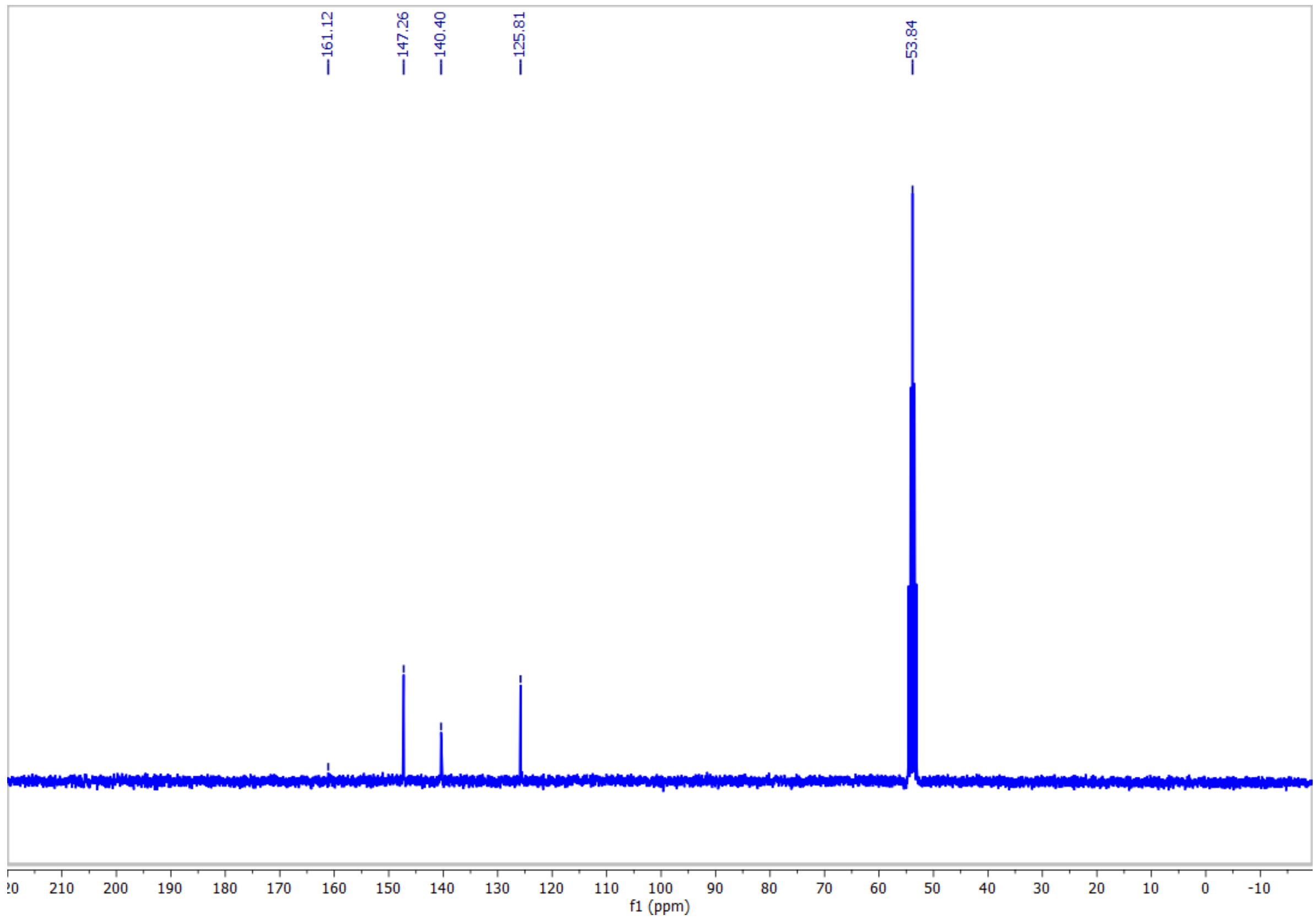

Figure S30. ^{13}C NMR spectrum of compound **3-py-SbF$_6$** in DCM-d_2.

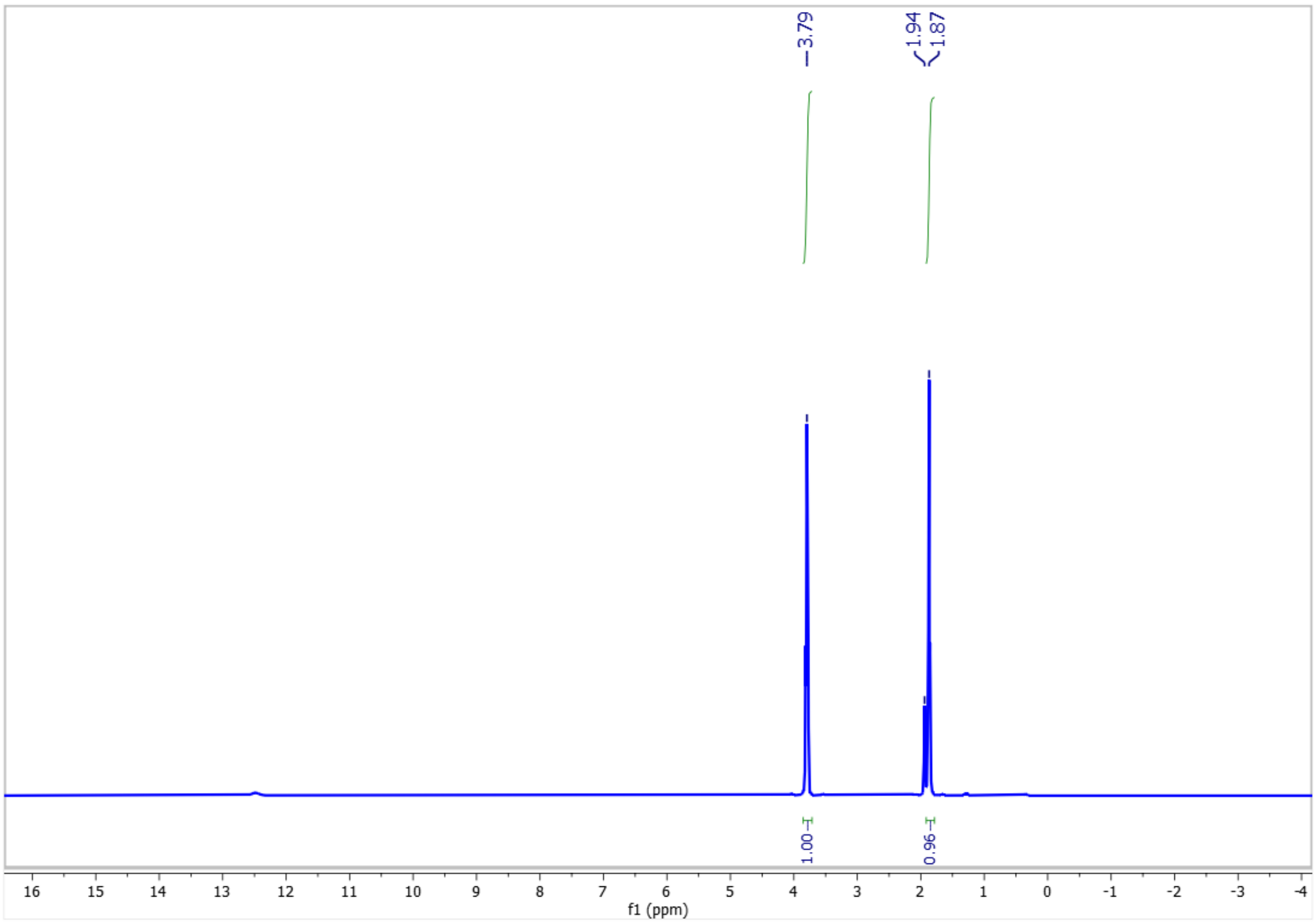

Figure S31. ^{1}H NMR spectrum of compound **4** in MeCN-d_3.

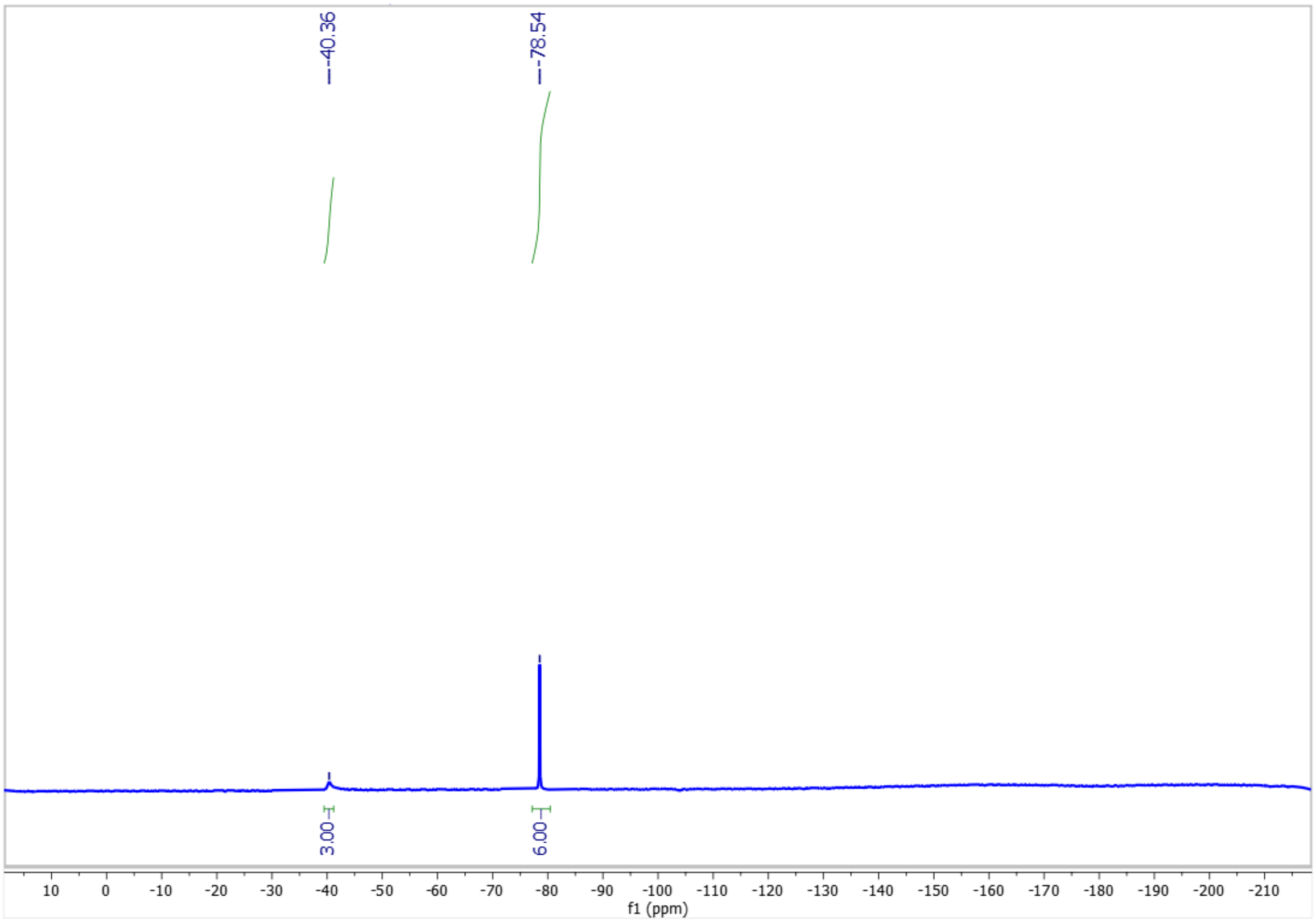

Figure S32. ^{19}F NMR spectrum of compound **4** in MeCN-d_3.

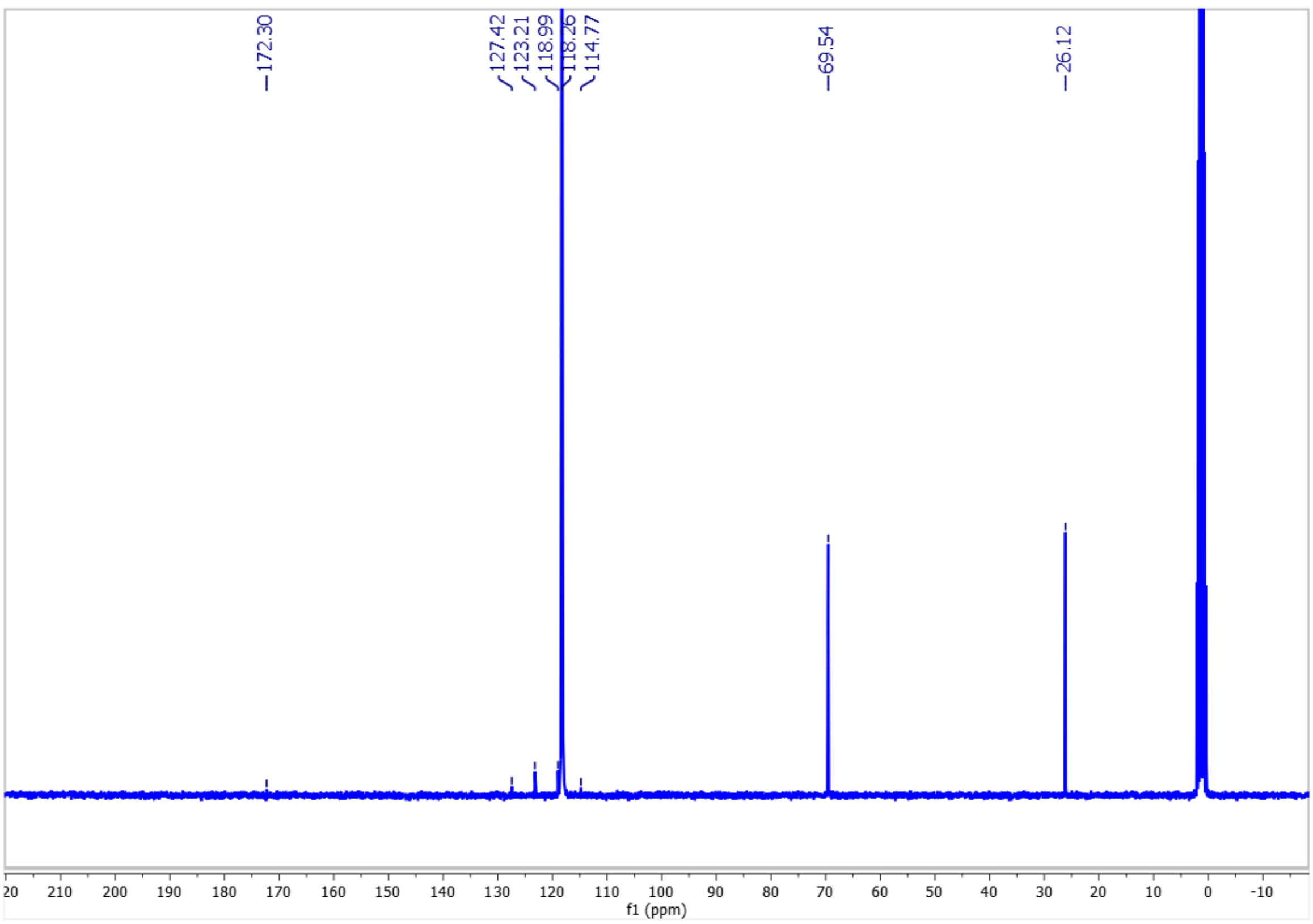

Figure S33. ^{13}C NMR spectrum of compound **4** in MeCN-d_3.

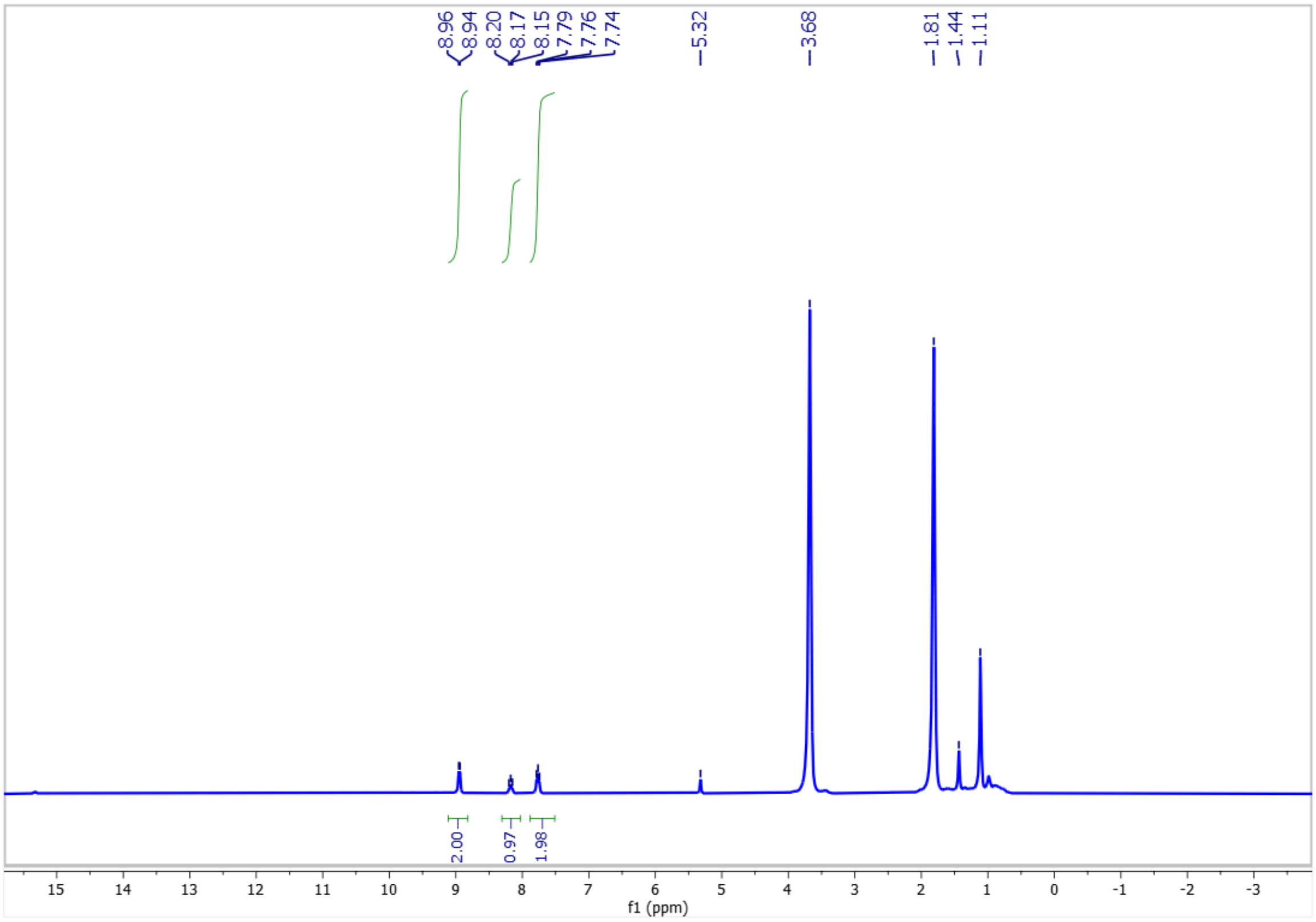

Figure S34. ^{1}H NMR spectrum of compound **4-py** in DCM-*d*$_2$.

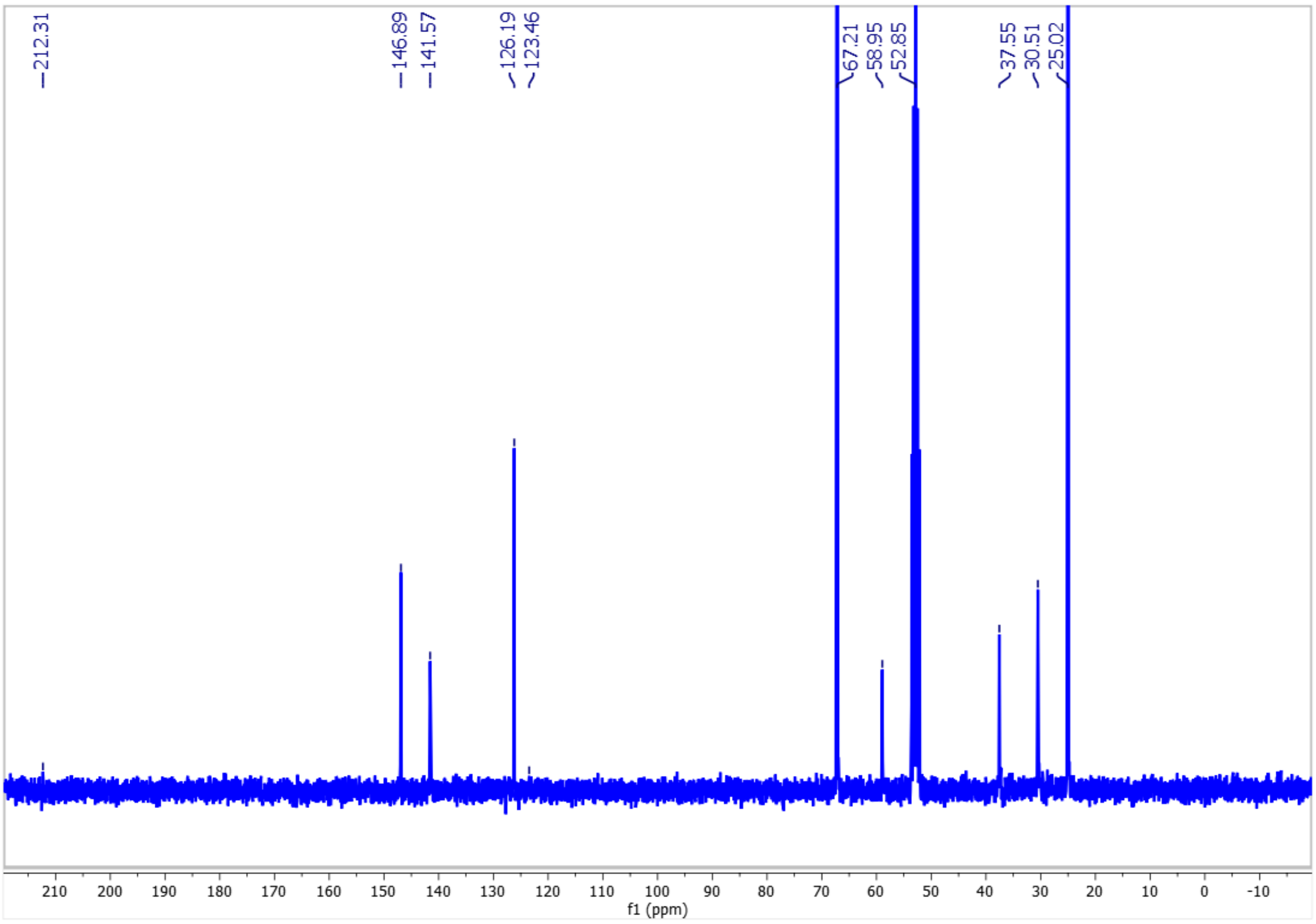

Figure S35. ^{13}C NMR spectrum of compound **4-py** in MeCN-*d*$_3$.

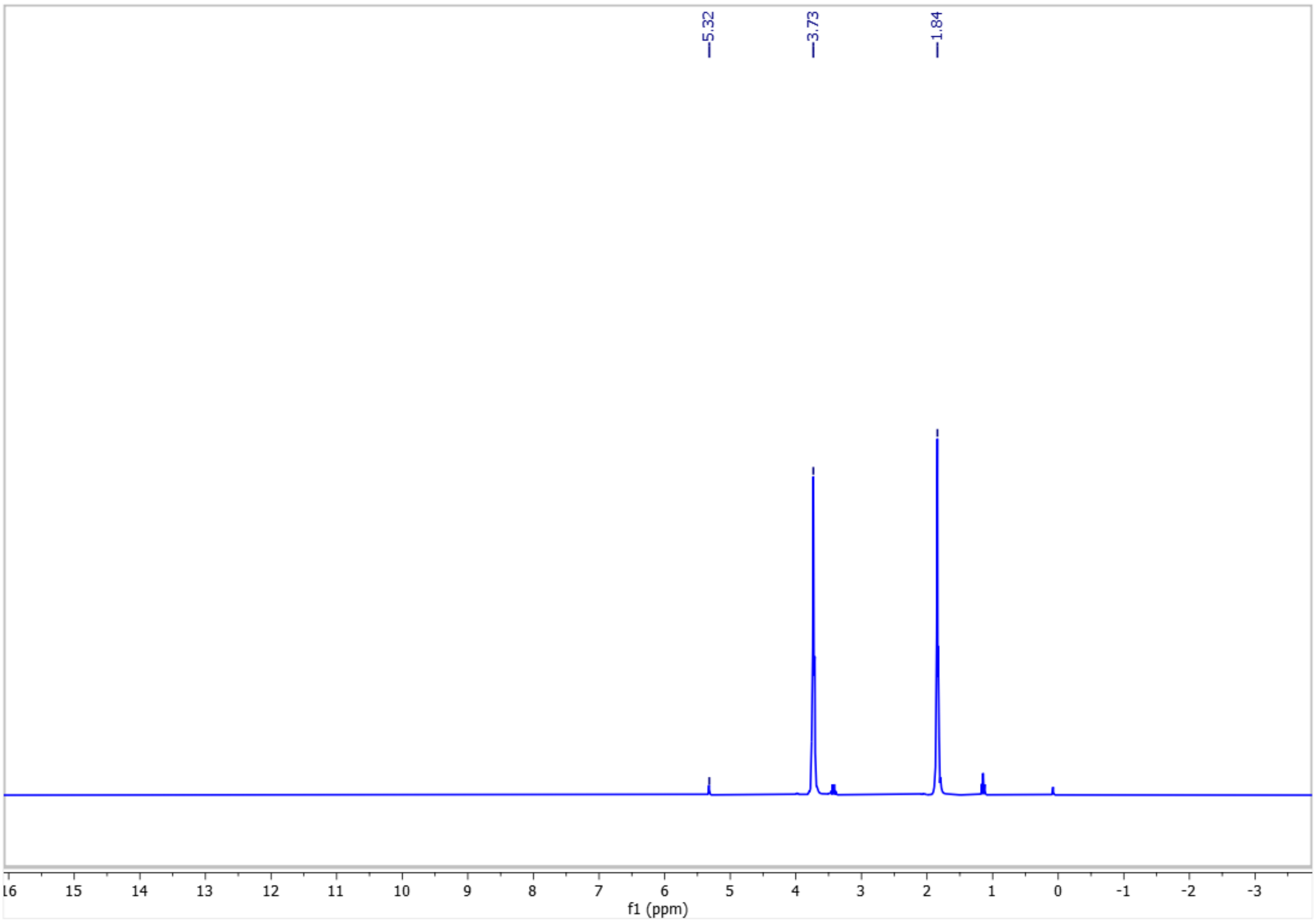

Figure S36. ^{1}H NMR spectrum of compound **5** in DCM-d_2.

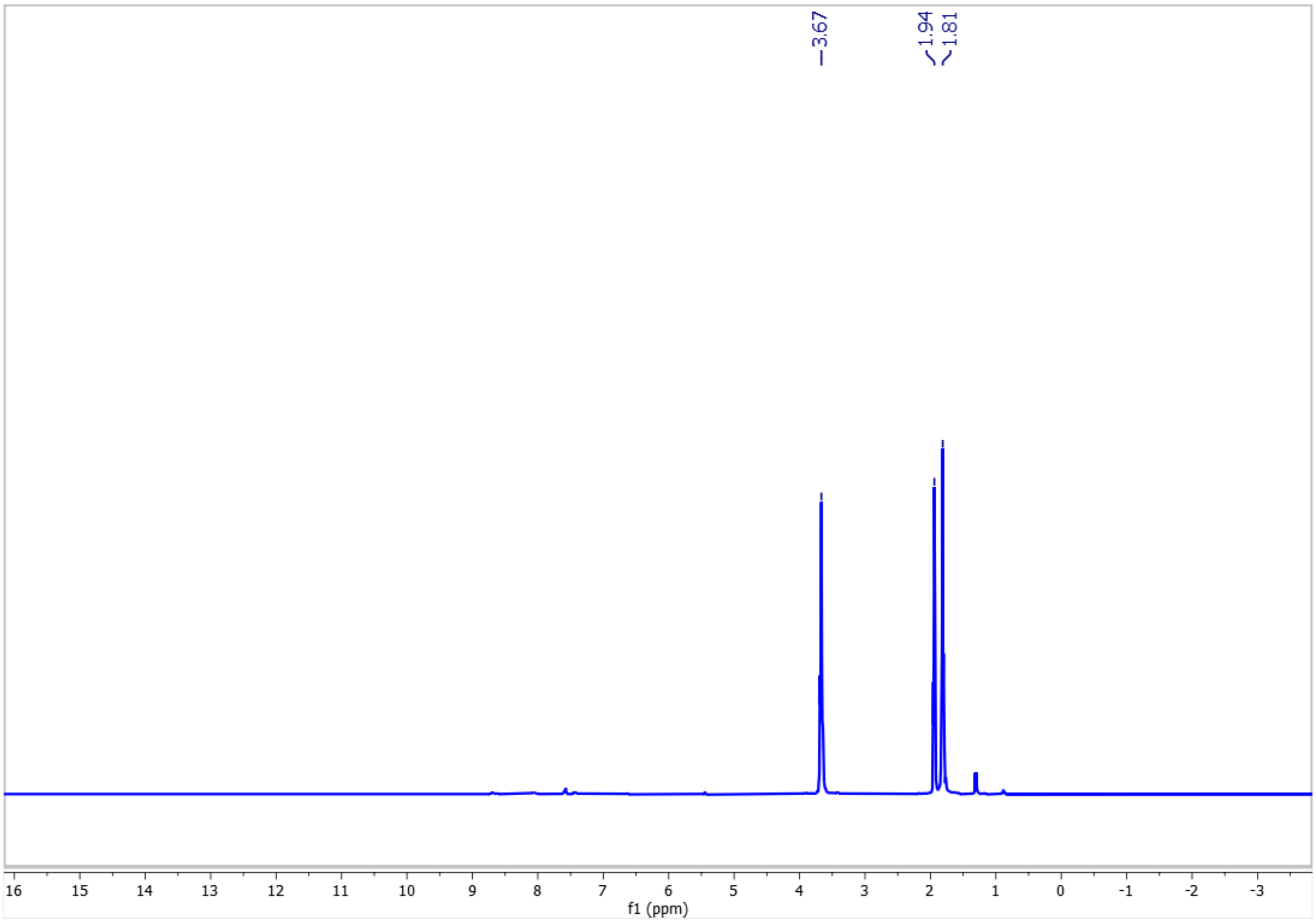

Figure S37. ^{1}H NMR spectrum of compound **5** in MeCN-d_3.

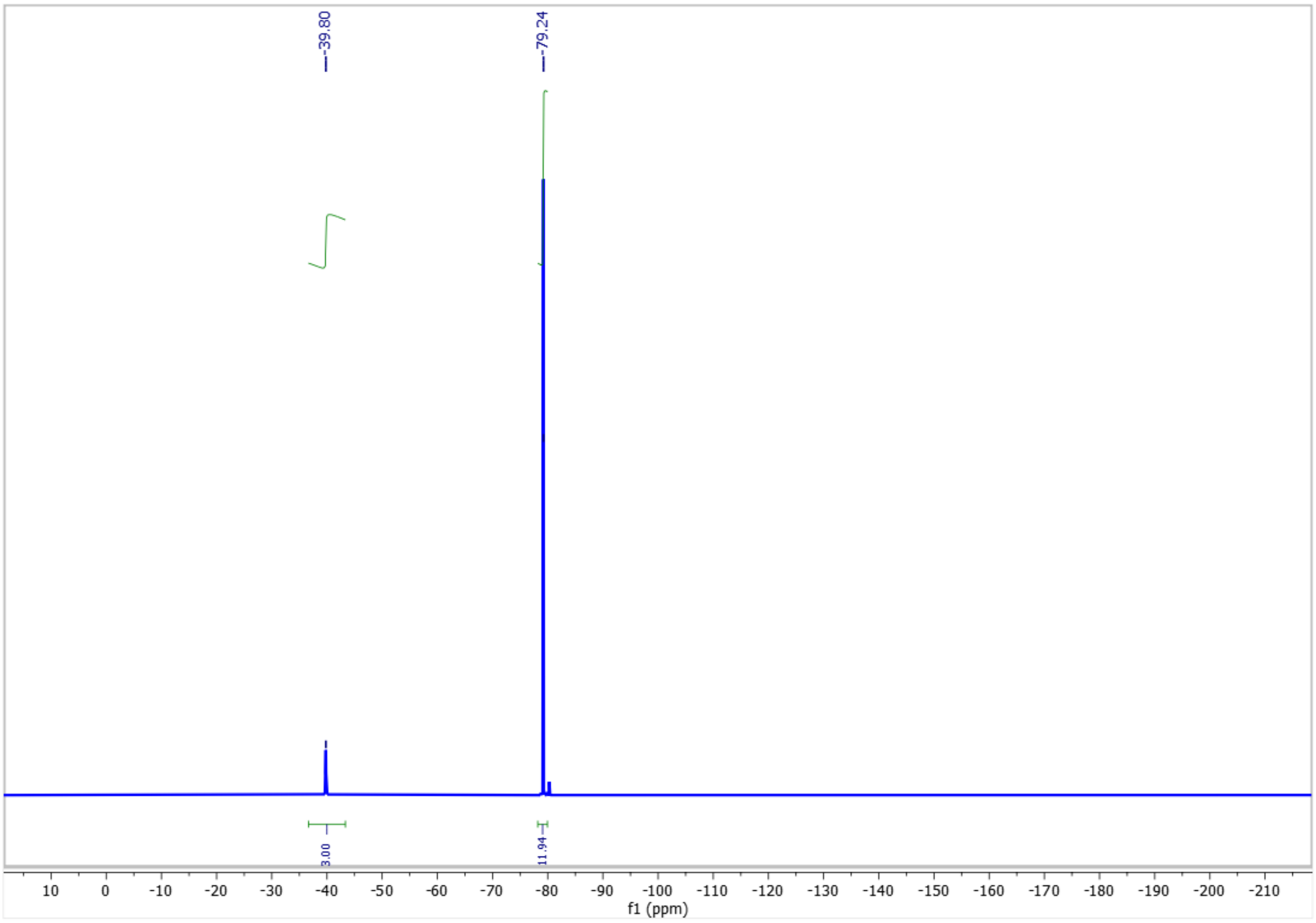

Figure S38. ^{19}F NMR spectrum of compound **5** in DCM-d_2.

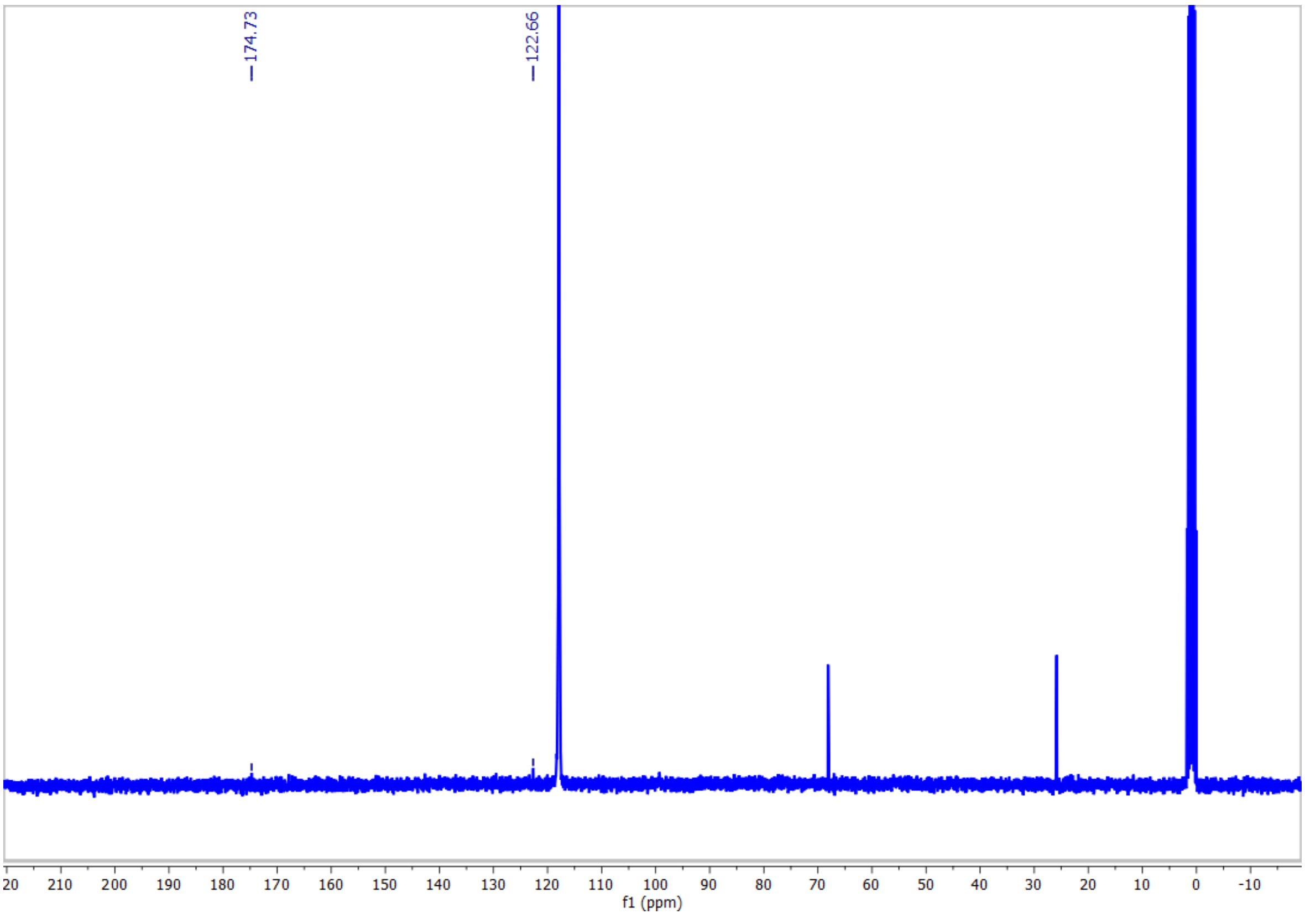

Figure S39. ^{13}C NMR spectrum of compound **5** in MeCN-d_3.

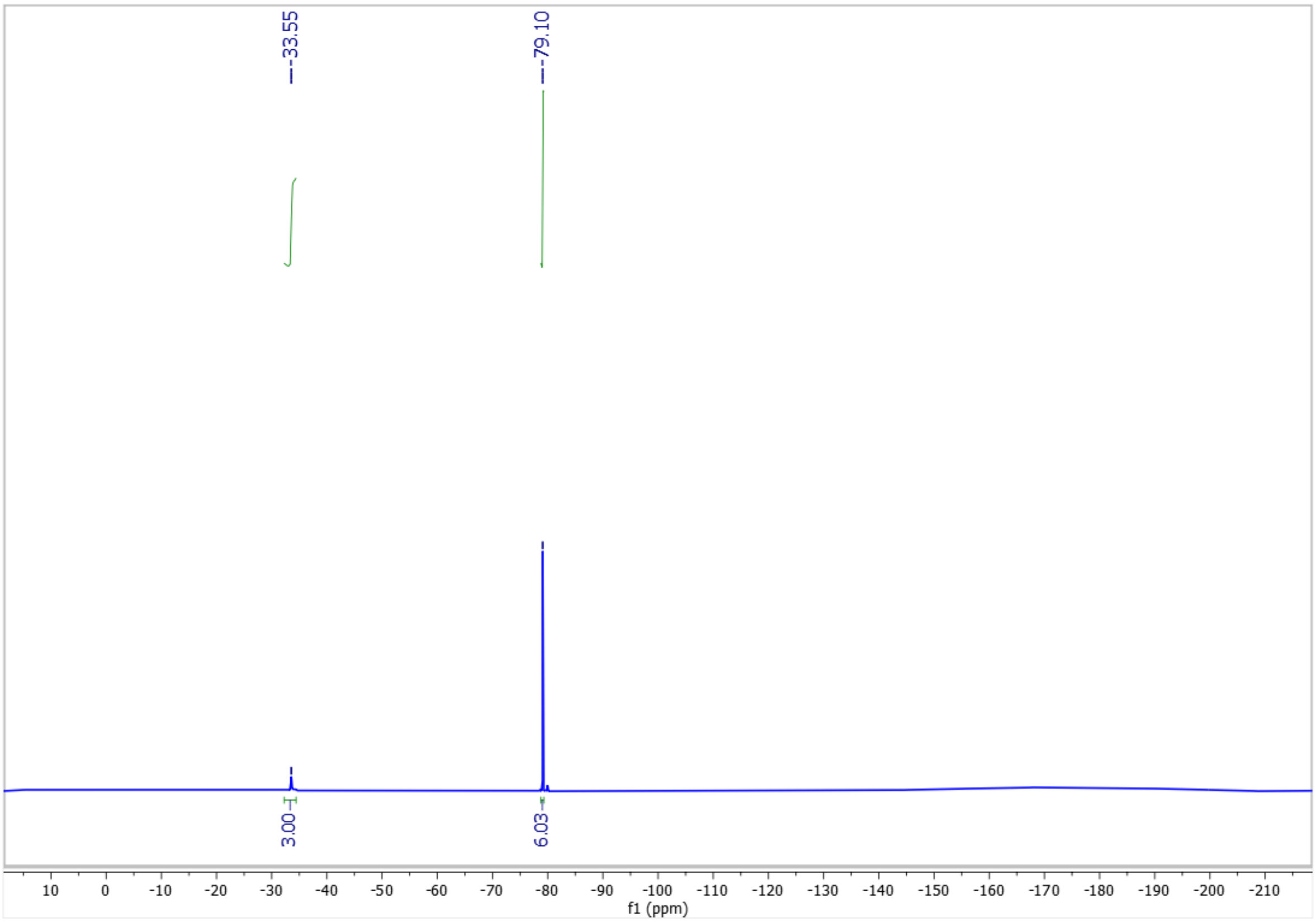

Figure S40. ^{19}F NMR spectrum of compound **6** in DCM-d_2.

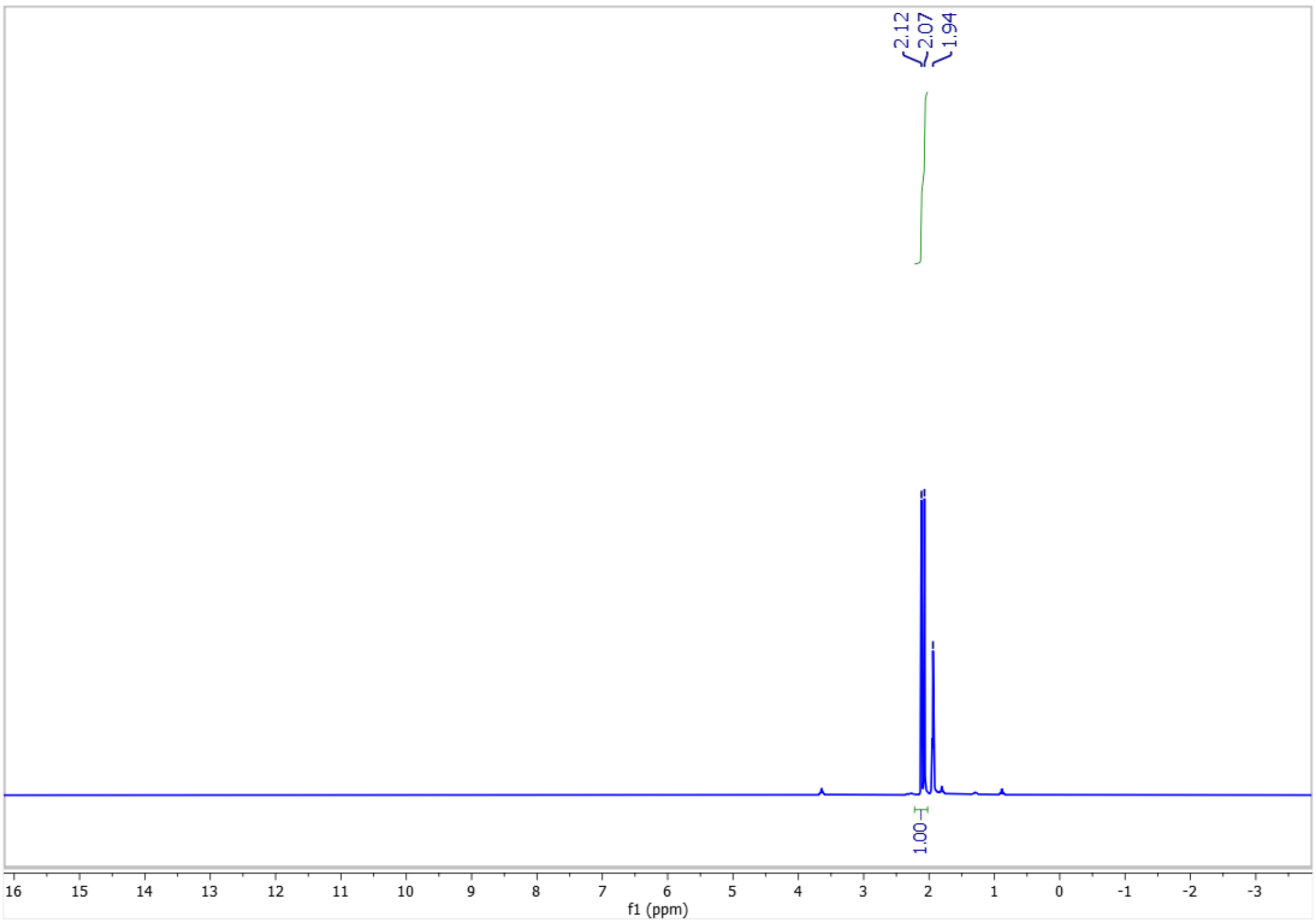

Figure S41. ^{1}H NMR spectrum of compound **6** in MeCN-d_3.

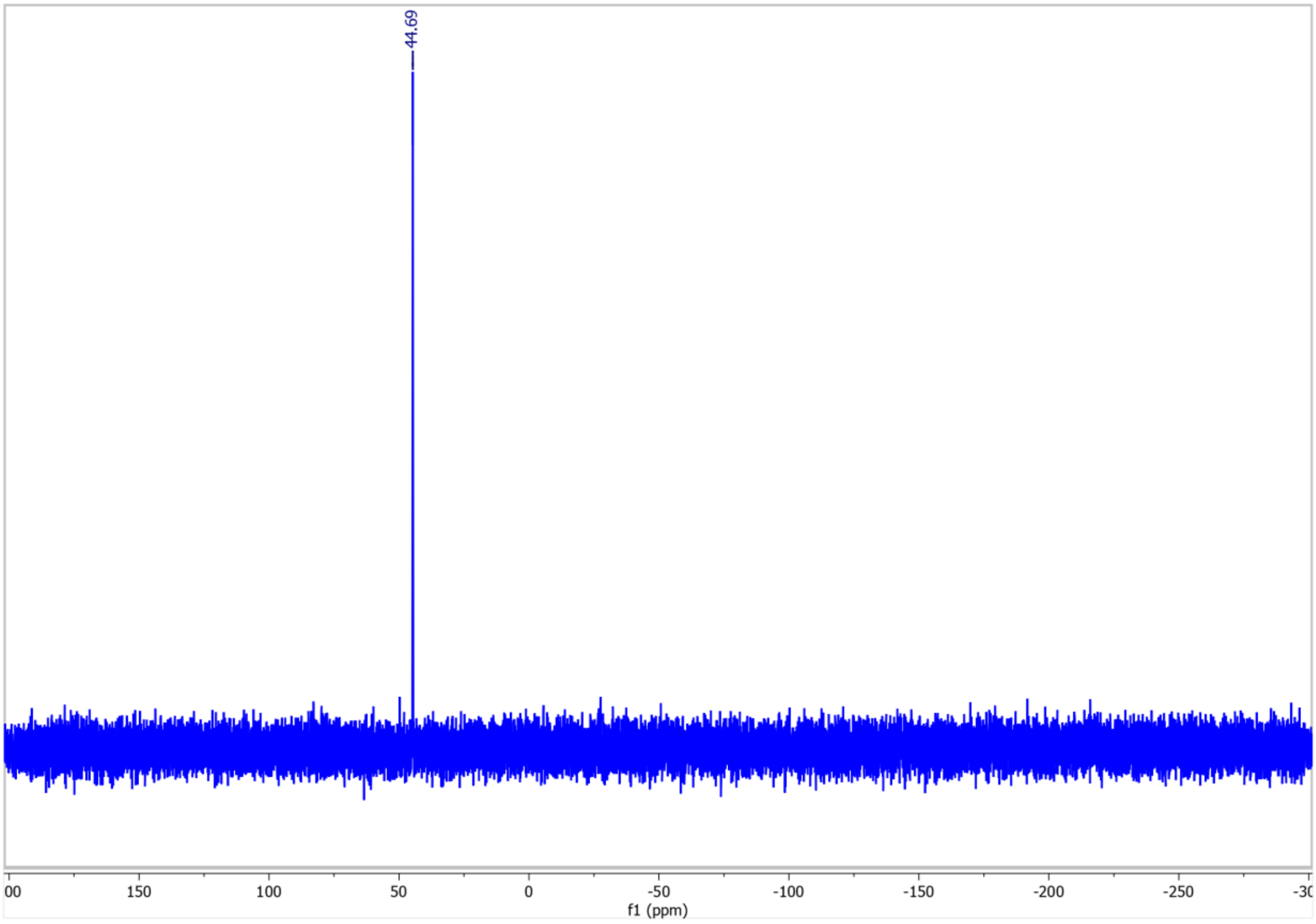

Figure S42. ^{31}P NMR spectrum of compound **6** in DCM-d_2.

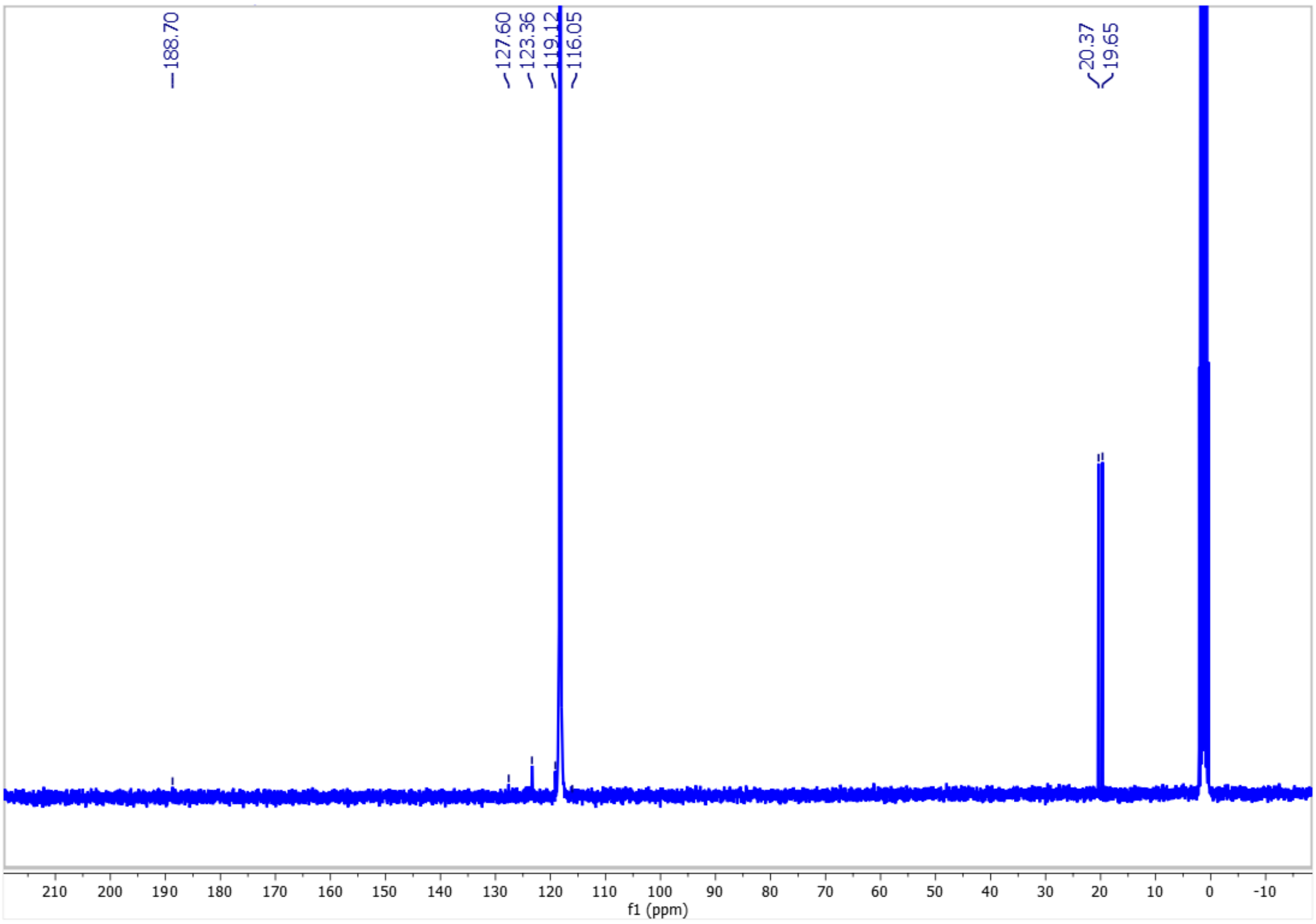

Figure S43. ^{13}C NMR spectrum of compound **6** in MeCN-d_3.

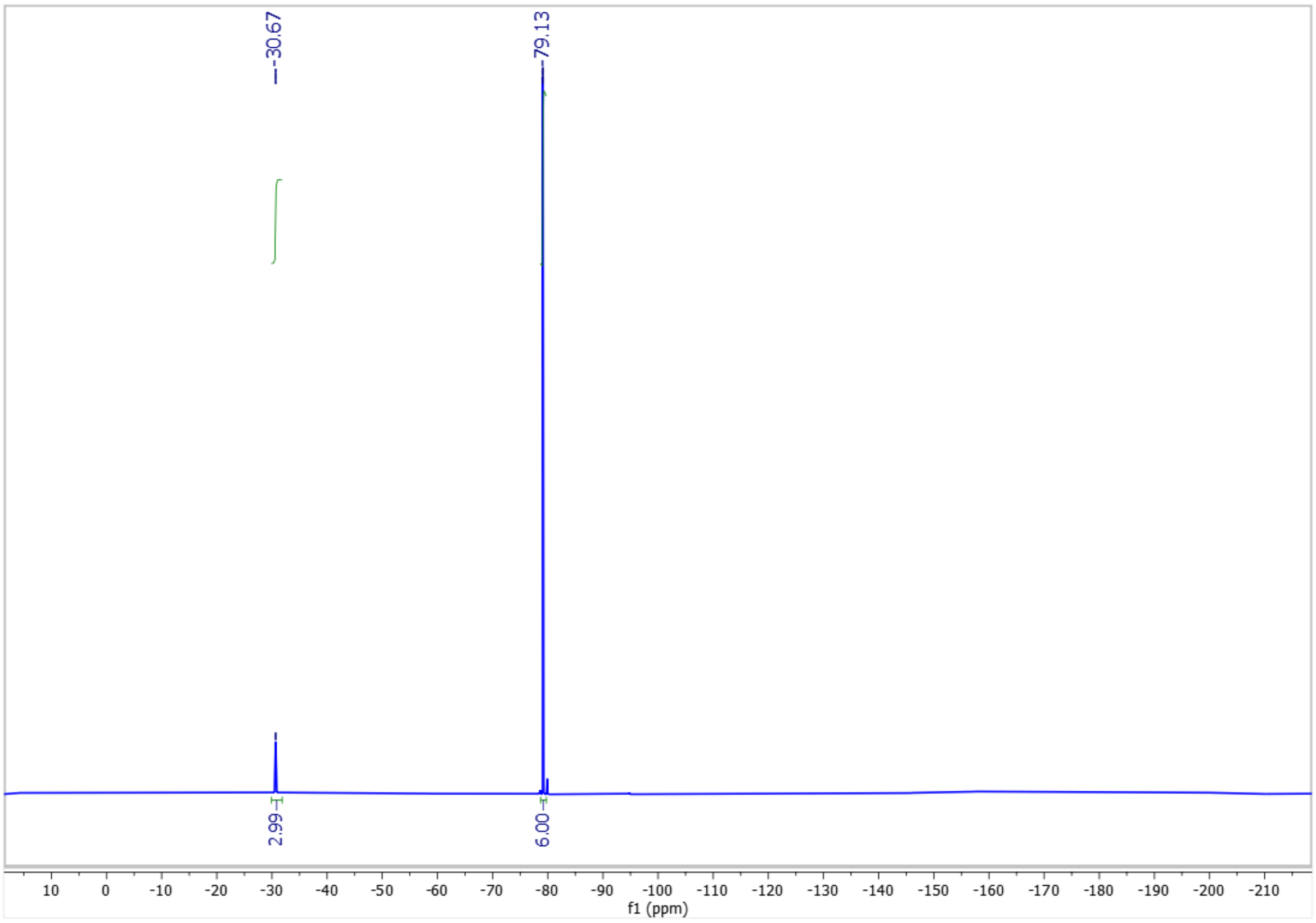

Figure S44. ^{19}F NMR spectrum of compound **7** in DCM-*d*$_2$.

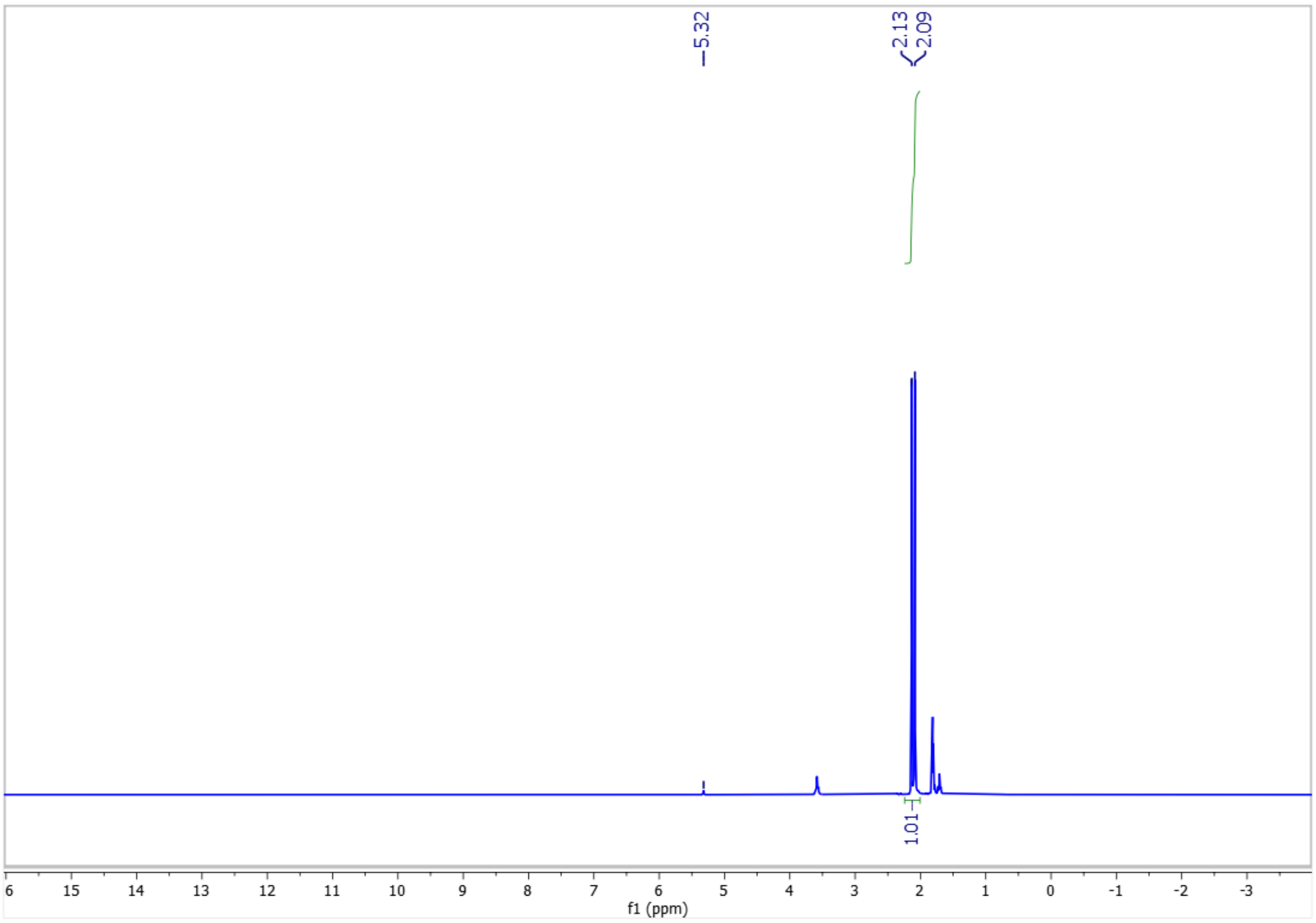

Figure S45. ^{1}H NMR spectrum of compound **7** in MeCN-*d*$_3$.

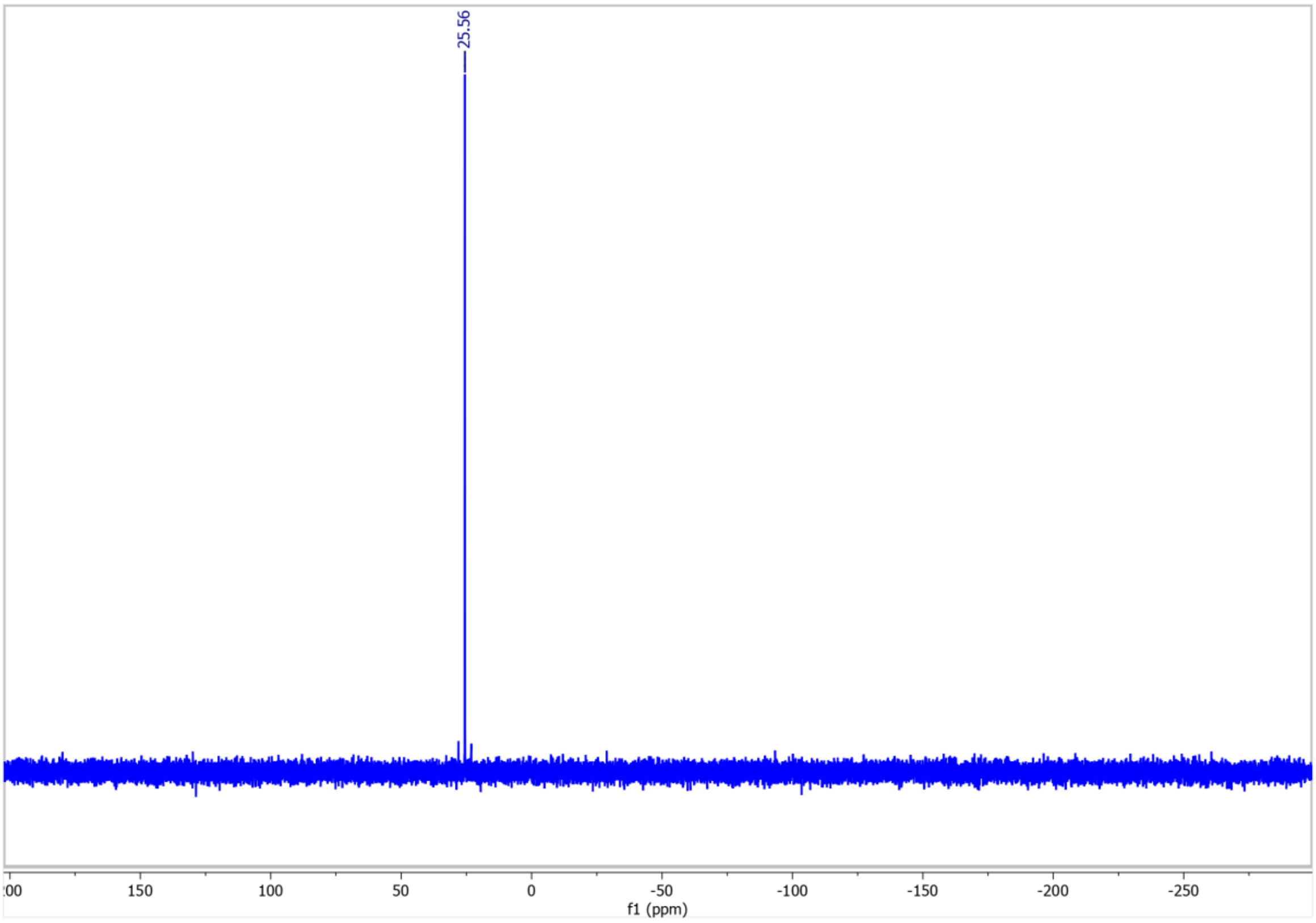

Figure S46. ^{31}P NMR spectrum of compound **7** in DCM-d_2.

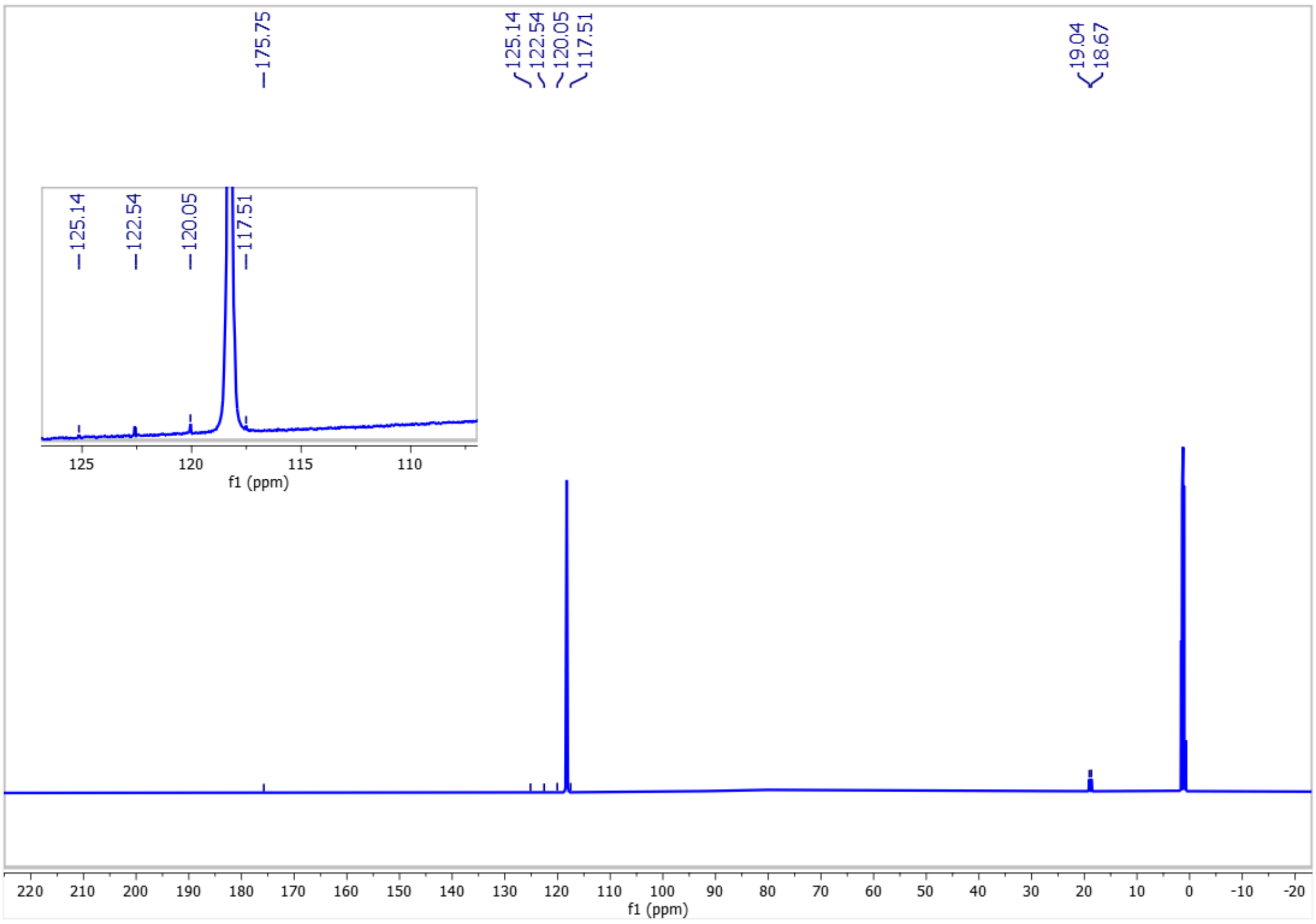

Figure S47. ^{13}C NMR spectrum of compound **7** in MeCN-d_3.

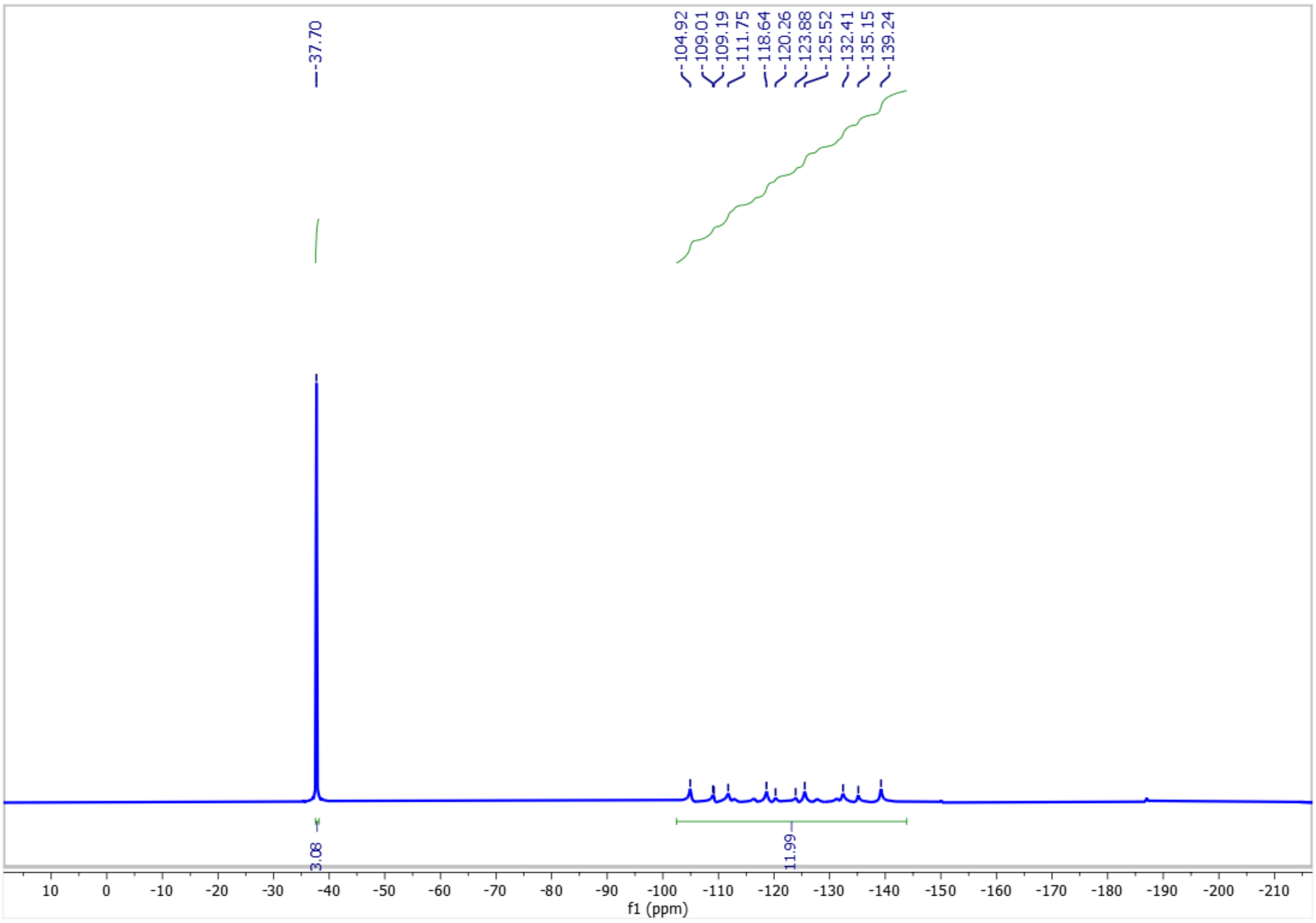

Figure S48. ^{19}F NMR spectrum of compound **8** in MeCN-*d*$_3$.

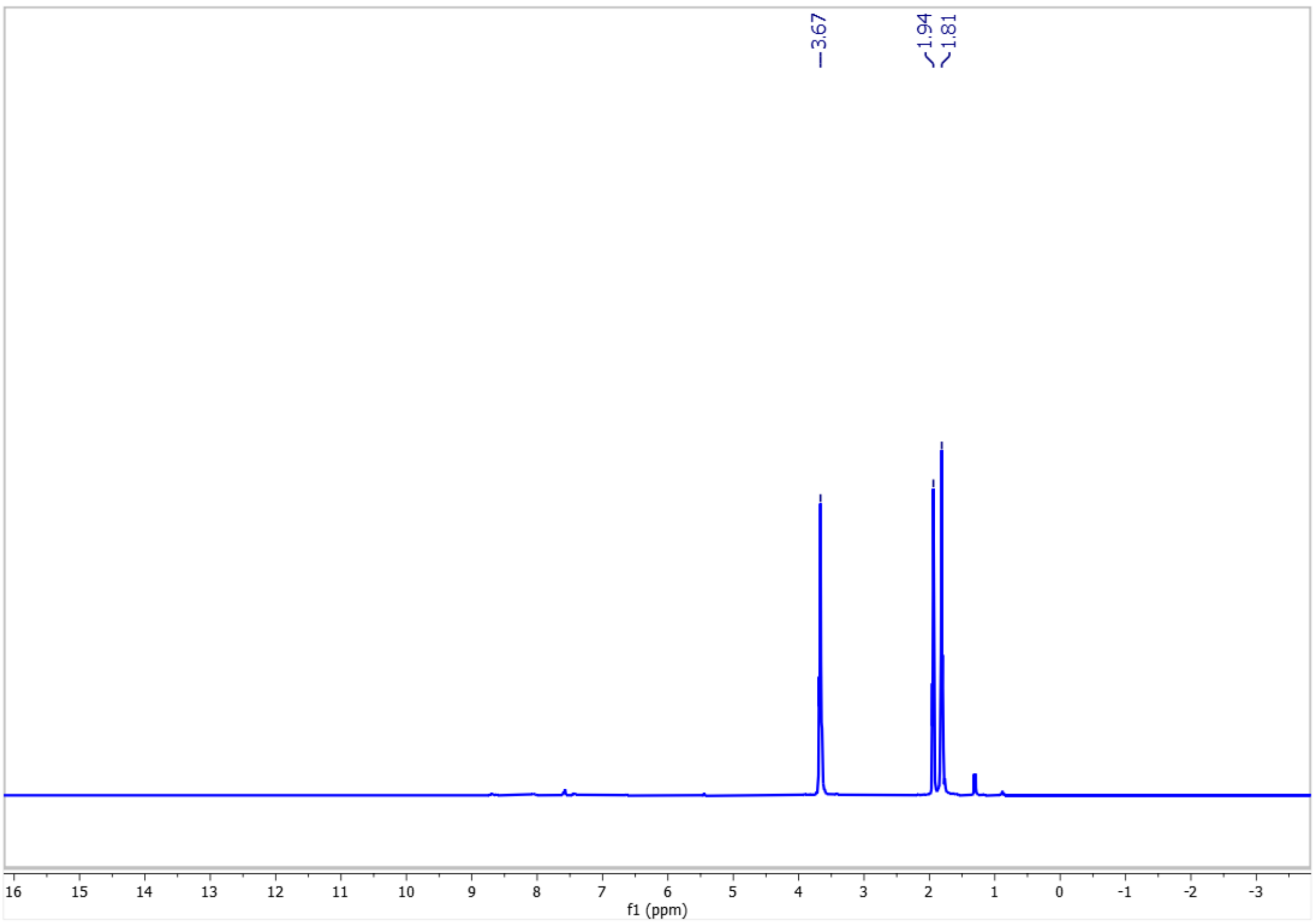

Figure S49. ^{1}H NMR spectrum of compound **8** in MeCN-*d*$_3$.

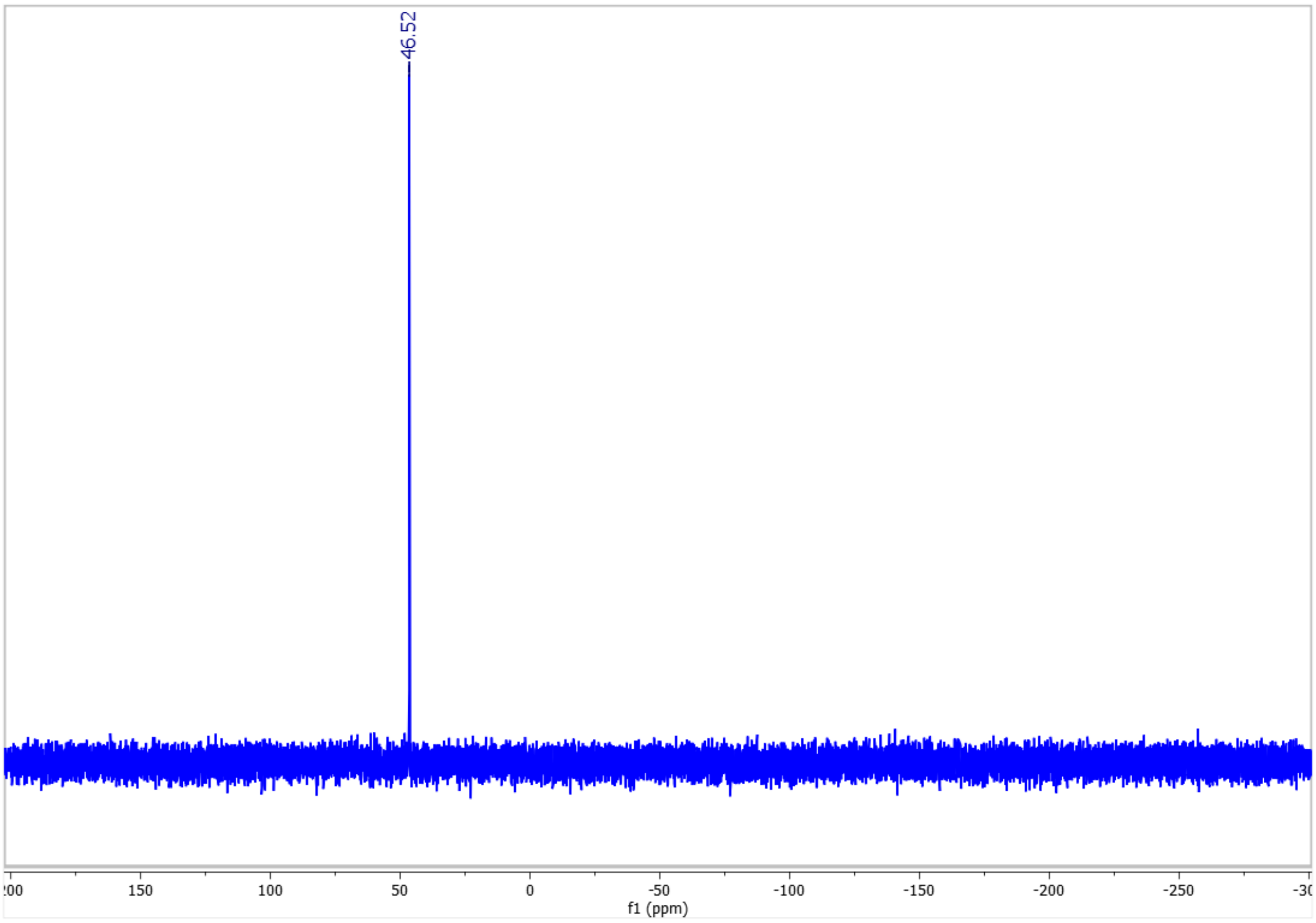

Figure S50. ^{31}P NMR spectrum of compound **8** in MeCN-d_3.

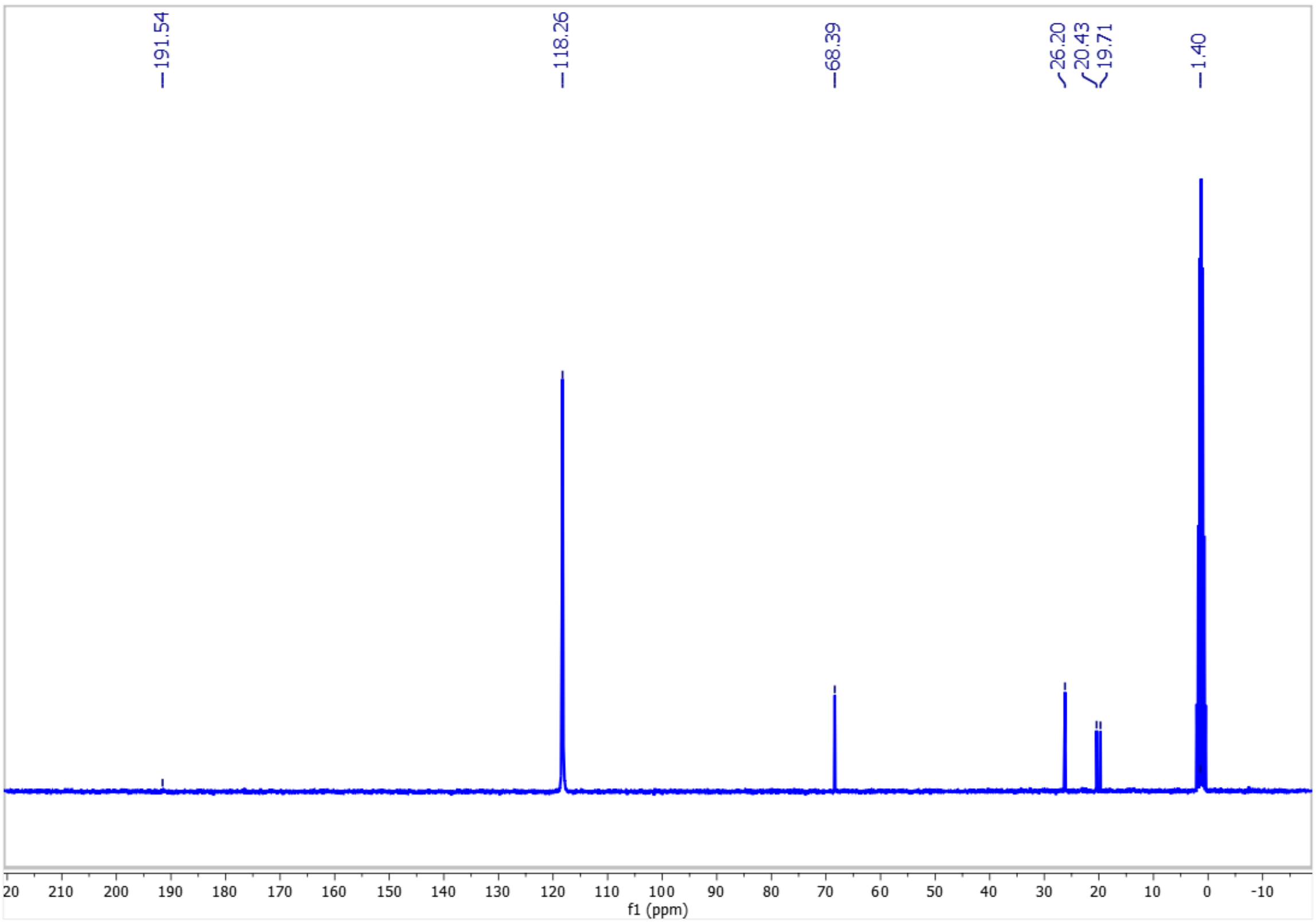

Figure S51. ^{13}C NMR spectrum of compound **8** in MeCN-d_3.

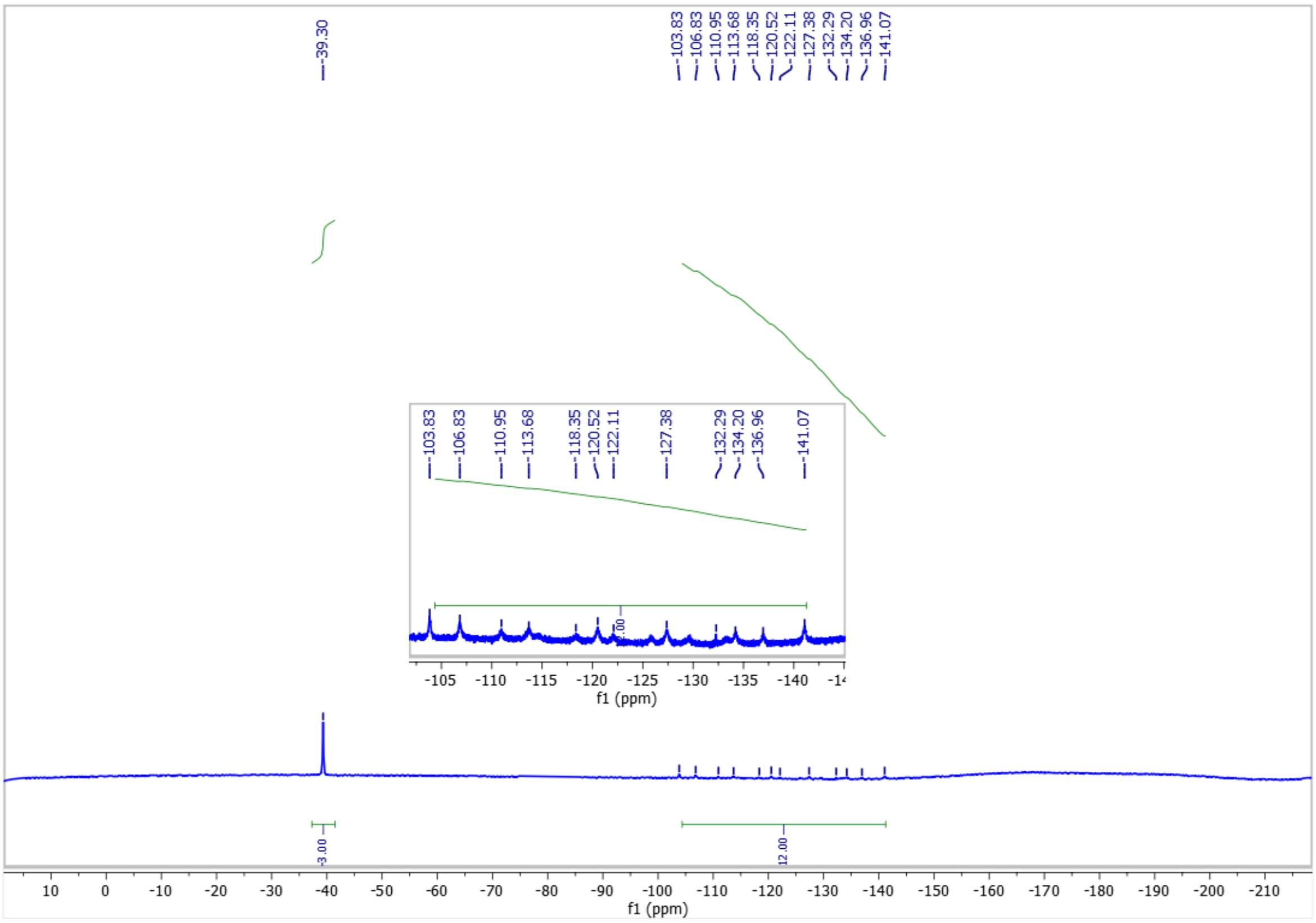

Figure S52. ^{19}F NMR spectrum of compound **9** in MeCN-d_3.

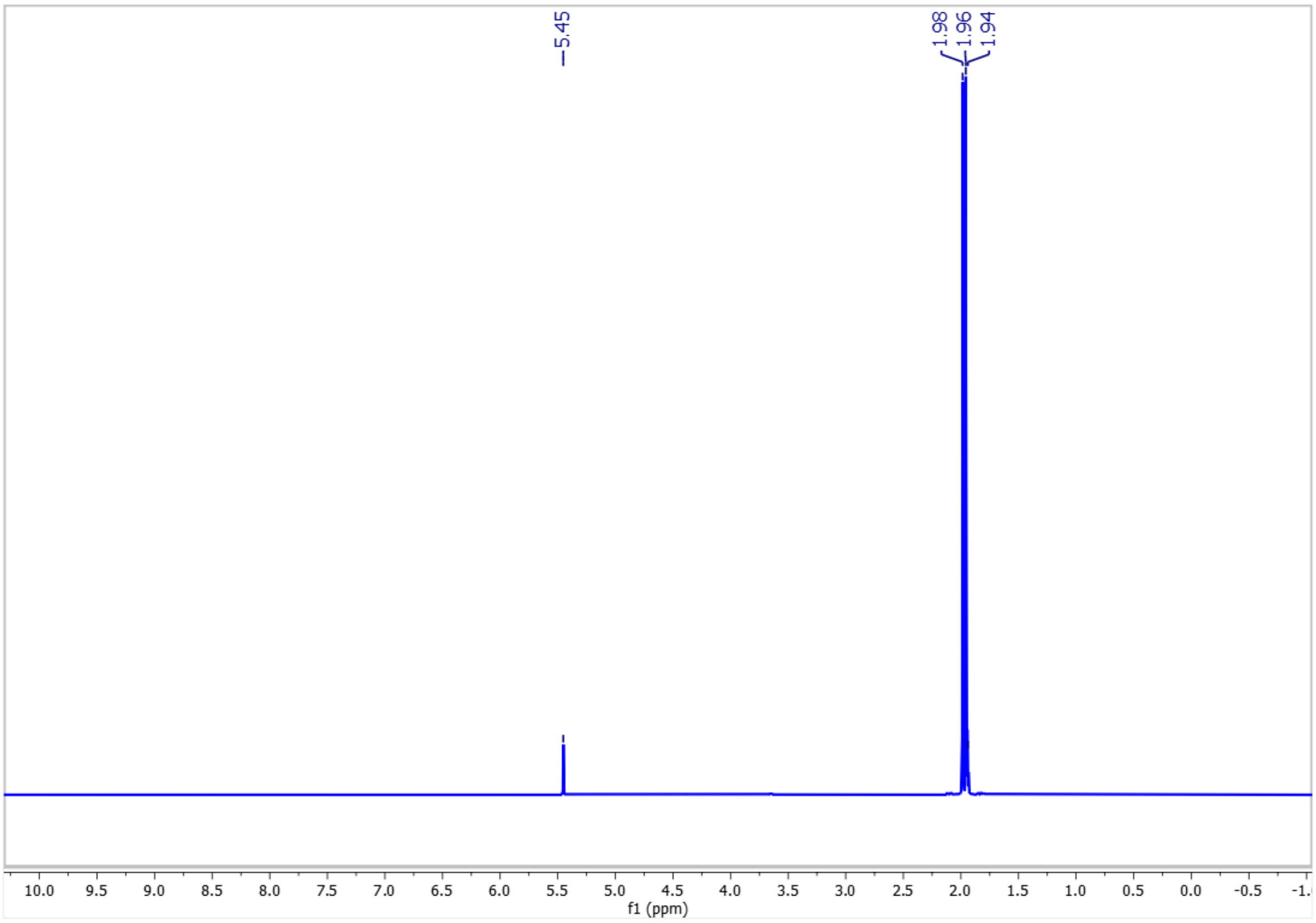

Figure S53. ^{1}H NMR spectrum of compound **9** in MeCN-d_3.

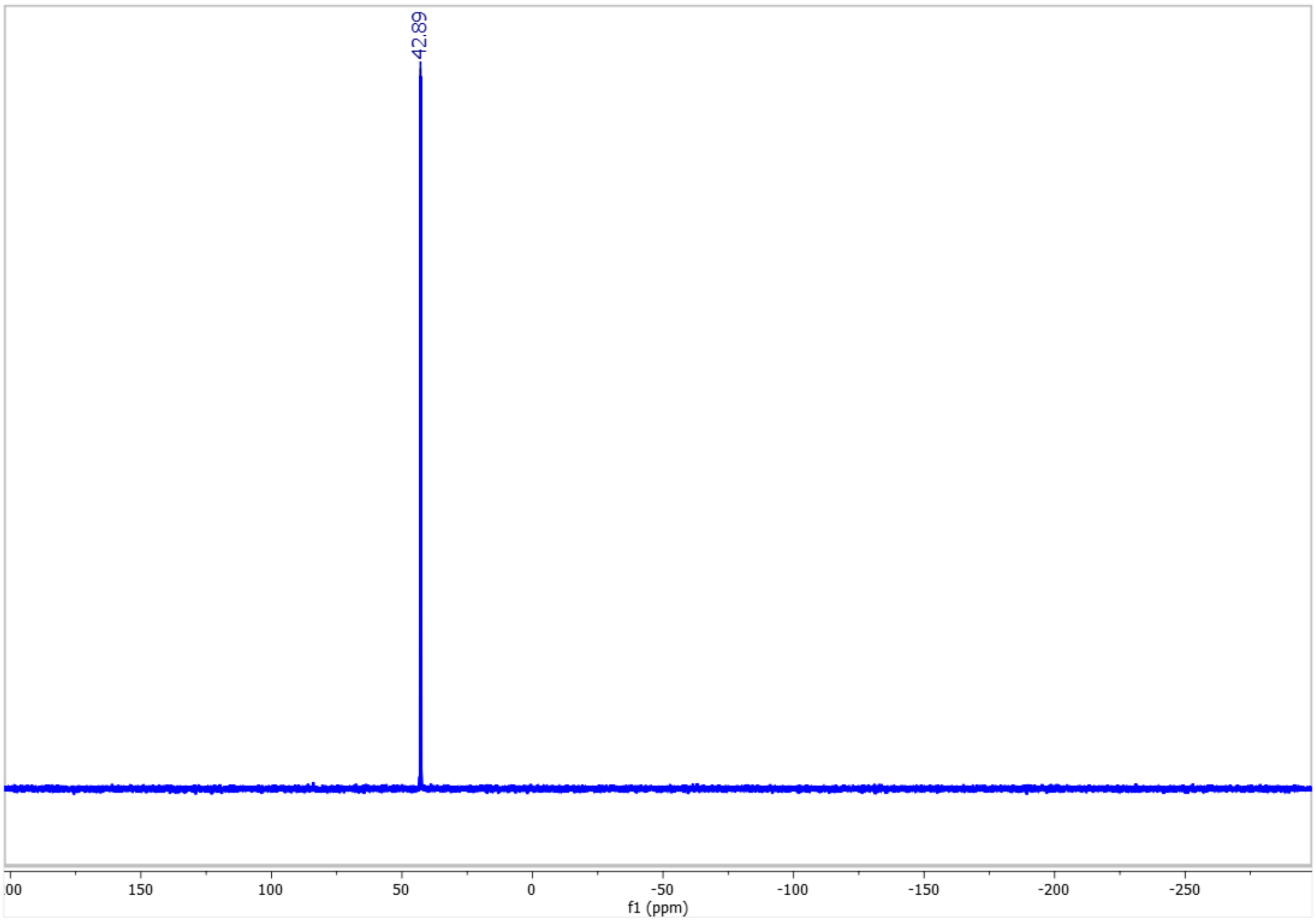

Figure S54. ^{31}P NMR spectrum of compound **9** in MeCN-d_3.

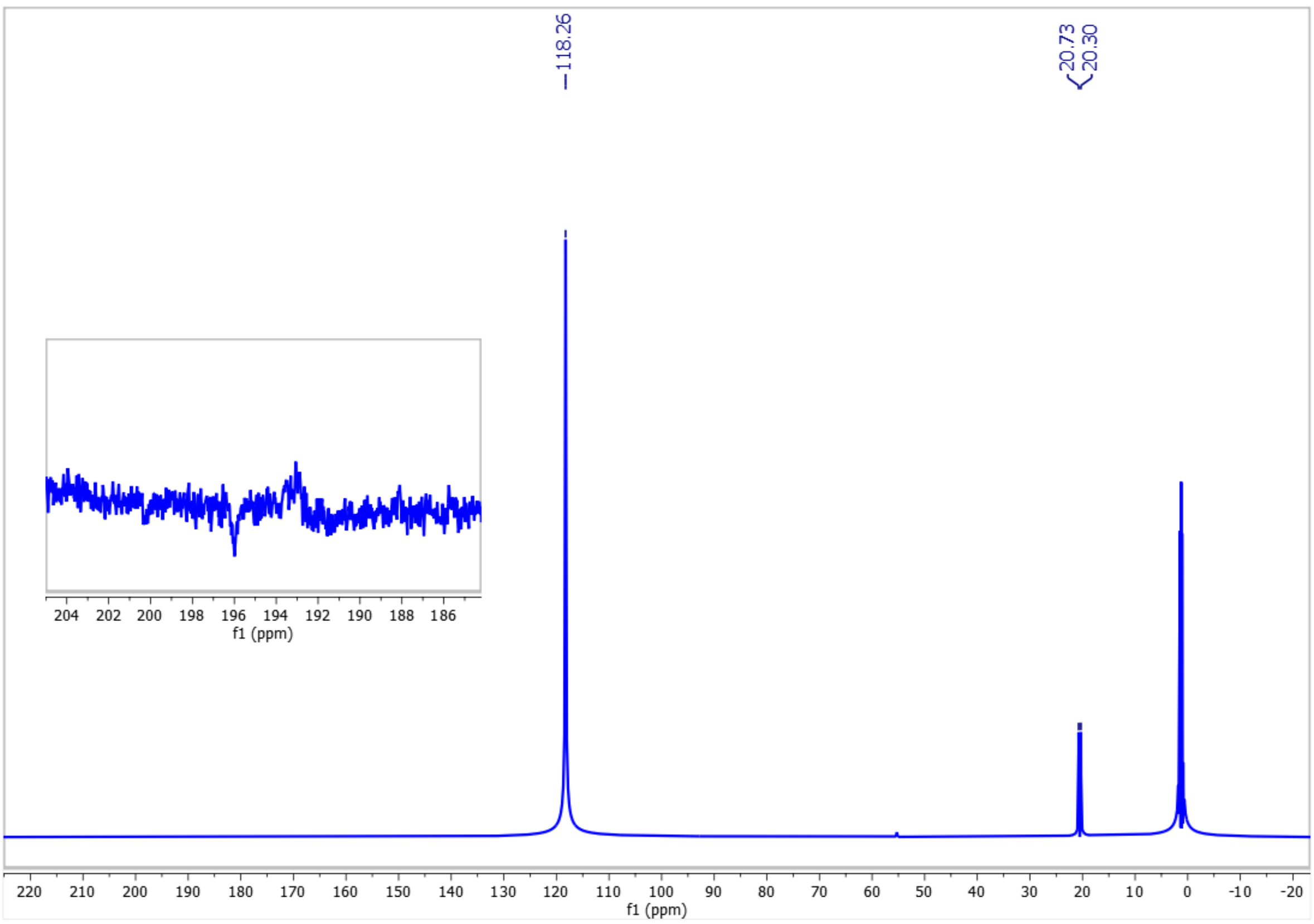

Figure S55. ^{13}C NMR spectrum of compound **9** in MeCN-d_3.

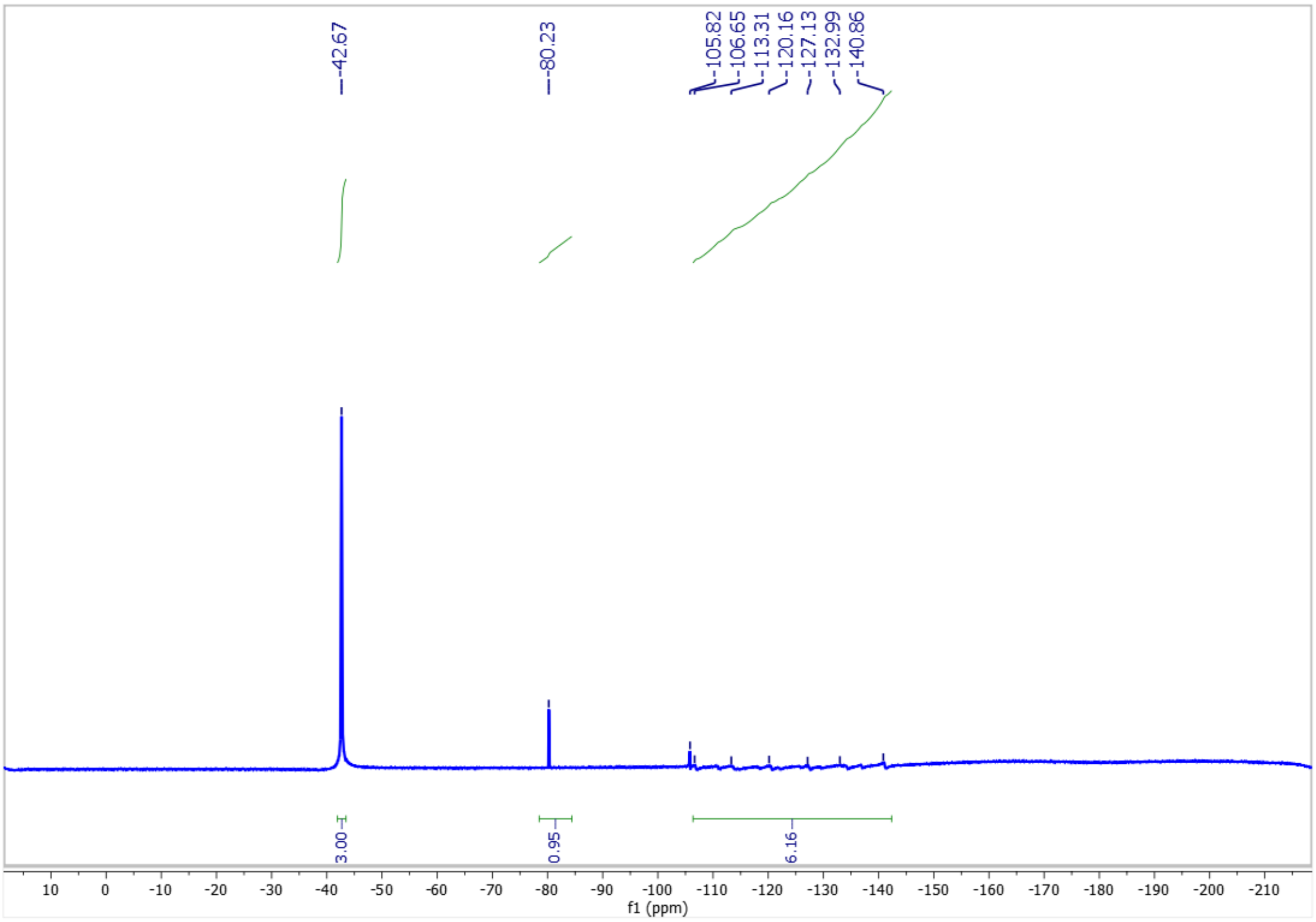

Figure S56. ^{19}F NMR spectrum of compound **10** in MeCN-d_3.

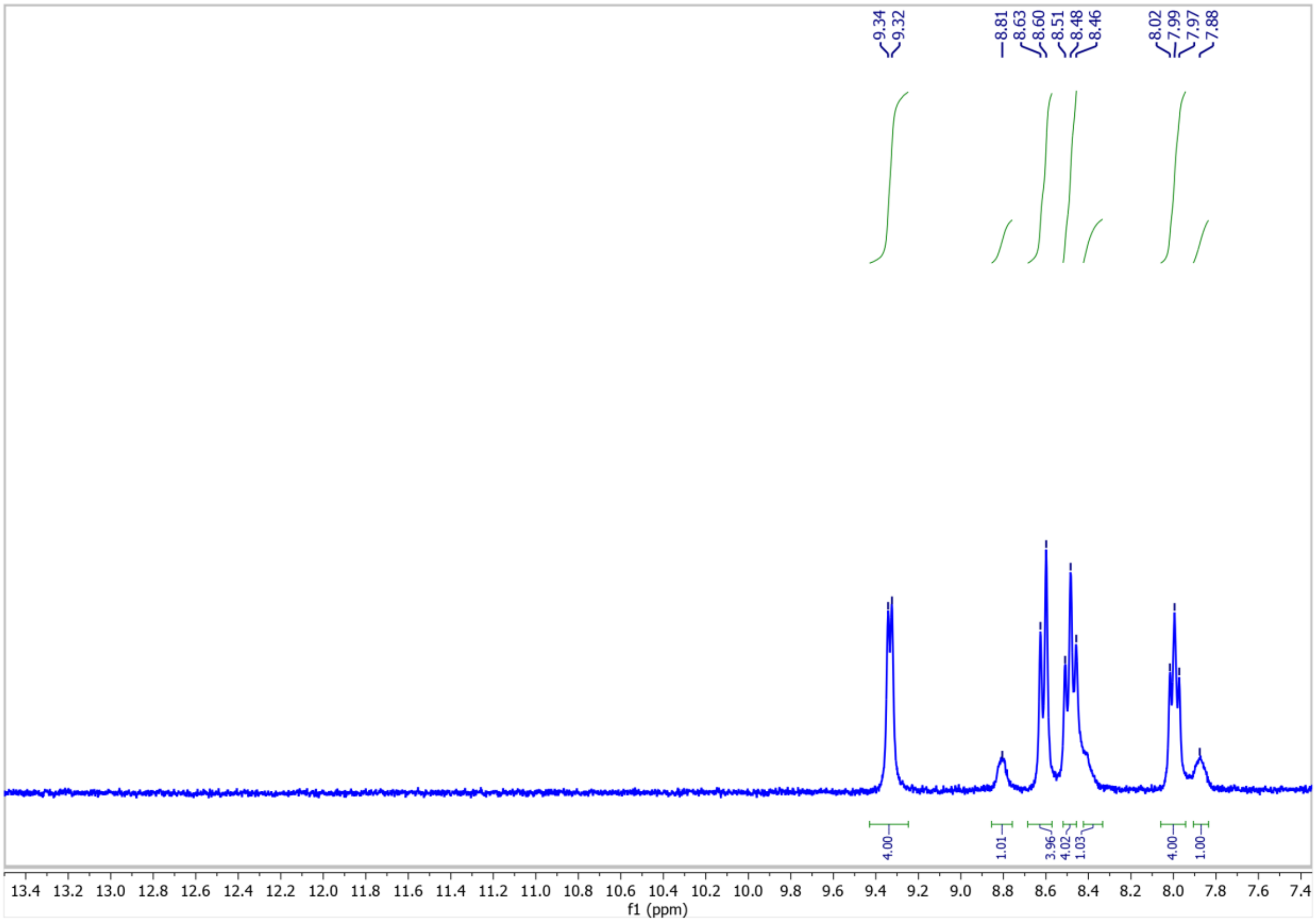

Figure S57. ^{1}H NMR spectrum of compound **10-py** in MeCN-d_3.

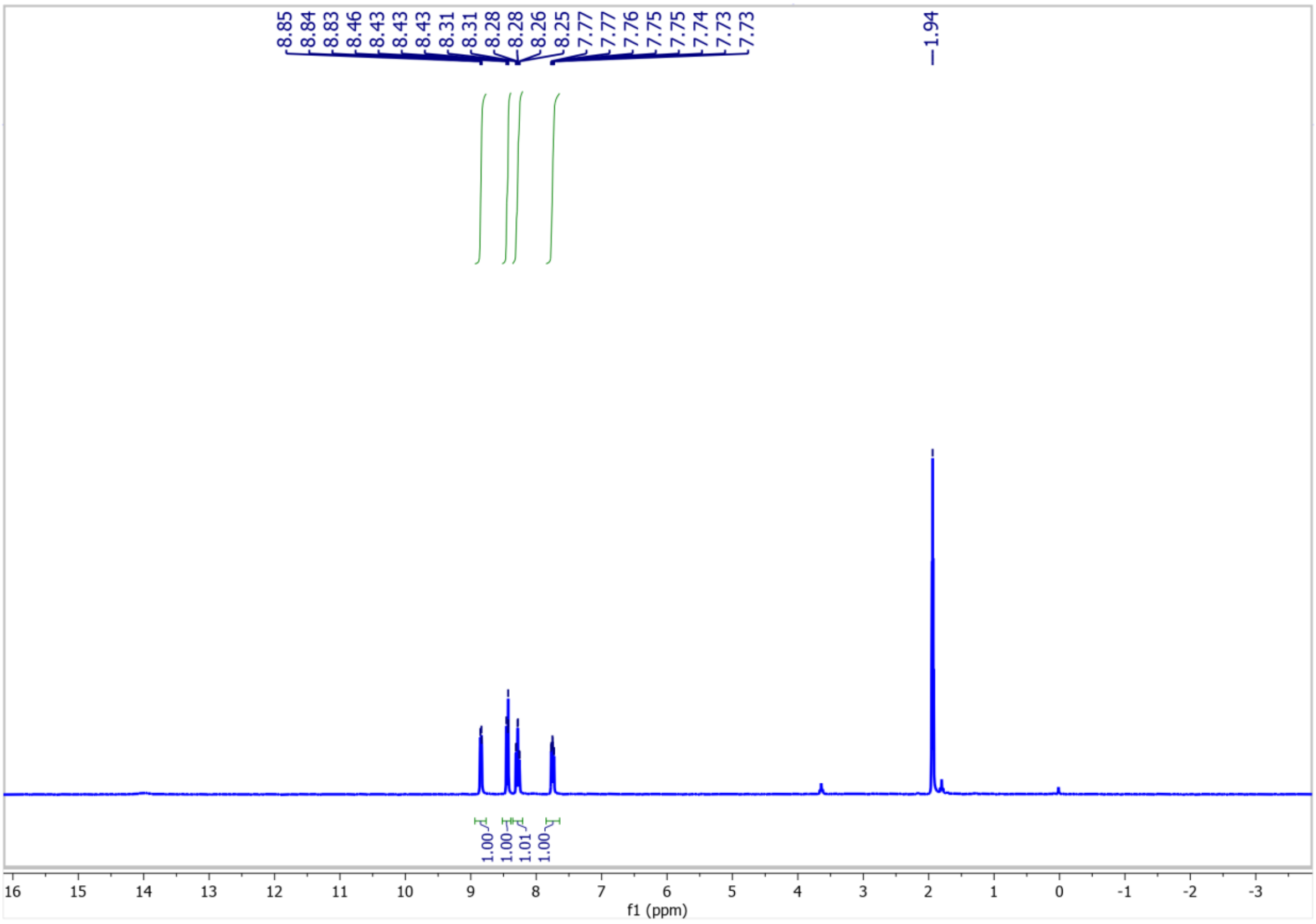

Figure S58. ^{1}H NMR spectrum of compound **10** in MeCN-*d₃*.

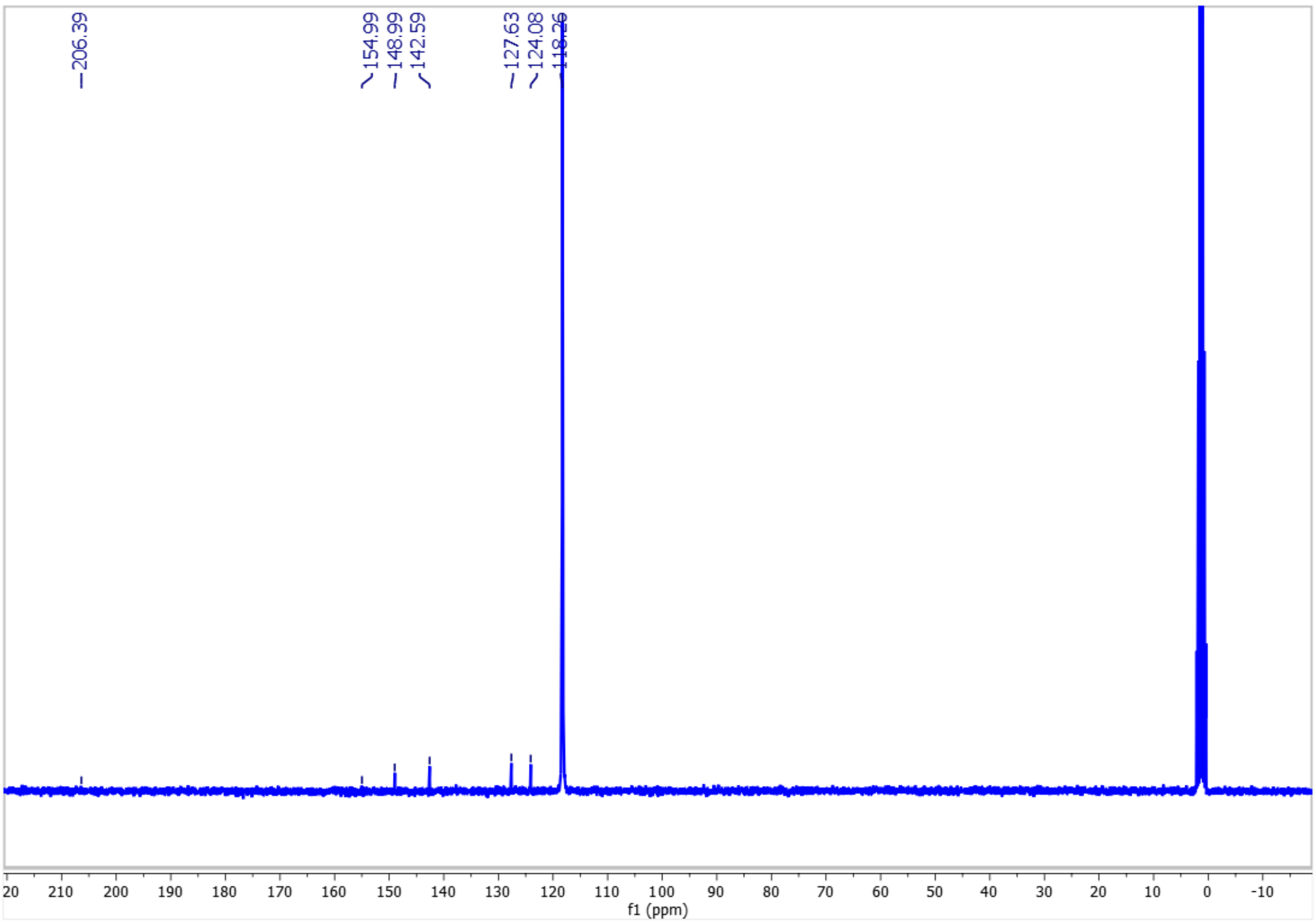

Figure S59. ^{13}C NMR spectrum of compound **10** in MeCN-*d₃*.

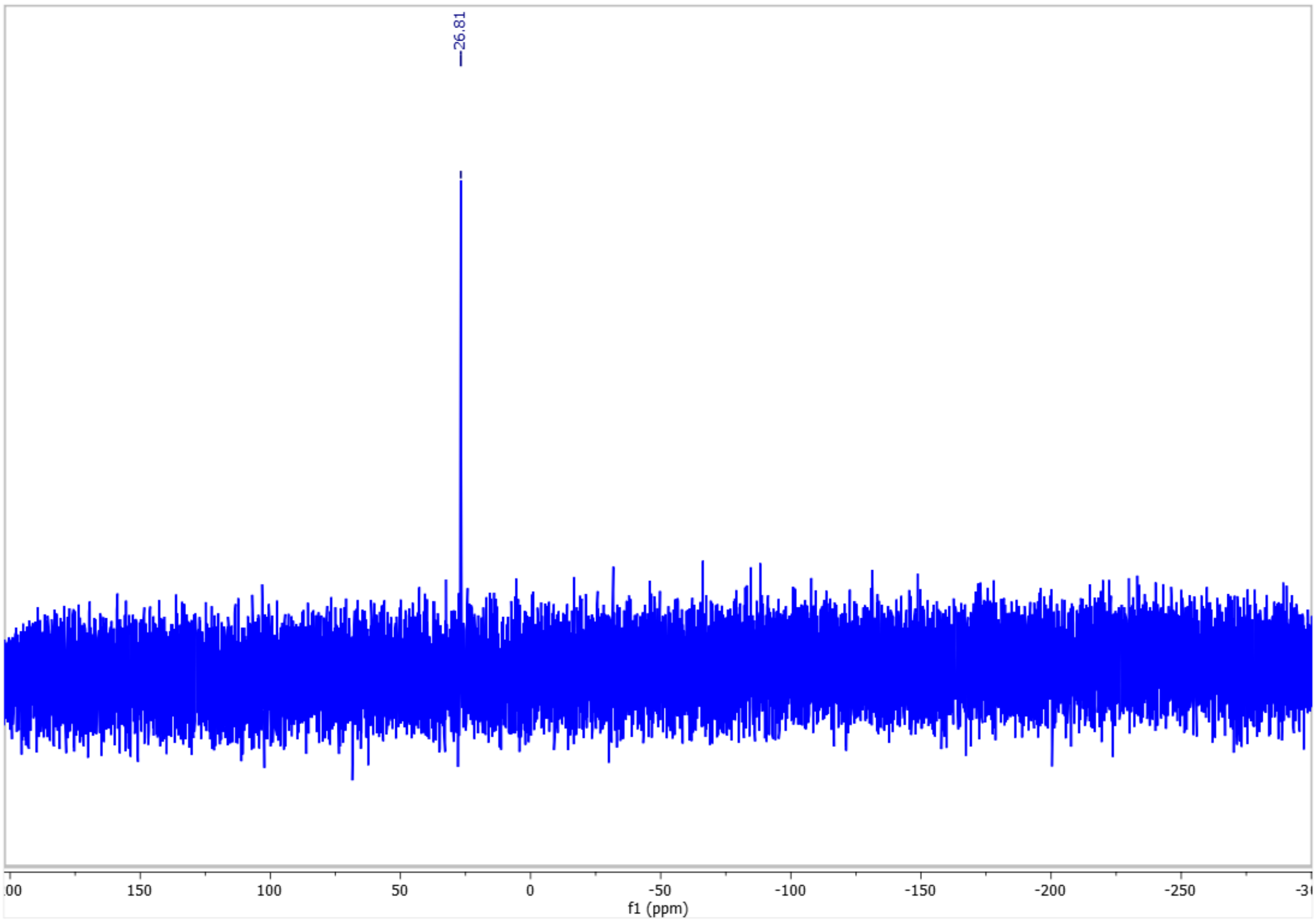

Figure S60. ^{31}P NMR spectrum of compound **11** in MeCN-d_3.

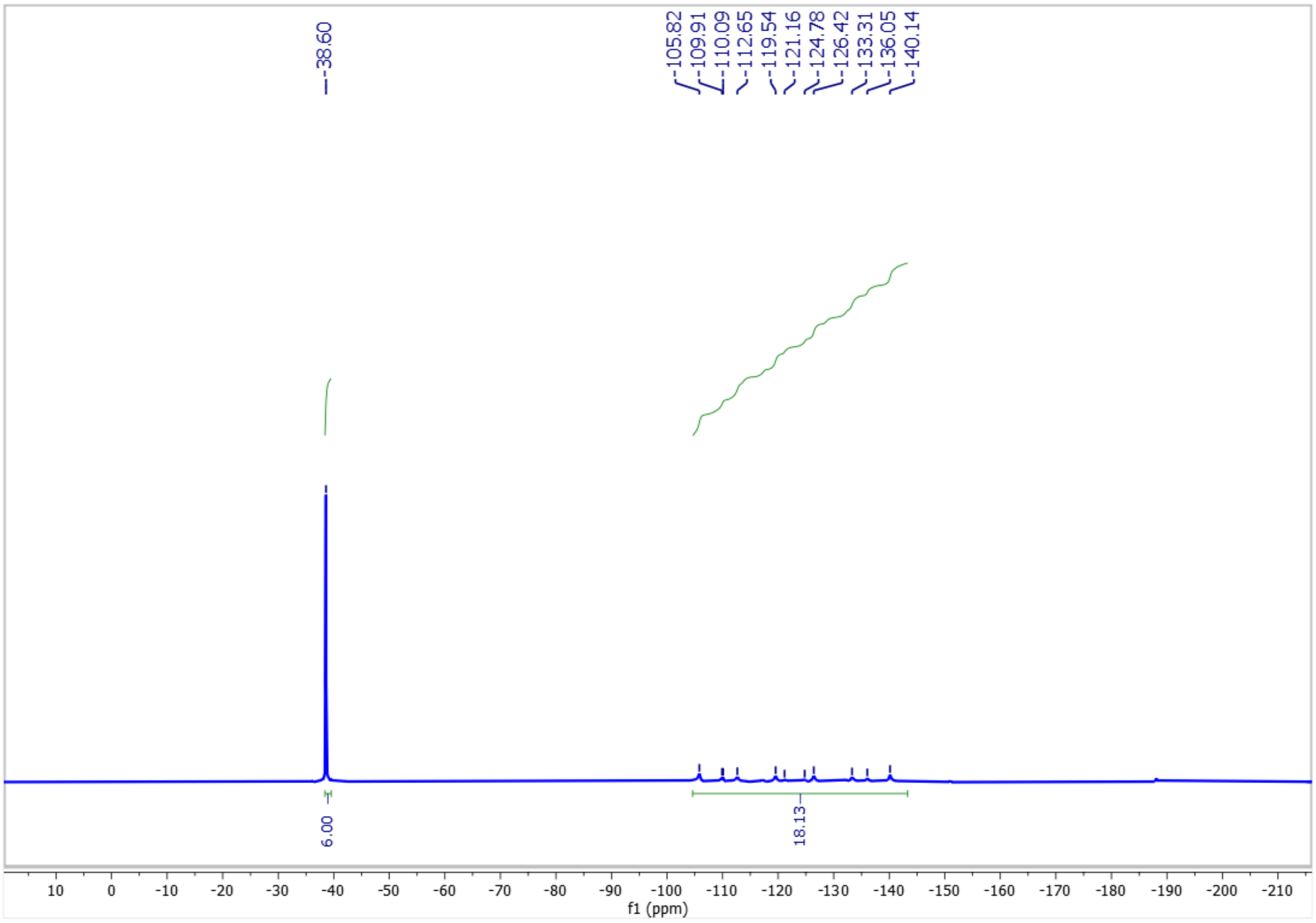

Figure S61. ^{19}F NMR spectrum of compound **11** in MeCN-d_3.

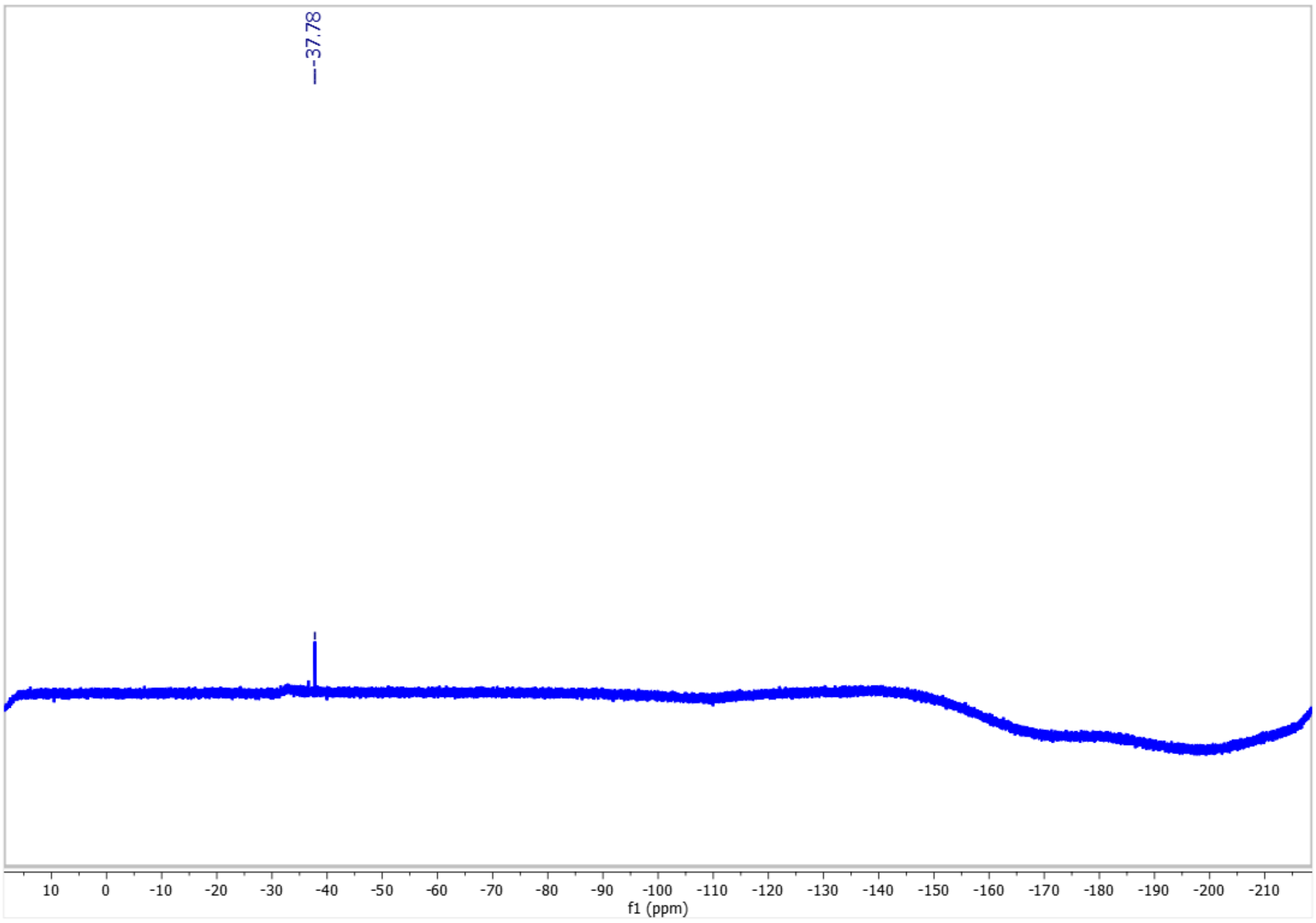

Figure S62. ^{19}F NMR spectrum of compound **11** in DCM-d_2.

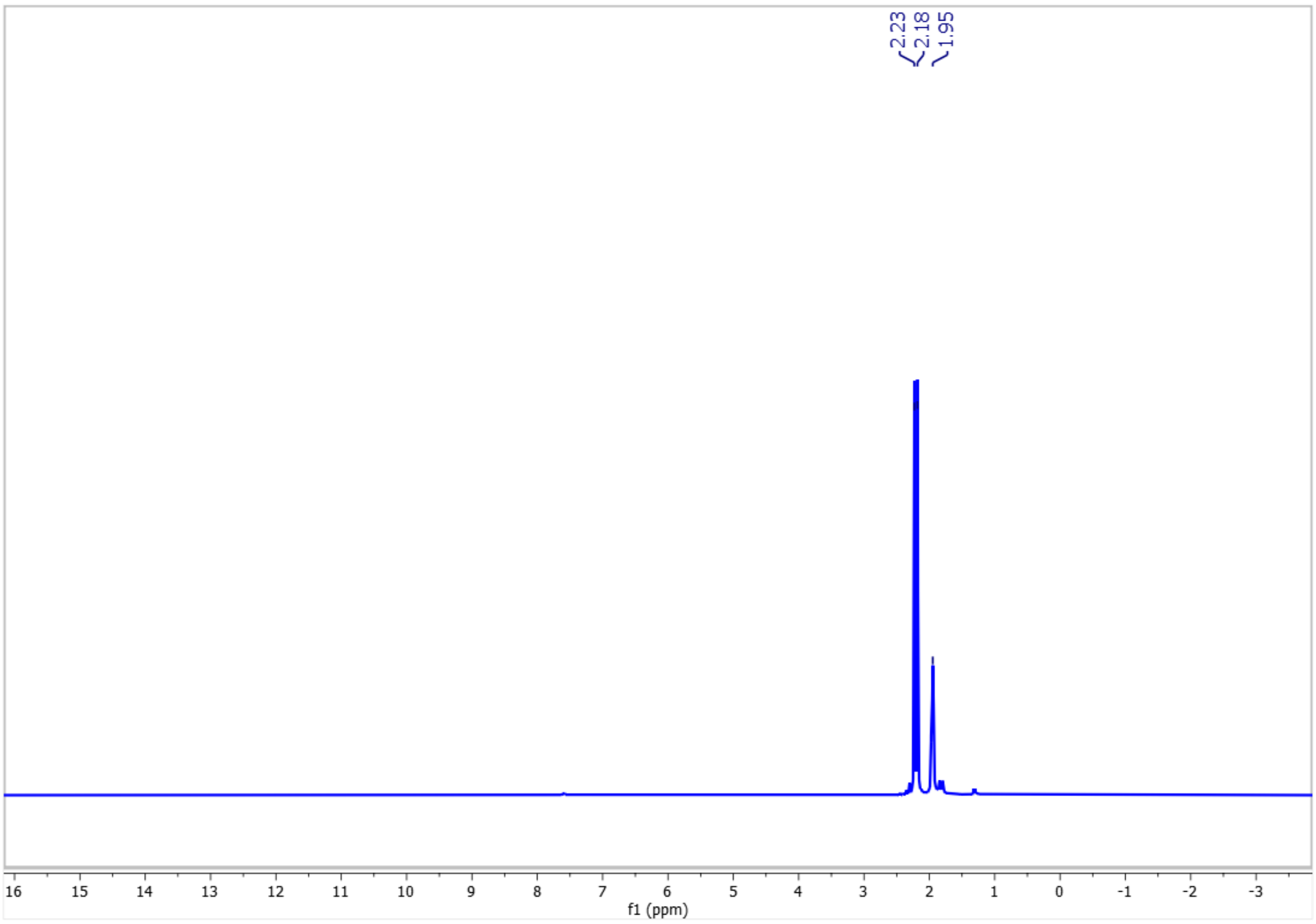

Figure S63. ^{1}H NMR spectrum of **11** in MeCN-d_3.

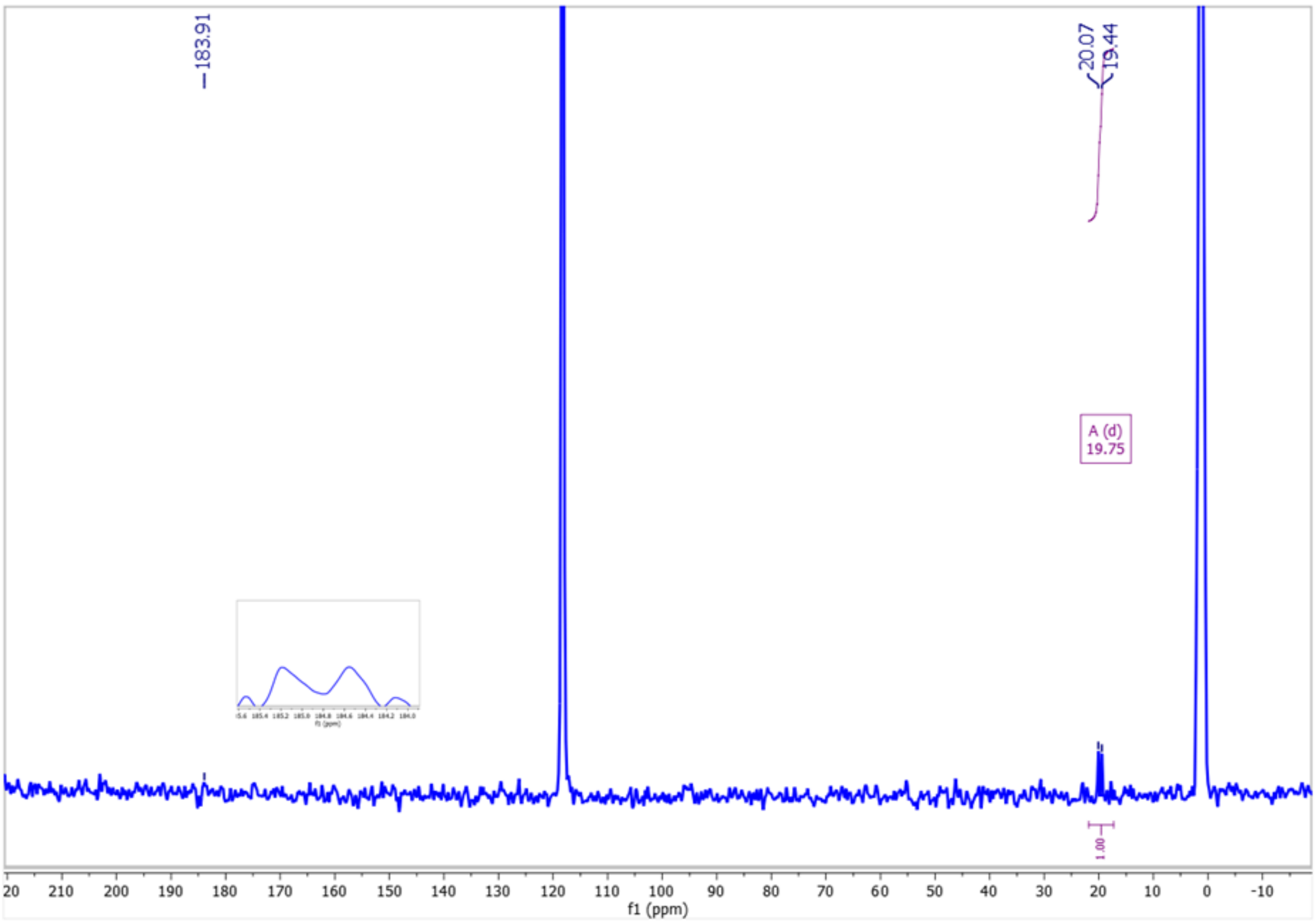

Figure S64. ^{13}C NMR spectrum of **11** in MeCN-d_3.

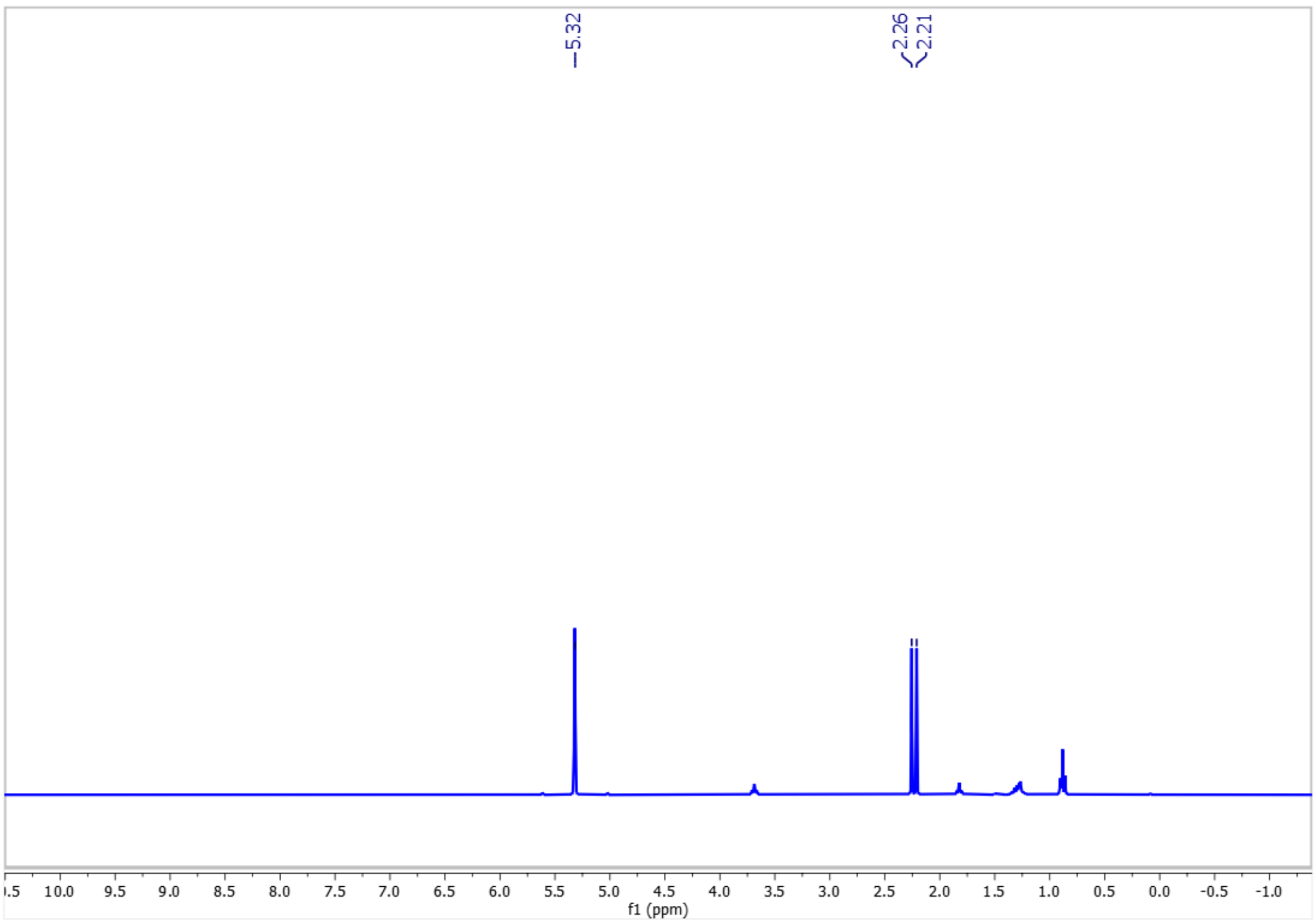

Figure S65. ^{1}H NMR spectrum of compound **12** in DCM-d_2.

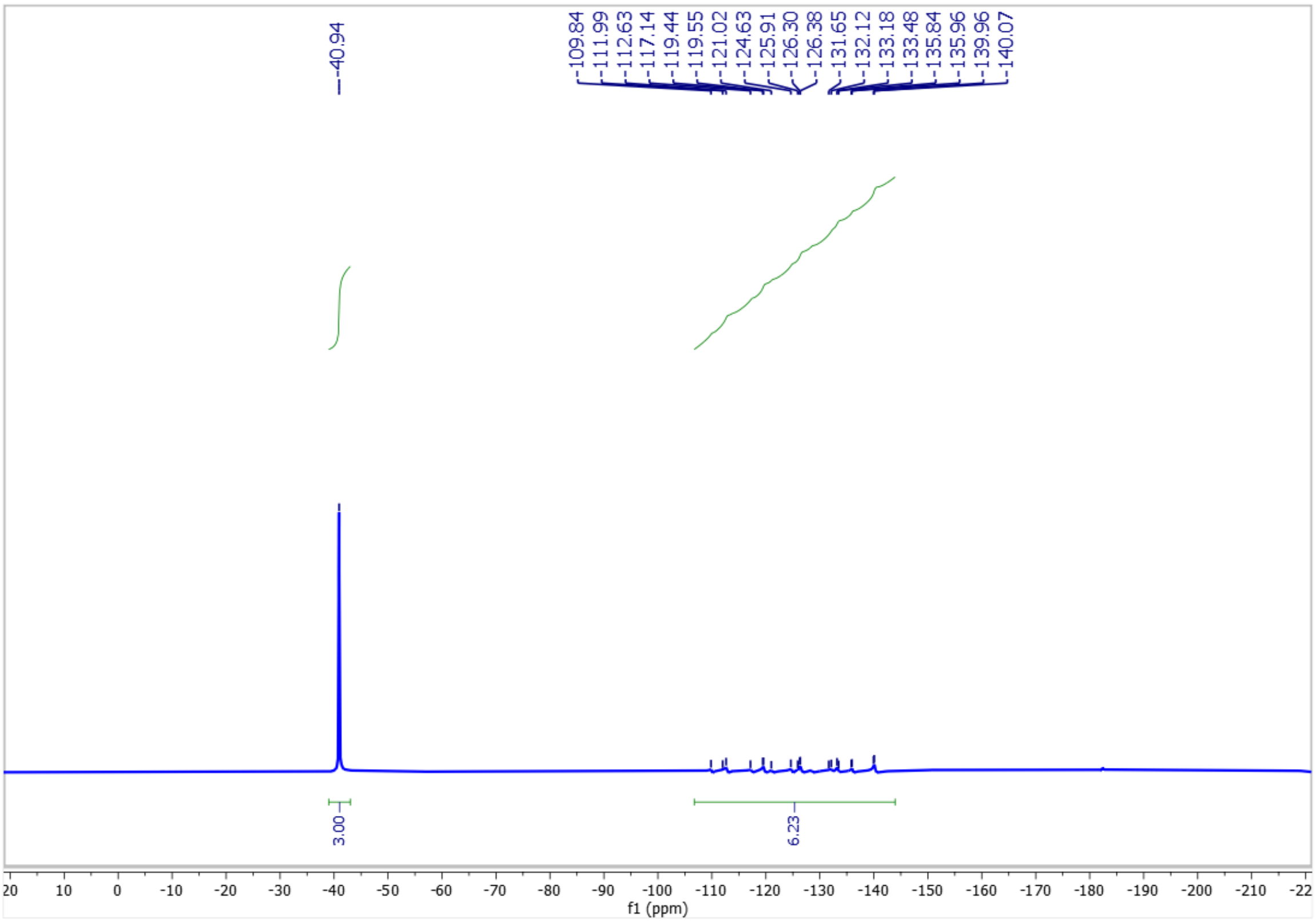

Figure S66. ^{19}F NMR spectrum of compound **12** in MeCN-d_3.

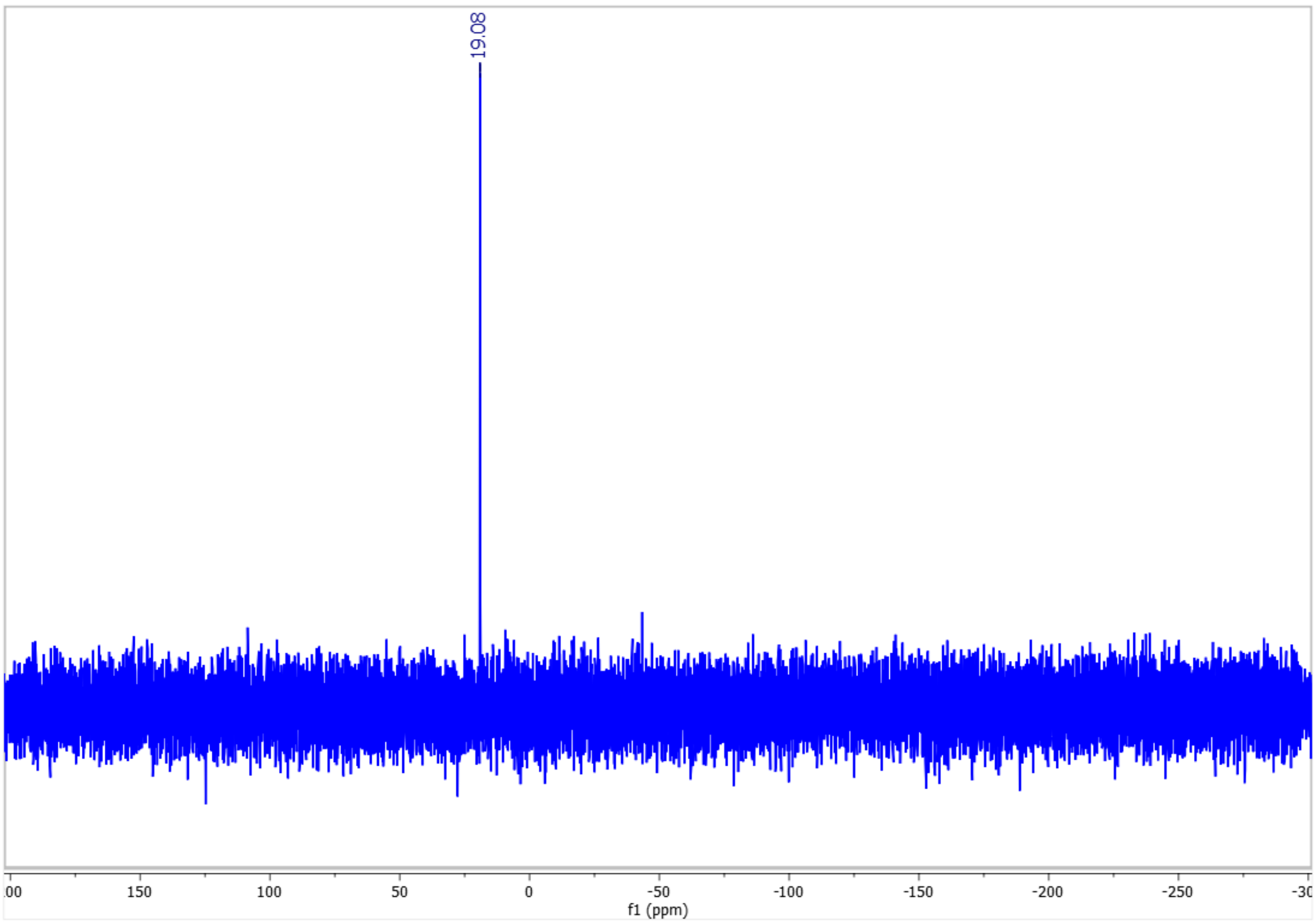

Figure S67. ^{31}P NMR spectrum of compound **12** in DCM-d_2.

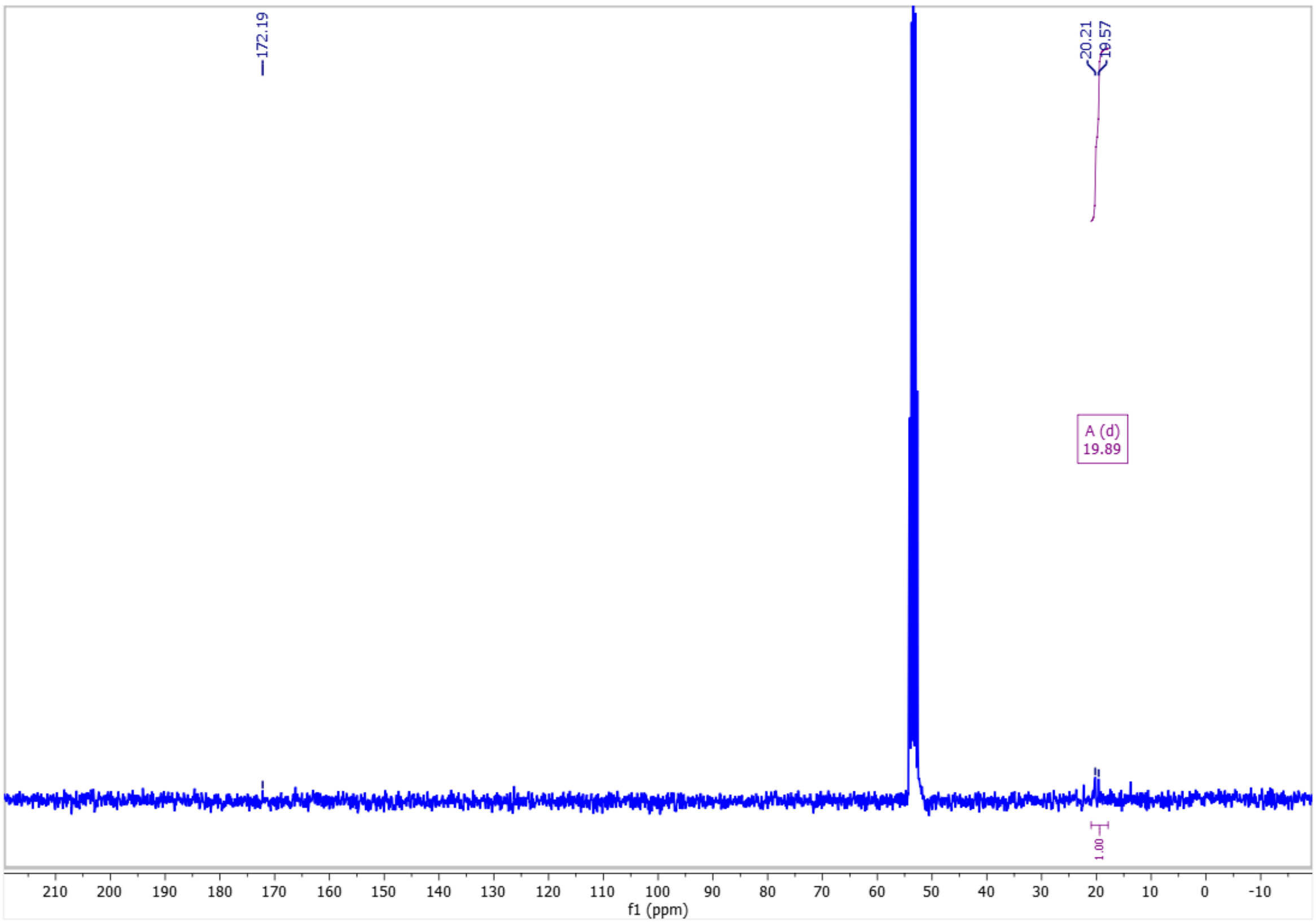

Figure S68. ^{13}C NMR spectrum of compound **12** in DCM-d_2.

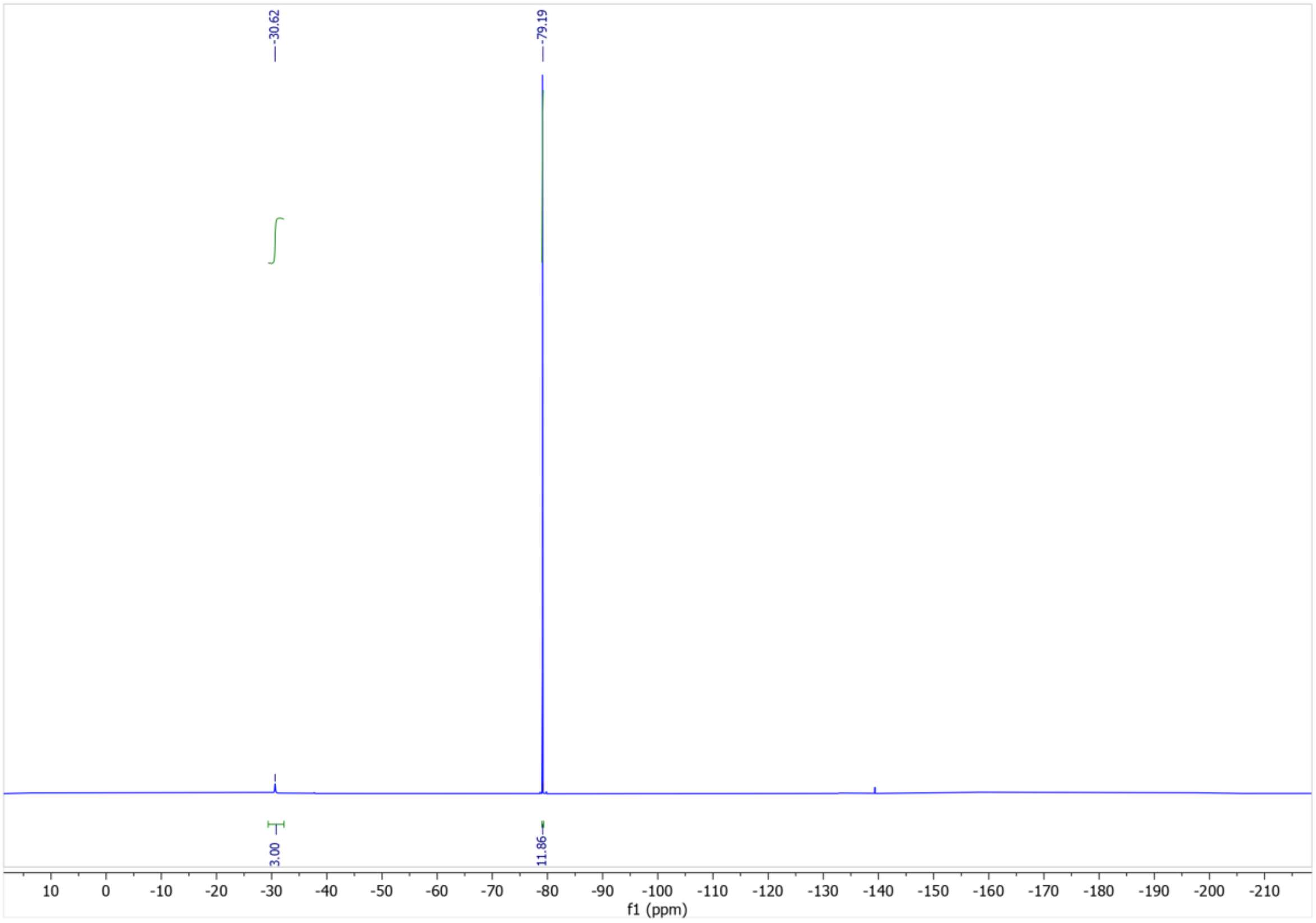

Figure S69. ^{19}F NMR spectrum of compound **13** in DCM-d_2.

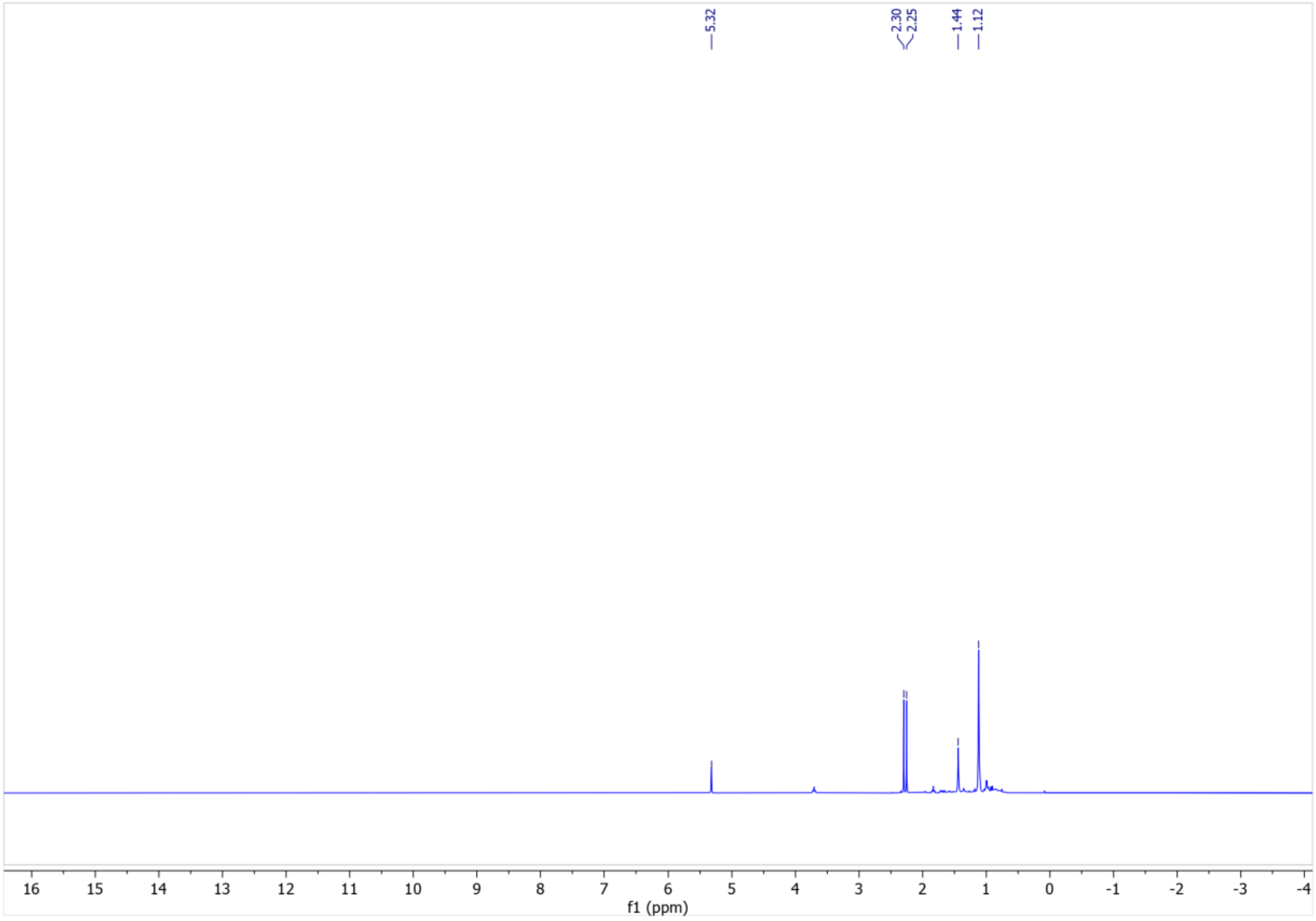

Figure S70. ^{1}H NMR spectrum of compound **13** in DCM-*d₂*.

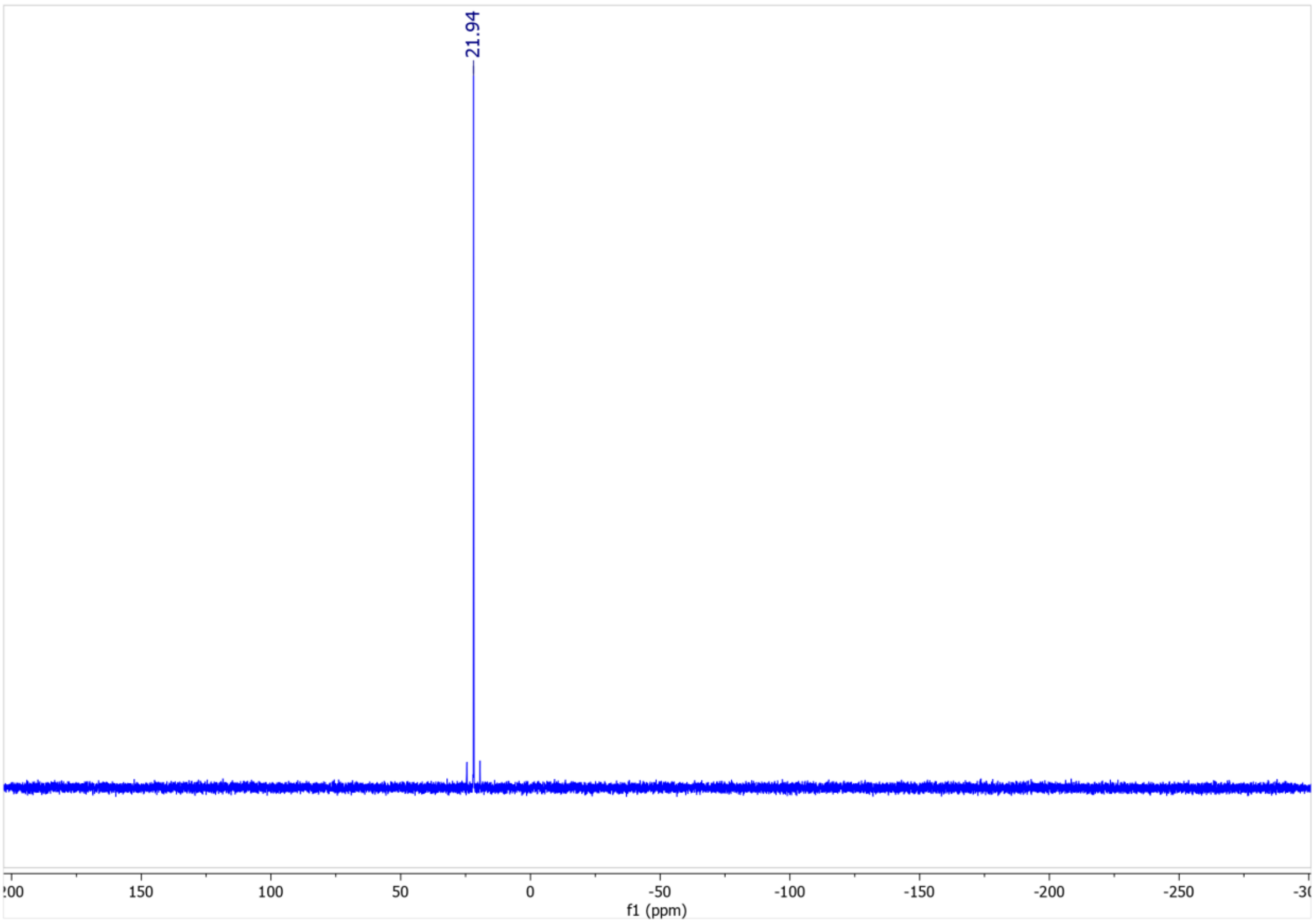

Figure S71. ^{31}P NMR spectrum of compound **13** in DCM-*d₂*.

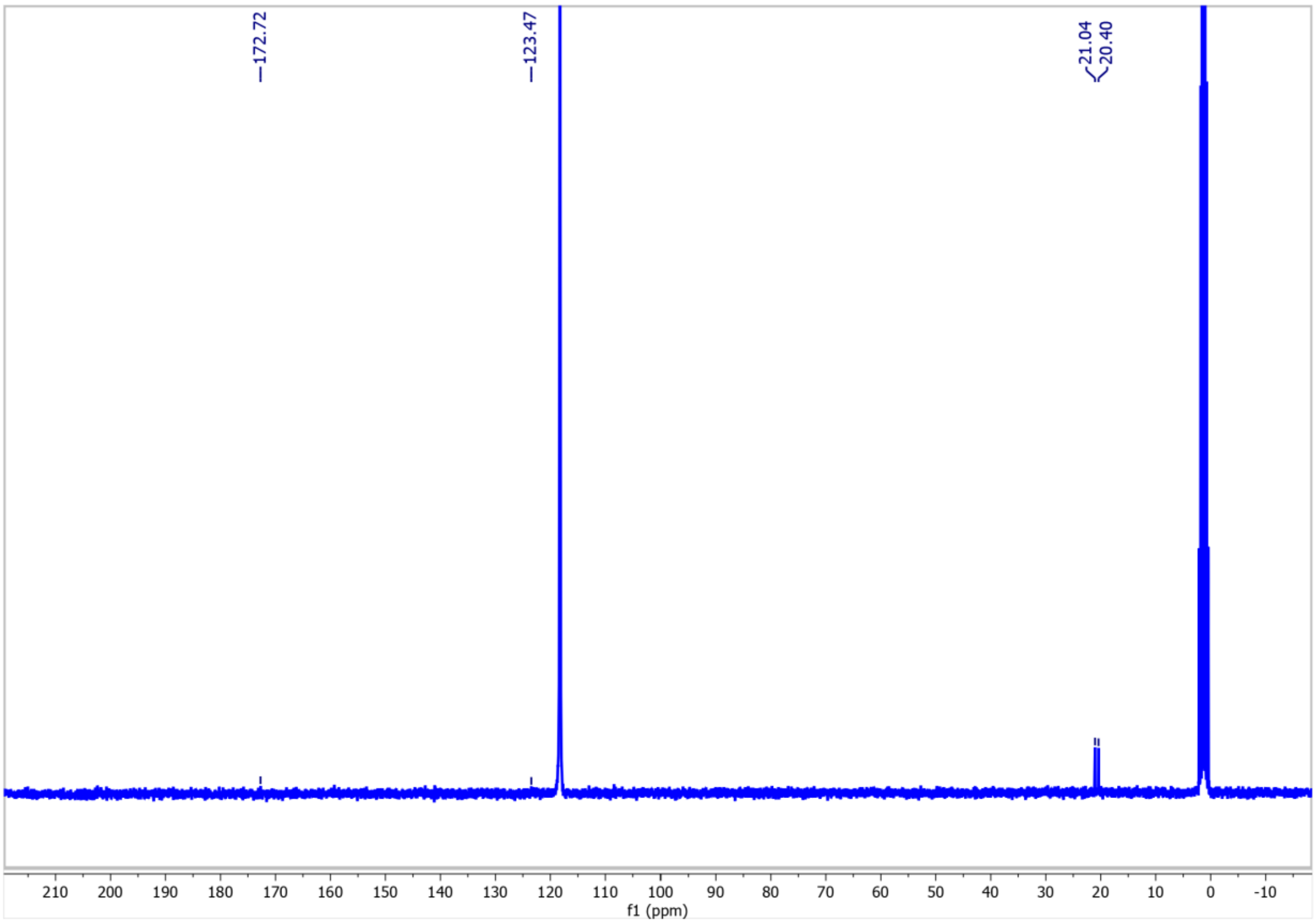

Figure S72. ^{13}C NMR spectrum of compound **13** in DCM-*d*$_2$.

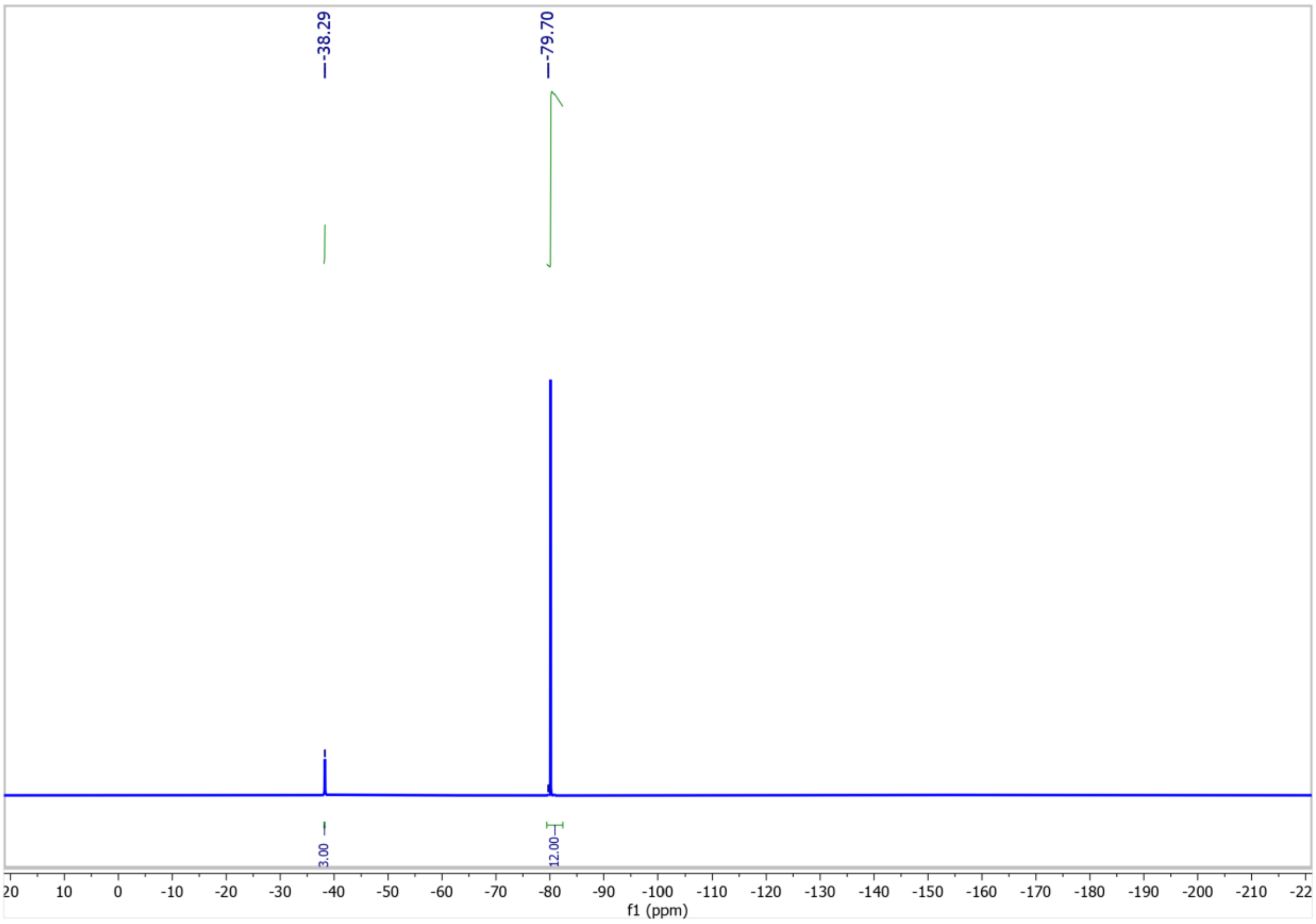

Figure S73. ^{19}F NMR spectrum of compound **[Bi(CF$_3$)(thf)(NTf$_2$)$_2$(bipy)]** in MeCN-*d*$_3$.

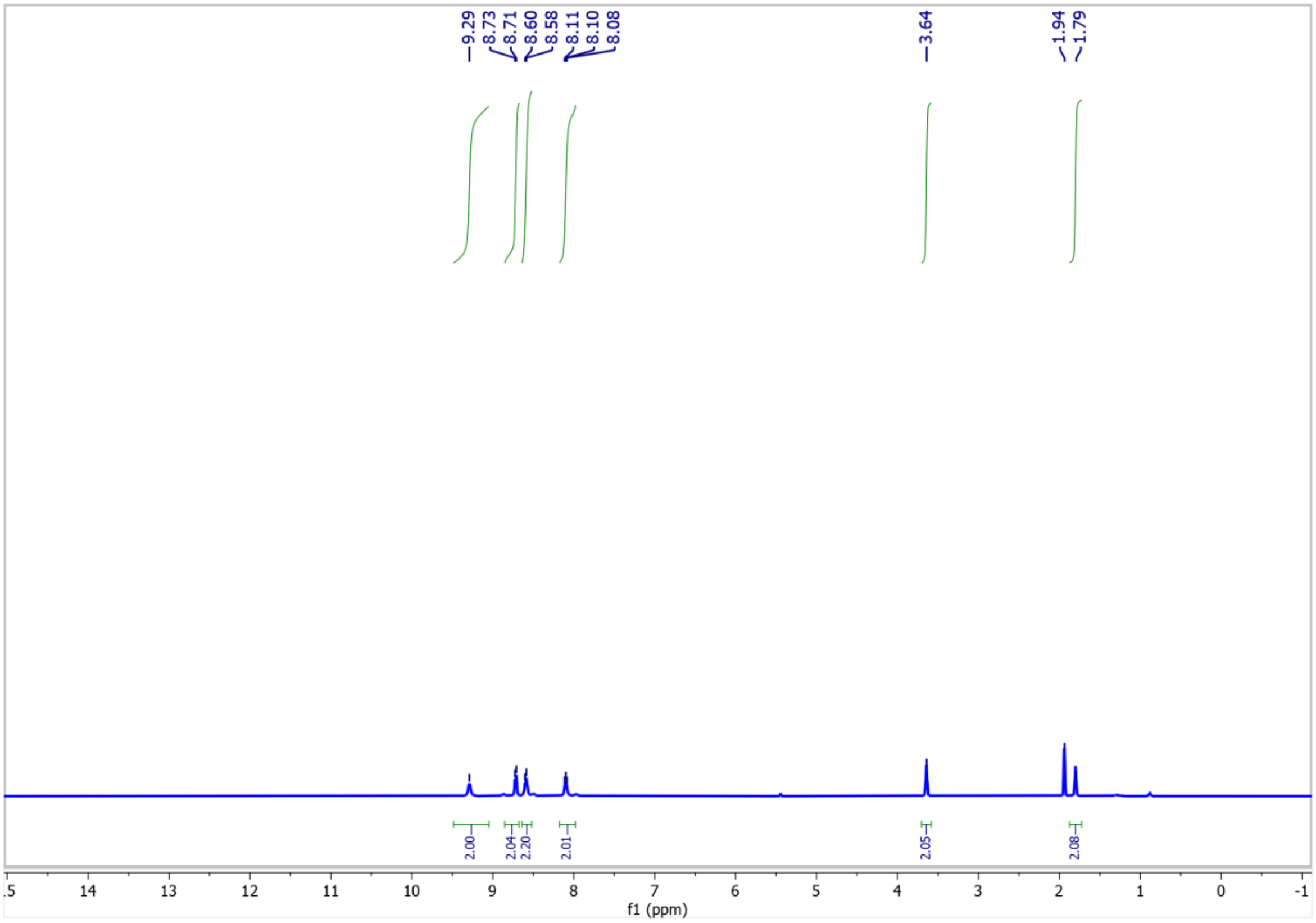

Figure S74. ^{1}H NMR spectrum of compound **[Bi(CF$_3$)(thf)(NTf$_2$)$_2$(bipy)]** in MeCN-d_3.

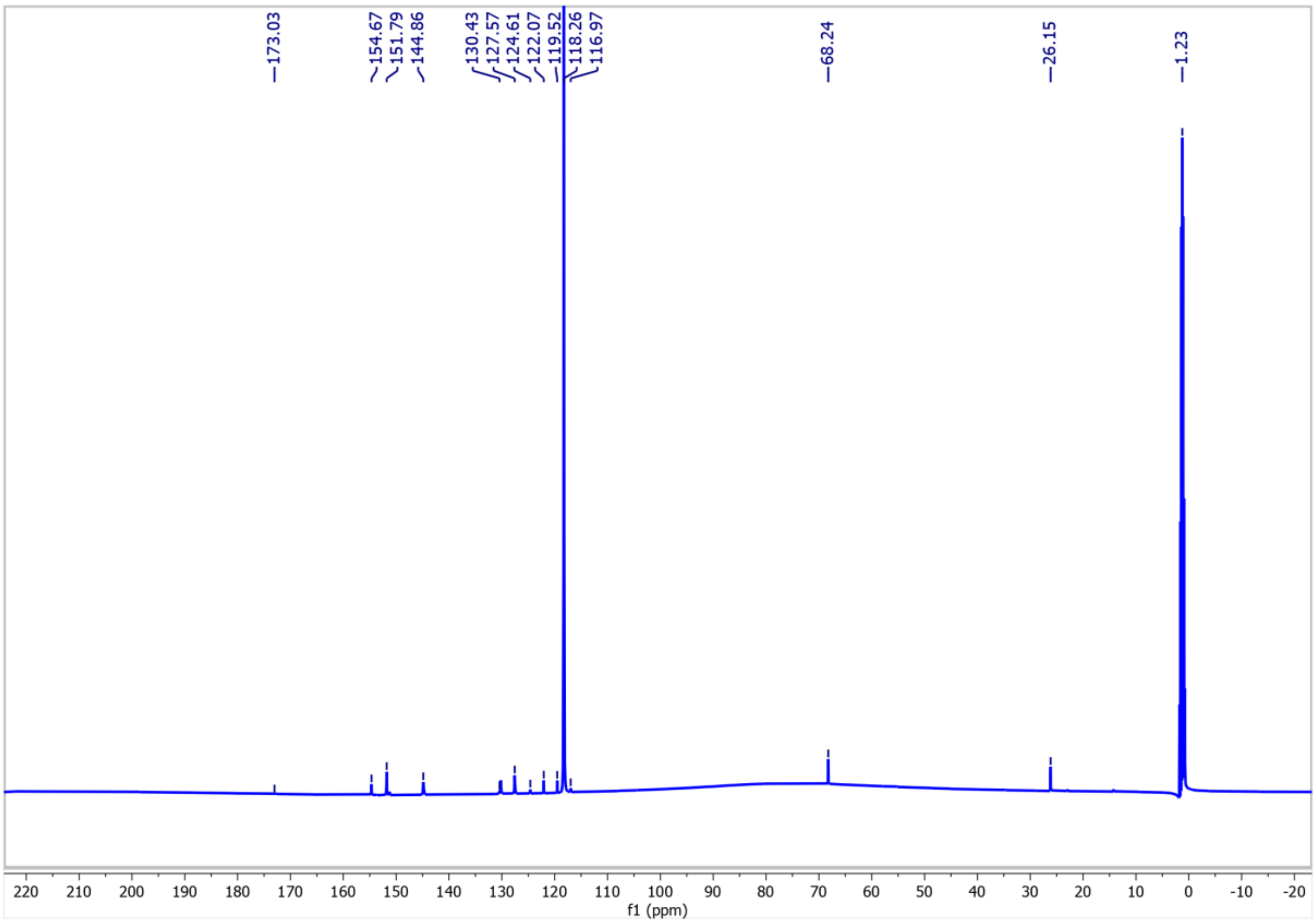

Figure S75. ^{13}C NMR spectrum of compound **[Bi(CF$_3$)(thf)(NTf$_2$)$_2$(bipy)]** in MeCN-d_3.

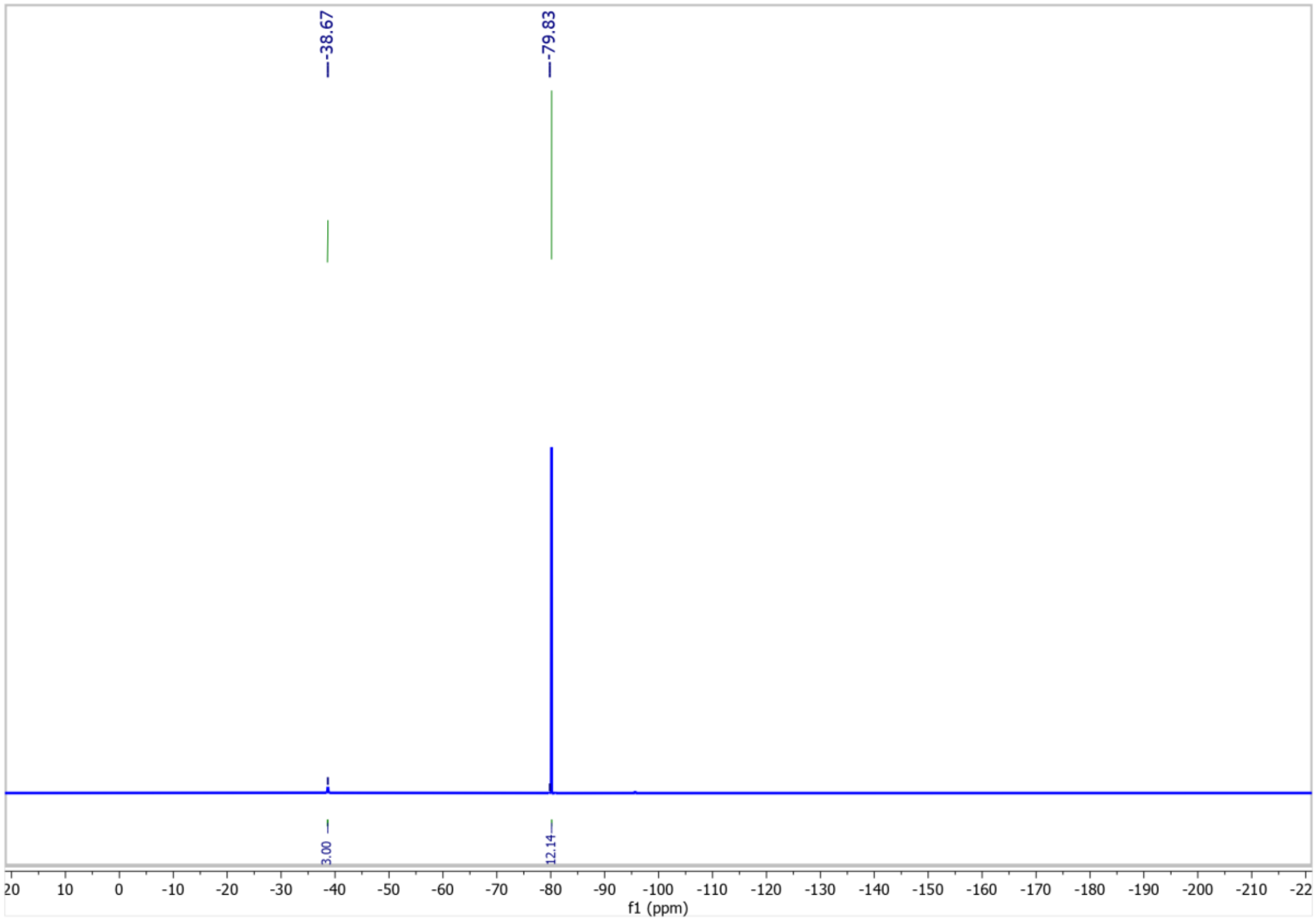

Figure S76. ^{19}F NMR spectrum of compound **[Bi(CF$_3$)(thf)(NTf$_2$)$_2$(phen)]** in MeCN-*d$_3$*.

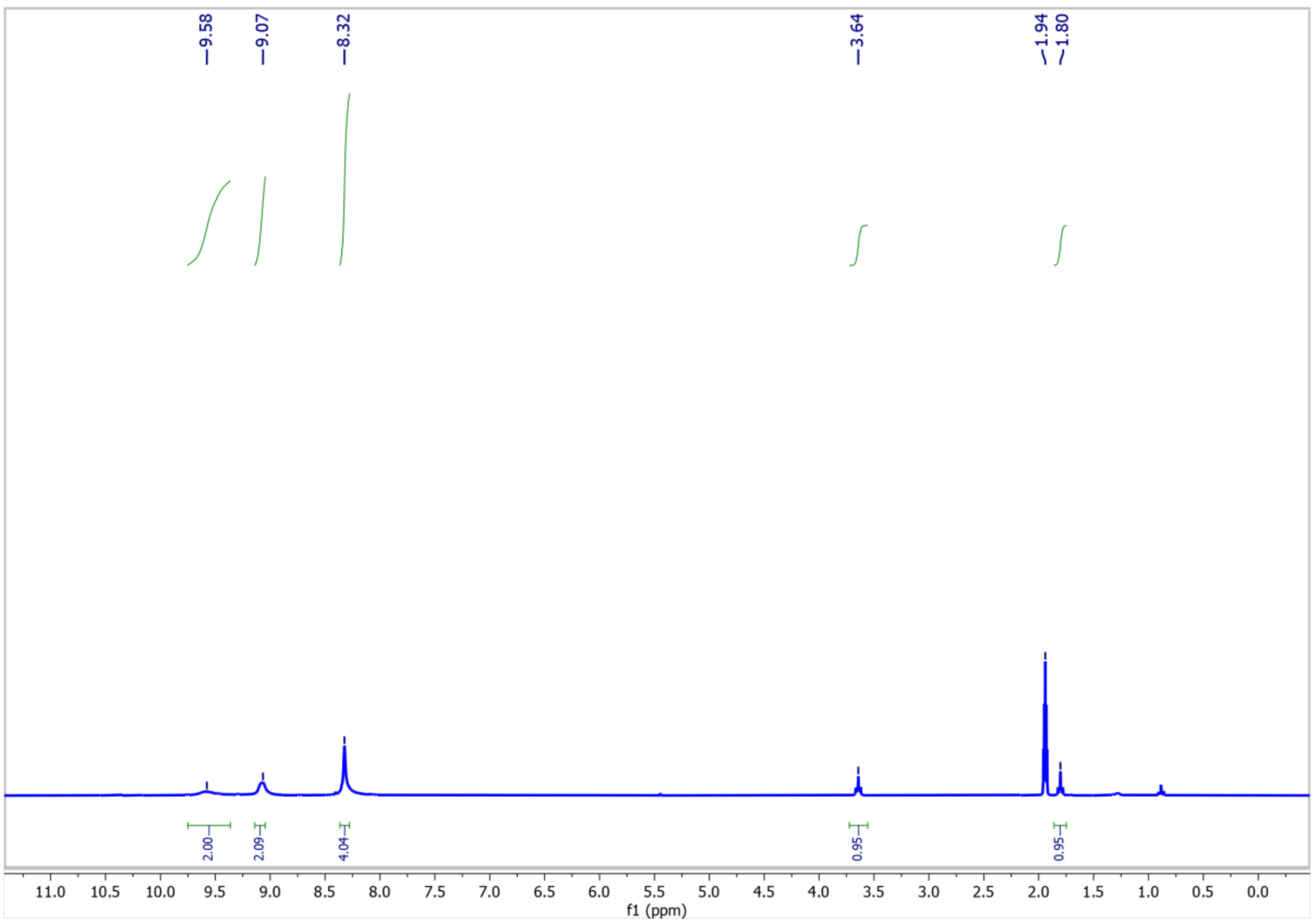

Figure S77. ^{1}H NMR spectrum of compound **[Bi(CF₃)(thf)(NTf₂)₂(phen)]** in MeCN-*d₃*.

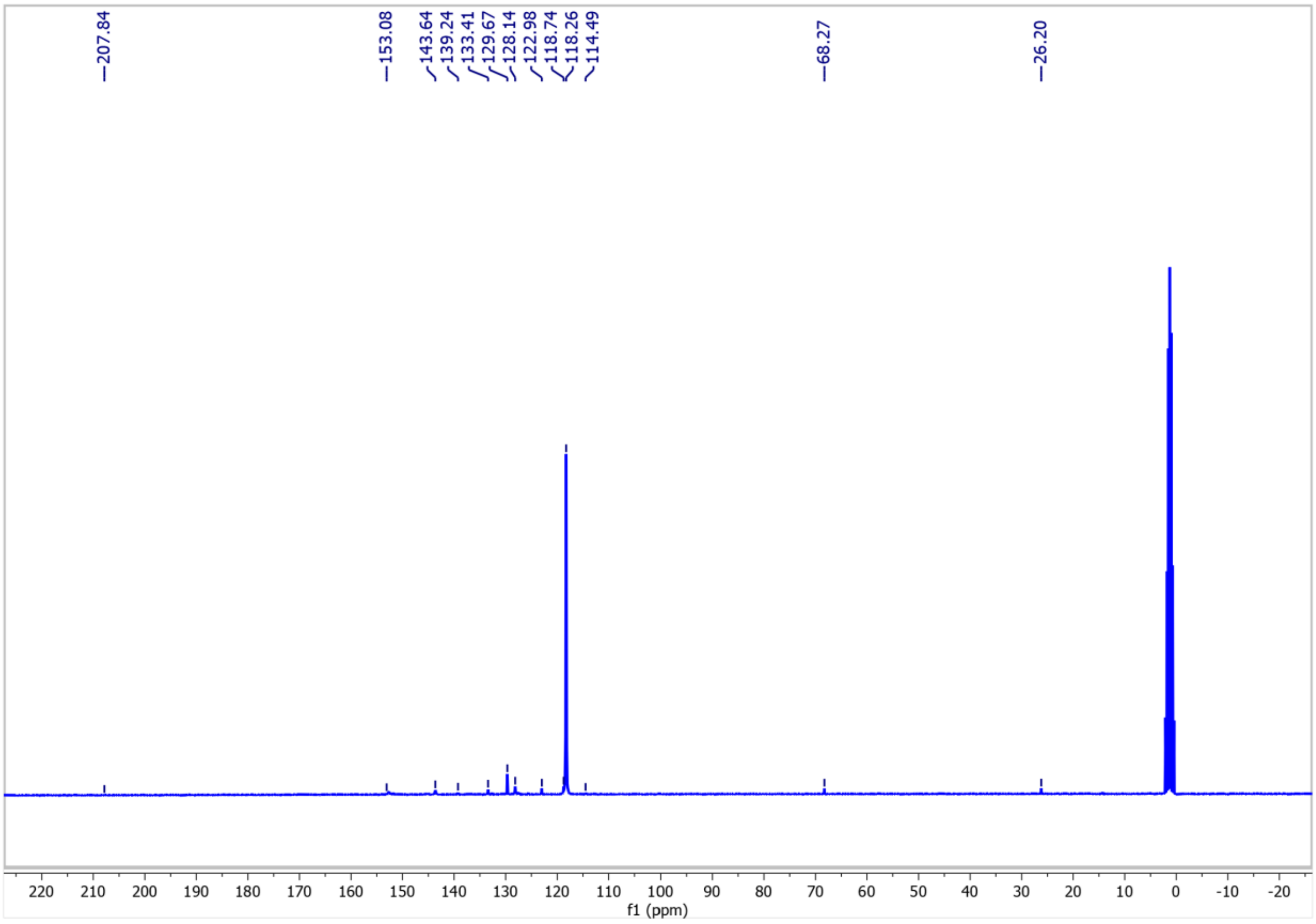

Figure S78. ^{13}C NMR spectrum of compound **[Bi(CF$_3$)(thf)(NTf$_2$)$_2$(phen)]** in MeCN-*d$_3$*.

7. Catalysis

General procedure:

All the manipulations were carried out under an argon atmosphere either using Schlenk techniques or a glovebox. J-Young NMR tubes were oven-dried at 110 °C. The phosphane oxides were purchased from TCI or BLD pharma and used without further purification. The catalytic reactions were performed in C_6D_6 unless stated otherwise.

Experimental procedure for reduction of phosphine oxide:

The required amount of the catalyst (typically: 2 mol% of [Bi(CF_3)(OTf)$_2$(thf)$_2$] (**4**)) and the respective phosphine oxide (typically 50 mg) were loaded into a J-Young NMR tube in a glovebox. C_6D_6 (0.6 mL) was added to the reaction mixture, followed by 3 equivalents of $PhSiH_3$ (with respect to the phosphane oxide). The NMR tube was then closed, removed from the glovebox, and transferred to a heating block pre-heated to 60 °C. The reaction was then monitored at various time intervals by ^{31}P NMR until complete conversion of the phosphane oxide to the phosphane was achieved. ^{1}H NMR spectra are provided for the reaction mixture after complete conversion of the phosphane oxide.

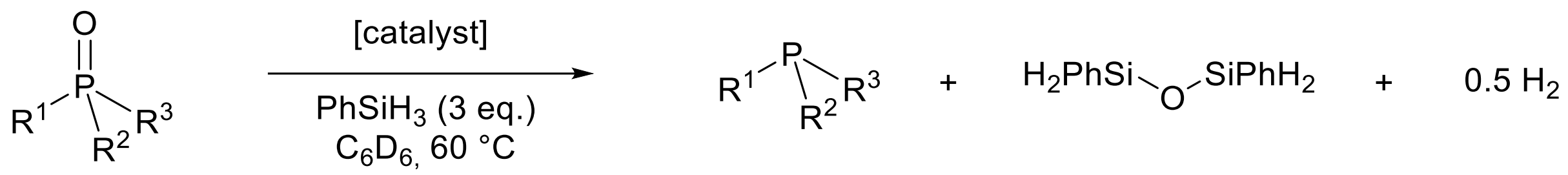

Proposed Mechanism:

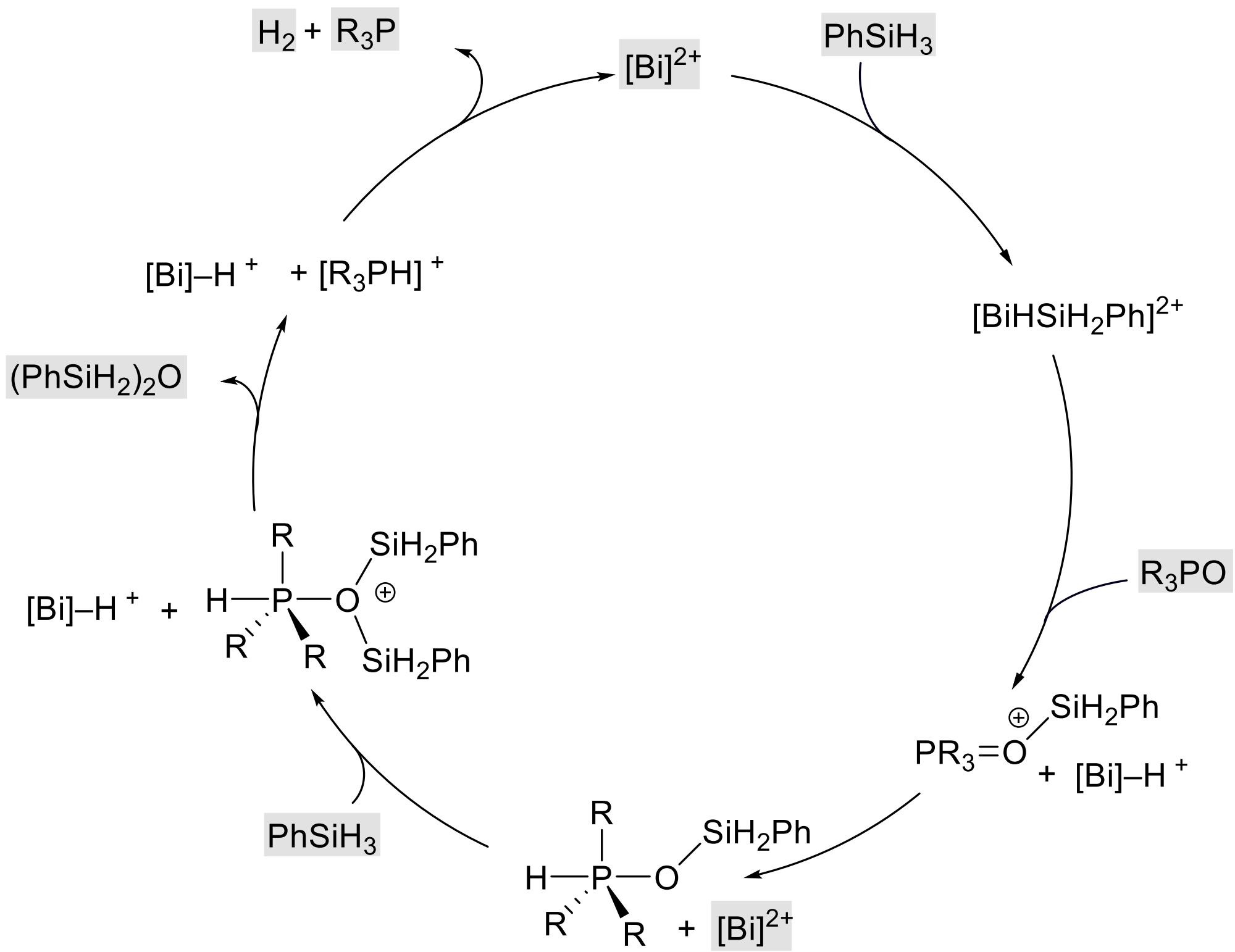

Scheme S1. Tentatively suggested mechanism[15] (experimentally detected components highlighted with a grey box). Compound (**4**) is abbreviated as $[Bi]^{2+}$. Triflate anions are omitted for clarity.

In addition to the quantification of the desired products **P1-P9**, H_2 and $(PhSiH_2)_2O$ were detected as products of the reaction by ^{1}H and ^{29}Si NMR spectroscopy, respectively.[1,16] The formation of a tentatively proposed bismuth hydride intermediate would be in agreement with the fact that the stabilization of a bismuth hydride species by electron-withdrawing substituents has been suggested $(HBi(C_2F_5)_2$ has been reported to be generated in situ at low temperature and release H_2 at temperatures of ≥ -30 °C).[17]

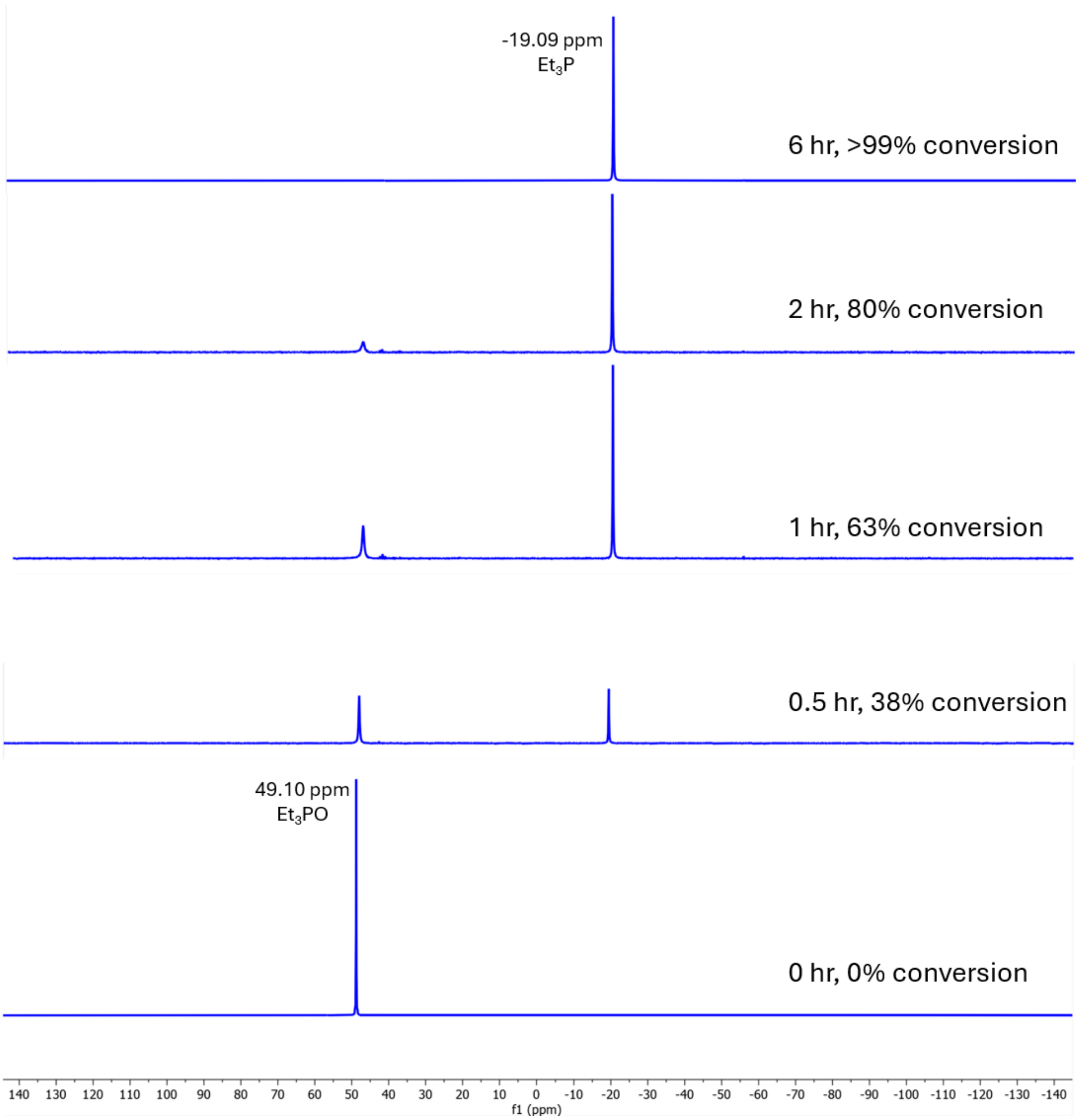

Figure S79: Stacked ^{31}P NMR spectra for the reduction of Et_3PO with 3 equiv. $PhSiH_3$ to Et_3P using 2 mol% of compound **4** in C_6D_6.

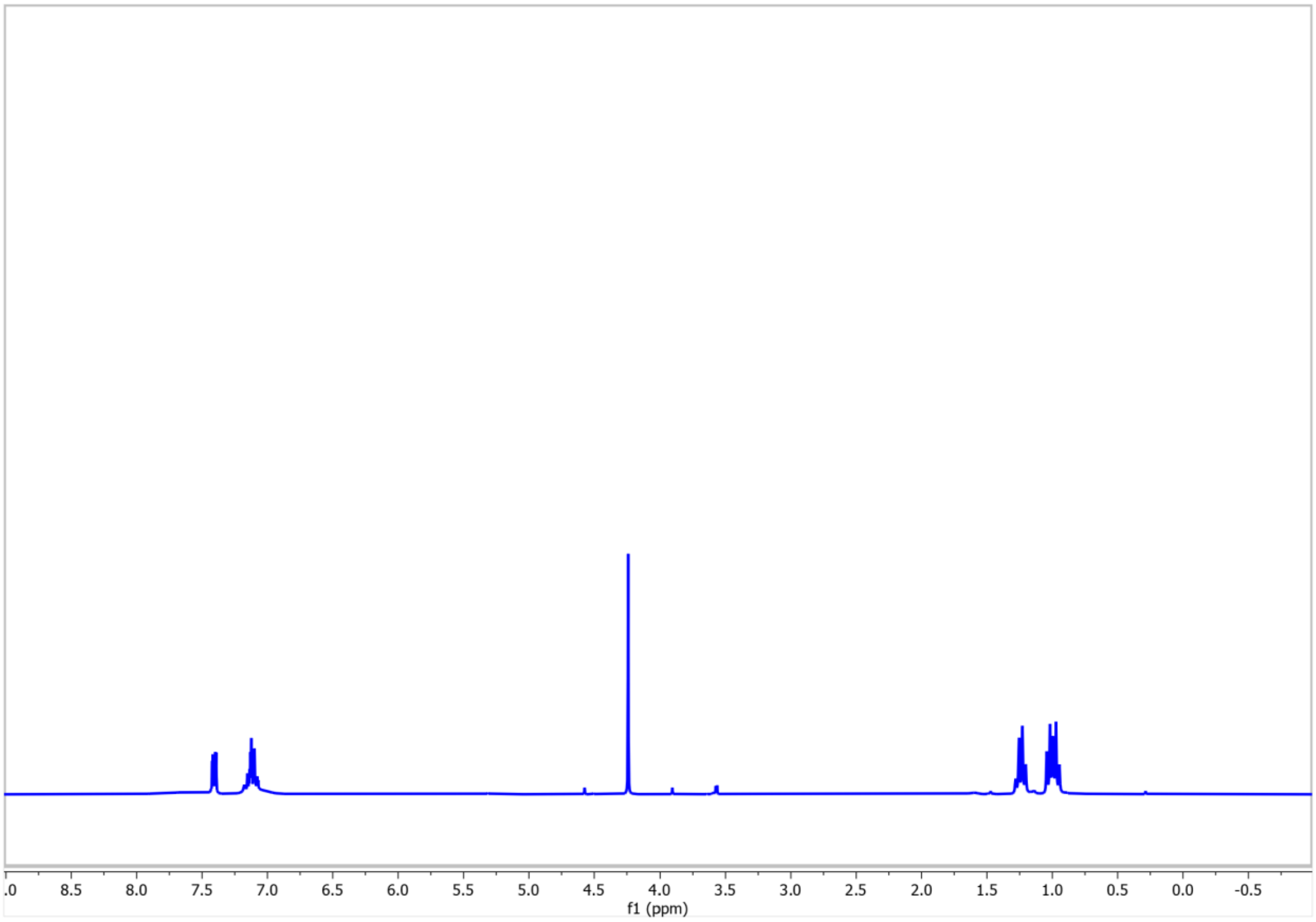

Figure S80: ^{1}H NMR spectrum after full conversion of Et_3PO with 3 equiv. $PhSiH_3$ to Et_3P using 2 mol% of compound **4** in C_6D_6.

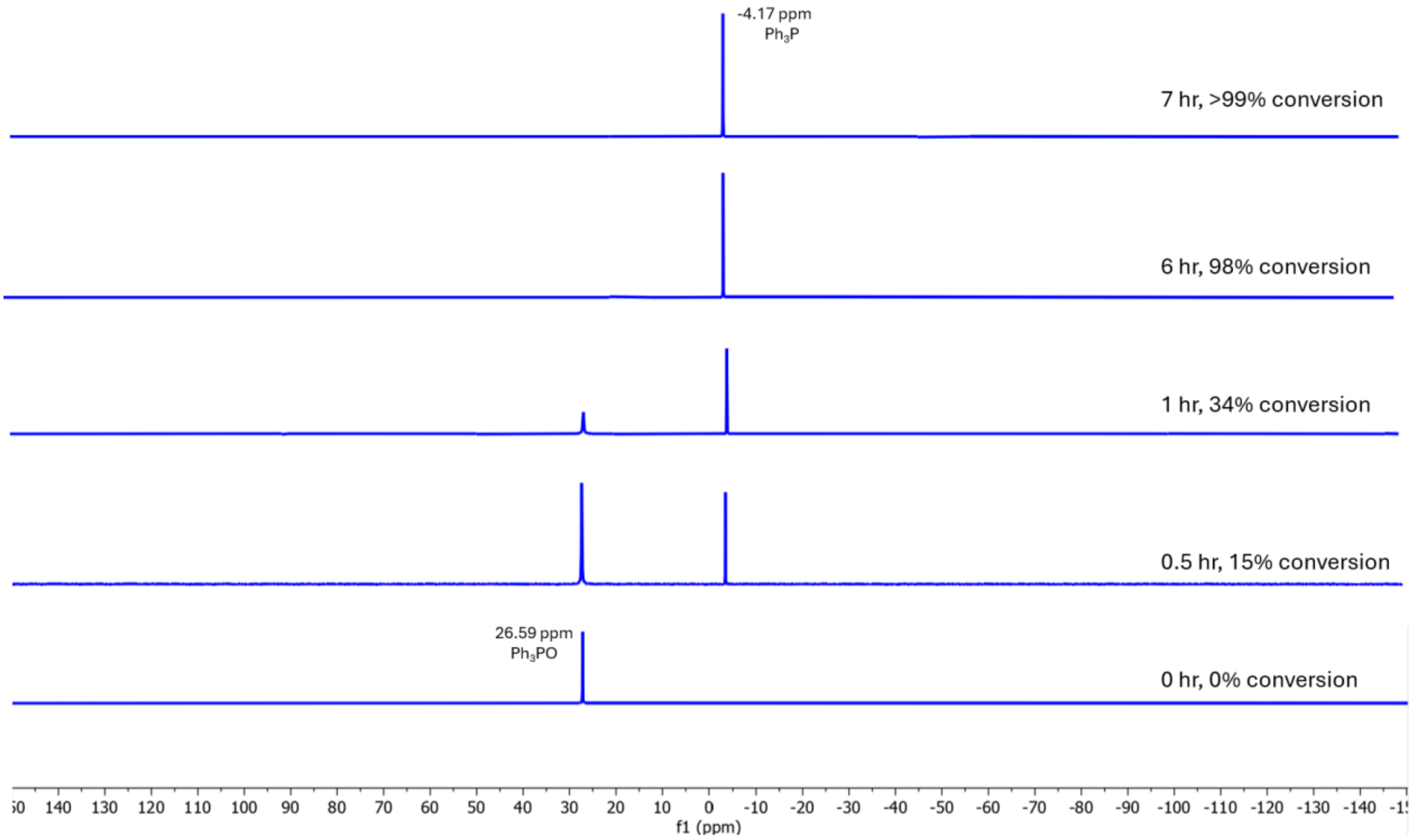

Figure S81: Stacked ^{31}P NMR spectra for the reduction of Ph_3PO with 3 equiv. $PhSiH_3$ to Ph_3P using 2 mol% of compound **4** in C_6D_6.

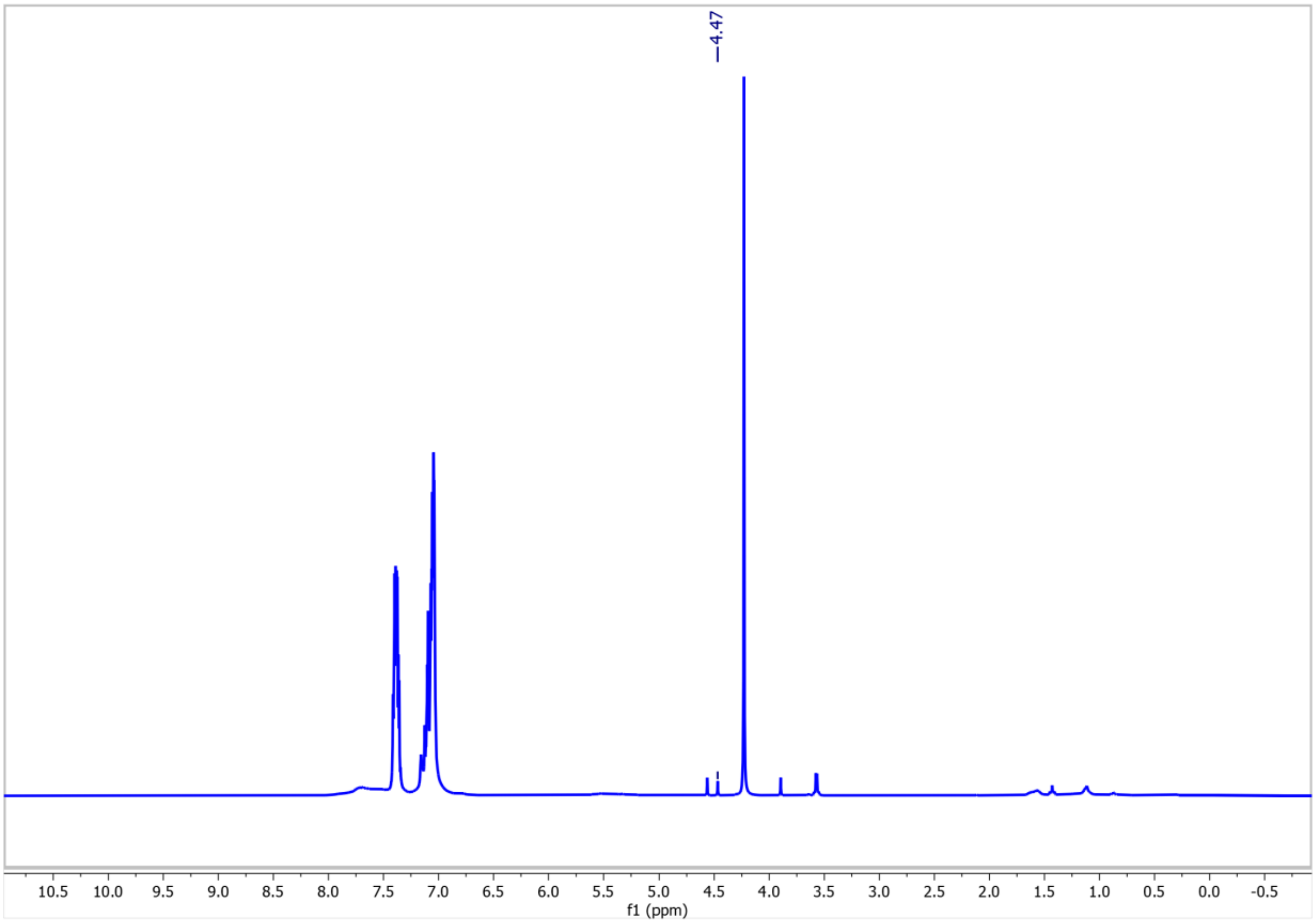

Figure S82: ^{1}H NMR spectrum after full conversion of Ph_3PO with 3 equiv. $PhSiH_3$ to Ph_3P using 2 mol% of compound **4** in C_6D_6.

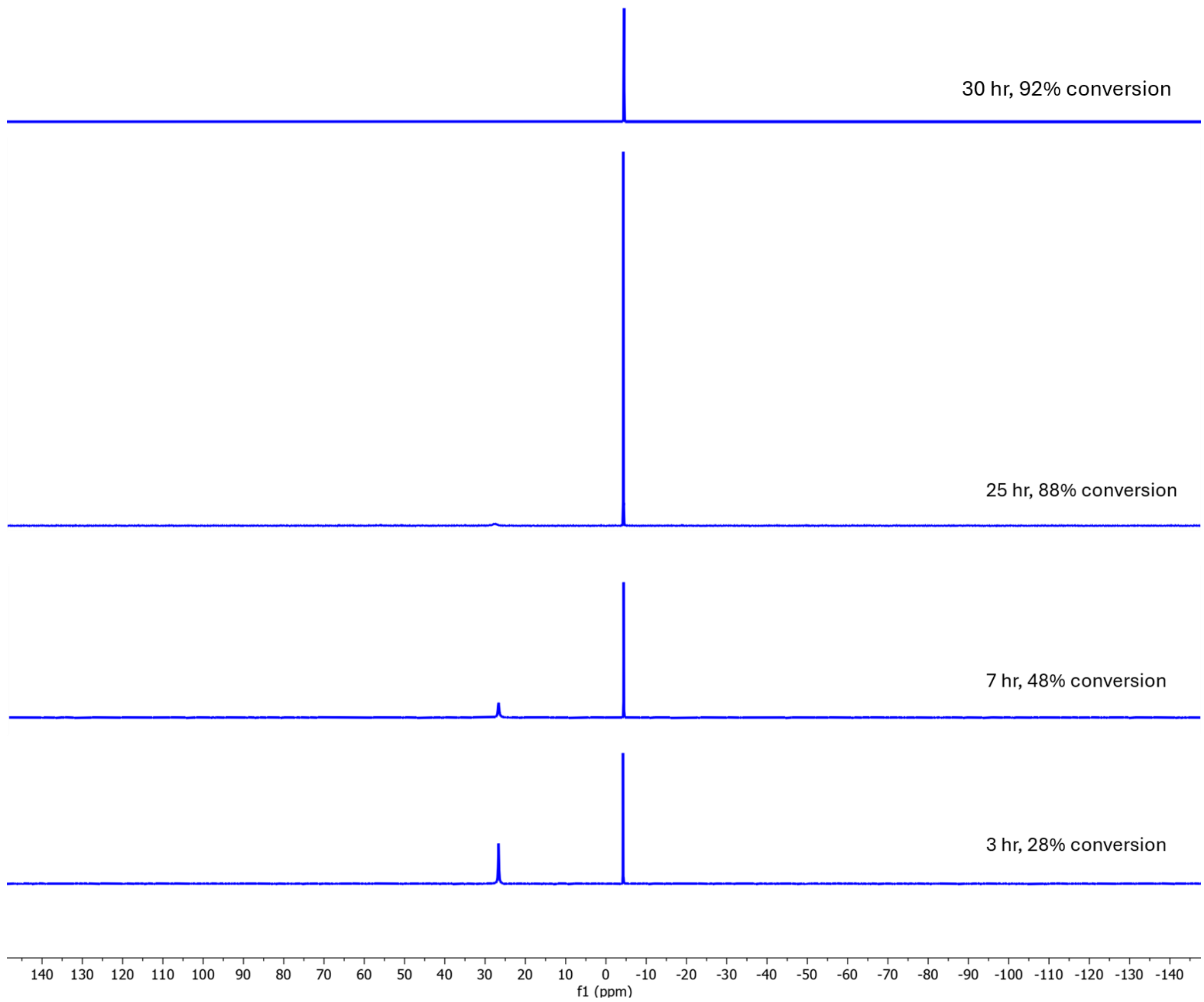

Figure S83: Stacked ^{31}P NMR spectra for the reduction of Ph_3PO with 3 equiv. $PhSiH_3$ to Ph_3P using 2 mol% of $Bi(O_3SCF_3)_3$ in C_6D_6.

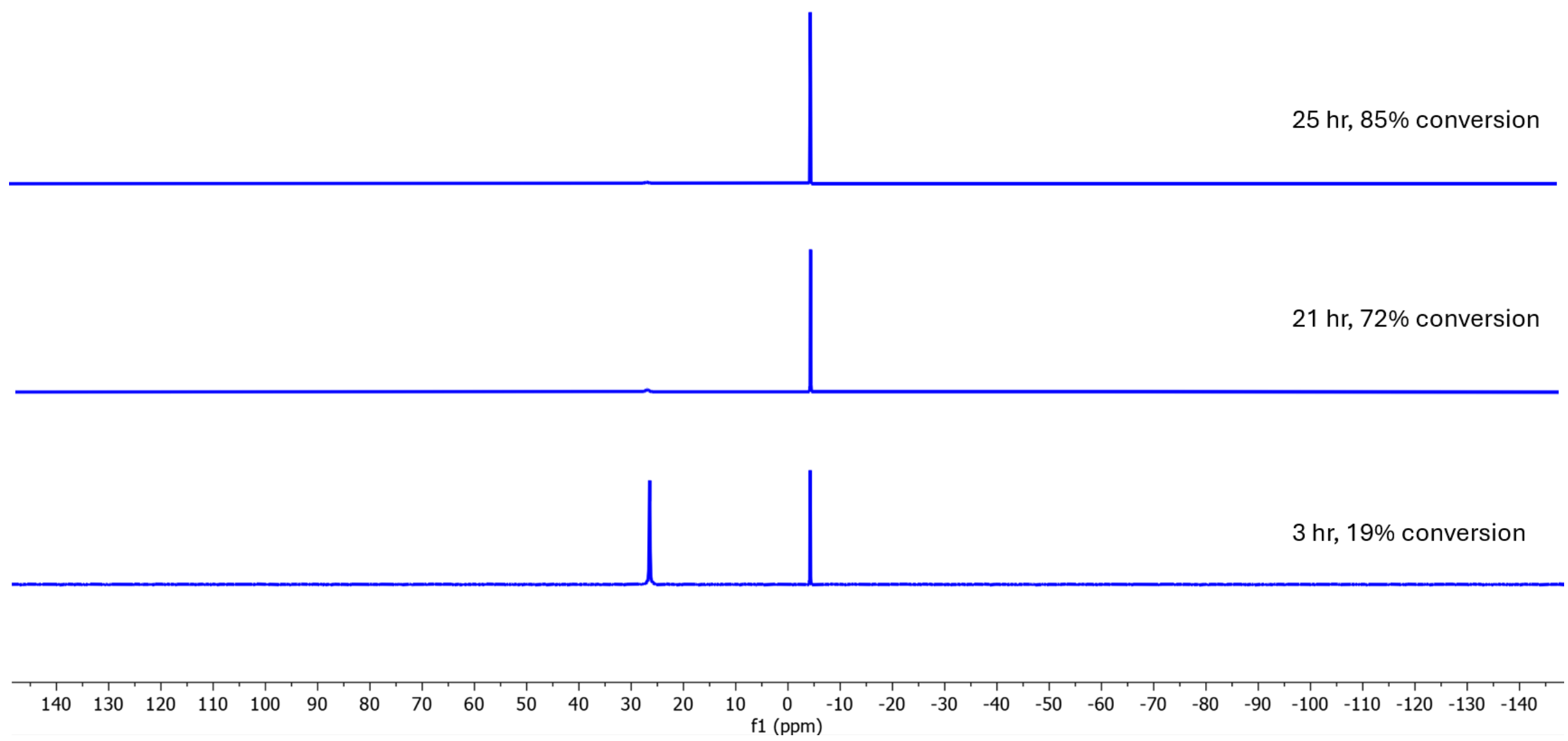

Figure S84: Stacked ^{31}P NMR spectra for the reduction of Ph_3PO with 3 equiv. $PhSiH_3$ to Ph_3P using 2 mol% of $[Bi(CH_3)(OTf)_2(thf)_2]$ in C_6D_6.

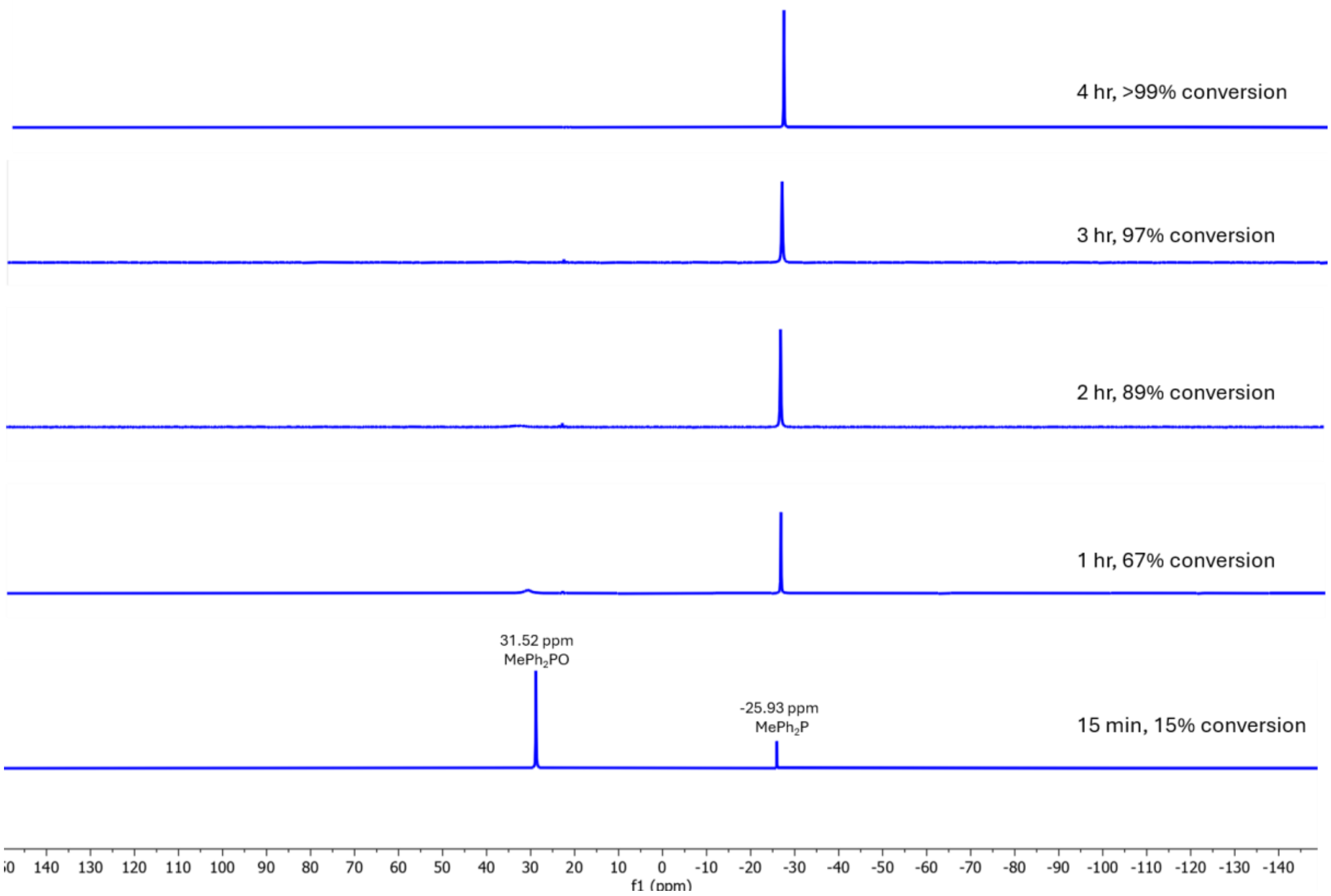

Figure S85: Stacked ^{31}P NMR spectra for the reduction of $MePh_2PO$ with 3 equiv. $PhSiH_3$ to $MePh_2P$ using 2 mol% of compound **4** in C_6D_6.

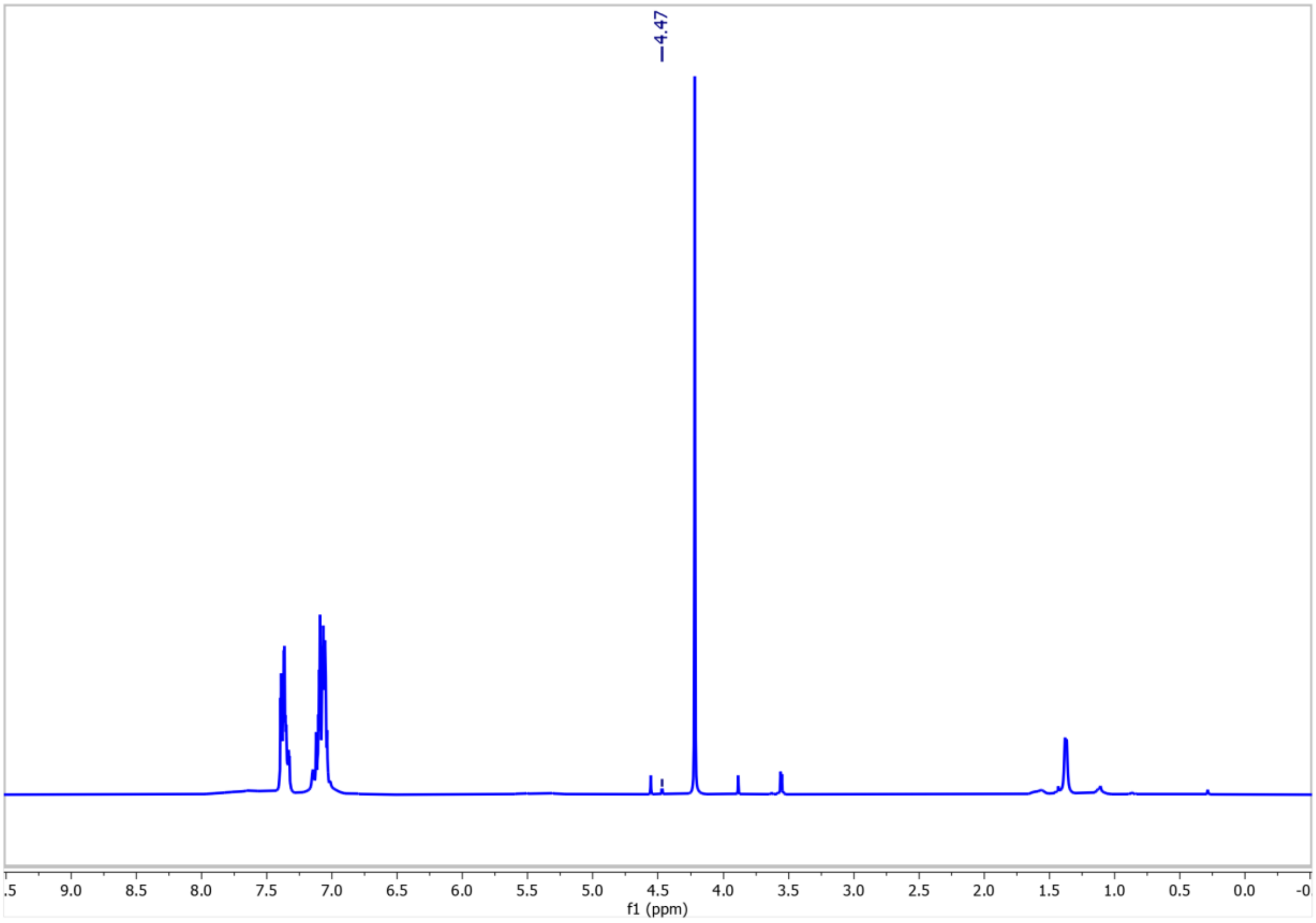

Figure S86: ^{1}H NMR spectrum after full conversion of $MePh_2PO$ with 3 equiv. $PhSiH_3$ to $MePh_2P$ using 2 mol% of compound **4** in C_6D_6.

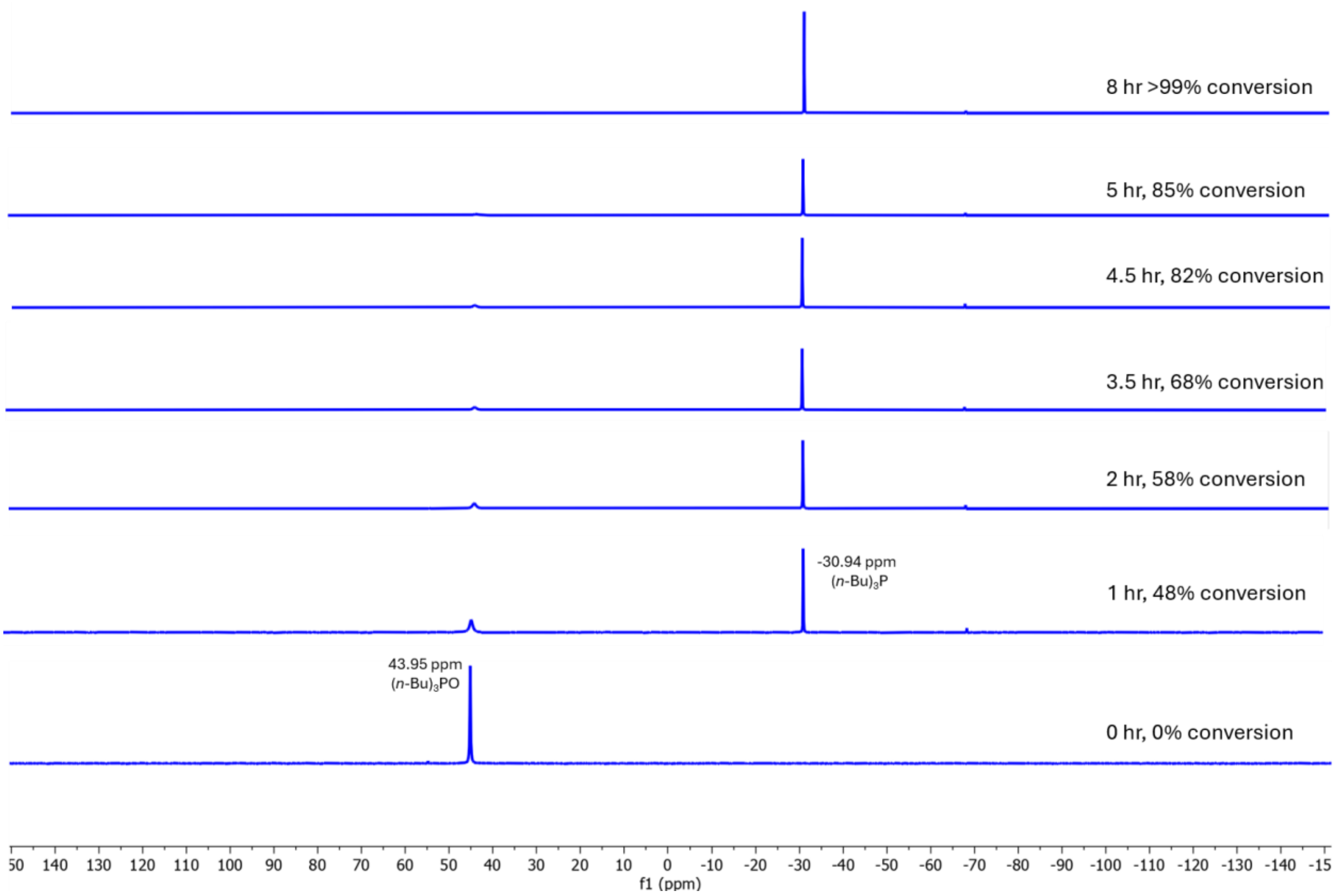

Figure S87: Stacked ^{31}P NMR spectra for the reduction of (*n*-Butyl)$_3$PO with 3 equiv. $PhSiH_3$ to (*n*-Butyl)$_3$P using 2 mol% of compound **4** in C_6D_6.

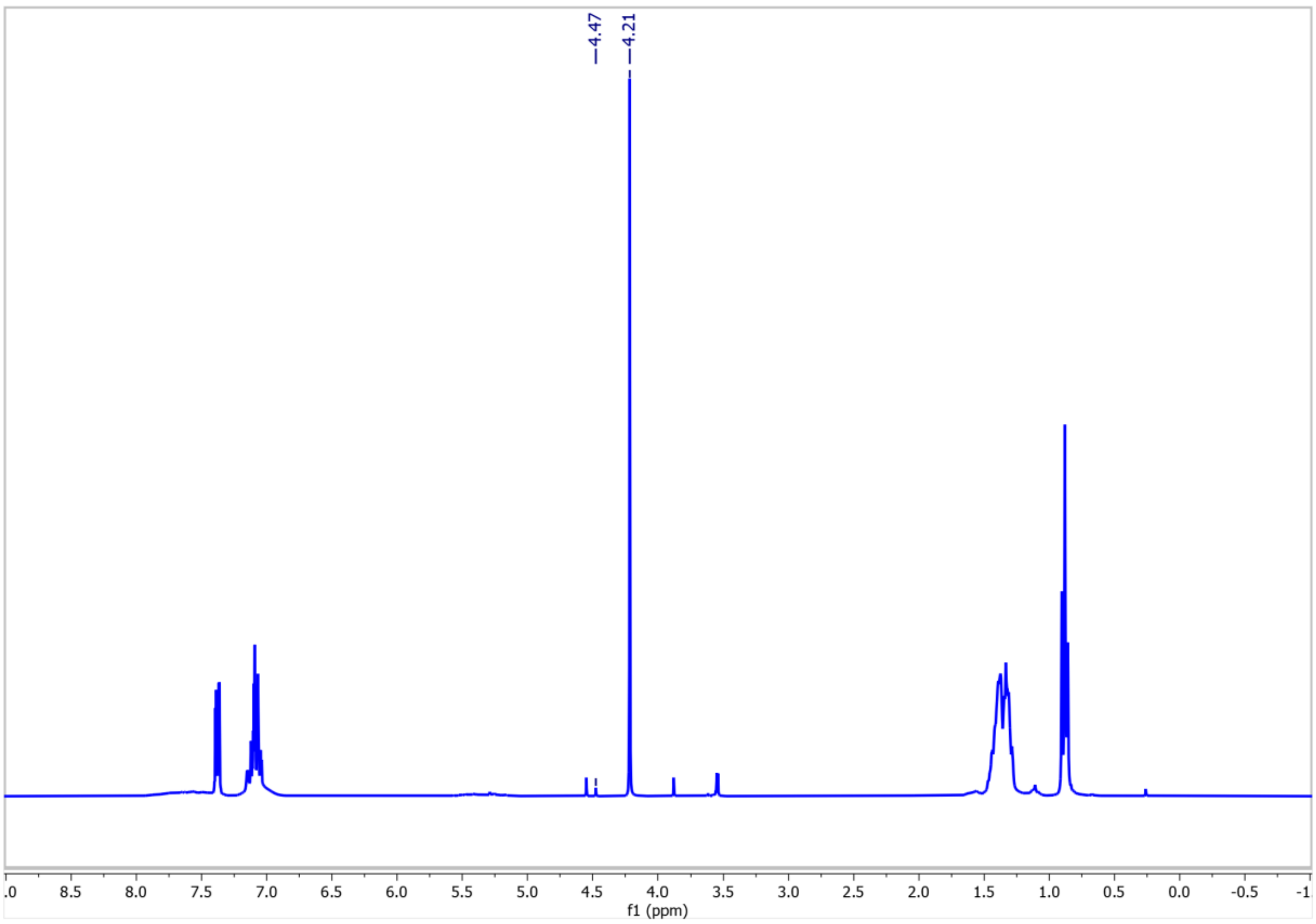

Figure S88: ^{1}H NMR spectrum after full conversion of (*n*-Butyl)$_3$PO with 3 equiv. $PhSiH_3$ to (*n*-Butyl)$_3$P using 2 mol% of compound **4** in C_6D_6.

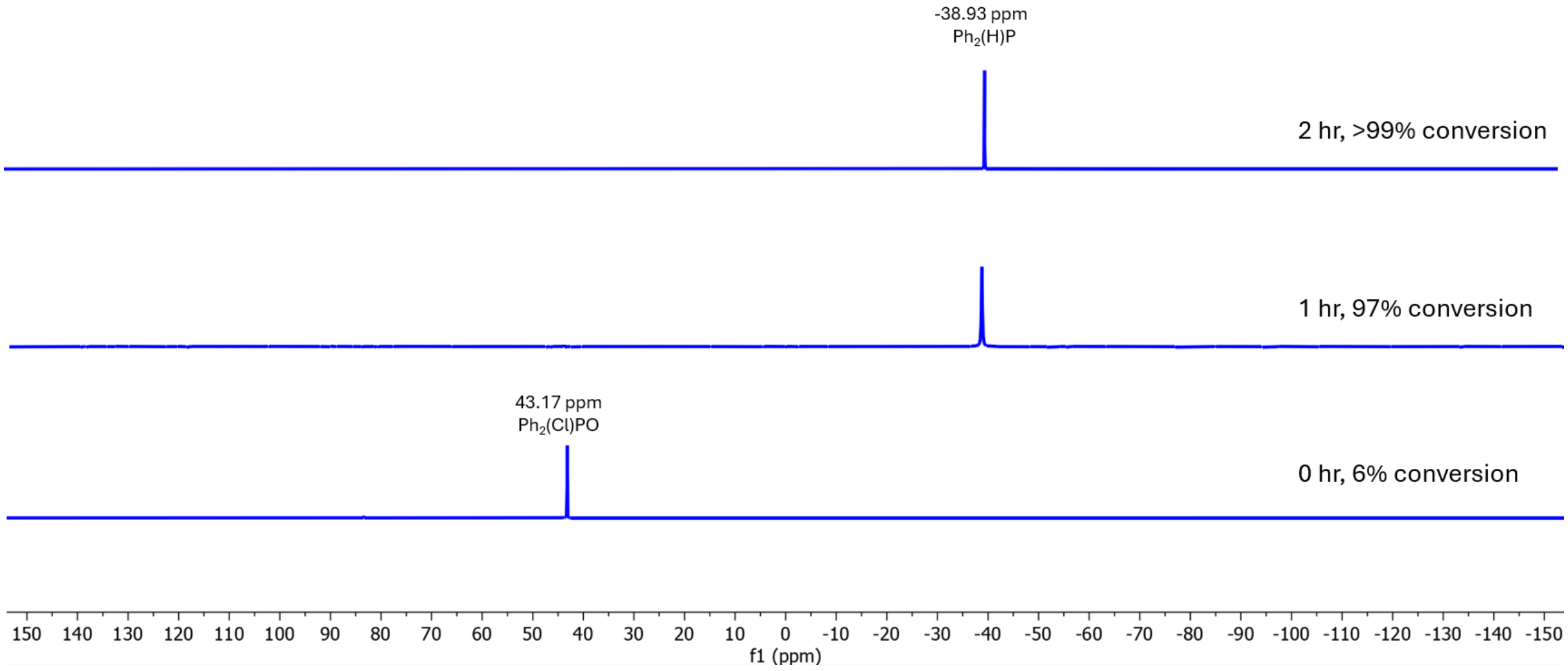

Figure S89: Stacked ^{31}P NMR spectra for the reduction of $Ph_2(Cl)PO$ with 3 equiv. $PhSiH_3$ to $Ph_2(H)P$ using 2 mol% of compound **4** in C_6D_6.

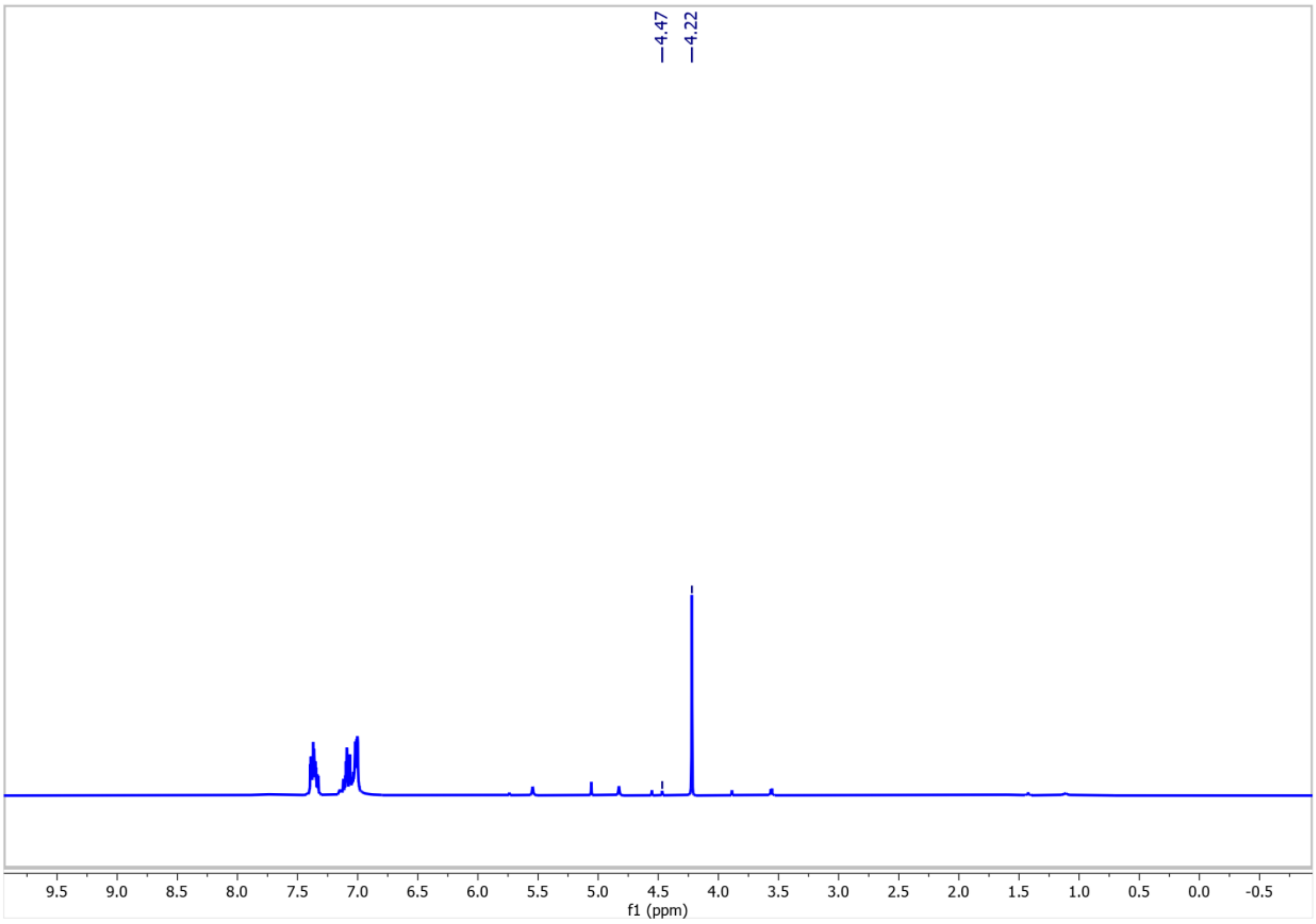

Figure S90: ^{1}H NMR spectrum after full conversion of $Ph_2(Cl)PO$ with 3 equiv. $PhSiH_3$ to $Ph_2(H)P$ using 2 mol% of compound **4** in C_6D_6.

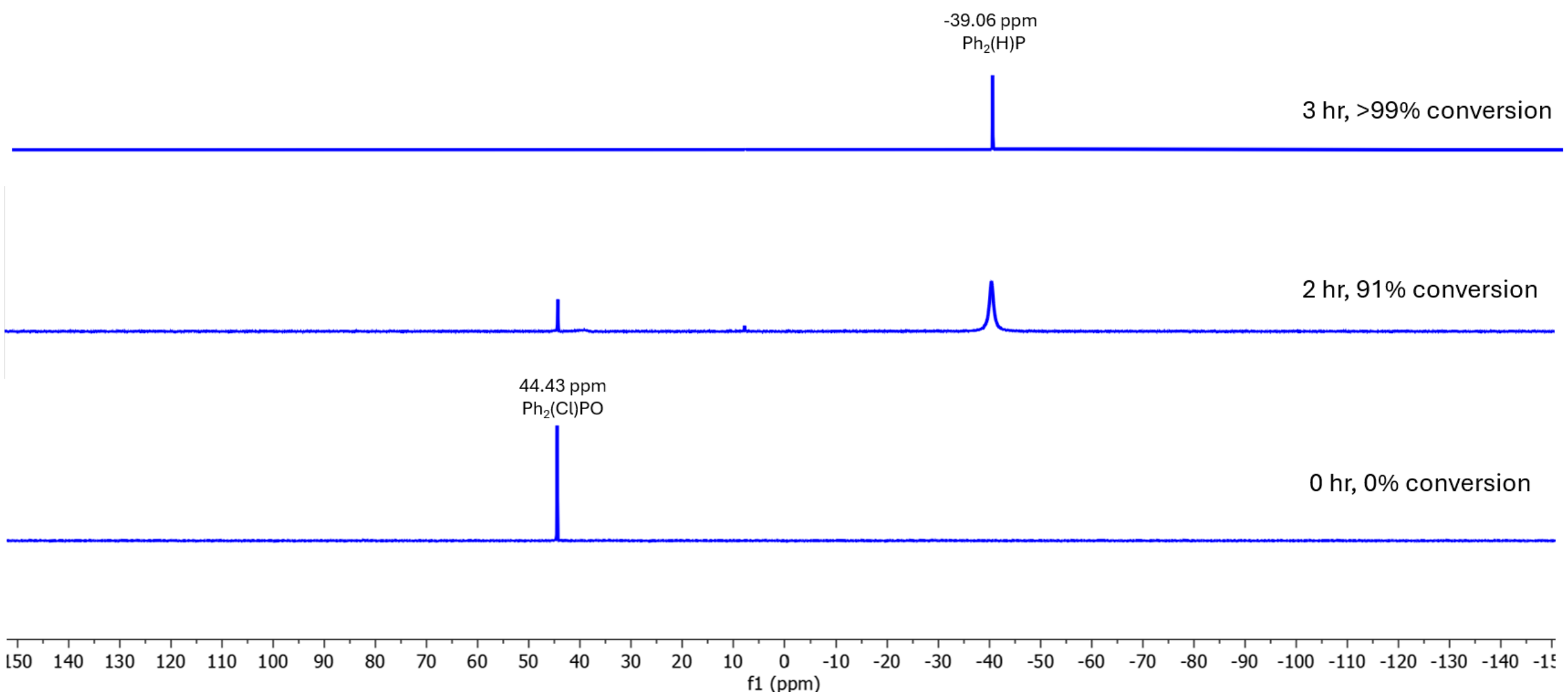

Figure S91: Stacked ^{31}P NMR spectra for the reduction of $Ph_2(Cl)PO$ with 3 equiv. $PhSiH_3$ to $Ph_2(H)P$ using 2 mol% of compound **4** in CD_2Cl_2.

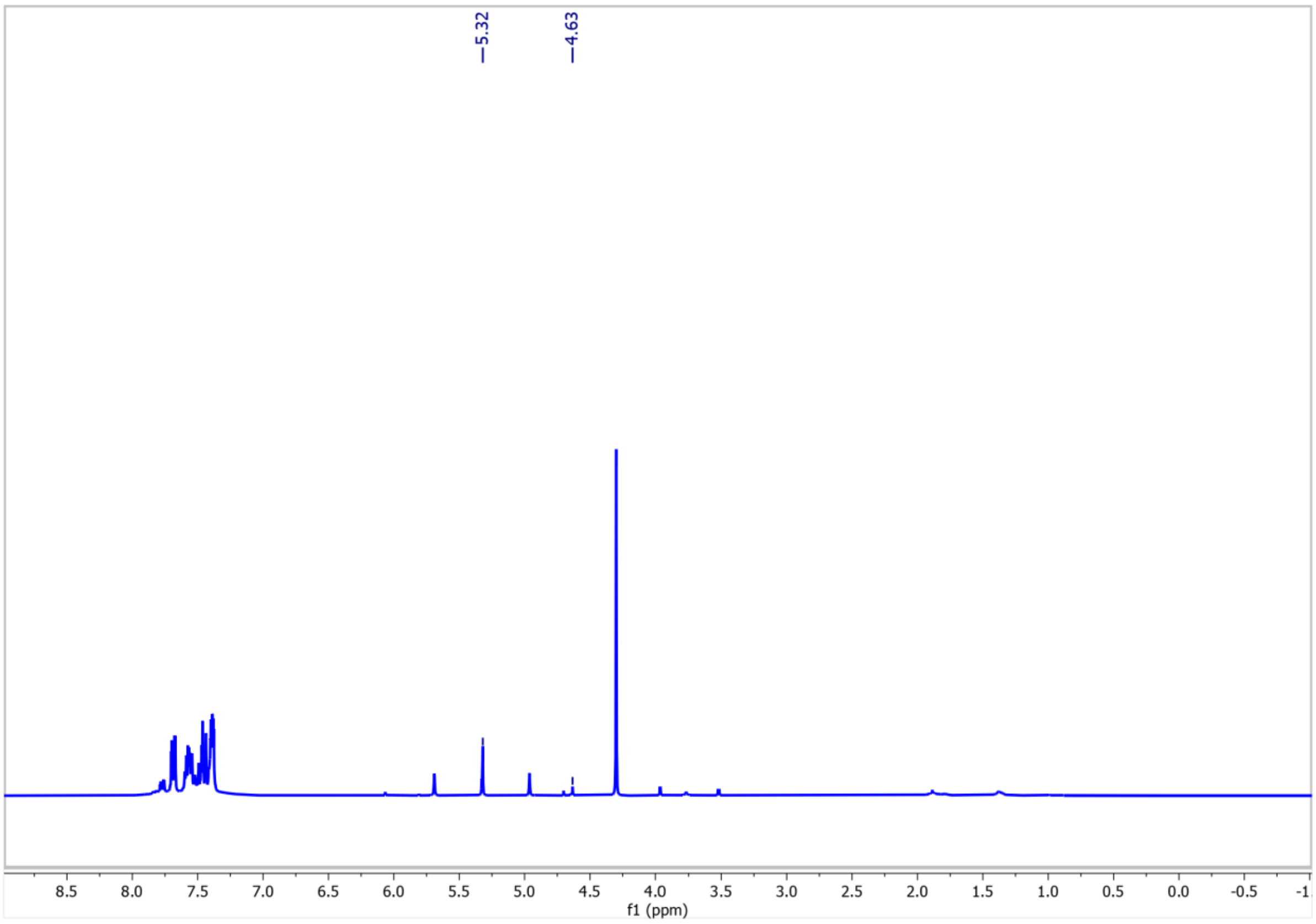

Figure S92: ^{1}H NMR spectra after full conversion of $Ph_2(Cl)PO$ with 3 equiv. $PhSiH_3$ to $Ph_2(H)P$ using 2 mol% of compound **4** in CD_2Cl_2.

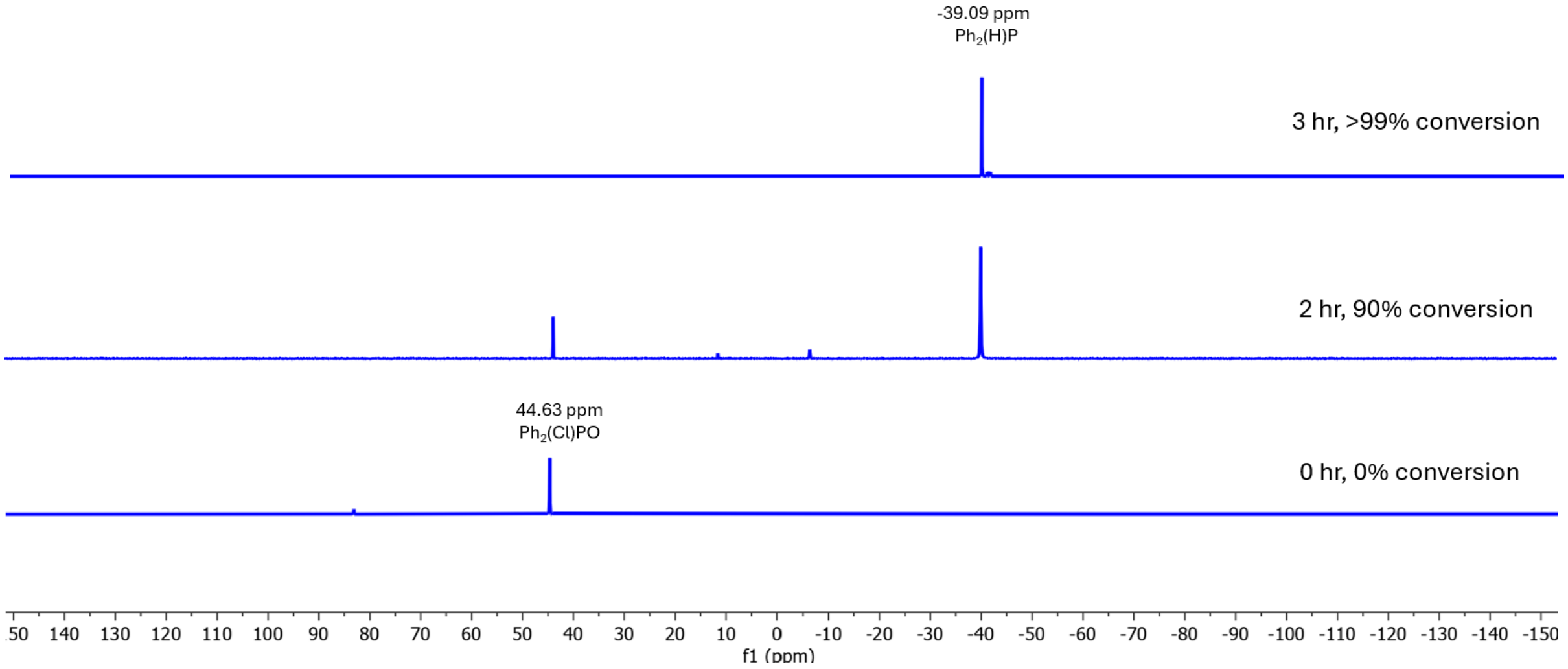

Figure S93: Stacked ^{31}P NMR spectra for the reduction of $Ph_2(Cl)PO$ with 3 equiv. $PhSiH_3$ to $Ph_2(H)P$ using 2 mol% of compound **4** in CD_3CN.

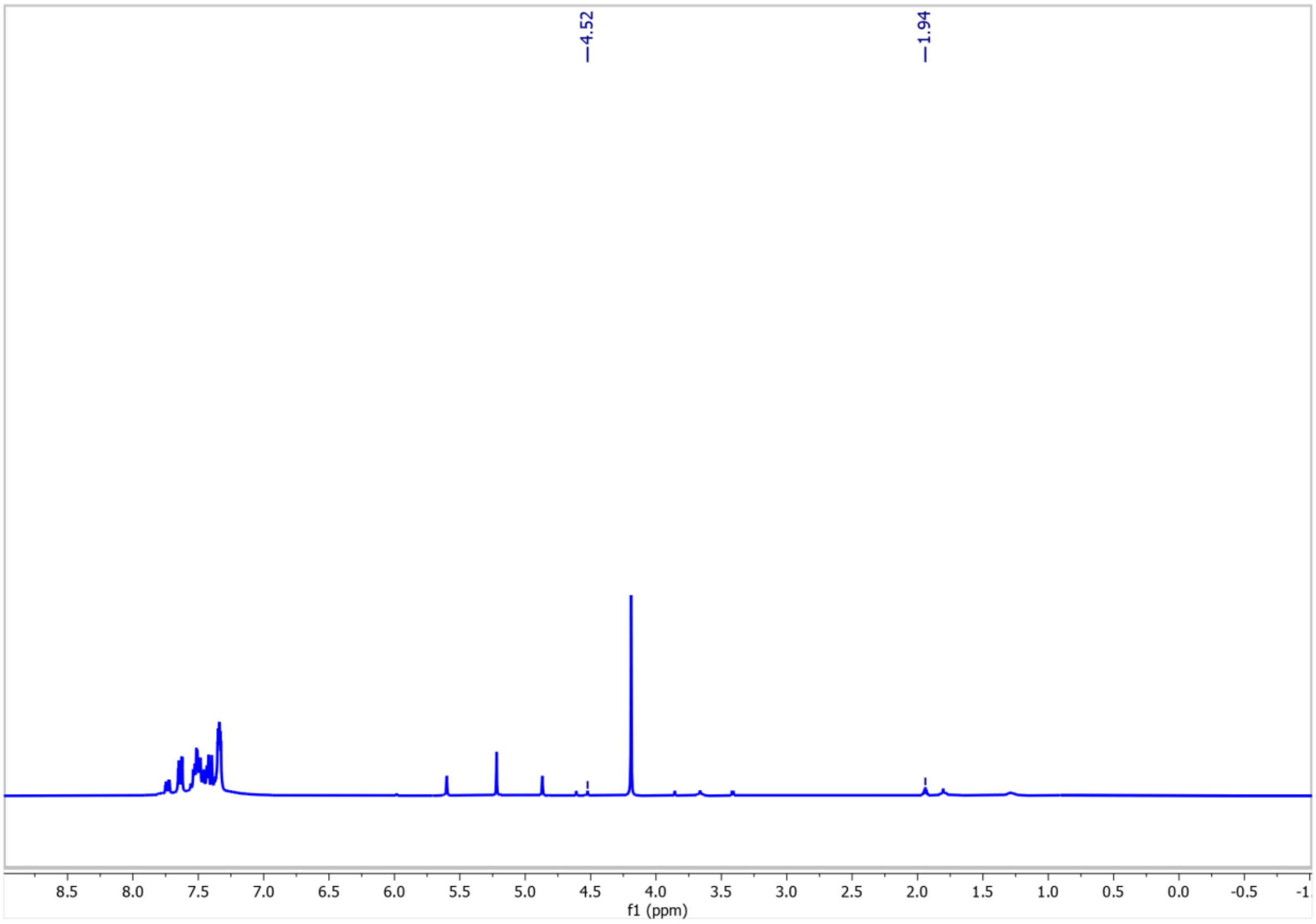

Figure S94: ^{1}H NMR spectrum after full conversion of $Ph_2(Cl)PO$ with 3 equiv. $PhSiH_3$ to $Ph_2(H)P$ using 2 mol% of compound **4** in CD_3CN.

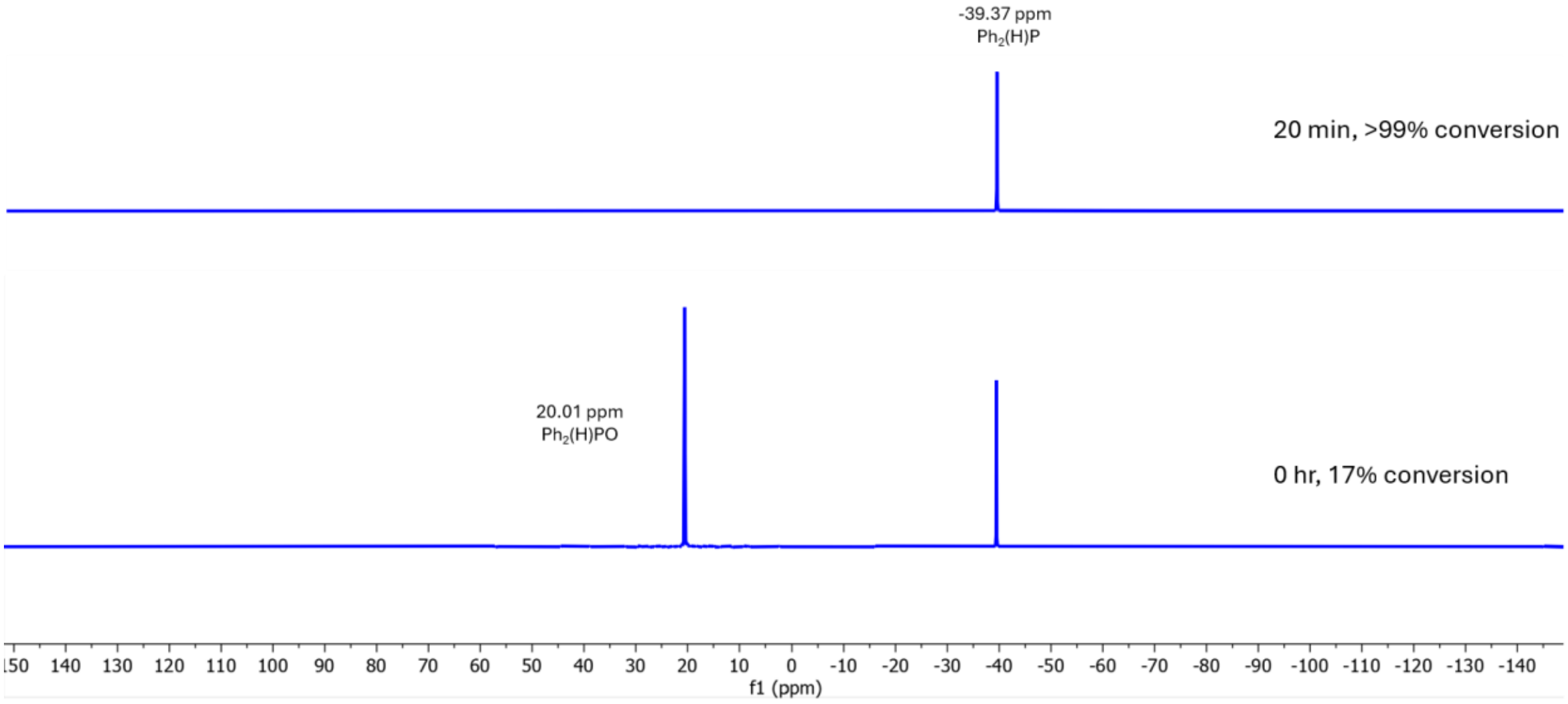

Figure S95: Stacked ^{31}P NMR spectra for the reduction of $Ph_2(H)PO$ with 3 equiv. $PhSiH_3$ to $Ph_2(H)P$ using 2 mol% of compound **4** in C_6D_6.

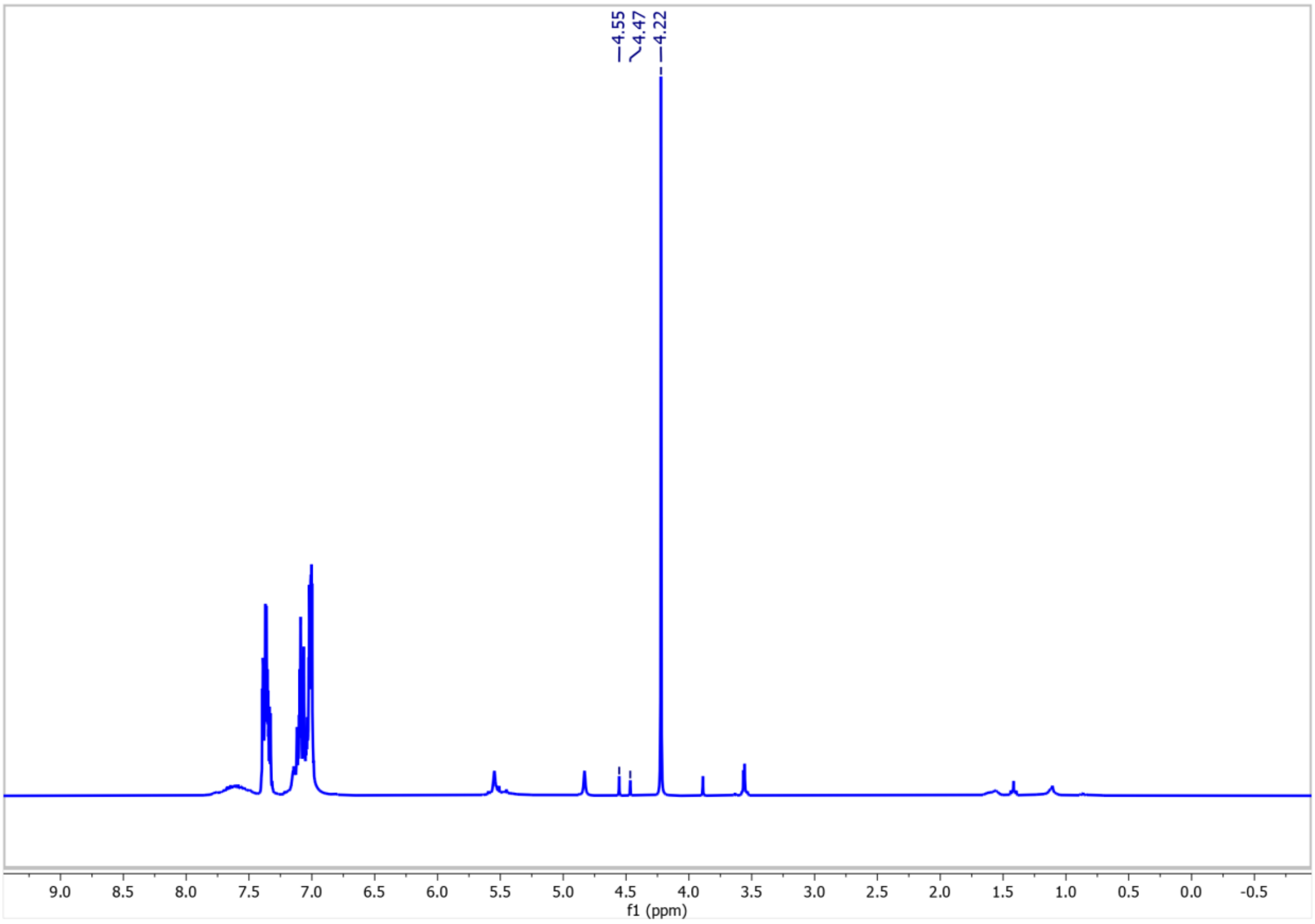

Figure S96: ^{1}H NMR spectrum after full conversion of $Ph_2(Cl)PO$ with 3 equiv. $PhSiH_3$ to $Ph_2(H)P$ using 2 mol% of compound **4** in CD_3CN.

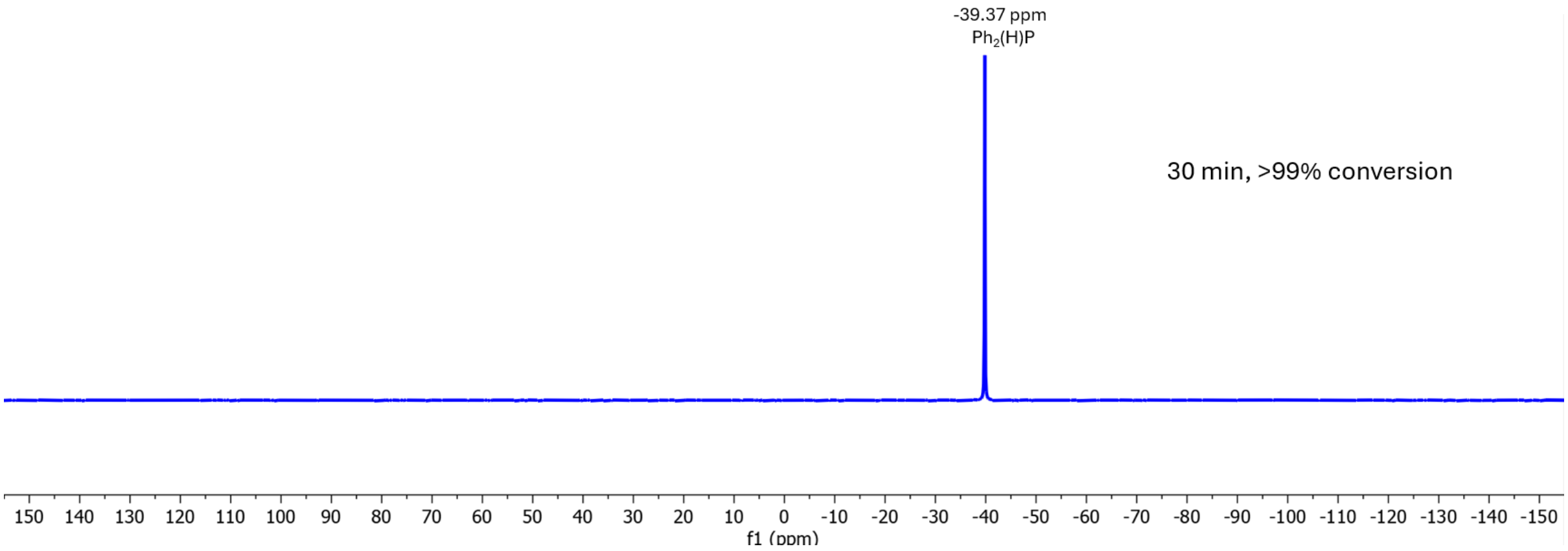

Figure S97: ^{31}P NMR spectrum for the reduction of $Ph_2(H)PO$ with 3 equiv. $PhSiH_3$ to $Ph_2(H)P$ using 1 mol% of compound **4** in C_6D_6.

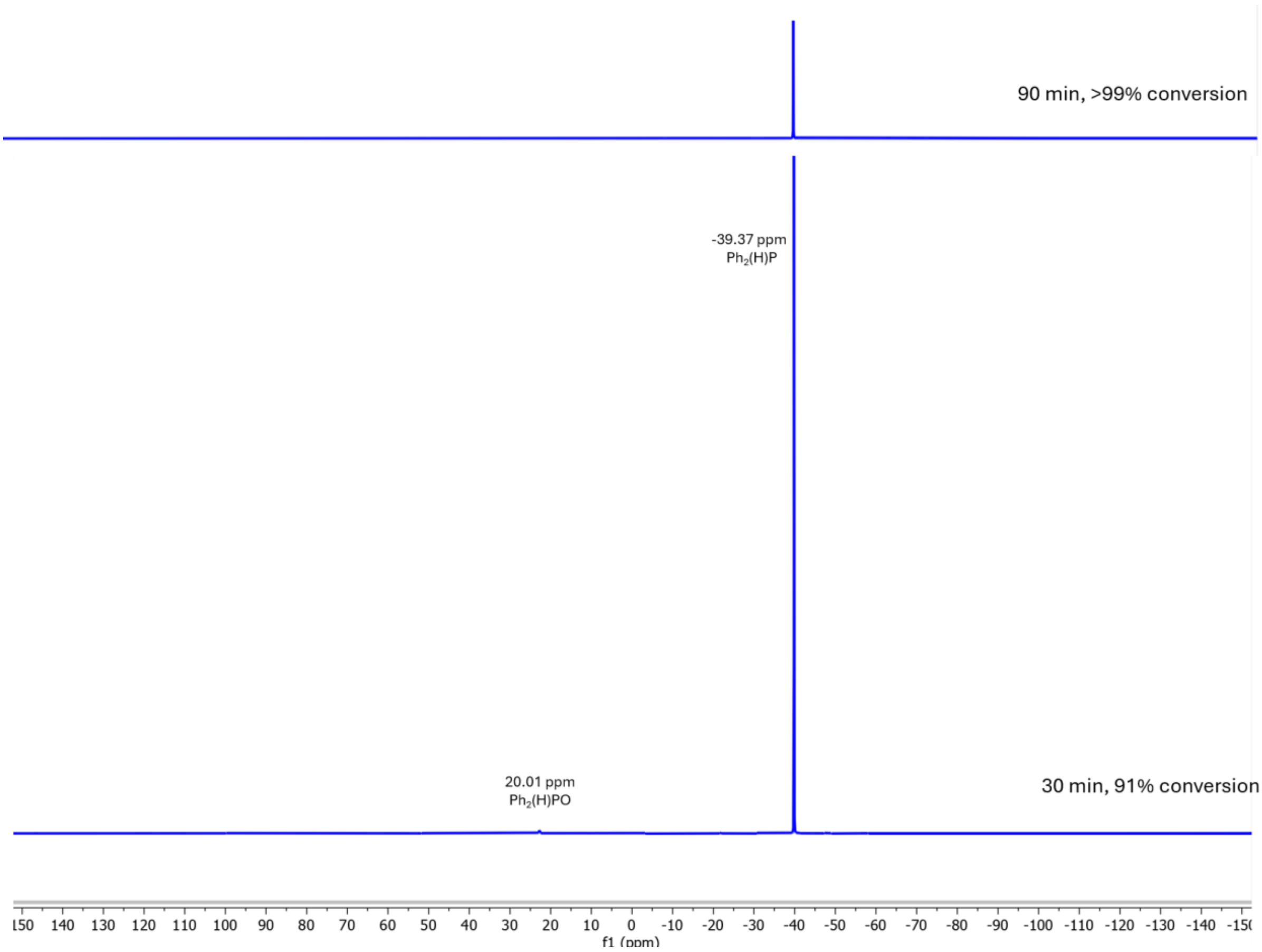

Figure S98: Stacked ^{31}P NMR spectra for the reduction of $Ph_2(H)PO$ with 3 equiv. $PhSiH_3$ to $Ph_2(H)P$ using 0.5 mol% of compound **4** in C_6D_6.

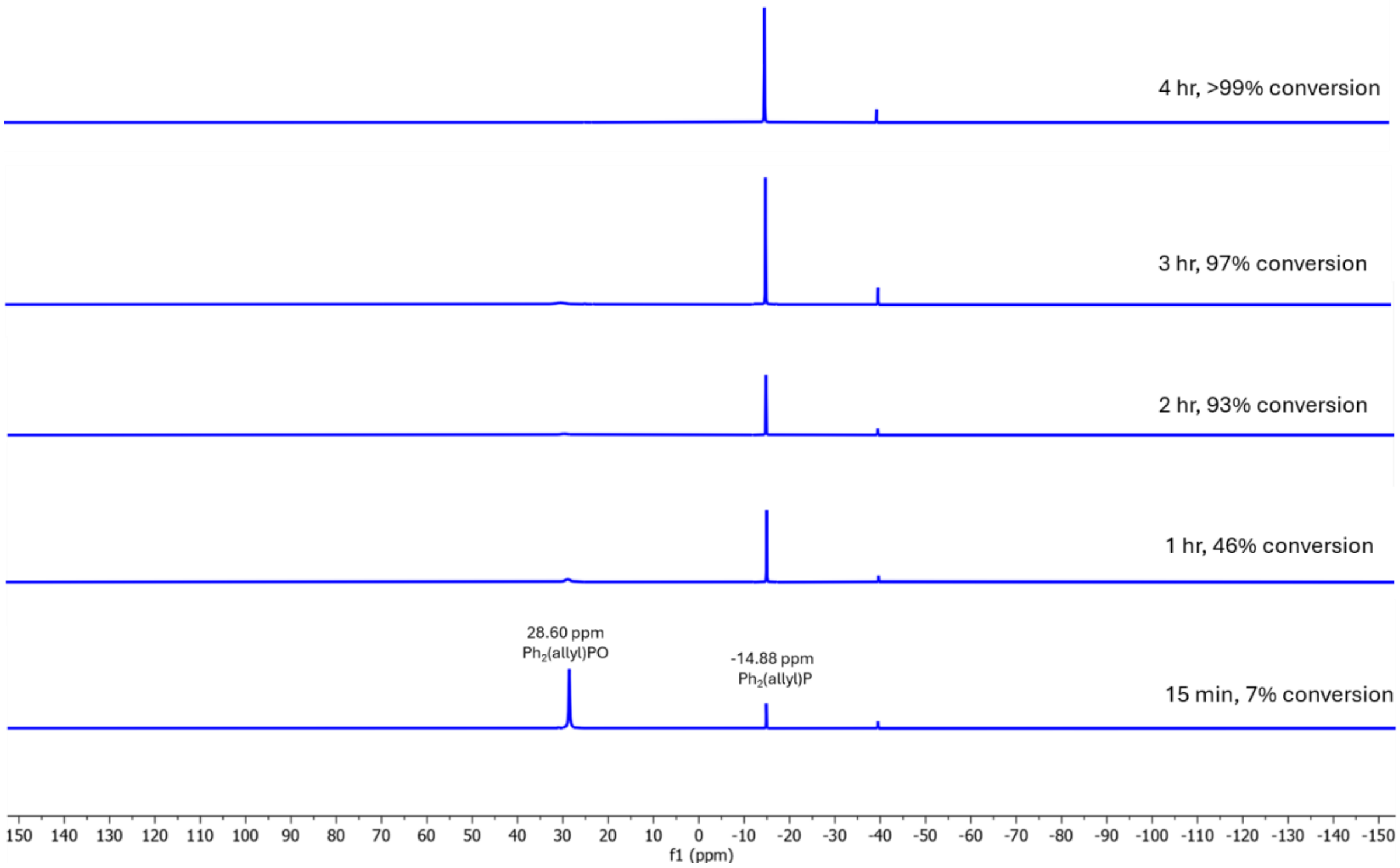

Figure S99: Stacked ^{31}P NMR spectra for the reduction of Ph_2(allyl)PO with 3 equiv. $PhSiH_3$ to Ph_2(allyl)P using 2 mol% of compound **4** in C_6D_6.

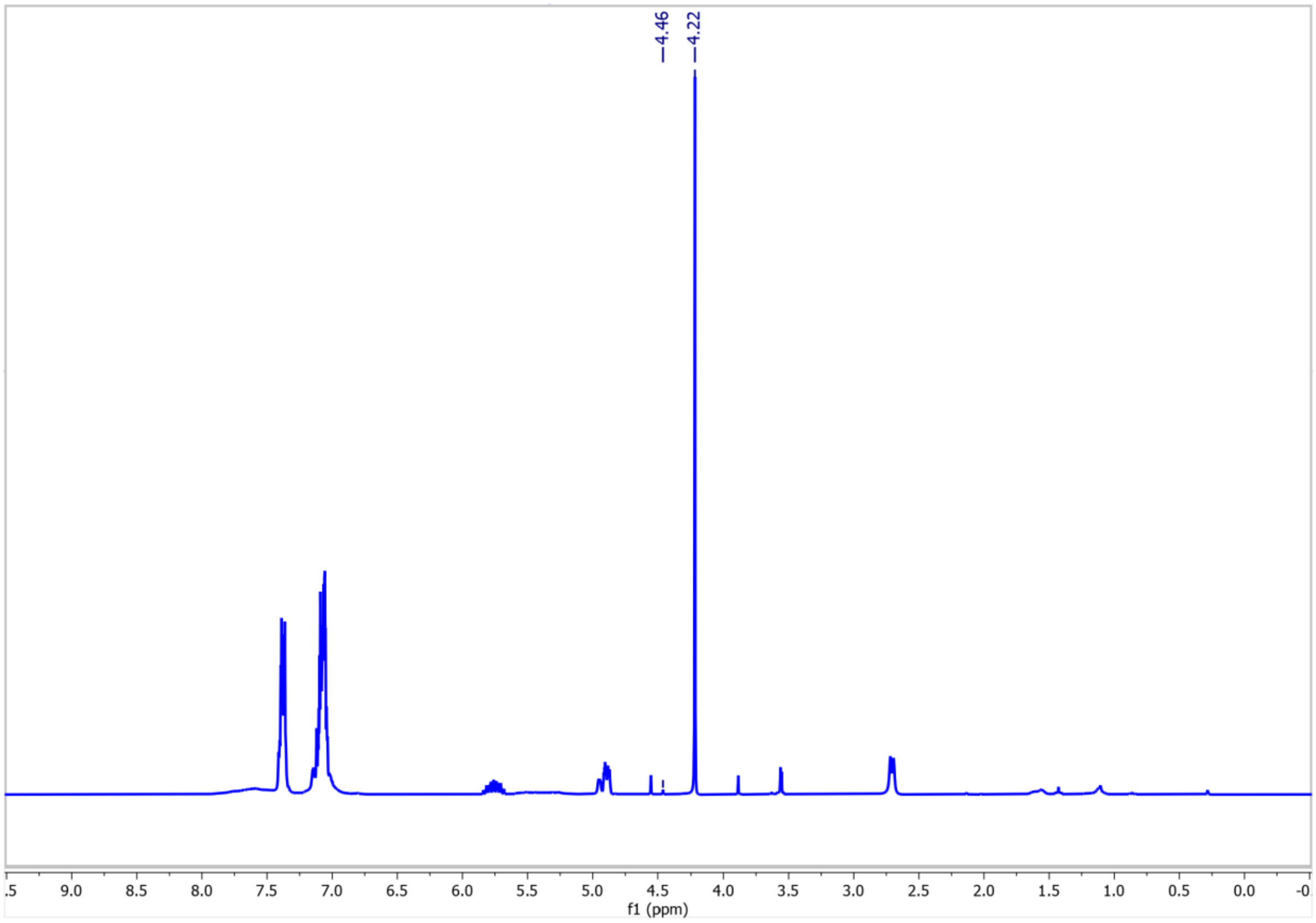

Figure S100: ^{1}H NMR spectrum after full conversion of Ph_2(allyl)PO with 3 equiv. $PhSiH_3$ to Ph_2(allyl)P using 2 mol% of compound **4** in C_6D_6.

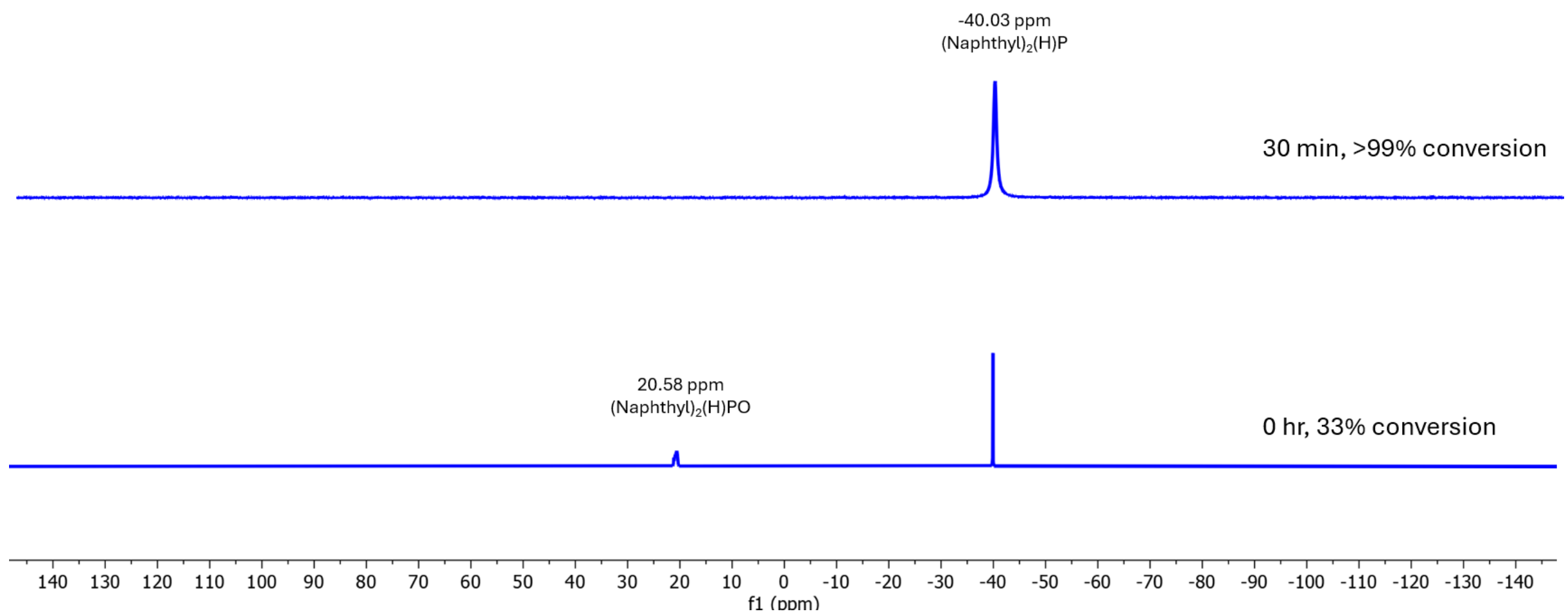

Figure S101: Stacked ^{31}P NMR spectra for the reduction of (naphthyl)$_2$(H)PO to (naphthyl)$_2$(H)P using 2 mol% of compound **4** in C_6D_6.

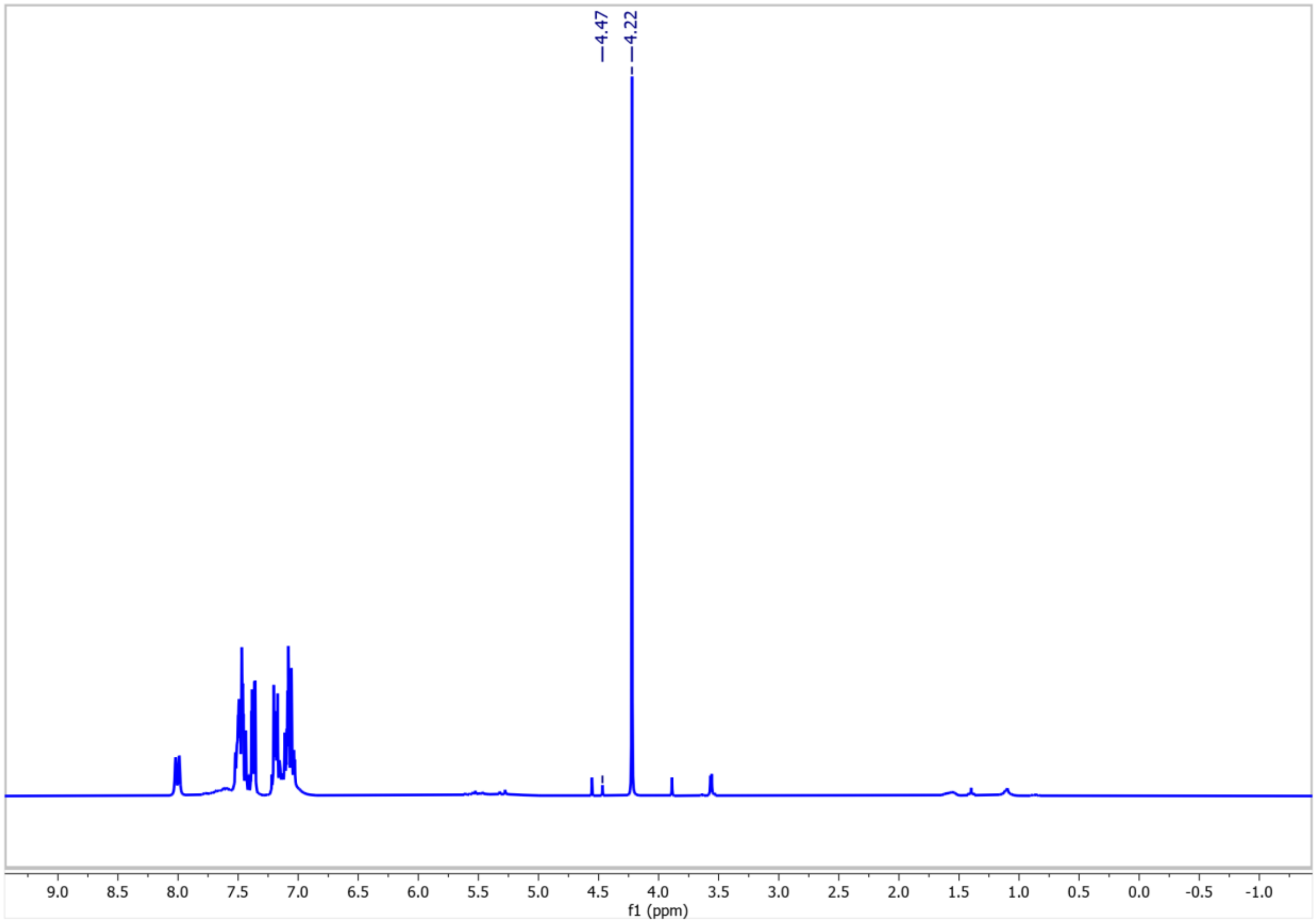

Figure S102: ^{1}H NMR spectrum after full conversion of (naphthyl)$_2$(H)PO with 3 equiv. $PhSiH_3$ to (naphthyl)$_2$(H)P using 2 mol% of compound **4** in C_6D_6.

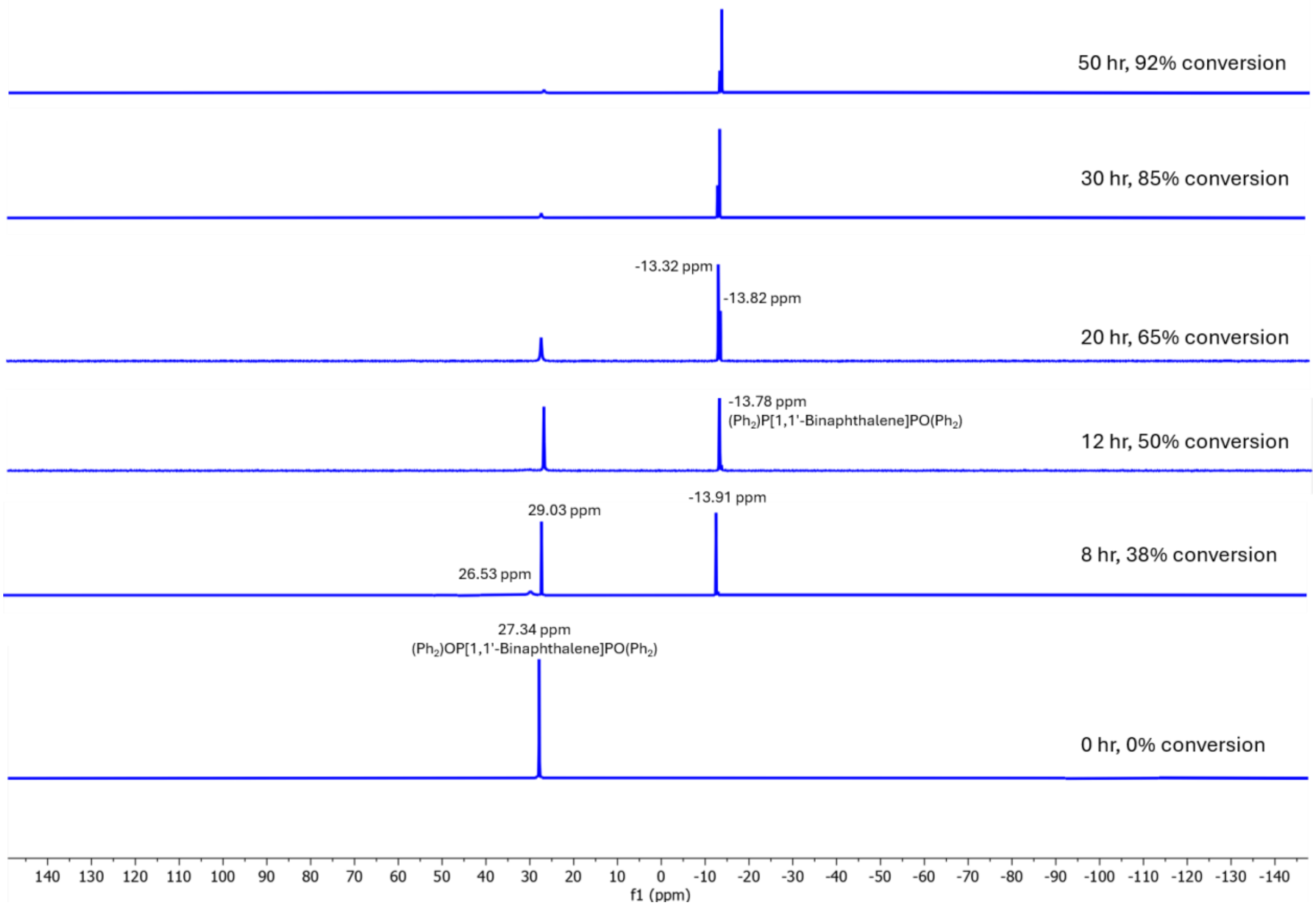

Figure S103: Stacked ^{31}P NMR spectra for the reduction of $(Ph_2)OP[1,1'$-binaphthalene$]PO(Ph_2)$ with 3 equiv. $PhSiH_3$ to $(Ph_2)P[1,1'$-binaphthalene$]P(Ph_2)$ using 2 mol% of compound **4** in C_6D_6.

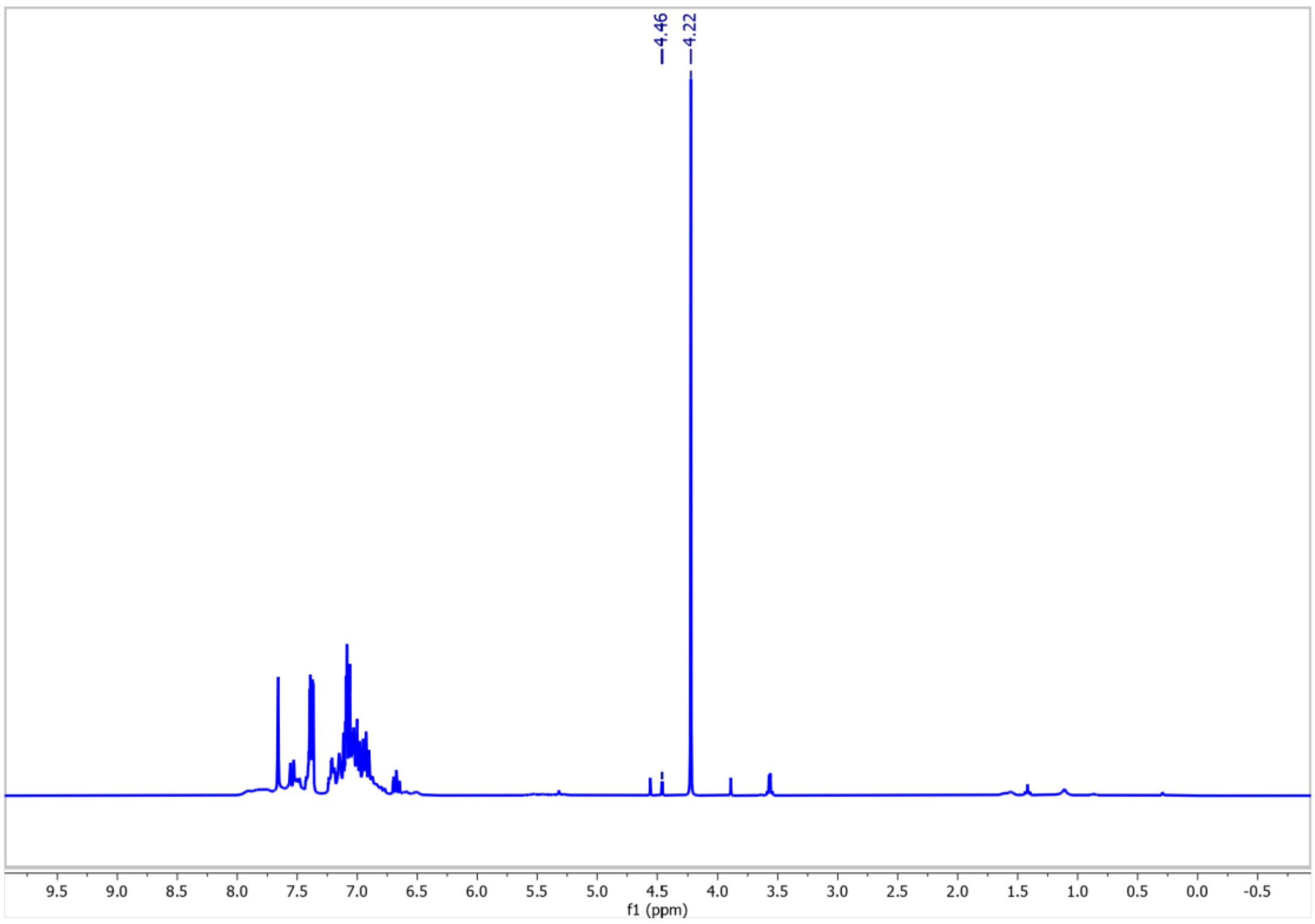

Figure S104: ^{1}H NMR spectrum after 50 hours for the conversion of $(naphthyl)_2(H)PO$ with 3 equiv. $PhSiH_3$ to $(naphthyl)_2(H)P$ using 2 mol% of compound **4** in C_6D_6.

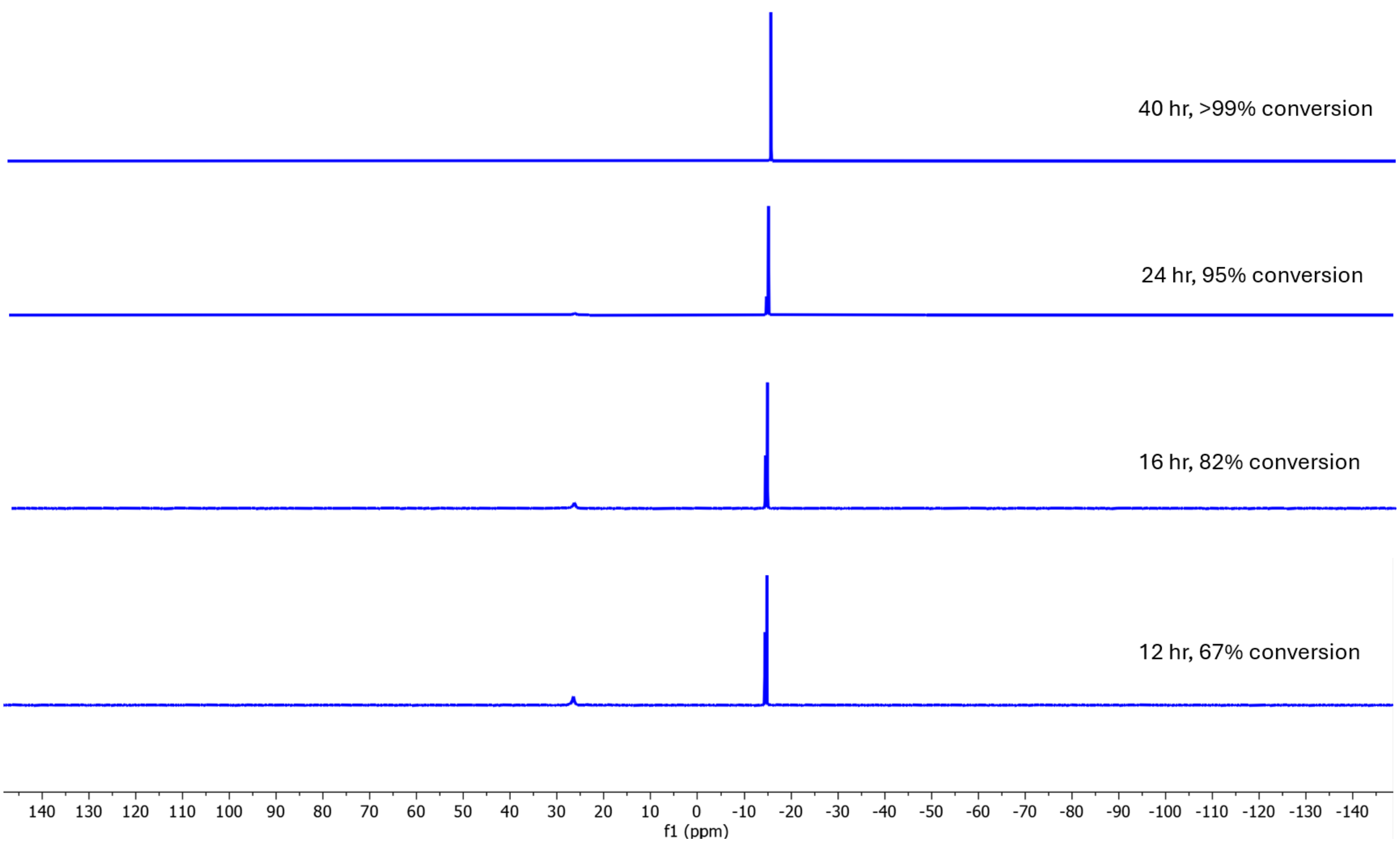

Figure S105: Stacked ^{31}P NMR spectra for the reduction of $(Ph_2)OP$[1,1'-binaphthalene]$PO(Ph_2)$ with 3 equiv. $PhSiH_3$ to $(Ph_2)P$[1,1'-binaphthalene]$P(Ph_2)$ using 10 mol% of compound **4** in C_6D_6.

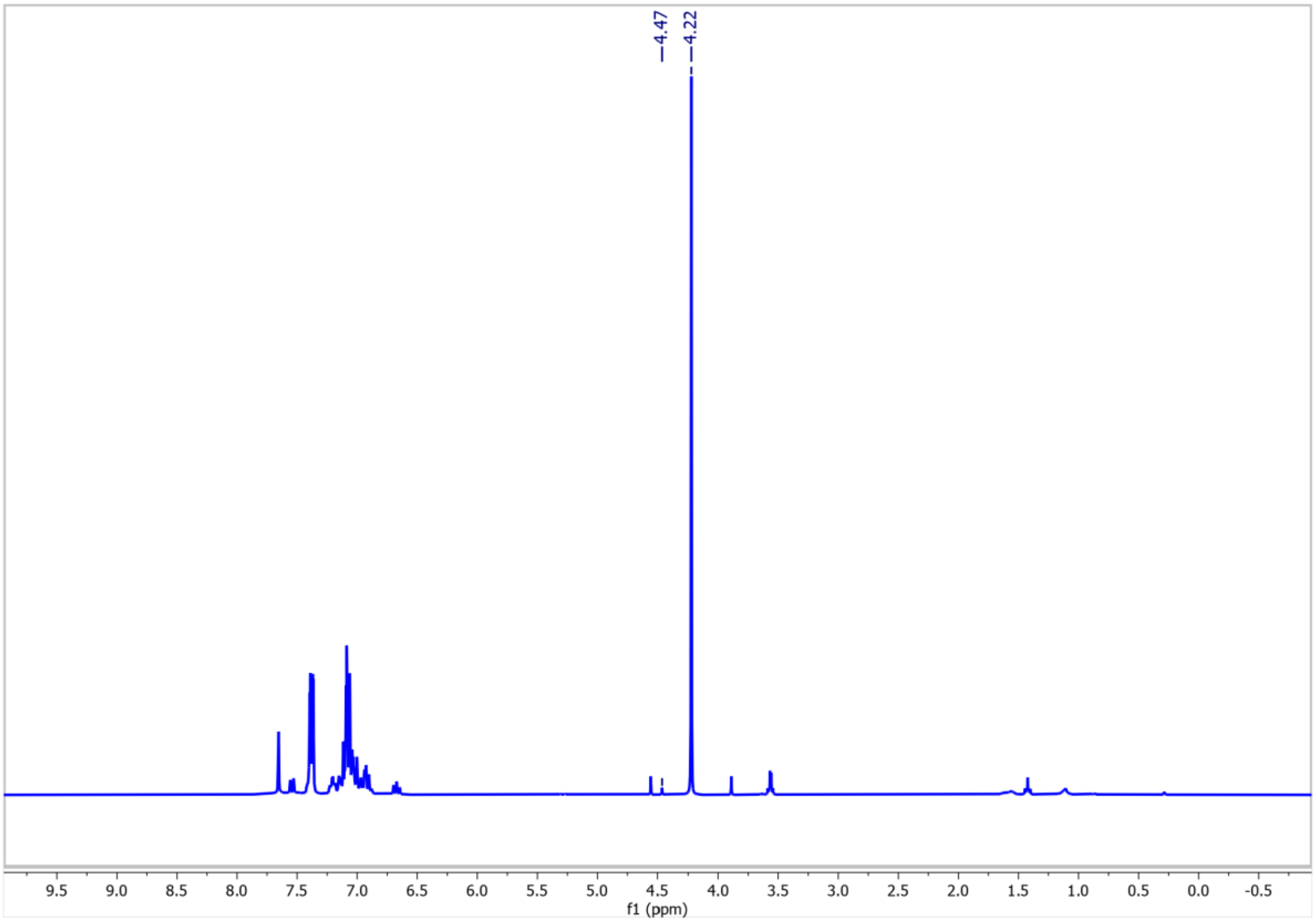

Figure S106: ^{1}H NMR spectrum after full conversion of $(Ph_2)OP$[1,1'-binaphthalene]$PO(Ph_2)$ with 3 equiv. $PhSiH_3$ to $(Ph_2)P$[1,1'-binaphthalene]$P(Ph_2)$ using 10 mol% of compound **4** in C_6D_6.

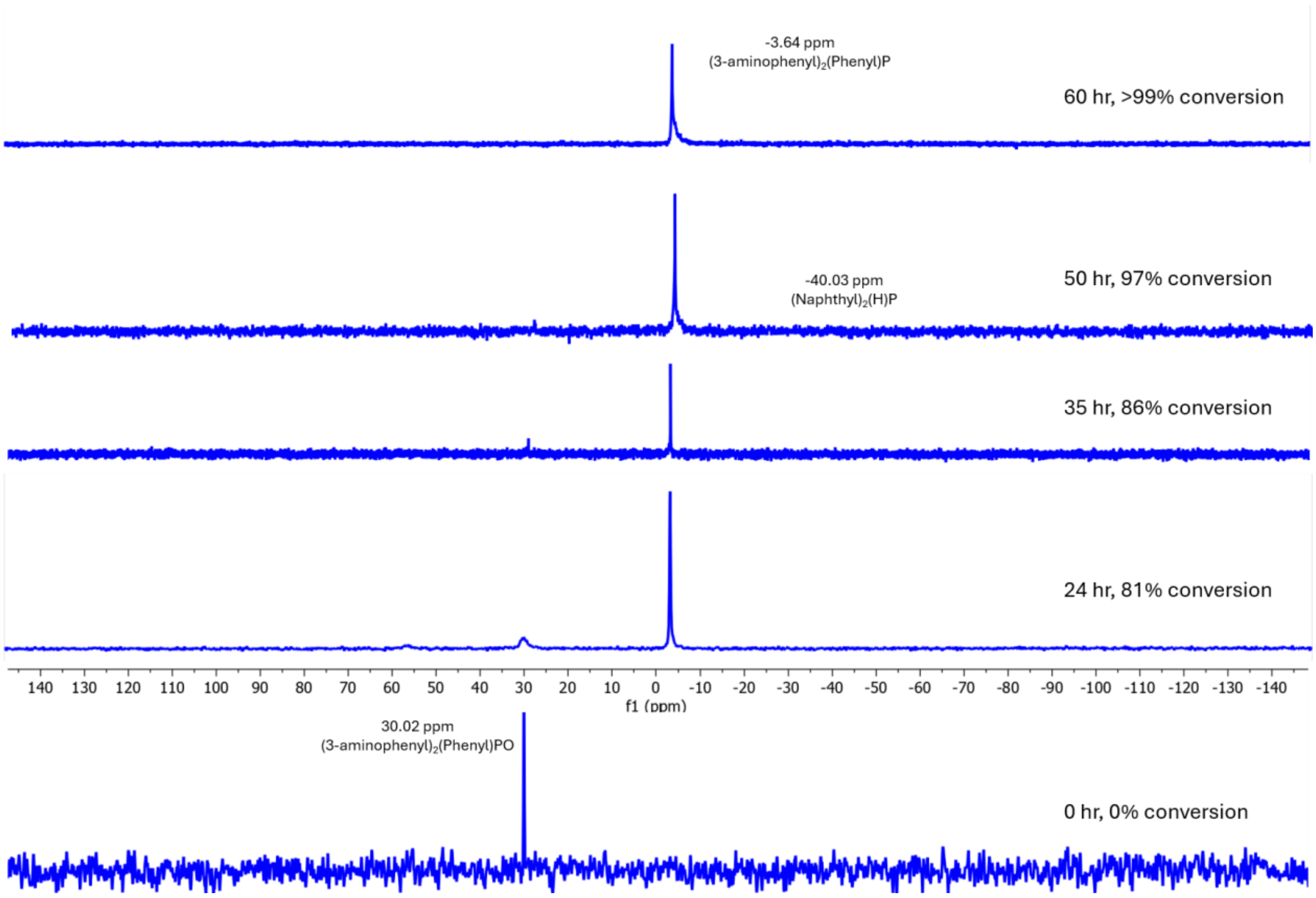

Figure S107: Stacked ^{31}P NMR spectra for the reduction of bis(3-aminophenyl)(phenyl)phosphine oxide with 3 equiv. $PhSiH_3$ to bis(3-aminophenyl)(phenyl)phosphine using 2 mol% of compound **4** in C_6D_6.

8. Single-crystal X-ray diffraction

Compound	**1**	**2**
Empirical formula	$C_{13}H_{10}BiF_3$	$C_{14}H_{13}BiCl_2F_3N_2$
Formula weight	432.19	546.14
Temperature/K	100(2)	100(2)
Crystal system	monoclinic	monoclinic
Space group	$P2_1/c$ (14)	$P2_1/n$ (14)
a/Å	10.653(6)	8.1554(10)
b/Å	10.772(2)	14.891(3)
c/Å	10.905(3)	13.998(2)
α/°	90	90
β/°	95.198(14)	105.840(5)
γ/°	90	90
Volume/Å^3	1246.2(8)	1635.4(4)
Z	4	4
ρ_{calc}g/cm^3	2.304	2.218
μ/mm^{-1}	14.158	11.133
F(000)	792	1020
Crystal size/mm	0.230×0.207×0.142	0.173×0.189×0.192
Crystal colour	colourless	colourless
Crystal shape	block	block
Radiation	MoK_α (λ=0.71073 Å)	MoK_α (λ=0.71073 Å)
2Θ range /°	5.33 to 67.34 (0.64 Å)	5.25 to 67.35 (0.64 Å)
Index ranges	$-16 \leq h \leq 14$	$-12 \leq h \leq 12$
	$-16 \leq k \leq 15$	$-22 \leq k \leq 23$
	$-15 \leq l \leq 15$	$-20 \leq l \leq 21$
Reflections collected	35285	68066
Independent reflections	4501	6001
	R_{int} = 0.0341	R_{int} = 0.0323
	R_{sigma} = 0.0233	R_{sigma} = 0.0154
Completeness to θ = 25.242°	99.9 %	100.0 %
Data/restraint/parameters	4501/0/154	6001 / 0 / 199
Goodness-of-fit on F^2	1.010	1.121
Final R indexes [I≥2σ(I)]	R_1 = 0.0173	R_1 = 0.0162
	wR_2 = 0.0316	wR_2 = 0.0302
Final R indexes [all data]	R_1 = 0.0235	R_1 = 0.0198
	wR_2 = 0.0331	wR_2 = 0.0308
Largest diff. peak/hole/ e Å^{-3}	0.79/−0.80	1.21/−0.70
CCDC	2546515	2546516

Compound	**3-py-PF$_6$**	**3-SbF$_6$**
Empirical formula	$C_{16}H_{15}BiClF_9N_3P$	$C_{13}H_{24}BiClF_9O_3Sb$
Formula weight	695.71	765.50
Temperature/K	100.00	100(2)
Crystal system	triclinic	monoclinic
Space group	$P\overline{1}$ (2)	$P2_1/c$ (14)
a/Å	7.8159(18)	9.480(4)
b/Å	9.533(3)	13.753(7)
c/Å	15.054(4)	16.103(6)
α/°	79.778(19)	90
β/°	89.074(9)	91.361(10)
γ/°	72.976(16)	90
Volume/Å^3	1054.7(5)	2098.9(15)
Z	2	4
ρ_{calc}g/cm^3	2.191	2.422
μ/mm^{-1}	8.647	9.876
F(000)	656	1432
Crystal size/mm	0.148 × 0.153 × 0.255	0.181×0.147×0.088
Crystal colour	white	colourless
Crystal shape	block	block
Radiation	MoK_α (λ=0.71073 Å)	MoK_α (λ=0.71073 Å)
2Θ range /°	5.50 to 50.72 (0.83 Å)	3.89 to 51.33 (0.82 Å)
Index ranges	$-9 \leq h \leq 9$<br>$-11 \leq k \leq 11$<br>$-18 \leq l \leq 18$	$-11 \leq h \leq 11$<br>$-16 \leq k \leq 16$<br>$-19 \leq l \leq 19$
Reflections collected	20293	28115
Independent reflections	3858<br>R_{int} = 0.0197<br>R_{sigma} = 0.0143	3880<br>R_{int} = 0.0930<br>R_{sigma} = 0.0593
Completeness to θ = 25.242°	99.8 %	99.9 %
Data/restraint/parameters	3858/0/280	3880/1139/455
Goodness-of-fit on F^2	1.063	1.043
Final *R* indexes [$I \geq 2\sigma(I)$]	R_1 = 0.0108<br>wR_2 = 0.0262	R_1 = 0.0585<br>wR_2 = 0.1525
Final *R* indexes [all data]	R_1 = 0.0110<br>wR_2 = 0.0262	R_1 = 0.0825<br>wR_2 = 0.1714
Largest diff. peak/hole/ e Å^{-3}	0.39/−0.46	1.79/−1.70
CCDC	2546517	2546519

Compound	**3-py-SbF$_6$**	**4**
Empirical formula	$C_{16}H_{15}BiClF_9N_3Sb$	$C_{21}H_{32}Bi_2F_{18}O_{15}S_4$
Formula weight	786.49	1412.6618376
Temperature/K	100.00	100(2)
Crystal system	triclinic	monoclinic
Space group	$P\overline{1}$ (2)	$P2_1/c$ (14)
a/Å	7.8318(14)	15.4130(7)
b/Å	9.5877(18)	14.3643(6)
c/Å	15.172(7)	19.2649(8)
α/°	80.631(15)	90
β/°	89.712(19)	105.810(2)
γ/°	74.810(8)	90
Volume/Å^3	1083.9(6)	4103.8(3)
Z	2	4
ρ_{calc}g/cm^3	2.410	2.215
μ/mm^{-1}	9.561	8.905
F(000)	728	2576
Crystal size/mm	0.405×0.153×0.094	0.146×0.252×0.303
Crystal colour	white	colourless
Crystal shape	block	block
Radiation	MoK_α (λ=0.71073 Å)	MoK_α (λ=0.71073 Å)
2Θ range /°	4.47 to 50.81 (0.83 Å)	3.95 to 56.71 (0.75 Å)
Index ranges	−9 ≤ h ≤ 9<br>−11 ≤ k ≤ 11<br>−18 ≤ l ≤ 18	−18 ≤ h ≤ 18<br>−17 ≤ k ≤ 17<br>−22 ≤ l ≤ 22
Reflections collected	39727	74093
Independent reflections	3985<br>R_{int} = 0.0339<br>R_{sigma} = 0.0153	7242<br>R_{int} = 0.0605<br>R_{sigma} = 0.0304
Completeness to θ = 25.242°	99.9 %	99.9
Data/restraint/parameters	3985/0/280	7242 / 1533 / 692
Goodness-of-fit on F^2	1.106	1.029
Final R indexes [I≥2σ(I)]	R_1 = 0.0123<br>wR_2 = 0.0297	R_1 = 0.0335<br>wR_2 = 0.0868
Final R indexes [all data]	R_1 = 0.0127<br>wR_2 = 0.0298	R_1 = 0.0382<br>wR_2 = 0.0894
Largest diff. peak/hole/ e Å^{-3}	0.60/−0.60	1.48/−1.14
CCDC	2546518	2546520

Compound	**4-py**	**5**
Empirical formula	$C_{14}H_{12}BiCl_2F_9N_2O_6S_2$	$C_{17}H_{24}BiF_{15}N_2O_{11}S_4$
Formula weight	819.26	1054.60
Temperature/K	100(2)	299(2)
Crystal system	orthorhombic	monoclinic
Space group	$P2_12_12_1$ (19)	$P2_1/c$ (14)
a/Å	9.3491(18)	19.4503(6)
b/Å	11.3189(18)	15.3155(5)
c/Å	22.821(4)	22.5722(7)
α/°	90	90
β/°	90	103.9410(10)
γ/°	90	90
Volume/Å^3	2415.0(7)	6526.0(4)
Z	4	8
ρ_{calc}g/cm^3	2.253	2.147
μ/mm^{-1}	7.799	5.799
F(000)	1552	4080
Crystal size/mm	0.238×0.114×0.109	0.086×0.091×0.459
Crystal colour	colourless	colourless
Crystal shape	block	plate
Radiation	MoK_α (λ=0.71073 Å)	MoK_α (λ=0.71073 Å)
2Θ range /°	4.02 to 52.70 (0.80 Å)	3.82 to 56.61 (0.75 Å)
Index ranges	$-11 \leq h \leq 11$<br>$-14 \leq k \leq 14$<br>$-25 \leq l \leq 28$	$-25 \leq h \leq 23$<br>$-20 \leq k \leq 20$<br>$-30 \leq l \leq 30$
Reflections collected	36758	223503
Independent reflections	4902<br>R_{int} = 0.0510<br>R_{sigma} = 0.0389	16209<br>R_{int} = 0.0712<br>R_{sigma} = 0.0310
Completeness to θ = 25.242°	100.0 %	99.9 %
Data/restraint/parameters	4902/0/325	16209 / 1557 / 938
Goodness-of-fit on F^2	1.031	1.022
Final R indexes [I≥2σ(I)]	R_1 = 0.0195<br>wR_2 = 0.0400	R_1 = 0.0314<br>wR_2 = 0.0653
Final R indexes [all data]	R_1 = 0.0221<br>wR_2 = 0.0406	R_1 = 0.0455<br>wR_2 = 0.0703
Largest diff. peak/hole/ e Å^{-3}	0.56/−0.68	1.58/−0.95
CCDC	2546521	2546522

Compound	**6**	**7**
Empirical formula	$C_9H_{18}BiF_9O_6P_2S_4$	$C_9H_{18}BiF_9O_6P_2S_2Se_2$
Formula weight	792.39	886.19
Temperature/K	293(2)	100(2)
Crystal system	triclinic	triclinic
Space group	$P\overline{1}$ (2)	$P\overline{1}$ (2)
a/Å	12.0062(5)	9.6797(15)
b/Å	12.0398(5)	12.1295(15)
c/Å	18.0223(7)	12.4104(17)
α/°	76.7470(10)	107.391(5)
β/°	79.9520(10)	91.686(5)
γ/°	73.3140(10)	113.337(3)
Volume/Å^3	2412.46(17)	1258.4(3)
Z	4	2
ρ_{calc}g/cm^3	2.182	2.339
μ/mm^{-1}	7.879	10.279
F(000)	1512	828
Crystal size/mm	0.044×0.045×0.086	0.054×0.067×0.094
Crystal colour	yellow	yellow
Crystal shape	block	block
Radiation	MoK_α (λ=0.71073 Å)	MoK_α (λ=0.71073 Å)
2Θ range /°	3.90 to 50.05 (0.84 Å)	3.89 to 51.36 (0.82 Å)
Index ranges	$-14 \leq h \leq 14$<br>$-14 \leq k \leq 14$<br>$-21 \leq l \leq 21$	$-11 \leq h \leq 11$<br>$-14 \leq k \leq 14$<br>$-15 \leq l \leq 15$
Reflections collected	52581	34404
Independent reflections	8518<br>R_{int} = 0.0713<br>R_{sigma} = 0.0484	4765<br>R_{int} = 0.1393<br>R_{sigma} = 0.0941
Completeness to θ = 25.242°	99.9 %	99.7 %
Data/restraint/parameters	8518 / 987 / 632	4765 / 1280 / 559
Goodness-of-fit on F^2	1.032	1.113
Final *R* indexes [$I \geq 2\sigma(I)$]	R_1 = 0.0442<br>wR_2 = 0.0933	R_1 = 0.0486<br>wR_2 = 0.1145
Final *R* indexes [all data]	R_1 = 0.0640<br>wR_2 = 0.1028	R_1 = 0.0909<br>wR_2 = 0.1209
Largest diff. peak/hole/ e Å^{-3}	3.03/−1.84	1.18/−0.98
CCDC	2546523	2546524

Compound	**8**	**9**
Empirical formula	$C_7H_{18}BiF_{15}N_0P_2S_2Sb_2$	$C_{15}H_{39}BiF_{15}NP_4S_4Sb_2$
Formula weight	965.75	1223.07
Temperature/K	100.00	100(2)
Crystal system	triclinic	monoclinic
Space group	$P\overline{1}$ (2)	$C2/c$ (15)
a/Å	9.3387(16)	23.6846(9)
b/Å	12.0369(17)	12.4920(5)
c/Å	12.897(2)	25.6844(9)
α/°	107.124(6)	90
β/°	104.606(6)	92.2470(10)
γ/°	100.549(5)	90
Volume/Å^3	1287.7(4)	7593.3(5)
Z	2	8
ρ_{calc}g/cm^3	2.491	2.140
μ/mm^{-1}	9.286	6.511
F(000)	884	4640
Crystal size/mm	0.118×0.202×0.291	0.065×0.069×0.135
Crystal colour	colourless	yellow
Crystal shape	needle	block
Radiation	MoK_α (λ=0.71073 Å)	MoK_α (λ=0.71073 Å)
2Θ range /°	4.85 to 67.02 (0.64 Å)	3.99 to 56.66 (0.75 Å)
Index ranges	−12 ≤ h ≤ 12	−29 ≤ h ≤ 31
	−18 ≤ k ≤ 18	−16 ≤ k ≤ 16
	−19 ≤ l ≤ 19	−34 ≤ l ≤ 34
Reflections collected	31990	94054
Independent reflections	8551	9451
	R_{int} = 0.0307	R_{int} = 0.0547
	R_{sigma} = 0.0310	R_{sigma} = 0.0273
Completeness to θ = 25.242°	99.4 %	99.9 %
Data/restraint/parameters	8551 / 0 / 268	9451 / 715 / 435
Goodness-of-fit on F^2	1.026	1.030
Final R indexes [I≥2σ(I)]	R_1 = 0.0280	R_1 = 0.0284
	wR_2 = 0.0722	wR_2 = 0.0557
Final R indexes [all data]	R_1 = 0.0326	R_1 = 0.0365
	wR_2 = 0.0747	wR_2 = 0.0594
Largest diff. peak/hole/ e Å^{-3}	2.50/−1.84	2.27/−2.27
CCDC	2546525	2546526

Compound	**10-py**	**11**
Empirical formula	$C_{32}H_{26}Bi_2F_{20}N_6Sb_2$	$C_{18}H_{46}Bi_2Cl_3F_{24}P_5Sb_3Se_5$
Formula weight	1536.05	2157.76
Temperature/K	100(2)	100(2)
Crystal system	monoclinic	monoclinic
Space group	$P2_1/c$ (14)	$P2_1/n$ (14)
a/Å	8.6757(6)	21.241(6)
b/Å	26.0555(16)	13.083(4)
c/Å	9.5127(6)	23.828(8)
α/°	90	90
β/°	106.548(2)	108.288(11)
γ/°	90	90
Volume/Å^3	2061.3(2)	6287(3)
Z	2	4
ρ_{calc}g/cm^3	2.475	2.280
μ/mm^{-1}	9.933	10.088
F(000)	1416	3940
Crystal size/mm	0.053×0.059×0.093	0.079×0.083×0.176
Crystal colour	colourless	yellow
Crystal shape	block	block
Radiation	MoK_α (λ=0.71073 Å)	MoK_α (λ=0.71073 Å)
2Θ range /°	4.73 to 56.64 (0.75 Å)	4.04 to 51.39 (0.82 Å)
Index ranges	$-11 \leq h \leq 11$<br>$-34 \leq k \leq 34$<br>$-12 \leq l \leq 12$	$-25 \leq h \leq 25$<br>$-15 \leq k \leq 15$<br>$-28 \leq l \leq 28$
Reflections collected	64552	78192
Independent reflections	5141<br>R_{int} = 0.0547<br>R_{sigma} = 0.0241	11895<br>R_{int} = 0.0768<br>R_{sigma} = 0.0603
Completeness to θ = 25.242°	100.0 %	99.9 %
Data/restraint/parameters	5141 / 0 / 280	11895 / 1242 / 683
Goodness-of-fit on F^2	1.337	1.031
Final *R* indexes [$I \geq 2\sigma(I)$]	R_1 = 0.0388<br>wR_2 = 0.0708	R_1 = 0.0615<br>wR_2 = 0.1719
Final *R* indexes [all data]	R_1 = 0.0441<br>wR_2 = 0.0721	R_1 = 0.0899<br>wR_2 = 0.1931
Largest diff. peak/hole/ e Å^{-3}	1.31/−1.87	1.98/−1.86
CCDC	2546527	2546528

Compound	**12**	**13**
Empirical formula	$C_{16}H_{40}Bi_2Cl_6F_{18}P_4Sb_2Se_4$	$C_{17}H_{36}BiF_{15}N_2O_8P_4S_4Se_4$
Formula weight	1888.36	1458.42
Temperature/K	100(2)	100(2)
Crystal system	triclinic	monoclinic
Space group	$P\overline{1}$ (2)	$P2_1/c$ (14)
a/Å	9.5406(10)	15.2399(6)
b/Å	10.8610(11)	21.7795(8)
c/Å	13.6086(13)	15.8714(7)
α/°	111.052(3)	90
β/°	104.442(3)	117.9260(10)
γ/°	97.459(3)	90
Volume/Å^3	1236.3(2)	4654.6(3)
Z	1	4
ρ_{calc}g/cm^3	2.536	2.081
μ/mm^{-1}	11.658	7.330
F(000)	864	2776
Crystal size/mm	0.06×0.12×0.18	0.047×0.084×0.1
Crystal colour	yellow	yellow
Crystal shape	needle	block
Radiation	MoK_α (λ=0.71073 Å)	MoK_α (λ=0.71073 Å)
2Θ range /°	4.14 to 50.05 (0.84 Å)	3.74 to 50.05 (0.84 Å)
Index ranges	$-10 \le h \le 11$	$-18 \le h \le 18$
	$-12 \le k \le 12$	$-25 \le k \le 25$
	$-16 \le l \le 16$	$-18 \le l \le 18$
Reflections collected	18623	93023
Independent reflections	4221	8209
	R_{int} = 0.1107	R_{int} = 0.0535
	R_{sigma} = 0.0952	R_{sigma} = 0.0246
Completeness to θ = 25.242°	96.5 %	100.0 %
Data/restraint/parameters	4221 / 547 / 309	8209 / 1445 / 643
Goodness-of-fit on F^2	1.074	1.063
Final *R* indexes [$I \ge 2\sigma(I)$]	R_1 = 0.0714	R_1 = 0.0401
	wR_2 = 0.1763	wR_2 = 0.1052
Final *R* indexes [all data]	R_1 = 0.0993	R_1 = 0.0494
	wR_2 = 0.1930	wR_2 = 0.1109
Largest diff. peak/hole/ e Å^{-3}	2.64/−2.66	3.01/−1.01
CCDC	2546529	2546530

Compound	**[Bi(CF$_3$)(thf)(NTf$_2$)$_2$(bipy)]**	**[Bi(CF$_3$)(thf)(NTf$_2$)$_2$(phen)]**
Empirical formula	$C_{19}H_{16}BiF_{15}N_4O_9S_4$	$C_{21}H_{16}BiF_{15}N_4O_9S_4$
Formula weight	1066.58	1090.60
Temperature/K	100(2)	100(2)
Crystal system	monoclinic	monoclinic
Space group	$P2_1/n$ (14)	$P2_1/n$ (14)
a/Å	10.4488(4)	10.4448(4)
b/Å	19.4651(7)	19.1669(6)
c/Å	16.1383(6)	16.8495(6)
α/°	90	90
β/°	96.113(2)	98.2180(10)
γ/°	90	90
Volume/Å^3	3263.7(2)	3338.5(2)
Z	4	4
ρ_{calc}g/cm^3	2.171	2.170
μ/mm^{-1}	5.797	5.670
F(000)	2048	2096
Crystal size/mm	0.073×0.081×0.097	0.050×0.081×0.105
Crystal colour	colourless	colourless
Crystal shape	block	block
Radiation	MoK_α	MoK_α
2Θ range /°	4.44 to 56.64 (0.75 Å)	4.33 to 59.95 (0.71 Å)
Index ranges	$-13 \leq h \leq 13$ $-25 \leq k \leq 25$ $-21 \leq l \leq 21$	$-14 \leq h \leq 14$ $-26 \leq k \leq 26$ $-23 \leq l \leq 23$
Reflections collected	99183	111454
Independent reflections	8121 R_{int} = 0.0647 R_{sigma} = 0.0292	9709 R_{int} = 0.0936 R_{sigma} = 0.0472
Completeness to θ = 25.242°	100.0 %	100.0 %
Data/restraint/parameters	8121 / 0 / 469	9709 / 0 / 487
Goodness-of-fit on F^2	1.041	1.035
Final R indexes [I≥2σ(I)]	R_1 = 0.0249 wR_2 = 0.0410	R_1 = 0.0335 wR_2 = 0.0495
Final R indexes [all data]	R_1 = 0.0355 wR_2 = 0.0444	R_1 = 0.0548 wR_2 = 0.0554
Largest diff. peak/hole/ e Å^{-3}	0.63/−0.75	0.85/−0.97
CCDC	2575824	2575825

9. DFT calculations

General remarks

All geometry optimizations were performed using the Gaussian16[18] suite of programs at the B3LYP+GD3/def2-TZVP level of theory.[19] The polarizable continuum model (PCM) has been used to model solvation in single point calculations.[20] Fluoride ion affinities (FIA) were computed with the $SiMe_3F$ anchor point,[21] and using the solvation correction approach for charged species.[22] Data for SbF_5, $[SbF_6]^-$, $SiMe_3F$, $[SiMe_3]^+$, and F^- has previously been reported.[5]

Table S1. Enthalpy (*ΔH*) and free enthalpy (*ΔG*), as determined by DFT calculations on the B3LYP+GD3/def2-TZVP level of theory in the gas phase.

compound	*ΔH* [hartree]	*ΔG* [hartree]
$Bi(CF_3)(OTf)_2$	-2475.965141	-2476.048933
$BiMe(OTf)_2$	-2178.103859	-2178.181955
$[Bi(CF_3)(bipy)]^{2+}$	-1047.312118	-1047.374202
$[Bi(CF_3)F(bipy)]^+$	-1147.611086	-1147.676365
$[Bi(CF_3)\mu_2\text{-}F(bipy)]_2^{2+}$	-2295.211974	-2295.322271

Table S2. Thermal correction to enthalpy (H_{corr}) for compounds without a solvation model.

compound	H_{corr} [hartree]
$[Bi(CF_3)(bipy)]^{2+}$	0.189796
$[Bi(CF_3)F(bipy)]^+$	0.193139
$[Bi(CF_3)\mu_2\text{-}F(bipy)]_2^{2+}$	0.387975

Table S3. Fluoride ion affinities (values in $kJ \cdot mol^{-1}$) calculated with the $SiMe_3F$ anchor point, using the B3LYP functional, the def2-tzvp basis set, and solvation by varying solvents modeled using the PCM approach.

Starting material	Product	FIA (B3LYP/def2-tzvp)				
		gas phase	pentane	Et_2O	THF	DCM
$[Bi(CF_3)(bipy)]^{2+}$	$[Bi(CF_3)F(bipy)]^+$	1116	717	453	366	346
$[Bi(CF_3)(bipy)]^{2+}$	0.5 $[Bi(CF_3)\mu_2\text{-}F(bipy)]_2^{2+}$	1102	732	481	397	378
SbF_5	$[SbF_6]^-$	524	446	390	371	366

Table S4. Cartesian coordinates (Å) of geometry-optimized structures, as determined by DFT calculations on the B3LYP+GD3/def2-TZVP level of theory in the gas phase.

$Bi(CF_3)(OTf)_2$

Bi	0.00259633	-0.45968236	-0.59549617
S	2.32306806	1.26030352	0.70327923
S	-2.74374127	0.09837875	0.47930522
F	1.07315056	-3.06528103	0.73023507
F	4.60391403	-0.08194852	0.59312257
F	-0.93875150	-2.64314762	1.43770075
F	-4.54363782	1.63240628	-0.66100004
F	-0.63364530	-3.44959827	-0.56167261
O	2.70035779	1.78231129	1.97163505
O	-1.31725887	0.42188105	0.93119998
F	4.60955812	1.80288980	-0.48332748
O	-3.61733420	-0.31035716	1.52056309
F	-2.48563769	2.20527922	-1.06722410
O	1.62468233	-0.10892887	0.82244043
O	1.59905888	2.06174002	-0.25808667
F	3.55312978	0.08628513	-1.30255969
O	-2.58867318	-0.72173670	-0.72850575
F	-3.37189433	2.62893426	0.87214086
C	3.88759730	0.73411837	-0.16986242
C	-0.13490462	-2.60282777	0.36990790
C	-3.33386910	1.76422090	-0.13102484

$BiMe(OTf)_2$

Bi	-0.00110074	-0.85453863	-0.35791067
S	2.38092949	1.16821468	0.53859993
S	-2.82167033	-0.17189631	0.53393670
F	4.56675024	-0.33082110	0.66794836
F	-4.64044031	1.12311387	-0.84895817
O	2.83262322	1.87569180	1.68971871
O	-1.41787448	0.24987791	0.95423600
F	4.67126979	1.29581522	-0.76518772
O	-3.70451788	-0.42578385	1.62002799
F	-2.59124852	1.67407975	-1.32247583
O	1.60366204	-0.10162855	0.92932255
O	1.71154677	1.84155155	-0.54398705
F	3.47933243	-0.46990891	-1.20860995
O	-2.65206464	-1.17295343	-0.52134161
F	-3.50911349	2.37434082	0.51908921
C	3.88283455	0.37041347	-0.23181023
C	-3.43797942	1.36211024	-0.33550928
C	-0.03245633	-2.63860094	0.98967013
H	0.89590415	-3.19525546	0.86535548
H	-0.10919116	-2.26169034	2.00779787
H	-0.89384243	-3.25459880	0.73867671

$[Bi(CF_3)(bipy)]^{2+}$

Bi	-1.07905101	0.00200396	-0.76927659
F	-0.43184769	-0.00076048	2.25470896
F	-2.26724772	1.09269172	1.84564053
F	-2.27045855	-1.08832221	1.84418158
N	0.65934647	1.35929893	-0.32479777

N	0.65533895	-1.36079654	-0.32608913
C	-1.56773630	0.00137161	1.54942107
C	1.63227265	-3.50789229	-0.02686786
H	1.52656172	-4.58346310	-0.06268692
C	1.81395519	0.73567284	0.03597488
C	1.81164572	-0.74092010	0.03573996
C	0.56780997	2.70309937	-0.35887876
H	-0.37956715	3.13433263	-0.65641409
C	0.55966220	-2.70429851	-0.36083290
H	-0.38881178	-3.13245594	-0.65931905
C	1.64316161	3.50321565	-0.02537314
H	1.54076238	4.57912621	-0.06059896
C	2.91360108	-1.51431975	0.37644863
H	3.84227245	-1.04625212	0.66558664
C	2.82515178	-2.90156159	0.34773157
H	3.68495828	-3.50204726	0.61562386
C	2.83447575	2.89301721	0.34792273
H	3.69640513	3.49071638	0.61522582
C	2.91862085	1.50549691	0.37603129
H	3.84611722	1.03442824	0.66406340

$[Bi(CF_3)F(bipy)]^+$

Bi	-1.13074282	-0.18837986	-0.70344558
F	-2.46624077	1.30205313	-0.23950901
F	-0.96354025	-2.28125477	1.53199310
F	-0.23433073	-0.36879565	2.27725865
F	-2.37034558	-0.72201156	2.10121641
N	0.41600991	1.48248187	-0.14540316
N	1.20321973	-1.08836144	-0.43454127
C	-1.19233820	-0.94524524	1.52326900
C	2.78227937	-2.87051834	-0.40273054
H	2.98324802	-3.92540039	-0.52376567
C	1.72581472	1.20047267	0.04890064
C	2.15974365	-0.20691395	-0.10524472
C	-0.04325541	2.73545883	-0.00072122
H	-1.10463776	2.87381242	-0.14964633
C	1.49792943	-2.38200429	-0.57825850
H	0.68067564	-3.04620397	-0.83155437
C	0.79280222	3.78330616	0.34169751
H	0.38352744	4.77734747	0.45046026
C	3.47448143	-0.62776250	0.08398442
H	4.25193599	0.07152460	0.34936772
C	3.78500715	-1.97159502	-0.06663463
H	4.80142069	-2.31143442	0.08081075
C	2.13906725	3.52026638	0.54014752
H	2.82347670	4.31340727	0.81030587
C	2.60470217	2.22184008	0.39356702
H	3.65011295	2.00988353	0.55312445

$[Bi(CF_3)\mu_2\text{-}F(bipy)]_2^{2+}$

Bi	-1.83993766	-0.23430260	-0.56615413
F	-0.43327601	1.27791970	0.14374412
F	-2.10881746	-2.26915526	1.70613047
F	-3.06089203	-0.40721613	2.30518706
F	-0.90114984	-0.59169451	2.38814247
N	-3.38133068	1.49878152	-0.11246022
N	-4.17322979	-1.07521376	-0.48631273

C	-1.99326423	-0.92460523	1.67272363
C	-5.77684467	-2.83805130	-0.54952350
H	-5.98507020	-3.88891039	-0.69182817
C	-4.70651571	1.22583749	-0.02782873
C	-5.13745601	-0.18039718	-0.20120070
C	-2.93650725	2.75682103	0.03662836
H	-1.86813452	2.89734514	-0.03161989
C	-4.47974185	-2.36512692	-0.65524369
H	-3.66117224	-3.03961822	-0.87490766
C	-3.79307617	3.81506876	0.27868754
H	-3.39389973	4.81278194	0.39209434
C	-6.46293731	-0.59046361	-0.07862731
H	-7.24479638	0.11468798	0.15636110
C	-6.78326730	-1.92932404	-0.25415379
H	-7.80992032	-2.25676076	-0.15829438
C	-5.15298422	3.55879982	0.36835978
H	-5.85577384	4.35978176	0.55560614
C	-5.60818228	2.25796457	0.21375025
H	-6.66542656	2.05491671	0.27939274
Bi	1.84001412	0.23429746	0.56633053
F	0.43328225	-1.27797115	-0.14333290
F	2.10860310	2.26904890	-1.70606913
F	3.06056263	0.40706923	-2.30518563
F	0.90080570	0.59157158	-2.38782805
N	3.38139714	-1.49877214	0.11249458
N	4.17327454	1.07527145	0.48606991
C	1.99303498	0.92450076	-1.67259126
C	5.77684615	2.83816035	0.54893904
H	5.98506269	3.88903306	0.69115597
C	4.70655756	-1.22578558	0.02760426
C	5.13748122	0.18047084	0.20084017
C	2.93659221	-2.75683660	-0.03644187
H	1.86823837	-2.89739869	0.03201160
C	4.47977521	2.36520164	0.65489405
H	3.66121900	3.03967968	0.87464913
C	3.79315437	-3.81506618	-0.27860459
H	3.39399335	-4.81280046	-0.39187950
C	6.46293160	0.59056991	0.07804467
H	7.24477442	-0.11457061	-0.15703104
C	6.78324920	1.92944843	0.25345563
H	7.80987682	2.25691167	0.15741644
C	5.15303569	-3.55875204	-0.36854945
H	5.85581940	-4.35971764	-0.55588810
C	5.60821546	-2.25789260	-0.21409130
H	6.66544000	-2.05480958	-0.27994350

10. Conductivity measurements

The conductivities of the solutions were determined by means of electrochemical impedance spectroscopy using a TSC 70 closed cell in combination with a Microcell HC cell stand (rhd instruments, Darmstadt, Germany). The cell consisted of a platinum crucible and a glass rod with four embedded platinum wires. The cell was filled with 70 µL of the solution within an argon-filled glovebox and was sealed airtight. Outside the glovebox the cell was heated to 25 °C using an Eurotherm 2416 temperature controller (accuracy ±0.1 °C) and held at this temperature for another 10 minutes before any measurements were performed. The impedance measurements were carried out using an Alpha-A impedance analyzer (Novocontrol Technologies, Montabaur, Germany) equipped with a ZG2-Interface in a frequency range from 1 MHz to 1 Hz with an AC amplitude of 10 mV_{rms}. The cell constant K was determined using a 0.1 $mol{\cdot}L^{-1}$ KCl standard solution (Hanna Instruments, Vöhringen, Germany) with a known conductivity at 25 °C. For each solution two cells were prepared and three impedance spectra were recorded for each cell. The impedance spectra were then fitted by using the software RelaxIS (rdh instruments, Darmstadt, Germany) and an equivalent circuit consisting of a resistance in series to a constant phase element (CPE). In this circuit, the resistance describes the bulk ionic resistance R_{bulk} of the solution whereas the CPE accounts for electrode polarization due to double layer formation. Figure S108 shows an exemplary impedance spectrum in the Nyquist-Plot.

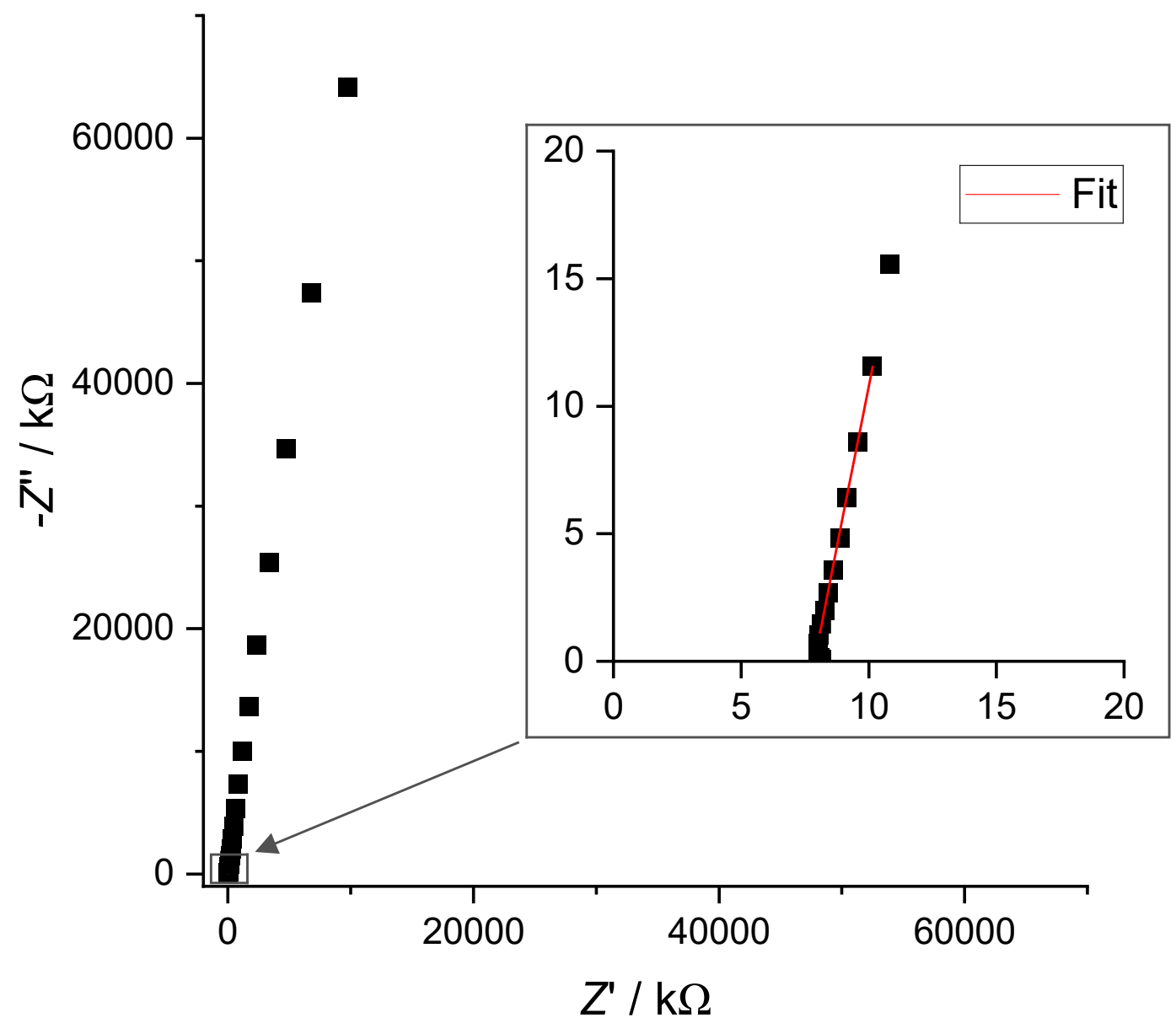

Figure S108: Nyquist-Plot of the impedance recorded for the solution of 5 (10 $mmol{\cdot}L^{-1}$) in acetonitrile. Measured at 25 °C in a frequency range from 1 MHz to 1 Hz and fitted with a resistance and a CPE in series.

Extracting the bulk resistances R_{bulk} allows the calculation of the conductivities σ of each sample by means of equation 4.

$$\sigma = R_{\mathrm{bulk}} \cdot K \qquad \text{(eq. 4)}$$

The conductivity of 10 mmol·L^{-1} solutions of $Bi(CF_3)_3$, $BiPh_2(OTf)$, compound **4** and compound **5** in acetonitrile was measured. Reference measurements were carried out on the pure solvent. Table S5 contains the average values of the measured conductivities of all samples.

Table S5: Averaged conductivities of all samples measured at 25 °C using 10 mmol·L^{-1} solutions of the samples in acetonitrile (or the solvent itself for comparison).

Sample	Chemical formula	Conductivity / µS · cm^{-1}
Acetonitrile	CH_3CN	2.6
$Bi(CF_3)_3$	10 mM $Bi(CF_3)_3$ in CH_3CN	4.1
$BiPh_2(OTf)$	10 mM $Bi(C_6H_5)_2OSO_2CF_3$ in CH_3CN	771
**4**	10 mM $BiCF_3(C_4H_8O)_2(OSO_2CF_3)_2$ in CH_3CN	900
**5**	10 mM $BiCF_3(C_4H_8O)_3(O_2SCF_3NCF_3SO_2)_2$ in CH_3CN	2170

11. References

1 G. R. Fulmer, A. J. M. Miller, N. H. Sherden, H. E. Gottlieb, A. Nudelman, B. M. Stoltz, J. E. Bercaw and K. I. Goldberg, *Organometallics*, 2010, **29**, 2176.

2 C. B. Hubschle, G. M. Sheldrick and B. Dittrich, *J. Appl. Cryst.*, 2011, **44**, 1281.

3 O. V. Dolomanov, L. J. Bourhis, R. J. Gildea, J. A. K. Howard and H. Puschmann, *J. Appl. Cryst.*, 2009, **42**, 339.

4 a) G. M. Sheldrick, *Acta crystallographica. Section A, Foundations of crystallography*, 2008, **64**, 112; b) G. M. Sheldrick, *Acta crystallographica. Section C, Structural chemistry*, 2015, **71**, 3;

5 J. Schwarzmann, T. Z. Kirsch, B. Narz and C. Lichtenberg, *Angew. Chem. Int. Ed.*, 2025, e16140.

6 a) A.-M. Preda, M. Krasowska, L. Wrobel, P. Kitschke, P. C. Andrews, J. G. MacLellan, L. Mertens, M. Korb, T. Rüffer, H. Lang, A. A. Auer and M. Mehring, *Beilstein J. Org. Chem.*, 2018, **14**, 2125; b) K. Oberdorf, A. Hanft, J. Ramler, I. Krummenacher, F. M. Bickelhaupt, J. Poater and C. Lichtenberg, *Angew. Chem. Int. Ed.*, 2021, **60**, 6441;

7 a) U. Mayer, V. Gutmann and W. Gerger, *Monatsh. Chem.*, 1975, **106**, 1235; b) M. A. Beckett and G. C. Strickland, *Polymer*, 1996, **37**, 4629;

8 J. Ramler and C. Lichtenberg, *Chem. Eur. J.*, 2020, **26**, 10250.

9 S. S. Chitnis, N. Burford, A. Decken and M. J. Ferguson, *Inorg. Chem.*, 2013, **52**, 7242.

10 S. Mazières, C. Le Roux, M. Peyronneau, H. Gornitzka and N. Roques, *Eur. J. Inorg. Chem.*, 2004, **2004**, 2823.

11 T. A. Hanna, G. Keitany, C. Ibarra, R. D. Sommer and A. L. Rheingold, *Polyhedron*, 2001, **20**, 2451.

12 M. Mantina, A. C. Chamberlin, R. Valero, C. J. Cramer and D. G. Truhlar, *J. Phys. Chem. A*, 2009, **113**, 5806.

13 L. J. Farrugia, F. J. Lawlor and N. C. Norman, *J. Chem. Soc., Dalton Trans.*, 1995, 1163.

14 A. Cogne, A. Grand, J. Laugier, J. B. Robert and L. Wiesenfeld, *J. Am. Chem. Soc.*, 1980, **102**, 2238.

15 D. Hérault, D. H. Nguyen, D. Nuel and G. Buono, *Chem. Soc. Rev.*, 2015, **44**, 2508.

16 J. Moineau, M. Granier and G. F. Lanneau, *Langmuir : the ACS journal of surfaces and colloids*, 2004, **20**, 3202.

17 S. Solyntjes, J. Bader, B. Neumann, H.-G. Stammler, N. Ignat'ev and B. Hoge, *Chem. Eur. J.*, 2017, **23**, 1557.

18 *M. J. Frisch, G. W. Trucks, H. B. Schlegel, G. E. Scuseria, M. A. Robb, J. R. Cheeseman, G. Scalmani, V. Barone, G. A. Petersson, H. Nakatsuji, X. Li, M. Caricato, A. V. Marenich, J. Bloino, B. G. Janesko, R. Gomperts, B. Mennucci, H. P. Hratchian, J. V. Ortiz, A. F. Izmaylov, J. L. Sonnenberg, D. Williams-Young, F. Ding, F. Lipparini, F. Egidi, J. Goings, B. Peng, A. Petrone, T. Henderson, D. Ranasinghe, V. G. Zakrzewski, J. Gao, N. Rega, G. Zheng, W. Liang, M. Hada, M. Ehara, K. Toyota, R. Fukuda, J. Hasegawa, M. Ishida, T. Nakajima, Y. Honda, O. Kitao, H. Nakai, T. Vreven, K. Throssell, Montgomery, J. A., Jr., J. E. Peralta, F. Ogliaro, M. J. Bearpark, J. J. Heyd, E. N. Brothers, K. N. Kudin, V. N. Staroverov, T. A. Keith, R. Kobayashi, J. Normand, K. Raghavachari, A. P. Rendell, J. C. Burant, S. S. Iyengar, J. Tomasi, M. Cossi, J. M. Millam, M. Klene, C. Adamo, R. Cammi, J. W. Ochterski, R. L. Martin, K. Morokuma, O. Farkas, J. B. Foresman, D. J. Fox, Gaussian 16, Revision A.03, 2016.*

19 a) A. D. Becke, *J. Chem. Phys.*, 1993, **98**, 5648; b) C. Lee, W. Yang and R. G. Parr, *Phys. Rev. B Condens. Matter*, 1988, **37**, 785; c) S. H. Vosko, L. Wilk and M. Nusair, *Can. J. Phys.*, 1980, **58**, 1200; d) P. J. Stephens, F. J. Devlin, C. F. Chabalowski and M. J. Frisch, *J. Phys. Chem.*, 1994, **98**, 11623; e) S. Grimme, J. Antony, S. Ehrlich and H. Krieg, *J. Chem. Phys.*, 2010, **132**, 154104; f) F. Weigend and R. Ahlrichs, *Phys. Chem. Chem. Phys.*, 2005, **7**, 3297;

20 J. Tomasi, B. Mennucci and R. Cammi, *Chem. Rev.*, 2005, **105**, 2999.

21 H. Böhrer, N. Trapp, D. Himmel, M. Schleep and I. Krossing, *Dalton Trans.*, 2015, **44**, 7489.

22 P. Erdmann, M. Schmitt, L. M. Sigmund, F. Krämer, F. Breher and L. Greb, *Angew. Chem. Int. Ed.*, 2024, **63**, e202403356.
